# Developmental roles of 21 *Drosophila *transcription factors are determined by quantitative differences in binding to an overlapping set of thousands of genomic regions

**DOI:** 10.1186/gb-2009-10-7-r80

**Published:** 2009-07-23

**Authors:** Stewart MacArthur, Xiao-Yong Li, Jingyi Li, James B Brown, Hou Cheng Chu, Lucy Zeng, Brandi P Grondona, Aaron Hechmer, Lisa Simirenko, Soile VE Keränen, David W Knowles, Mark Stapleton, Peter Bickel, Mark D Biggin, Michael B Eisen

**Affiliations:** 1 Genomics Division, Lawrence Berkeley National Laboratory, Cyclotron Road MS 84-181, Berkeley, CA 94720, USA; 2 Howard Hughes Medical Institute, University of California Berkeley, Berkeley, CA 94720, USA; 3 Department of Statistics, University of California Berkeley, Berkeley, CA 94720, USA; 4 Life Sciences Division, Lawrence Berkeley National Laboratory, Cyclotron Road MS 84-181, Berkeley, CA 94720, USA; 5 Department of Molecular and Cell Biology, University of California Berkeley, Berkeley, CA 94720, USA; 6 Current address: Cancer Research UK Cambridge Research Institute, Li Ka Shing Centre, Robinson Way, Cambridge, CB2 0RE, UK

## Abstract

**Background:**

We previously established that six sequence-specific transcription factors that initiate anterior/posterior patterning in *Drosophila *bind to overlapping sets of thousands of genomic regions in blastoderm embryos. While regions bound at high levels include known and probable functional targets, more poorly bound regions are preferentially associated with housekeeping genes and/or genes not transcribed in the blastoderm, and are frequently found in protein coding sequences or in less conserved non-coding DNA, suggesting that many are likely non-functional.

**Results:**

Here we show that an additional 15 transcription factors that regulate other aspects of embryo patterning show a similar quantitative continuum of function and binding to thousands of genomic regions *in vivo*. Collectively, the 21 regulators show a surprisingly high overlap in the regions they bind given that they belong to 11 DNA binding domain families, specify distinct developmental fates, and can act via different *cis*-regulatory modules. We demonstrate, however, that quantitative differences in relative levels of binding to shared targets correlate with the known biological and transcriptional regulatory specificities of these factors.

**Conclusions:**

It is likely that the overlap in binding of biochemically and functionally unrelated transcription factors arises from the high concentrations of these proteins in nuclei, which, coupled with their broad DNA binding specificities, directs them to regions of open chromatin. We suggest that most animal transcription factors will be found to show a similar broad overlapping pattern of binding *in vivo*, with specificity achieved by modulating the amount, rather than the identity, of bound factor.

## Abstract 2

Distinct developmental fates in *Drosophila melanogaster* are specified by quantitative differences in transcription factor occupancy on a common set of bound regions.

## Background

Sequence-specific transcription factors regulate spatial and temporal patterns of mRNA expression in animals by binding in different combinations to *cis*-regulatory modules (CRMs) located generally in the non-protein coding portions of the genome (reviewed in [1-4]). Most of these factors recognize short, degenerate DNA sequences that occur multiple times in every gene locus. Yet only a subset of these recognition sequences are thought to be functional targets [1,5,6]. Because we do not sufficiently understand the rules determining DNA binding *in vivo *or the transcriptional output that results from particular combinations of bound factors, we cannot at present predict the locations of CRMs or patterns of gene expression from genome sequence and *in vitro *DNA binding specificities alone.

To address this challenge, the Berkeley *Drosophila *Transcription Network Project (BDTNP) has initiated an interdisciplinary analysis of the network controlling transcription in the *Drosophila melanogaster *blastoderm embryo [7-12]. Only 40 to 50 sequence-specific regulators provide the spatial and temporal patterning information to the network, making it particularly tractable for system-wide analyses [13-15].

The factors are arranged into several temporal cascades and can be grouped into classes based on the aspect of patterning they control and their time of action (Table 1) [16-19]. Along the anterior-posterior (A-P) axis, maternally provided Bicoid (BCD) and Caudal (CAD) first establish the expression patterns of gap and terminal class factors, such as Giant (GT) and Tailless (TLL). These A-P early regulators then collectively direct transcription of A-P pair-rule factors, such as Paired (PRD) and Hairy (HRY), which in turn cross-regulate each other and may redundantly repress gap gene expression [20]. A similar cascade of maternal and zygotic factors controls patterning along the dorsal-ventral (D-V) axis [19]. Approximately 1 hour after zygotic transcription has commenced, the expression of around 1,000 to 2,000 genes is directly or indirectly regulated in complex three-dimensional patterns by this collection of factors [12,21-23].

**Table 1:** The 21 sequence-specific transcription factors studied

Factor	Symbol	DNA binding domain	Regulatory class
Bicoid	BCD	Homeodomain	A-P early maternal
Caudal	CAD	Homeodomain	A-P early maternal
Giant	GT	bZip domain	A-P early gap
Hunchback	HB	C2H2 zinc finger	A-P early gap
Knirps	KNI	Receptor zinc finger	A-P early gap
Kruppel	KR	C2H2 zinc finger	A-P early gap
Huckebein	HKB	C2H2 zinc finger	A-P early terminal
Tailless	TLL	Receptor zinc finger	A-P early terminal
Dichaete	D	HMG/SOX class	A-P early gap-like
Ftz	FTZ	Homeodomain	A-P pair rule
Hairy	HRY	bHLH	A-P pair rule
Paired	PRD	Homeodomain/paired domain	A-P pair rule
Runt	RUN	Runt domain	A-P pair rule
Sloppy paired 1	SLP1	Forkhead domain	A-P pair rule
Daughterless	DA	bHLH	D-V maternal
Dorsal	DL	NFkB/rel	D-V maternal
Mad	MAD	SMAD-MH1	D-V zygotic
Medea	MED	SMAD-MH1	D-V zygotic
Schnurri	SHN	C2H2 zinc finger	D-V zygotic
Snail	SNA	C2H2 zinc finger	D-V zygotic
Twist	TWI	bHLH	D-V zygotic

A-P, anterior-posterior; D-V, dorsal-ventral.

Tens of functional CRMs have been mapped within the network (for example, [8,19,24-26]), which each drive distinct subsets of target gene expression and which have generally been assumed to be each directly controlled by only a limited subset of the blastoderm factors. For example, the four stripe CRMs in the *even-skipped *(*eve*) gene are each controlled by various combinations of A-P early regulators, such as BCD and Hunchback (HB), and a separate later activated autoregulatory CRM is controlled by A-P pair rule regulators, including EVE and PRD [24,27-29].

The different transcriptional regulatory activities of these factors leads them to convey quite distinct developmental fates and morphological behaviors on the cells in which they are expressed. For example, the D-V factors Snail (SNA) and Twist (TWI) specify mesoderm, the pair rule factors EVE and Fushi-Tarazu (FTZ) specify location along the trunk of the A-P axis, and TLL and Huckebein (HKB) specify terminal cell fates.

The blastoderm regulators include members of most major animal transcription factor families (for example, Table 1) and act by mechanisms common to all metazoans [1]. Thus, the principles of transcription factor targeting and activity elucidated by our studies should be generally applicable.

We previously used immunoprecipitation of *in vivo *crosslinked chromatin followed by microarray analysis (ChIP/chip) to measure binding of the six gap and maternal regulators involved in A-P patterning in developing embryos (Table 1) [11]. These proteins were found to bind to overlapping sets of several thousand genomic regions near a majority of all genes. The levels of factor occupancy vary significantly though, with the few hundred most highly bound regions being known or probable CRMs near developmental control genes or near genes whose expression is strongly patterned in the early embryo. The thousands of poorly bound regions, in contrast, are commonly in and around house keeping genes and/or genes not transcribed in the blastoderm and are either in protein coding regions or in non-coding regions that are evolutionarily less well conserved than highly bound regions. For five factors, their recognition sequences are no more conserved than the immediate flanking DNA, even in known or likely functional targets, making it difficult to identify functional targets from comparative sequence data alone.

Here we extend our analysis to an additional 15 blastoderm regulators belonging to four new regulatory classes: A-P terminal, A-P gap-like, A-P pair rule and D-V (Table 1). We find that these proteins, like the A-P maternal and gap factors, bind to thousands of genomic regions and show similar relationships between binding strength and apparent function. Remarkably, these structurally and functionally distinct factors bind to a highly overlapping set of genomic regions. Our analyses of this uniquely comprehensive dataset suggest that distinct developmental fates are specified not by which genes are bound by a set of factors, but rather by quantitative differences in factor occupancy on a common set of bound regions.

## Results and Discussion

We performed ChIP/chip experiments to map the genome-wide binding of 15 transcription factors and analyzed these data along with the six factors whose binding we have previously described. In addition to these 21 factors, we also determined the *in vivo *binding of the general transcription factor TFIIB, which, together with previous data on the transcriptionally elongating, phosphorylated form of RNA polymerase [11], provide markers for transcriptionally active genes and proximal promoter regions.

### ChIP/chip is a quantitative measure of relative DNA occupancy *in vivo*

We applied stringent statistical criteria to identify the regions bound by each factor with either a 1% or 25% expected false discovery rate (FDR) [11]. While there was considerable variation in the number of bound regions identified for each factor, there were typically around 1,000 bound regions at a 1% FDR and 5,000 at a 25% FDR (Table 2). We ranked bound regions for each factor based on the maximum array hybridization intensity within the 500-bp "peak" window of maximal binding within each region.

**Table 2:** The numbers of genomic regions bound in blastoderm embryos

			Number of bound regions		Overlap between antibodies for the same factor	
				
Regulatory class	Factor antibody	Amino acids recognized	1% FDR	25% FDR	% overlap	r
A-P early	BCD 1*	56-330	619	3,295	95	0.79
maternal	BCD 2*	330-489	702	3,404	93	0.81
	CAD 1*	1-240	1,591	6,326	NA	NA
						
A-P early gap	GT 2*	182-353	1,070	3,968	NA	NA
	HB 1*	1-305	1,832	4,707	86	0.64
	HB 2*	306-758	1,718	6,675	92	0.80
	KNI 1*	130-280	36	330	97	0.90
	KNI 2*	281-425	197	5,167	83	0.86
	KR 1*	1-230	3,593	11,323	96	0.91
	KR 2*	350-502	4,084	12,255	93	0.93
						
A-P early terminal	HKB 1	1-100	1,012	5,339	99, 94	0.88, 0.64
	HKB 2	101-200	614	4,241	99, 89	0.81, 0.34
	HKB 3	201-297	638	3,766	99, 99	0.92, 0.99
	TLL 1	110-259	429	2,650	NA	NA
						
A-P early gap-like	D 1	1-103	6,452	16,501	NA	NA
						
A-P pair rule	FTZ 3	All	403	3,721	NA	NA
	HRY 1	123-221	1,704	6,053	97	0.80
	HRY 2	254-337	2,729	10,979	80	0.73
	PRD 1	355-450	2,061	7,145	96	0.93
	PRD 2	450-613	1,273	5691	99	0.92
	RUN 1	24-127, 240-318	921	8,809	77	0.79
	RUN 2	319-510	172	2,903	99	0.75
	SLP1 1	1-119	1,171	6,974	NA	NA
						
D-V maternal	DA 2	511-693	5,534	14,144	NA	NA
	DL 3	All	9,358	18,113	NA	NA
						
D-V zygotic	MAD 2	144-254	204	10,969	NA	NA
	MED 2	385-523, 630-713	5,458	9,273	NA	NA
	SHN 2	1617-1750	341	1,400	47	0.70
	SHN 3	2115-2279	121	363	87	0.38
	SNA 1	75-166	596	4,868	100	0.87
	SNA 2	167-258	2,800	15,811	61	0.82
	TWI 1	1-178	6,686	17,486	99	0.98
	TWI 2	259-363	7,416	19,605	98	0.98
						
General	Pol II H14*	CTD	3,108	7,991	NA	NA
	TFIIB	All	1,943	6,002	NA	NA

The number of bound regions at 1% and 25% false discovery rate (FDR) thresholds were determined by the symmetric null test [11]. The percentage overlap is defined as the percentage of 1% FDR 500-bp peak windows for one antibody that completely overlap a 25% FDR bound region for the other antibody/antibodies for the same factor. The Pearson correlation coefficient (r) is the correlation between the peak score from 1% FDR bound regions for one antibody and the corresponding 500 bp window score for the second antibody. Asterisks indicate previously published data [11]. NA, not applicable.

We carried out an extensive series of controls and analyses to validate the antibodies and array data, and to ensure that our array intensities could be interpreted as a quantitative measure of relative transcription factor occupancy on each genomic region, that is, as a measure of the average numbers of molecules of a particular factor occupying each region (see [11] for further details).

**Figure 1:**
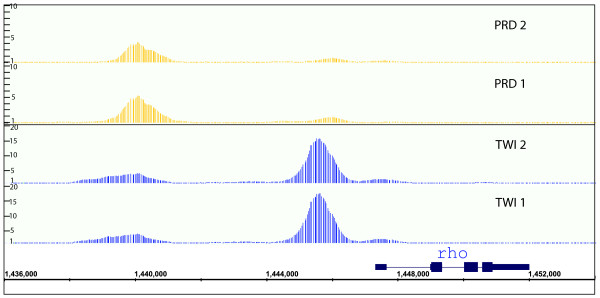
Similar patterns of *in vivo *DNA binding are detected by antibodies recognizing distinct epitopes on the same factor. The 675-bp window scores for ChIP/chip experiments across the *rhomboid *(*rho*) gene locus. Data are shown for pairs of antibodies against non-contigous portions of PRD and TWI proteins (Table 2). Nucleotide coordinates in the genome are given in base-pairs.

For all but three factors, antisera were affinity-purified against recombinant versions of the target protein from which all regions of significant homology to other *Drosophila *proteins were removed. Where practical, antisera were independently purified against non-overlapping portions of the factor. When this was done, the ChIP/chip data from these different antisera gave strikingly similar array intensity patterns (for example, Figure 1), strong overlap between the bound regions identified (mean overlap = 91%; Table 2; Additional data file 1), and high correlation between peak window intensity scores (mean r = 0.79; Table 2), all of which strongly indicates that the antibodies significantly immunoprecipitate only the specific factor and that our ChIP/chip assay is very quantitatively reproducible. The specificity of the antibodies used is further confirmed by immunostaining experiments that show that they recognize proteins with the proper spatial and temporal pattern of expression (Additional data file 1).

We used two different methods to estimate FDRs, one based on precipitation with non-specific IgG, and the other based on statistical properties of data from the specific antibody alone. These estimates broadly agree (Additional data file 2). Our previously published quantitative PCR analysis of immunoprecipitated chromatin for regions randomly selected from the rank list of bound regions and also control BAC DNA 'spike in' experiments support the FDR estimates, suggest that the false negative rate is very low for all but the most poorly bound regions, and indicate that the array intensity signals correlate with the relative amounts of genomic DNA brought down in the immunoprecipitation [11].

**Figure 2:**
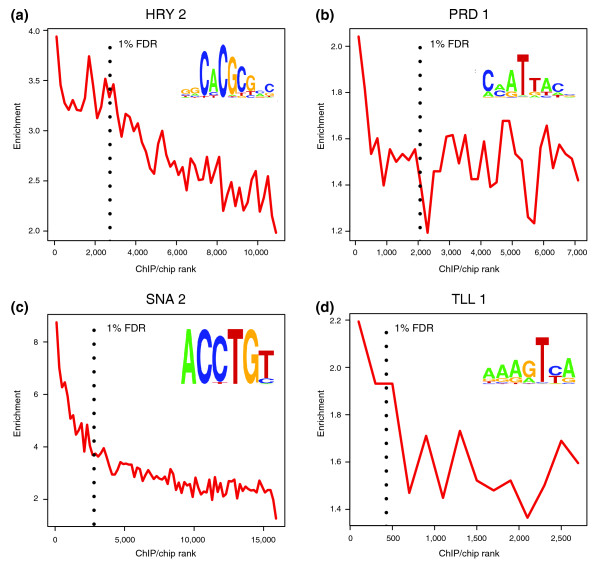
Recognition sequence enrichment correlates with ChIP/chip rank. Fold enrichment of matches to a position weight matrix (PWM) in the 500-bp windows around ChIP/chip peaks (± 250 bp), in non-overlapping cohorts of 200-peaks down the ChIP-chip rank list to the 25% FDR cutoff. Matches to the PWM below a *P*-value of ≤ 0.001 were scored. The PWMs used are shown as sequence logo representations [67]. The most highly bound peaks are to the left along the x-axis and the location of the 1% FDR threhold is indicated by a black, vertical dotted line. Shown are plots for the **(a) **HRY 2, **(b) **PRD 1, **(c) **SNA 2 and **(d) **TLL 1 antibodies.

The enrichment of factor recognition DNA sequences in ChIP/chip peaks shows a modest positive correlation with peak array intensity score. Importantly, this is seen even in the upper portion of the rank list where the percentages of false positives are too few to significantly influence the analysis (Figure 2; Additional data files 3 and 4) [11]. While the presence of predicted binding sites is neither a necessary nor sufficient determinant of binding, this correlation strongly suggests that the number of factor molecules bound to a DNA region *in vivo *significantly affects the amount of each DNA region crosslinked and immunoprecipitated in the assay.

Finally, the relative array intensity scores from our formaldehyde crosslinking ChIP/chip experiments broadly agree with the relative density of factor binding detected by earlier Southern blot-based *in vivo *UV crosslinking [30,31] (Additional data file 5). For BCD, FTZ and PRD the Pearson correlation coefficients are 0.79, 0.67, and 0.48, respectively, comparing the data from these two assays on the same genomic regions. This agreement is important because it argues that the measured relative signals in both assays are not powerfully influenced by differences in crosslinking efficiency to various DNAs, indirect crosslinking of proteins to DNA via intermediary proteins (which should not be detected by UV crosslinking), or differences in epitope accessibility during immunoprecipitation (which again should be much lower for UV crosslinking). Instead, the correspondence indicates that both these methods provide a reasonable estimate of the relative number of factor molecules in direct contact with different genomic regions *in vivo*.

### Binding to thousands of genomic regions over a relatively narrow range of occupancies

**Figure 3:**
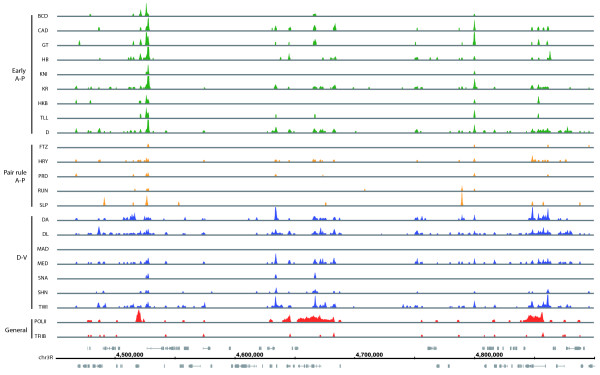
Broad, overlapping patterns of binding of transcription factors to the genome in blastoderm embryos. Data are shown for eight early A-P factors (green), six pair rule A-P factors (yellow), seven D-V factors (blue), and two general transcription factors (red). The 675-bp ChIP/chip window scores are plotted for regions bound above the 1% FDR threshold in a 500-kb portion of the genome. The locations of major RNA transcripts are shown below in grey for both DNA strands. The genome coordinates are given in base-pairs. For those factors for which ChIP/chip data are available for more than one antibody, data are shown for the antibody that gave the most bound regions above the 1% FDR threshold using the symmetric null test.

Like the 6 previously examined A-P factors, the 15 newly studied regulators are detectably bound to thousands of genomic regions widely spread throughout the genome (Figure 3; Table 2; Additional data files 2, 6 and 7). The median number of 1% FDR bound regions detected by the antibody giving the most efficient immunoprecipitation for each of the 21 factors is 1,591 and the median number detected at the 25% FDR level is 7,145. At a 1% FDR, 23 Mb of the euchromatic genome is covered by a bound region for at least one factor, and of this, 9.8 Mb is within 250 bp of a ChIP/chip peak. At a 25% FDR, 32.2 Mb of the genome is within 250 bp of a ChIP/chip peak, which is 27% of the 118.4 Mb euchromatic genome. This binding is so extensive that, for each factor, on average, the transcription start sites of 20% of *Drosophila *genes lie within 5,000 bp of its 1% FDR ChIP/chip peaks, and for its 25% FDR peaks the equivalent figure is 54% of genes (Table 3).

**Table 3:** Percentage of genes whose transcription start site is within 5 kb of ChIP/chip peaks

Regulatory class	Factor antibody	% genes close to 1% FDR peaks	% genes close to 25% FDR peaks
A-P early	BCD 2	6.2	29.6
	CAD 1	12.6	48.9
	GT 2	7.7	27.2
	HB 1	14.7	34.5
	KNI 2	1.2	37.2
	KR 2	27.0	65.3
	HKB 1	9.0	41.3
	TLL 1	2.8	20.8
	D 1	52.6	84.1
			
A-P pair rule	FTZ 3	2.6	29.1
	HRY 2	20.4	64.3
	PRD 1	14.8	51.0
	RUN 1	6.0	60.4
	SLP1 1	11.4	52.4
			
D-V	DA 2	38.2	76.7
	DL 3	66.5	87.0
	MAD 2	1.6	73.5
	MED 2	50.6	74.8
	SHN 2	2.1	9.6
	SNA 2	23.6	83.0
	TWI 2	53.0	90.3

For those factors for which ChIP/chip data are available for more than one antibody, values shown are for the antibody that gave the most bound regions above the 1% FDR threshold using the symmetric null test.

**Figure 4:**
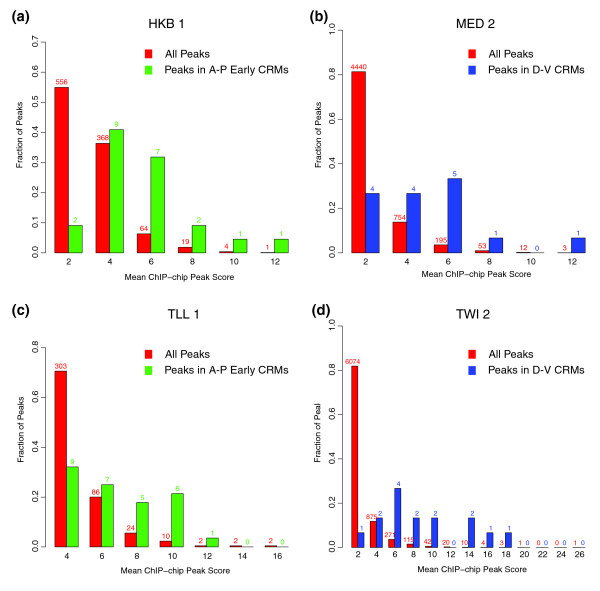
Known CRMs tend to be among the regions more highly bound *in vivo*. The 1% FDR bound regions for **(a) **HKB 1, **(b) **MED 2, **(c) **TLL 1 and **(d) **TWI were each divided into cohorts based on peak window score (x-axis). The fraction of all bound regions in each cohort (red bars) are shown (y-axis). In (a, c), the fraction of bound regions in each cohort in which the peak 500-bp window overlaps a CRM known to be regulated by at least some A-P early factors is shown (green bars). In (b, d), the fraction of bound regions that overlap a CRM known to be regulated by at least some D-V factors are shown (blue bars). The number of bound regions in each cohort is given above the bars.

For each factor, the numbers of regions bound at progressively lower array intensity signals increases near exponentially. At an array intensity of only 3- to 4-fold less than that of the most highly bound 20 to 30 regions, typically several thousand regions are bound by a protein (Figure 4; Additional data file 8). Because DNA amplification and array hybridization and imaging methods compress the measured differences in the amounts of DNA in an immunoprecipitation, the actual differences in transcription factor occupancy will be approximately three times greater than the differences in ChIP/chip peak intensity scores [11]. Nevertheless, many genes are bound over a surprisingly narrow range of transcription factor occupancies.

### A quantitative continuum of binding and function

Our earlier analyses of the six maternal and gap A-P factors showed that although these proteins bind to large number of regions, the most highly bound regions clearly differ in many regards from the more poorly bound, many of which may not be functional targets. Parallel analyses of the other 15 factors demonstrate the same trends.

First, for those factors for which a significant number of target CRMs are known, the few hundred most highly bound regions are enriched for these targets. Transgenic promoter, genetic, *in vitro *DNA binding and other data have identified a set of 44 CRMs as direct targets of subsets of the A-P early factors and 16 CRMs as direct targets of particular combinations of D-V regulators [8,25,32]. Figure 4 and Additional data file 8 show that the 500-bp ChIP/chip peaks that overlap CRMs known to be targets of at least some members of a given regulatory class are bound by all members of that class, on average, at higher levels than the majority of genomic regions at which these proteins are detected.

**Figure 5:**
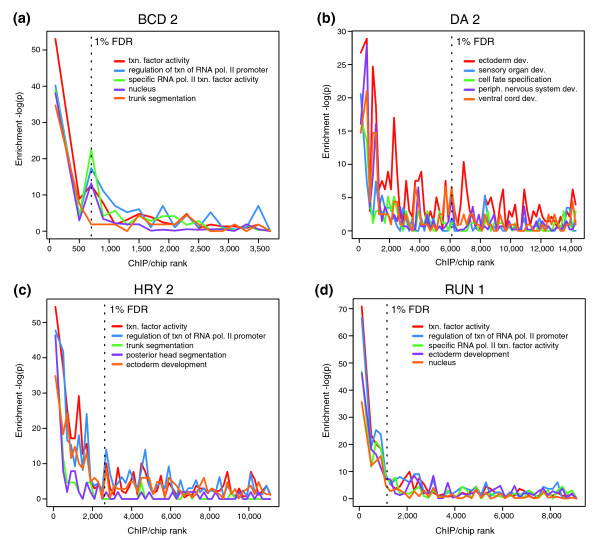
Genes that control development are enriched in highly bound regions. The five most enriched Gene Ontology terms [68] in the 1% FDR bound regions for each factor were identified (enrichment measured by a hyper geometric test). The significance of the enrichment (-log(*P*-value)) of these five terms in non-overlapping cohorts of 200 peaks are shown down to the rank list as far as the 25% FDR cutoff. The most highly bound regions are to the left along the x-axis and the location of 1% FDR threshold is indicated by a black, vertical dotted line. Shown are the results for the **(a) **BCD 2, **(b) **DA 2, **(c) **HRY 2, and **(d) **RUN 1 antibodies. Dev., development; periph., peripheral; RNA pol, RNA polymerase; txn, transcription.

Second, the most highly bound regions, on average, are closer to genes with developmental control functions, whereas poorly bound regions are frequently closer to metabolic enzymes and other 'house keeping' genes (Figure 5; Additional data files 4 and 9). For most of the 21 factors, this enrichment reduces significantly between the top of the rank list and the 1% FDR threshold, which, if our FDR estimates are good, rules out the possibility that the presence of false positives has influenced this result.

**Figure 6:**
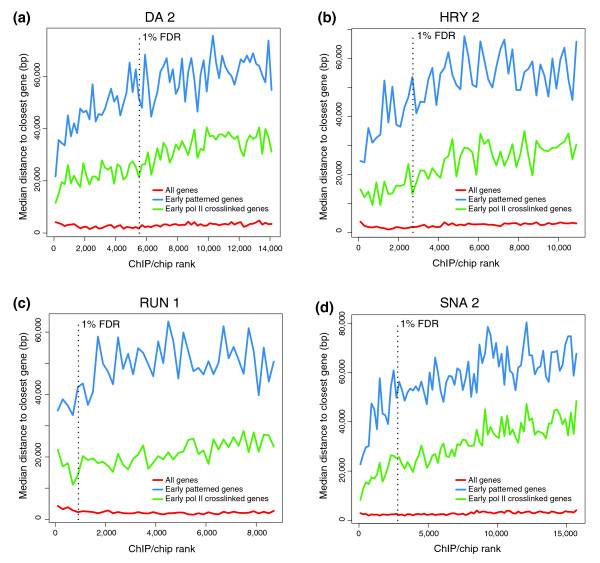
Highly bound regions are preferentially associated with genes transcribed and patterned in the blastoderm. Shown are the median distance of non-overlapping 200-peak cohorts to the closest gene belonging to each of three categories of gene: all genes (from genome release 4.3, March 2006; red lines); genes with known patterned expression (hand annotated based on Berkeley *Drosophila *Genome Project *in situ *images [23]; blue lines); and transcribed genes (defined by our RNA polymerase II (pol II) ChIP/chip binding [11]; green lines). Data are plotted down the ChIP/chip rank list to the 25% FDR threshold. The most highly bound regions are to the left along the x-axis and the location of 1% FDR threshold is indicated by a black, vertical dotted line. Shown are the results for the **(a) **DA 2, **(b) **HRY 2, **(c) **RUN 1, and **(d) **SNA 2 antibodies.

Third, for the majority of factors the more highly bound regions tend to be closest to genes that are transcribed at the blastoderm stage and whose spatial expression is patterned at this stage (Figure 6; Additional data files 4 and 10). Poorly bound regions, in contrast, are closest to genes that are transcriptionally inactive or not patterned at this stage. For a minority of factors this trend is not as pronounced. However, this is probably because the regions bound highly by these proteins are already further away from the transcription start site of their known or likely target genes than are those of other factors (for example, Runt (RUN) 1 in Figure 6; and Sloppy paired (SLP)1 in Additional data file 10).

**Figure 7:**
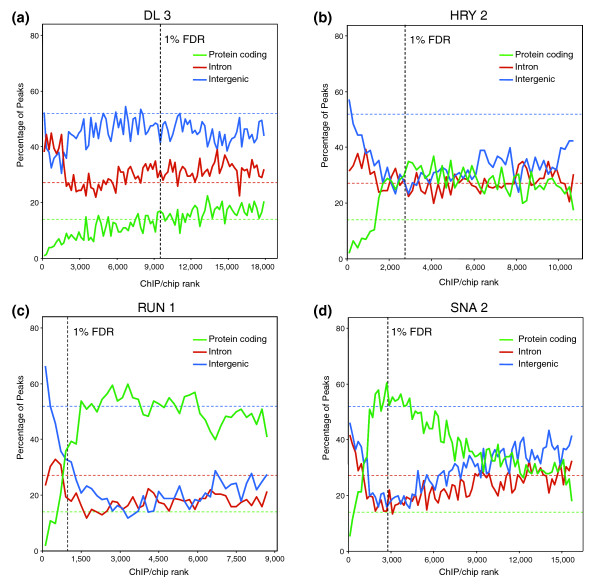
For some factors, poorly bound regions are preferentially found in protein coding sequences. The percentage of ChIP/chip peaks are plotted in non-overlapping cohorts of 200 peaks that are in protein coding (red), intronic (blue), and intergenic (green) sequences. Results are shown for cohorts down the rank lists to the 25% FDR cutoff. The percentages for each class of genomic feature are indicated as horizontal dotted lines in corresponding colors to the solid data lines. The most highly bound regions are to the left along the x-axis and the location of 1% FDR threshold is indicated by a black, vertical dotted line. Shown are the results for the **(a) **DL 3, **(b) **HRY 2, **(c) **RUN 1, and **(d) **SNA 2 antibodies.

Fourth, poorly bound regions for a subset of factors show a surprising preference to be located in protein coding regions. This is particularly striking for FTZ, Knirps (KNI), Mad (MAD), RUN and SNA, but a number of other factors show a less dramatic but similar trend (see regions between the 1% and 25% FDR thresholds in Figure 7 and Additional data file 11).

**Figure 8:**
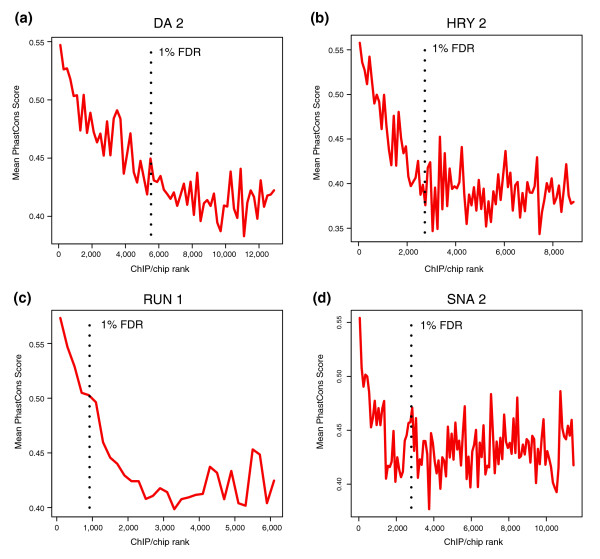
Highly bound regions are preferentially conserved. Mean PhastCons scores in the 500-bp windows (± 250 bp) around peaks, in non-overlapping cohorts of 200 peaks down the rank list towards the 25% FDR cutoff. The most highly bound peaks are to the left along the x-axis and the location of 1% FDR threshold is indicated by a black, vertical dotted line. Shown are the results for the **(a) **DA 2, **(b) **HRY 2, **(c) **RUN 1, and **(d) **SNA 2 antibodies.

Fifth, for those bound regions in intergenic and intronic sequences (that is, in non-protein coding sequences) the more highly bound are significantly more conserved than those poorly bound (Figure 8; Additional data files 4 and 12). For most factors, however, their specific recognition sequences are not particularly more conserved than the remaining portion of the 500-bp peak windows ([11] and our unpublished data). Thus, for most factors, it cannot be concluded from this analysis alone that recognition sequences are being conserved because they are functional targets. But it can be concluded that the more highly bound regions likely are, on average, more evolutionarily constrained function than poorly bound regions.

Taking all of these five analyses into account, the few hundred most highly bound regions have characteristics of likely functional targets of the early embryo network. Although some poorly bound regions are also likely to be functional targets at this time, including ones weakly modulating transcription of housekeeping genes (for example, [22]), many do not appear to be classical CRMs that drive transcription in the blastoderm. A minority do become more highly bound in the later embryo and may be active then (our unpublished data), but the binding to many others we feel is likely to be non-functional, including that to most of those in protein coding regions.

Our analysis contrasts with the predominant qualitative interpretation of *in vivo *crosslinking data by other groups studying animal regulators [32-46]. Many of these groups have also shown that factors bind to a large number of genomic regions. They have not, however, noted the many differences between highly bound and poorly bound regions shown in Figures 4 to 8. In addition, with only a few exceptions [43,44,46], they have not seriously considered the possibility that some portion of the binding detected is non-functional. We suspect that similar correlations between levels of factor occupancy and likely function of bound regions will be found for other factors once quantitative differences amongst bound regions are considered.

### Factors bind to highly overlapping regions

**Figure 9:**
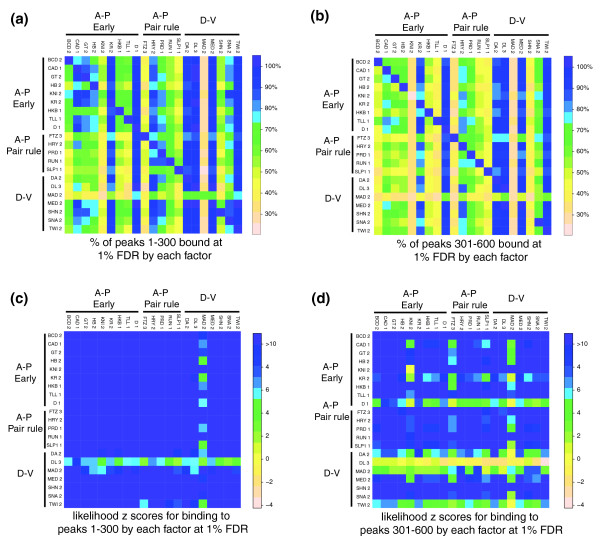
Heat maps showing high overlap in binding among the blastoderm factors. **(a, b) **Each row shows the percentage of a cohort of 300 single nucleotide position peaks for a factor that are overlapped by 1% FDR regions bound by each of the other factors in turn. **(c, d) **Each row shows the Genome Structure Correction z scores for the likelihood that the overlap plotted in (a, b) occurs by chance given the proportion of the genome bound by each factor. (a, c) Results for the most highly bound 300 peaks (1-300). (b, d) Results for the second most highly bound cohort of 300 peaks (301-600). Note that the 1% FDR threshold does not lie within ranks 1 to 600 for 17 of the 21 factors shown, and, thus, for these proteins the bulk of the differences observed between the 1-300 and the 301-600 cohorts are not attributable to false positives.

Another striking feature of our *in vivo *DNA binding data is that there is considerable overlap in the genomic regions bound by the 21 factors (Figures 3), even though they belong to 11 DNA binding domain families and multiple regulatory classes, often act via distinct CRMs, and clearly specify distinct developmental fates. To quantify this overlap, we scored for each protein the percent of peaks that are overlapped by a 1% FDR region for each factor in turn (Figure 9a, b; Additional data file 13). This analysis shows, for example, that of the 300 peaks most highly bound by the A-P early regulator BCD, between 6% and 100% are co-bound by the other 20 factors, some of the highest overlap (>94%) being with the D-V regulators Medea (MED), Dorsal (DL) and TWI (Figure 9a, top row). Peaks bound more poorly are overlapped to a lesser degree, but there is still considerable cross-binding to these regions (Figure 9b; unpublished data).

To calculate the probability that this extensive co-binding occurs by chance, we used the Genome Structure Correction (GSC) statistic [43], which is a conservative measure that takes into account the complex and often tightly clustered organization of bound regions across the genome. For the great majority of the pair-wise co-binding shown in Figures 9a, b, these probabilities have Bonferroni corrected *P*-values < 0.05 (all instances with z scores ≥4 in Figure 9c, d) and, thus, the overlap is highly unlikely to have occurred by chance. With such extensive co-binding, it is not surprising that some regions are bound by many factors. Averaged over all regulators, 88% of their top 300 peak windows are bound by 8 or more factors and 40% are bound by 15 or more factors (Additional data file 13).

Several recent *in vivo *crosslinking studies have also noted significant overlap in binding between some sequence-specific factors in animals [32,34,37,44,46]. In these other cases, however, the overlapping factors are known to have related functions and, thus, the co-binding is less surprising. Work using the DamID method showed a high overlap in binding when transcription factors with different functions and specificities were ectopically expressed in tissue culture cells [47], and it was suggested that these binding 'hotspots' were non-functional storage sites. In contrast to these other studies, we have found overlapping binding for a larger number of regulators, many of which are well characterized as having distinct biological and transcriptional regulatory specificities. The binding we have measured is for endogenous factors, and the greatest overlap in binding is at known and probable functional targets. Thus, it does not seem that overlapping patterns of binding reflect either shared functions or a lack of function. Instead, we must ask how the undoubtedly distinct specificities of the blastoderm factors arise despite the overlap.

### Quantitative differences in binding correlate with biological and transcriptional regulatory specificity

**Figure 10:**
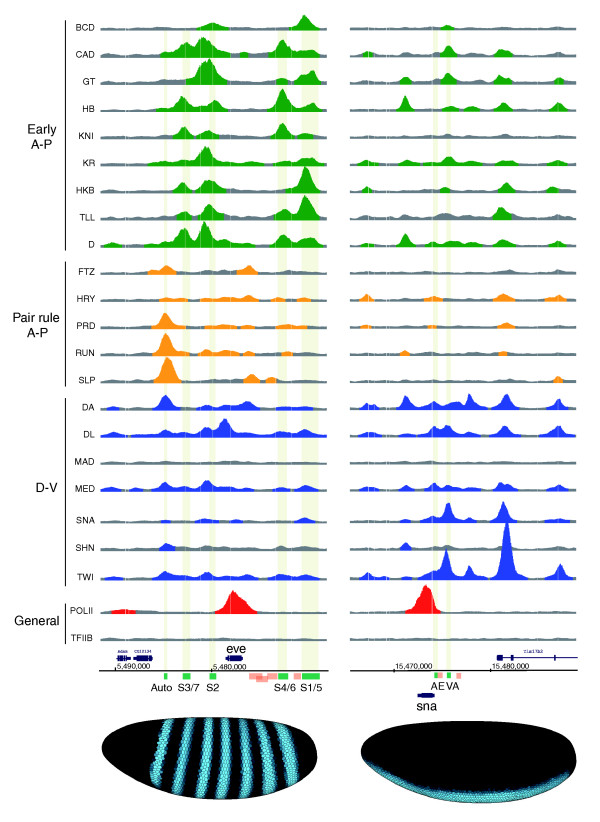
*In vivo *DNA binding of 21 sequence-specific and 2 general transcription factors to the *even skipped *(*eve*) and *snail *(*sna*) loci. ChIP/chip scores are plotted for 675-bp windows associated with all oligonucleotides on the array in the portions of the genome shown. In those regions bound above the 1% FDR threshold, the plots are colored green (Early A-P factors), yellow (Pair rule A-P factors), blue (D-V factors) or red (General factors). The locations of major RNA transcripts are shown below (blue) for both DNA strands together with the locations of CRMs active in blastoderm embryos (green) and later stages of development (salmon). Nucleotide coordinates in the genome are given in base-pairs. At the bottom is show the mRNA expression patterns of *eve *and *sna *in mid-stage 5 blastoderm embryos from the BDTNP's VirtualEmbryo using PointCloudXplore [12,69]. A more detailed plot comparing ChIP scores for both factor and negative control immunoprecipitations is shown in Additional data file 14, including data for all antibodies shown in Table 2.

To address this question, we first looked in detail at the pattern of binding on the CRMs of two well-studied target genes. The *eve *gene is expressed in a seven stripe pair-rule pattern along the A-P axis and contains four stripe CRMs that are known targets of the A-P early factors (Figure 10, S3/7, S2, S4/6 and S1/5) and a later activated autoregulatory CRM thought to be a target of the A-P pair rule factors EVE and PRD (Figure 10, Auto) [24,28,29]. The *sna *gene is expressed in a ventral stripe of expression and has two known CRMs that are targets of the D-V regulators TWI or DL (Figure 10, AE and VA) [48]. Consistent with the analysis in Figure 9, there is a high co-binding of members of all three major regulatory classes to each of these CRMs at a 1% FDR (Figure 10; Additional data file 14), and even more extensive co-binding is seen when lower level interactions detected at a 25% FDR and in *in vivo *UV crosslinking experiments are taken into account [31] and our unpublished data). However, the factors show quantitative preferences in binding to the CRMs that broadly correlates with their expected function: A-P early factors most strongly occupy the four *eve *stripe CRMs, A-P pair rule factors most strongly occupy the *eve *autoregulatory element, and the D-V factors TWI and SNA most strongly occupy the two *sna *CRMs (Figure 10). Thus, differences in the levels of occupancy on common genomic regions could be significant determinants of regulatory specificity.

**Figure 11:**
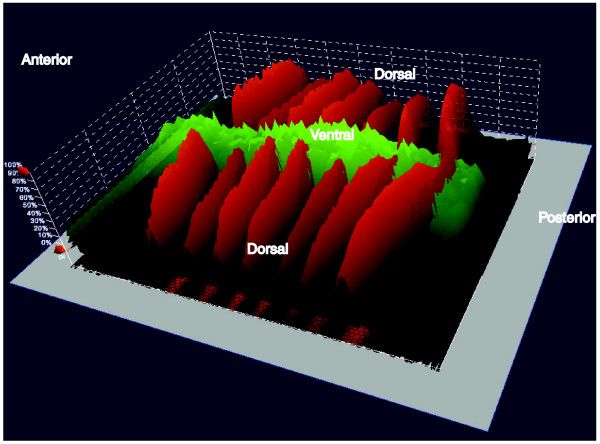
The relative levels of *eve *and *sna *mRNA expression in mid-stage 5 blastoderm embryos at cellular resolution. Shown is a display from PointCloudXplore of a two-dimensional cylindrical projection of a VirtualEmbryo (D_mel_wt__atlas_r2.vpc) [12,69,70], where the level of mRNA expression is shown by height above a two-dimensional projection of the embryo surface. *eve *mRNA expression is shown in red and *sna *in green. The *eve *data are the average from images of 368 embryos and the *sna *data from 12 embryos.

The fact that the higher levels of binding better reflect expectations based on earlier molecular genetic experiments, however, does not necessarily indicate that only these interactions are functional. For example, recent studies using image analysis of three-dimensional cellular resolution data have shown that there are modest quantitative affects of D-V regulators on *eve *expression and of A-P regulators on *sna *expression (Figure 11) [9-11], which could be due to the low-level occupancy of D-V regulators on *eve *and of A-P regulators on *sna*. Indeed, these quantitative methods show that the expression patterns of most genes in the blastoderm are much more complex than early low-resolution expression data implied [9,10,12]. Thus, the regulation of blastoderm genes may involve input from a broader range of factors than first assumed, with the degree of transcriptional regulation correlating with the degree of factor binding.

Other work, however, cautions against assuming that all of the lower level interactions shown in Figure 10 result in transcriptional regulation. In the case of the binding of A-P gap factors to the *eve *autoregulatory element, transgenic promoter analysis indicates that this binding is not sufficient to detectably activate this CRM in early stage 5 embryos [24]. A similar argument can be made for binding of A-P pair rule factors to the *eve *stripe CRMs [24,27,28,49]. In these cases, either this lower level binding is non-functional or it plays an augmentary role only in the context of multiple promoter elements. It is not sufficient for regulation on its own.

**Figure 12:**
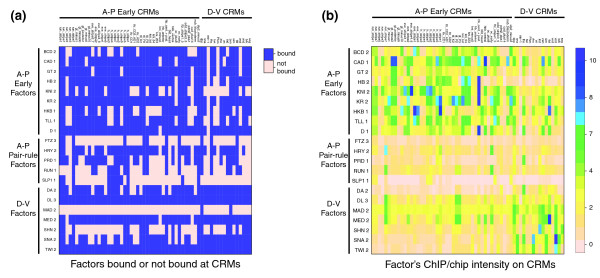
Heat maps showing the binding of blastoderm transcription factors to validated A-P early and D-V CRMs. **(a) **Each row shows if a factor is detected binding or not to each CRM, where binding is defined as a 1% FDR region that overlaps the CRM by 500 bp or more. **(b) **Each row shows the ChIP/chip intensity of the highest 675-bp window for a factor on each of the 44 A-P early CRMs and 16 D-V CRMs. The intensities of all factors were placed on a similar scale by normalizing the data such that the intensity score of the most highly bound region in the genome for each factor is set to 10.

To more fully explore if there is a correlation between the level of factor occupancy on common sequences and functional specificity, we next compared the binding of all 21 factors on the 44 A-P early CRMs and 16 D-V CRMs described earlier. (There are too few known A-P pair rule CRMs to analyze in this way.) While most of these CRMs are each bound above the 1% FDR threshold by members of all three of the major regulatory classes (Figure 12a), the normalized levels of factor occupancy can be seen to broadly meet expectations (Figure 12b): the A-P early factors bind more highly to A-P early CRMs, the D-V factors mostly bind more highly to D-V CRMs, and the pair rule factors bind at lower levels to all of these CRMs than they do to other regions of the genome. There are a few instances where relatively high levels of binding are found to CRMs initially identified as targets of another regulatory class, but these likely reflect the fact that some of these CRMs show strong patterning along both the A-P and D-V axes (for example, [32]). Averaged over all interactions for members of each regulatory class, the levels of binding of each class match the general expectations for their specificity (Bonferroni corrected Mann Whitney test *P*-values all < 1 × 10^-8^; Additional data file 13).

**Figure 13:**
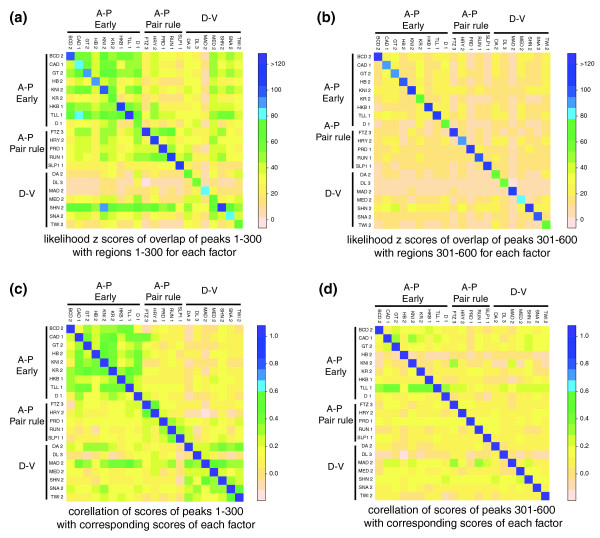
Heat maps showing two measures for factor binding specificity in blastoderm embryos. **(a, b) **Each row shows the GSC z score of the likelihood that the overlap between a cohort of ChIP/chip peaks for one factor and regions bound each factor in turn occurs by chance. **(c, d) **Each row shows the Pearson correlation coefficients between the intensity scores of a cohort of 300 peaks for a factor and the intensity scores of the equivalent 500-bp windows at the same genomic locations for each of the other factors in turn. (a, c) Results for the most highly bound 300 peaks (1-300). (b, d) Results for the second most highly bound cohort of 300 peaks (301-600). Note that the 1% FDR threshold does not lie within ranks 1 to 600 for 17 of the 21 factors (Table 2), and, thus, for these proteins the differences observed between the 1-300 and the 301-600 cohorts are not attributable to false positives.

Informative as the above analyses are, however, they are restricted to previously identified CRMs. These CRMs were identified experimentally using criteria that could well have excluded some types of functional targets. To explore if levels of occupancy correlate with functional specificity more widely, therefore, we examined binding to genomic regions without regard to any published information on which sequences different factors might act on. In the absence of any prior knowledge, we exploited our observation that, on known CRMs, members of a regulatory class have more similar specificities than members of different classes and used this to provide an expectation of specificity elsewhere in the genome. We used two measures to compare binding for factors within and between classes (Figure 13).

First, we used the previously described GSC statistic for the likelihood that two factors bind the same regions more frequently than expected by chance, but this time focusing only on the overlap between highly bound regions. All 441 pair-wise comparisons of overlap were computed between the 300 regions bound most highly by each factor (Figure 13a) and separately between the 300 next most highly bound regions (Figure 13b). For both cohorts, not surprisingly given our earlier analysis, co binding between most pair-wise combinations of factors occurs far more frequently than expected by chance, even where the proteins belong to different regulatory classes (z scores ≥4 in Figure 13a, b). However, for the top 300 bound regions, there is an obvious further preferential overlap among A-P early regulators as well as a moderate preference among the A-P pair rule factors and the D-V factors (Bonferroni corrected Mann Whitney tests suggest that, taken collectively, the preferential co-binding among A-P early regulators is highly significant (*P *< 9 × 10^-15^), while that among the A-P pair rule factors and the D-V factors is moderately significant (*P *< 2 × 10^-3^) for both; Additional data file 13). The next most highly bound cohort shows reduced preferential co-binding within regulatory classes, with only that among A-P early regulators being significant (*P *= 7 × 10^-9^; Figure 13b; Additional data file 13).

**Figure 14:**
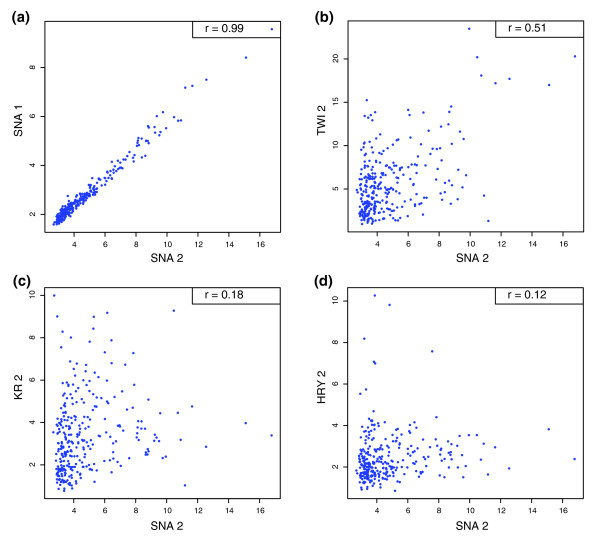
Scatter plots showing the correlation between 500-bp window scores. The 500-bp peak window scores for the top 300 regions detected by the SNA 2 antibody (x-axis) are compared against the score of the equivalent 500-bp windows detected in another Chip/chip experiment (y-axis) at the same genomic locations. The comparison is made against ChIP/chip data from experiments using the **(a) **SNA 1, **(b) **TWI 2, **(c) **Kruppel (KR) 2, and **(d) **HRY 2 antibodies. The Pearson correlation coefficients (*r*) for each comparison are shown in the top right of each panel.

Second, because the above measure only partially takes into account the different levels of occupancy on each bound region, we sought a measure that better captures this information. Scatter plots show that while ChIP/chip scores from experiments using antibodies to distinct portions of the same factor are highly correlated, pair-wise comparisons between factors reveal marked differences in scores, suggesting that correlation coefficients calculated in this way would be a useful measure of binding specificity (Figure 14). Therefore, we computed Pearson correlation coefficients for all pair-wise comparisons between factors for the most highly bound 300 regions and separately for the next most highly bound 300 regions (Figure 13c, d). Visual inspection shows that A-P early and D-V regulators generally show higher similarity in binding with members of their own regulatory class than they do with other factors. Similarly, the highest correlations for the A-P pair rule factors FTZ, HRY, RUN and SLP1 are with other pair rule proteins, though in this case preferences are shared with specific proteins rather than class-wide. Bonferroni corrected Mann Whitney tests indicate that correlation coefficients generally show more significant distinctions in binding preferences between the three regulatory classes than the z score measure, both taken collectively (A-P early *P *< 10^-15^, A-P pair rule *P *= 1 × 10^-6^, D-V *P *= 1 × 10^-9^), and on a per factor basis (Additional data file 13). They even detect moderate discrimination between the classes in the 301 to 600 cohort (A-P early *P *= 1 × 10^-3^, A-P pair rule *P *= 1 × 10^-3^, D-V *P *= 2 × 10^-2^).

Thus, across a broad array of mostly uncharacterized genomic regions the levels of binding of transcription factors correlate with the expectation that factors with more similar functions show more similar binding specificity. Consistent with our previous observation that highly bound regions appear more functionally significant, the distinctions in binding preferences between regulatory classes is larger on the most highly bound regions. Just as on the known CRMs, however, the distinctions between the different classes are relatively modest, suggesting that the regulatory specificity of transcription factors in general may be fuzzier than widely realized and perhaps also suggesting a role for post-DNA-binding events to increase the distinctions between factors.

All of the preceding analyses consider binding to short genomic regions. The target genes of blastoderm factors, however, are often found associated with several such regions (for example, Figure 10). Thus, while the above analyses establish that regulators show quantitative preferences for binding to individual genomic regions, they do not establish if they exhibit preferences for different genes.

**Figure 15:**
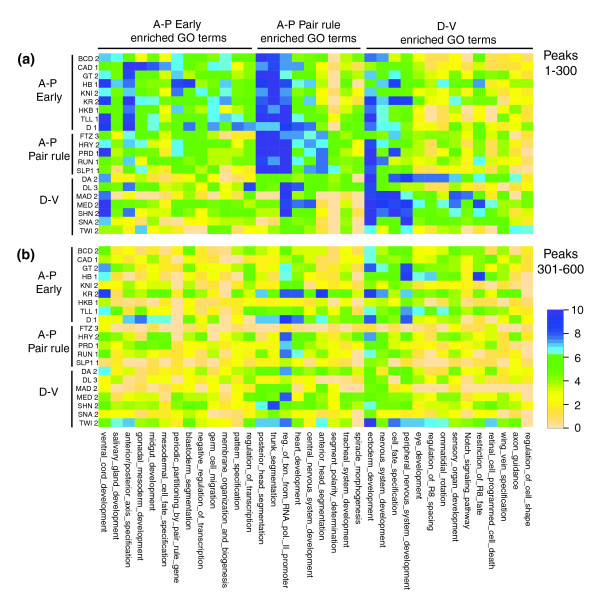
Heat map showing GO terms enriched in genes closest to regions bound by each factor. The seven most highly enriched GO terms associated with the closest genes to the 300 most highly bound peaks were determined for each of the 21 factors and the non-redundant set of all such terms identified. Each row shows the enrichment of each of these GO terms for one factor expressed as a normalized z score. The columns (GO terms) were arranged into three groups based on which of the three major regulatory classes of factor the GO terms are most enriched in, and are ranked from left to right based on the degree of this relative enrichment. **(a) **Results for the most highly bound 300 peaks (1-300); **(b) **results for the second most highly bound cohort of 300 peaks (301-600).

To determine whether these factors are targeting distinct sets of genes, for each bound region we identified the Gene Ontology (GO) term associated with the gene whose transcription start site is closest to the peak of binding. The enrichment of the GO terms associated with the 300 most highly bound peaks and the next most highly bound cohorts of peaks were then plotted as heat maps (Figure 15a, b). This shows that there are clear differences between factors as to which GO terms are associated with their top 300 peaks. In addition, there is a broad within regulatory class preference for which types of gene transcription factors bind to most strongly. More fine-grained similarities between subsets of factors within the major regulatory classes are also apparent. Thus, the quantitative preferences apparent at the level of individual bound regions must extend to some degree to the level of the clusters of regions associated with each gene and with different gene types.

The GO terms associated with the 301 to 600 ranked peaks show much less difference between each factor and regulatory class, consistent again with these less highly bound regions playing a lesser role in determining biological specificity and function.

Some of the types of gene preferentially bound by the regulatory classes readily fit expectations; for example, the strong association of genes involved in A-P axis specification, patterning by pair rule genes, and trunk and head segmentation with A-P early and A-P pair rule factors. Others are unexpected, such as the preference of D-V regulators for a series of GO terms related to eye development. Most likely the differences between factors revealed in these heat maps reflect differences due to target genes that are strongly patterned along the A-P axis versus those strongly patterned along the D-V axis. Because important effectors of blastoderm regulators' functions are patterned along both body axes, because the early factors both activate or repress target genes, and because GO terms imperfectly capture and categorize the biological function of each gene, this analysis does not provide a complete description of the different specificities of each factor at the target gene level.

### A general model for animal transcription factor binding and function

What mechanism, though, drives the extraordinarily extensive, overlapping pattern of binding? We speculate that the pattern is a natural consequence of these factors' intrinsic DNA binding specificities (as measured *in vitro*), the relatively high concentrations at which they are expressed in nuclei in which they are active, chromatin structure, and the law of mass action.

Most animal transcription factors recognize short degenerate DNA sequences that occur frequently throughout the length of most genes [1,5,6]. It has long been proposed on thermodynamic grounds that the majority of transcription factor molecules would be bound to DNA in the nucleus, rather than be free in solution [50-52]. In eukaryotes, only a subset of the genome is fully accessible to sequence-specific DNA binding factors because of the presence of nucleosomes [53-60]. Any several hundred base-pair segment of such accessible DNA will likely contain moderate to high affinity recognition sequences for a large proportion of transcription factors. Since many of the blastoderm factors are present at concentrations of many tens of thousand of molecules per cell [30,61], they may well be able to significantly occupy these sites, generating a highly overlapping pattern of binding focused at open chromatin regions.

In addition to the independent interactions of transcription factors with their target DNA sequences in open chromatin, some of the overlap in binding may likely arises from protein-protein interactions in which a factor associates with an accessible region as a result of direct interactions with protein molecules bound to the region. Such indirect binding - whether between transcription factors, or mediated by the large numbers of co-factors associated with CRMs - could explain the frequent absence of high affinity DNA recognition sequences for proteins bound to a given region. Like protein-DNA interactions, protein-protein interactions are enhanced when protein concentrations are high and, thus, *in vivo *could also mediate low level binding at non-functional sites.

How is it possible to have so much binding that has no or little effect on transcription? Natural selection clearly acts on CRMs to preserve the proper number, arrangement and affinity of recognition sequences for whichever factors are needed for its activity. There is also evidence that selection acts against sites that might interfere with activity [62]. Purifying selection will remove any 'spurious' binding that interferes with the proper expression of a gene. But weak binding that has only a small or no affect on transcription could well be tolerated in many cases. Just as there is a quantitative continuum of binding, there may also be a continuum of effects on transcription, and ultimately on phenotype.

## Conclusions

We have mapped genome-wide *in vivo *DNA binding for the largest group to date of animal transcription factors acting in a given tissue at the same time. The work supports and extends our previous studies indicating that animal sequence-specific transcription factors bind *in vivo *across a quantitative continuum to highly overlapping regions close to a large percentage of genes [11,31]. Highly bound genes include strongly regulated known and likely targets, moderately bound genes include unexpected targets whose transcription is regulated weakly, and poorly bound genes include thousands of non-transcribed genes and likely non-functional targets [9-11,22,31]. Factors with distinct biological specificities have highly overlapping patterns of binding. However, quantitative differences in binding to common targets generally correlate with each factor's known specificity, though these specificities appear to be more fuzzy and less distinct than commonly assumed, with a high proportion of shared targets. We propose that the broad DNA recognition properties of animal transcription factors and the relatively high concentrations at which they are expressed in cells focuses them to bind to highly overlapping sets of open chromatin regions. Our work illustrates that the qualitative analyses of *in vivo *DNA binding data that have widely been employed fail to reveal some of the most significant features of how transcriptional regulators behave in cells, and highlights the importance of a detailed quantitative interpretation of DNA binding patterns.

## Materials and methods

### *In vivo *formaldehyde crosslinking of embryos and chromatin purification

Embryos were collected in population cages for 1 hour, and then allowed to develop to the required stage before being harvested and fixed with formaldehyde [11]. Embryo aging times were determined based on the transcription factor analyzed: for A-P maternal, gap, and terminal factors, as well as D-V maternal and a subset of D-V zygotic factors, including SNA and TWI, the embryos used were 2 to 3 hours old (mainly between late stage 4 and early stage 5), while for A-P pair rule, and the D-V zygotic factors, MED, MAD, and Schnurri (SHN), the embryos were 2.5 to 3.5 hours old (mainly at mid- to late stage 5). The chromatin used for immunoprecipation was isolated from the fixed embryos by CsCl gradient ultracentrifugation and then fragmented to an average size of about 700 bp.

### Affinity purified antibody production

All of the antibodies used were immunoaffinity purified from rabbit antiserum. The two anti-PRD antibodies, PRD 1 and PRD 2, were available from a previous study [31]. The MAD and MED antisera were a generous gift from L Raftery [63], the RUN antiserum from E Wieschaus, and the TFIIB antibody from R Tjian. For other factors, antibodies were produced in rabbits immunized with recombinant His-tagged fusion proteins expressed and purified in *Escherichia coli *using the Invitrogen Gateway system. Rabitts were immunized with either the full length protein (Dichaete (D), HRY, SLP1, Daughterless (DA), DL, SNA, and TWI) or portions of the protein (TLL amino acids 110 to 259, SHN amino acids 1,617 to 1,750, and SHN amino acids 2115 to 2,279). Immunoaffinity purifications were performed using *E. coli*-expressed purified recombinant His-tagged proteins. The amino acid sequences used (listed in Table 2) were chosen to exclude regions with any significant homology to other *Drosophila *proteins, as previously described [11]. Additional results demonstrating the specificity of the antibodies are provided in Additional data file 1.

### Chromatin immunoprecipitation and DNA hybridization to high density microarrays

Chromatin was immunoprecipitated and the resulting DNA was amplified and hybridized to Affymetrix *Drosophila *Genomic Tiling Arrays as previously described [11]. For each antibody, duplicate immunoprecipitations were performed along with duplicate control IgG immunoprecipitations. These were each hybridized to separate arrays as were duplicate input DNA samples. All raw microarray data (CEL files) have been deposited at Array Express [E-TABM-736] [64]. In addition, these and more processed forms of the data are available from the BDTNP's public web site, together with more detailed information about antibodies used and so on [65].

### Primary array analysis

The data from the complete set of six arrays from each ChIP/chip experiment were processed using TiMAT [66] as described previously [11] to derive peak window locations, bound regions, 1% and 25% FDR thresholds for both the IgG and Symmetric null tests, and so on. Bound regions were associated with the gene (from release 4.3 of the *D. melanogaster *genome) whose 5' end was closest to the array intensity peak in the bound region. To identify the closest transcribed gene, the subset of release 4.3 annotations that completely overlap regions bound by RNA polymerase II in our ChIP-chip experiments was used.

### Correlation between ChIP/chip and UV crosslinking results

Relative percentages of UV crosslinking to defined restriction fragments [31] and the corresponding mean oligo ChIP/chip scores of the same genomic regions are plotted as scatter plots in Additional data file 5. Pearson correlation coefficients were calculated for each plot.

### Analysis of enrichment down the rank lists of recognition sequences, GO terms, distance to transcribed genes, genomic locations, and PhastCons scores

Enrichment of recognition sequences, GO terms, distance to transcribed genes, genomic locations and phastcons scores were determined essentially as described in [11]. A statistical analysis of the significance of these plots is presented in Additional data file 4.

### Distribution of ChIP/chip peak scores

In Figure 4, peaks were distributed by the mean ChIP/chip peak scores in the 500-bp peak window. For A-P early factors, a peak was associated with A-P early CRMs if the peak single nucleotide position was contained within one of the CRMs extended by 250-bp flanking regions. For D-V factors, a peak was associated with D-V CRMs in the same way.

### Overlap of bound regions between transcription factors

Overlap of bound regions between two transcription factors in Figure 9 was measured by the percentage of single nucleotide peak locations of one factor contained in 1% FDR bound regions of the other factor. The top 300 peaks (1-300) and separately peaks 301 to 600 of each factor were used in the analysis. Overlap of one factor by multiple factors was measured by the percentage of peaks of that factor contained in 1% FDR bound regions of a defined number of other factors (Additional data file 13).

To calculate the liklihood z score that overlap occurs by chance (Figures 9c, d and 13a, b), z-scores were computed using the GSC statistics [43]. A null distribution of feature-feature overlap was computed by selecting pair-wise block samples from the genome, and in each block in the pair the annotations of one of the two features of interest were swapped to yield artificial overlaps. The resulting null distribution is more realistic than that derived from other methods in that the complex and often tightly clustered organization of each feature across the genome is preserved, resulting in a much larger (conservative) estimate of standard deviation than derived via other methods. Like most methods, including feature start-site randomization, this null is Gaussian, and hence after centering, the only quantity that needs to be estimated is precisely the standard deviation.

### Heat map analysis of binding of transcription factors to CRMs

In Figure 12a a CRM is defined as being bound by a transcription factor if it was overlapped by at least 300 bp by one of the factor's 1% FDR bound regions, or for CRMs less than 300 bp long, if the CRM was completely overlapped by a 1% FDR region. In Figure 12b, the binding intensity of a transcription factor to a CRM is defined by the highest 675-bp smoothed window score in the CRM for that factor, without regard to FDR threshold. The window scores for each factor were placed on the same scale by setting the highest 675-bp window contained on the whole array data to 10.

### Heat map analysis of correlation of scores of bound regions between transcription factors

In Figure 13c, d, for each transcription factor, the score associated with each 500-bp peak window was derived from the mean score of oligos in the window. The scores for the equivalent 500-bp windows for each of the other 20 factors were then derived from the mean oligo scores from those datasets, without regard to any FDR threshold. Scores for each pair-wise comparison of factors were used to calculate the Pearson correlation between the top 300 bound regions (1-300) and separately for regions from 301 to 600 on the ChIP/chip rank list. Because the original data for the PRD 1 antibody was derived from a different array scanner than that used for the other factors and because we found that a subtle scaling difference between the two scanners affected the correlation coefficients, the PRD 1 data used in all of Figure 13 were from a replica set (PRD 1*) that used the same Affymetrix G7 scanner used to derive data for the other factors.

### Mann-Whitney tests

Mann-Whitney tests were applied to the binding intensity data of transcription factors to CRMs (Figure 12b), overlap GSC Z scores between factors (Figure 13a, b), and Pearson correlation of intensity scores of peak windows between factors (Figure 13c, d) and are reported in Additional data file 13. Each data set was divided into two categories by factor regulatory classes. The Mann-Whitney test was one-sided, with the null hypothesis that the two categories of data followed the same distribution. Bonferonni corrected values are provided where stated.

### Heat map analyses of the association of bound regions with GO terms

In Figure 15, each bound region is associated with the 'biological process' GO term for the gene whose transcription start site was closest to the array intensity peak in the bound region. The non-redundant set of the 7 most enriched GO terms associated with the top 300 bound regions of each factor were used in the analysis. Negative logged probabilities from a hypergeometric distribution were used to measure the association of the top 1 to 300 and 301 to 600 bound regions of each factor with a GO term. The scores of different factors were put on the same scale by setting the most enriched value to 10.

## Abbreviations

A-P: anterior-posterior; BDTNP: Berkeley *Drosophila *Transcription Network Project; ChIP/chip: chromatin immunoprecipitation followed by microarray analysis; CRM: *cis*-regulatory module; D-V: dorsal-ventral; FDR: false discovery rate; GO: Gene Ontology; GSC: Genome Structure Correction.

## Authors' contributions

XL, SM, JL, JBB, PB, MBE and MDB conceived and designed the experiments and analyses and wrote the paper. XL, HCC, and SVEK performed the wet laboratory experiments. SM, XL, JL, JBB, AH, PB, MBE and MDB analyzed the data. LS, XL, SM, MDB and MBE designed the database. AH, LZ, BDG, MS, SVEK, XL, HCC, DWK, MBE and MDB contributed reagents/materials/analysis tools. All authors read and approved the final manuscript.

## Additional data files

The following additional data are available with the online version of this paper: further evidence that the antibodies used specifically recognize the transcription factors they were raised against in the embryo (Additional data file 1); a table that shows for each factor the numbers of genomic regions bound in blastoderm embryos determined by the symmetric null FDR test and the IgG control FDR test (Additional data file 2); figures plotting down the ChIP/chip rank list in 200-peak cohorts the enrichment of factor recognition sequences using the conventions shown in Figure 2 (Additional data file 3); a table that shows statistical evidence that the top 200 ChIP/chip peaks are significantly enriched over all peaks in the 1% FDR set for the values plotted in Figures 2, 5, 6 and 8 (Additional data file 4); scatter plots comparing relative levels of mean UV crosslinking and mean ChIP/chip scores across a series of highly and poorly bound genomic regions (Additional data file 5); tables listing the genomic coordinates of regions bound by each factor for the 1% FDR data set, and information on the locations and scores of peak windows, and on the closest gene and closest transcribed gene for each peak (Additional data file 6); tables listing the genomic coordinates of regions bound by each factor for the 25% FDR data set, and information on the locations and scores of peak windows, and on the closest gene and closest transcribed gene for each peak (Additional data file 7); figures showing the fraction of bound regions in different cohorts distinguished by ChIP/chip score and, for some factors, the fraction of those bound regions that overlap known CRMs, using the conventions shown in Figure 4 (Additional data file 8); figures plotting down the ChIP/chip rank list in 200-peak cohorts the five most highly enriched GO terms of the closest gene using the conventions shown in Figure 5 (Additional data file 9); figures plotting down the ChIP/chip rank list in 200-peak cohorts the median distance to the closest gene and the distances to closest genes transcribed or patterned in blastoderm embryos using the conventions shown in Figure 6 (Additional data file 10); figures plotting down the ChIP/chip rank list in 200-peak cohorts the percent of peaks found in intergenic, intronic and protein coding regions using the conventions shown in Figure 7 (Additional data file 11); figures plotting down the ChIP/chip rank list in 200-peak cohorts the PhastCons scores of 500-bp peak windows using the conventions shown in Figure 8 (Additional data file 12); tables listing the values plotted in the heat maps in Figures 9, 12 and 13, percentages of the top 300 1% FDR peaks bound by 1, 8 or more, 15 or more or 21 factors, and the results of Mann-Whitney tests applied to the data in Figures 12 and 13 (Additional data file 13); a figure showing the pattern of ChIP/chip scores on the *eve *gene for both factor and negative control immunoprecipitations for all antibodies shown in Table 2 (Additional data file 14).

## Supplementary Material

Additional data file 1 The antibodies used specifically recognize the transcription factors they were raised against in the embryo. Click here for file

Additional data file 2 See Table 2, Li *et al*. [11], and Materials and methods for further details. Click here for file

Additional data file 3 These are plotted down the ChIP/chip rank list in non-overlapping 200-peak cohorts. Click here for file

Additional data file 4 The top 200 ChIP/chip peaks are significantly enriched over all peaks in the 1% FDR set for the values plotted in Figures 2, 5, 6 and 8. Click here for file

Additional data file 5 Relative levels of mean UV crosslinking and mean ChIP/chip scores across a series of highly and poorly bound genomic regions. Click here for file

Additional data file 6 Genomic coordinates of regions bound by each factor for the 1% FDR data set, locations and scores of peak windows, and the closest gene and closest transcribed gene for each peak. Click here for file

Additional data file 7 Genomic coordinates of regions bound by each factor for the 25% FDR data set, locations and scores of peak windows, and the closest gene and closest transcribed gene for each peak. Click here for file

Additional data file 8 Fraction of bound regions in different cohorts distinguished by ChIP/chip score and, for some factors, the fraction of those bound regions that overlap known CRMs, using the conventions shown in Figure 4. Click here for file

Additional data file 9 These are shown plotted down the ChIP/chip rank list in non-overlapping 200-peak cohorts. Click here for file

Additional data file 10 These are plotted down the ChIP/chip rank list in non-overlapping 200-peak cohorts. Click here for file

Additional data file 11 These are plotted down the ChIP/chip rank list in non-overlapping 200-peak cohorts. Click here for file

Additional data file 12 These are plotted down the ChIP/chip rank list in non-overlapping 200-peak cohorts. Click here for file

Additional data file 13 Values plotted in the heat maps in Figures 9, 12 and 13, percentages of the top 300 1% FDR peaks bound by 1, 8 or more, 15 or more or 21 factors, and the results of Mann-Whitney tests applied to the data in Figures 12 and 13. Click here for file

Additional data file 14 The pattern of ChIP/chip scores on the *eve *gene for both factor and negative control immunoprecipitations for all antibodies shown in Table 2. Click here for file
