# Supplementary material for: Developmental roles of 21 Drosophila transcription factors are determined by quantitative differences in binding to an overlapping set of thousands of genomic regions
Source: Genome Biol. 2009 Jul 23;10(7):R80. doi: 10.1186/gb-2009-10-7-r80 (PMC2728534; doi:10.1186/gb-2009-10-7-r80)

Additional evidence for the specificity of the antibodies used

All of the antibodies used in our ChIP-chip experiments were affinity purified, and the source of each antiserum is as described in Material and Methods. For 11 factors, we were able to obtain antibodies affinity purified against two non-overlapping portions of the protein chosen to avoid amino acid sequence found in other *Drosophila* proteins. Members of the same antibody pair give very similar patterns of ChIP/chip signal, whereas antibodies against different proteins give different patterns (Fig. 1; Table 2). Thus, these antibodies do not cross-react with other proteins and are specific to the factor that they were raised against. Here we describe and show additional data for antibodies against all 21 factors used in our ChIP/chip experiments, showing that they specifically recognize the protein that they were raised against in early embryos.

For five proteins, the actual antisera used in our affinity purifications had previously been shown to give the expected protein staining pattern in embryos. For these, we provide references to the publications showing these pattern. For antibodies against the remaining factors, we have performed immuno histochemical embryo staining and found that they produce staining that matches the expected expression patterns described previously in the literature for mRNA and/or protein. Embryo staining and imaging was performed essentially as described in Luengo Hendriks et al, 2006 [10]. The views shown are all sections from a 3D confocal image that have been collapsed to 2D. All of the embryo images are shown with dorsal or dorsolateral up, and anterior to the left. Information is given for each factor in turn in alphabetical order.

BCD: The BCD1 and BCD2 antibodies were affinity purified from a serum from P. MacDonald and G. Struhl. The BCD antibody was used for immuno histochemical embryo staining, which produced an anterior to posterior gradient that is consistent with the known protein expression pattern ([71] and data not shown). Moreover, the UV crosslinking signal from experiments using either of these purified antibodies is only seen in blastoderm stage embryos, not in 8-12 hr embryos, which do not express BCD [31].

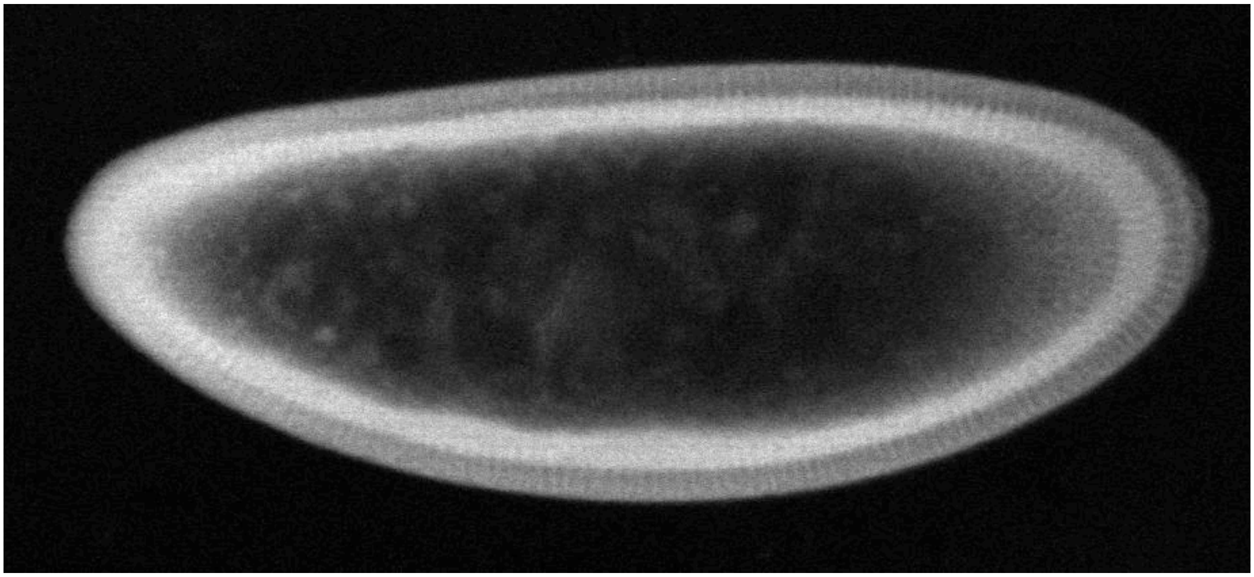

CAD: The CAD 1 antibody was affinity purified from a serum previously shown to give the expected pattern of CAD protein expression in embryos [72].

D: The affinity purified D 1 antibody was used for embryo staining. The staining pattern shown in the image is as expected based on published reports for D protein and mRNA expression [73-75].

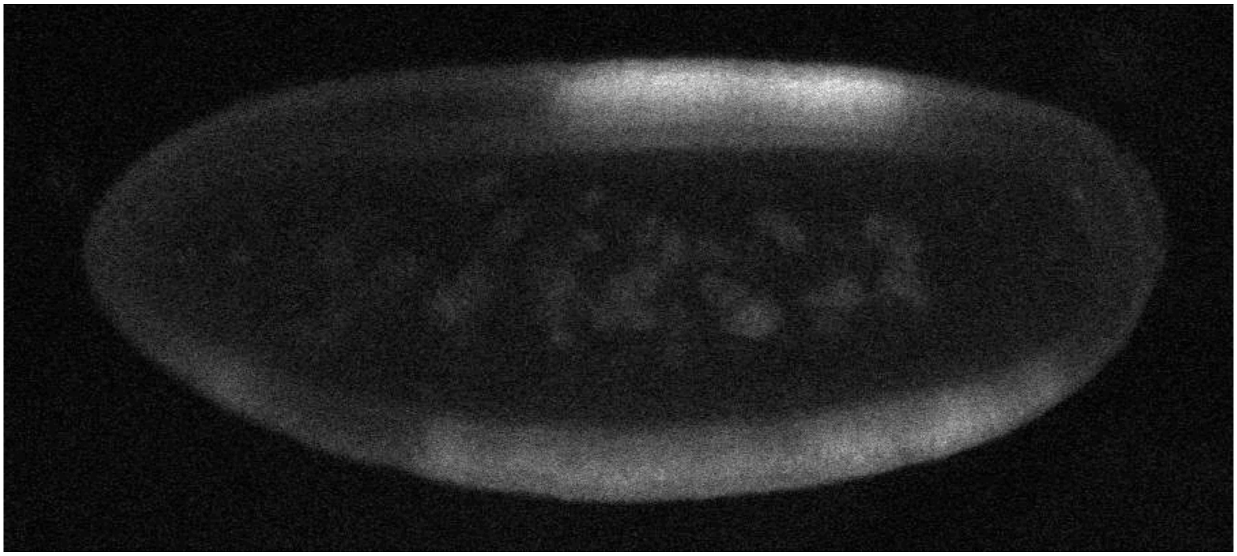

DA: The affinity purified DA 2 antibody was used for embryo staining. The staining shown in the image is as expected for DA, which is a ubiquitously expressed nuclear protein [76, 77].

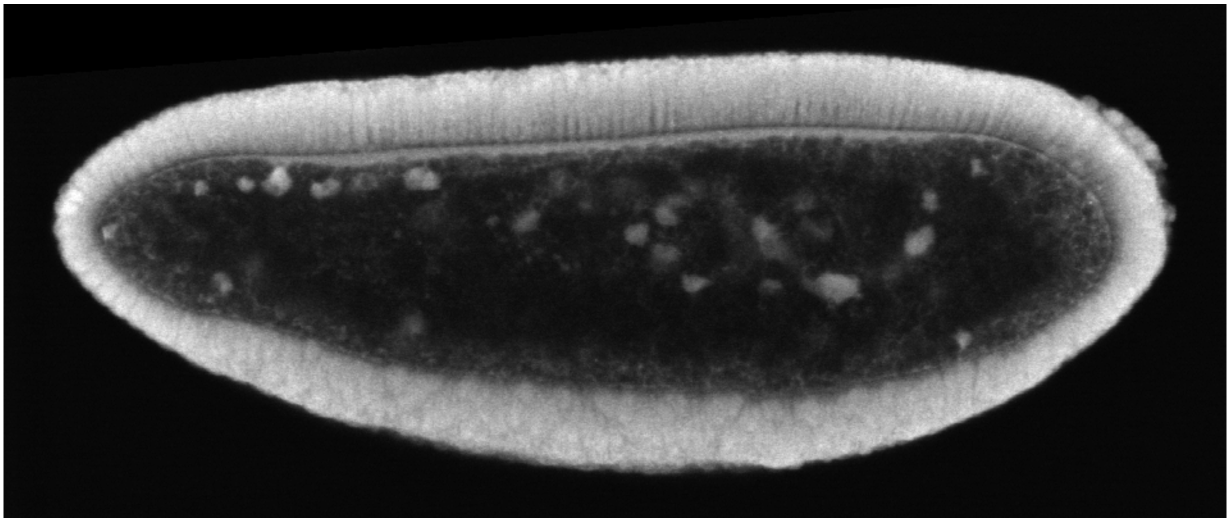

DL: The antiserum that was used for affinity purification of the DL 3 antibody was used for embryo staining. The staining shows the expected ventral – dorsal nuclear gradient of protein localization [78, 79].

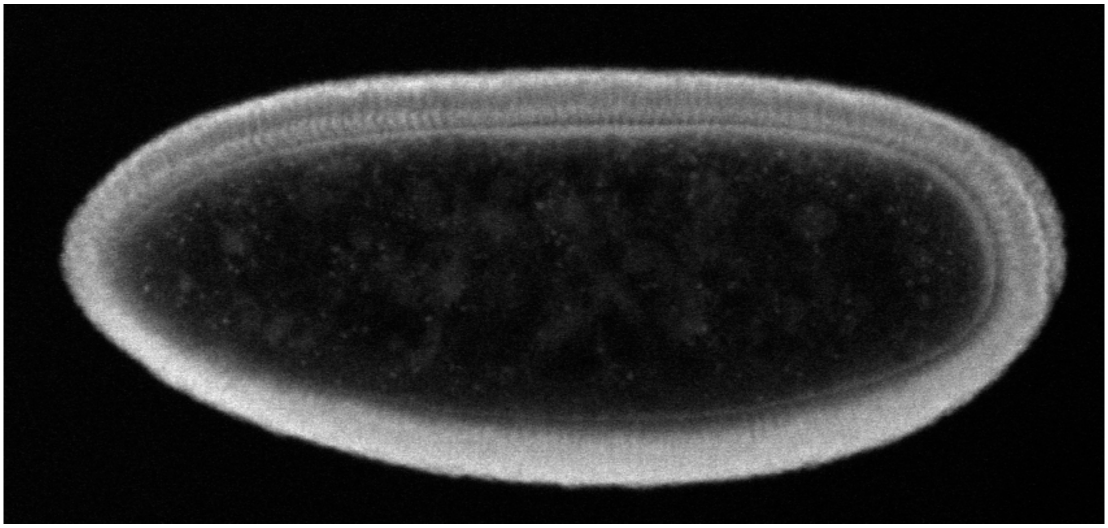

FTZ: The FTZ 3 antibody was affinity purified from a serum previously shown to give the expected expression pattern in embryos [80, 81]. In addition, the UV crosslinking signal obtained using this affinity purified antibody is only seen in blastoderm stage embryos, not in 8-12 hr embryos, which express FTZ in so few cells that no crosslinking signal should be detected [31].

GT: The affinity purified GT 2 antibody was used for embryo staining. The image shows that the staining matches the expected gap pattern for GT protein and mRNA [82] [83, 84].

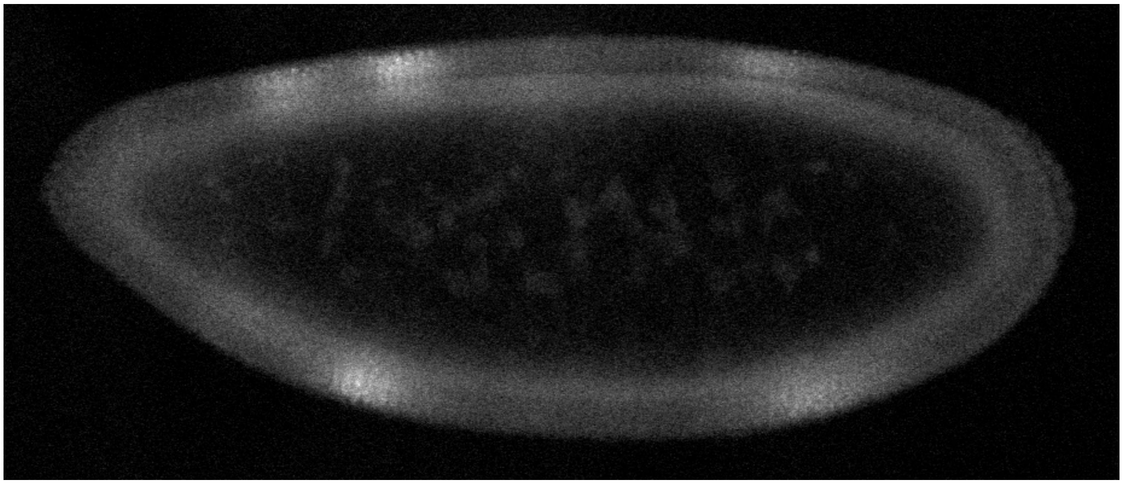

HB: The HB 1 and HB 2 antibodies were affinity purified from a serum previously shown to give the expected expression pattern in embryos [85].

HKB: The affinity purified HKB 2 antibody was used for embryo staining. The image shows that the staining matches the expected terminal nuclear staining pattern for HKB protein and mRNA [86-88].

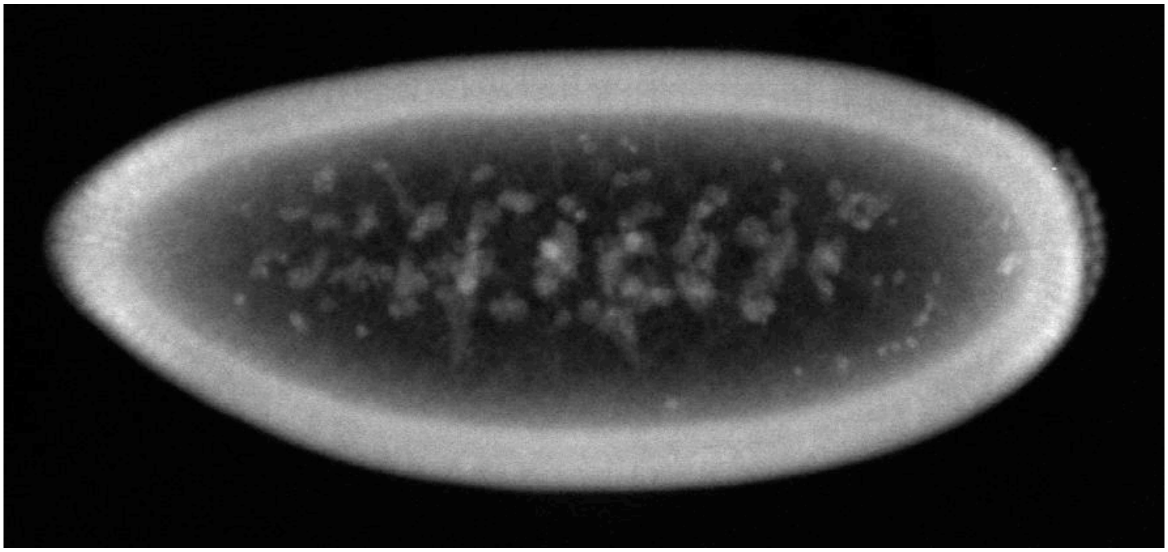

HRY (hairy): The affinity purified HRY 2 antibody was used for embryo staining. The image shows that the staining like the expected pair-rule pattern for hairy protein and mRNA expression [89, 90].

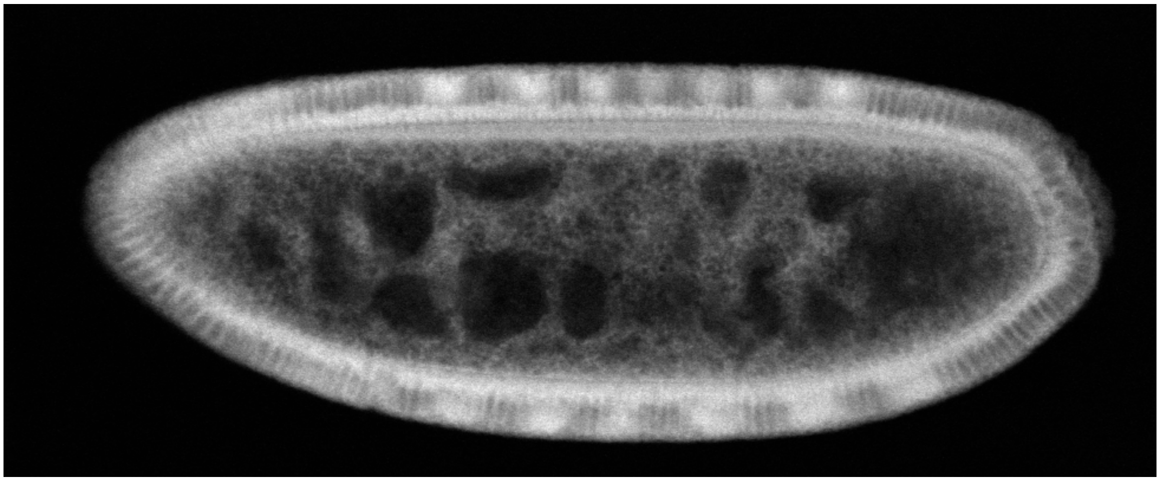

KNI: The affinity purified KNI 2 antibody was used for embryo staining. The image shows that the nuclear staining pattern matches the expected gap pattern for KNI protein and mRNA [91, 92].

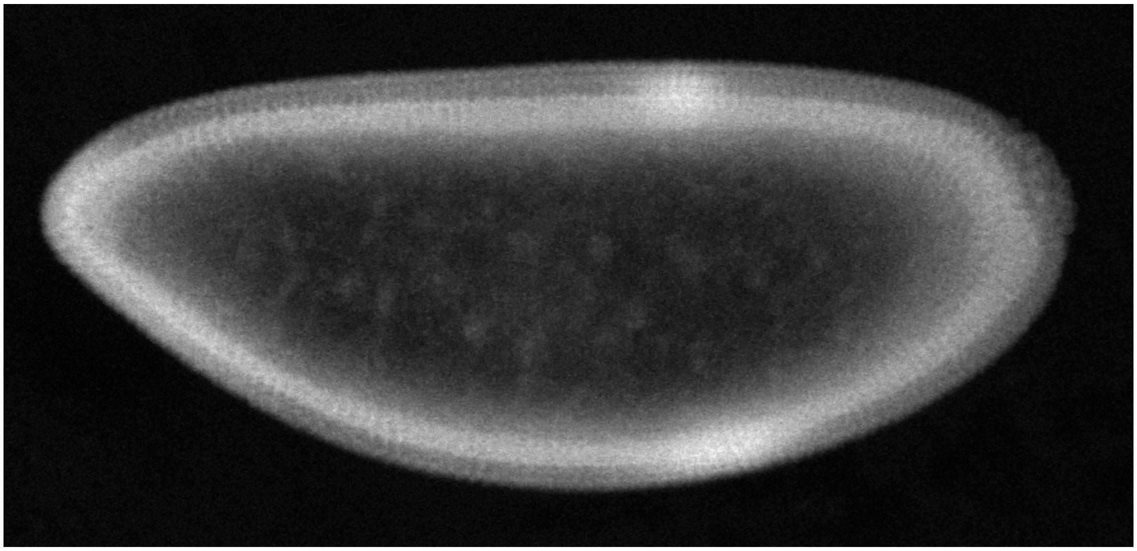

KR. The affinity purified KR 2 antibody was used for embryo staining. The image shows that the staining matches the expected gap pattern for KR protein and mRNA [93, 94].

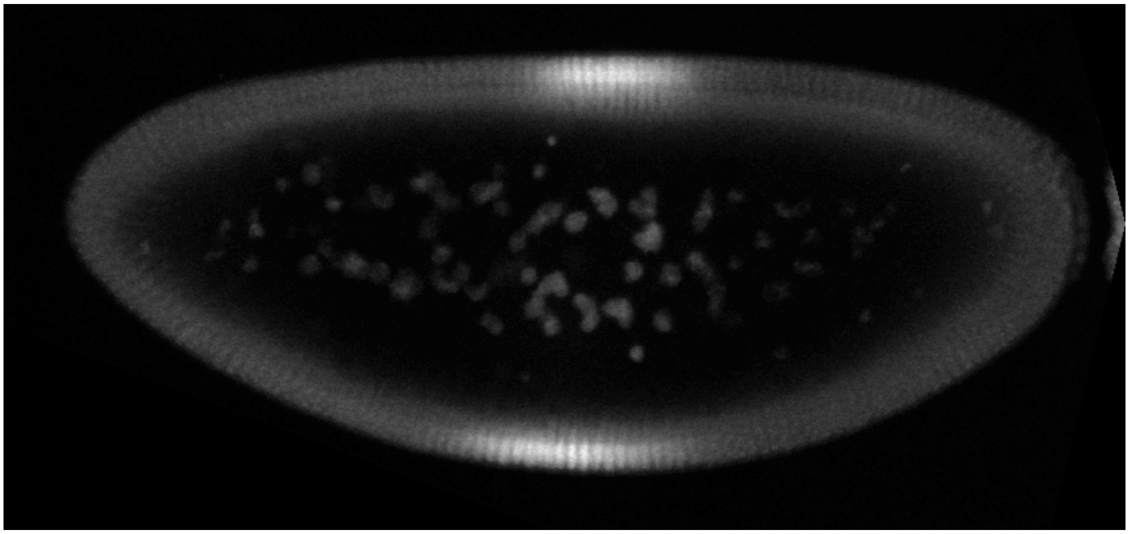

MAD: The affinity purified MAD-2 antibody was used for embryo staining. The image shows clear nuclear localization in a dorsal streak. This is consistent with the fact that while MAD's expression is ubiquitous, it becomes nuclear and is required for nuclear translocation of Medea, a mediator of dorsal Dpp signaling in late stage 5 and older embryos [63, 95, 96].

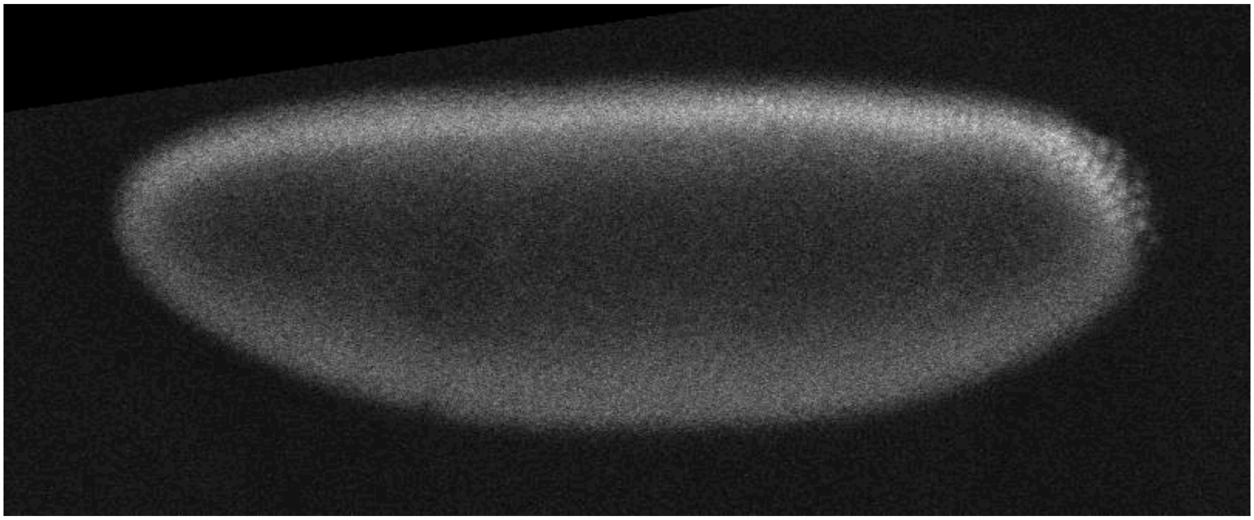

MED: The MED 2 antibody was affinity purified from a serum previously shown to give the expected expression pattern in embryos [63].

PRD: Both the PRD 1 and PRD 2 antibodies were affinity purified from a serum previously shown to detect expression in wild type embryos but not in embryos lacking Prd [97]. In addition, using these affinity purified antibodies in *in vivo* UV crosslinking experiments, a crosslinking signal is only seen in blastoderm stage embryos, not in 8-12 hr embryos, which express PRD in so few cells that no crosslinking signal should be detected [31].

RUN: The affinity purified RUN 1 antibody was used for embryo staining. The pair-rule pattern observed is as expected based on the known protein and mRNA expression patterns [98, 99].

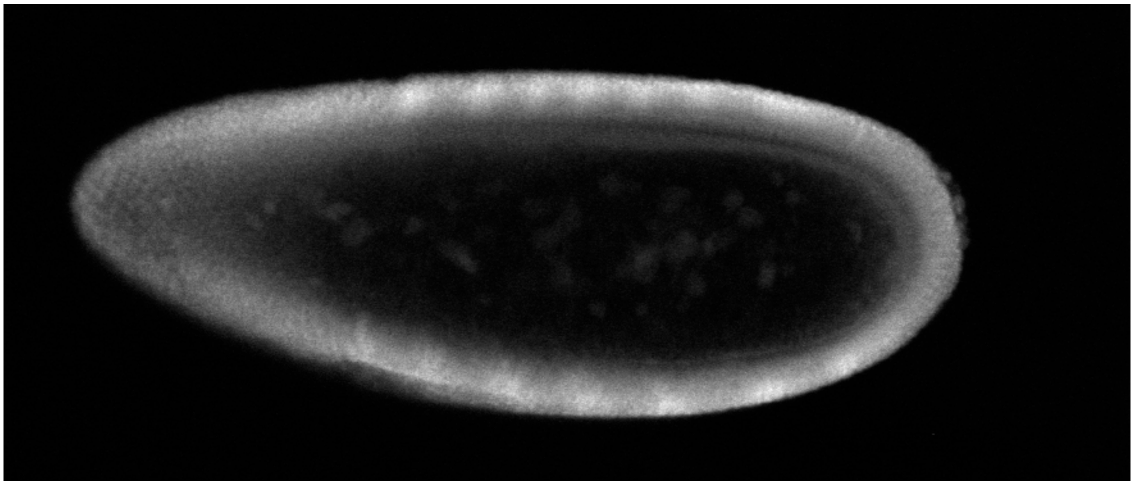

SHN: The affinity purified SHN 3 antibody was used for embryo staining. The dorsal staining pattern we detect is largely consistent with the known *Shn* mRNA transcript expression, which is located in the dorsal region in stage 4 and stage 5 embryos, though the mRNA is also detected ventrally in late stage 5 and upon gastrulation [100-102].

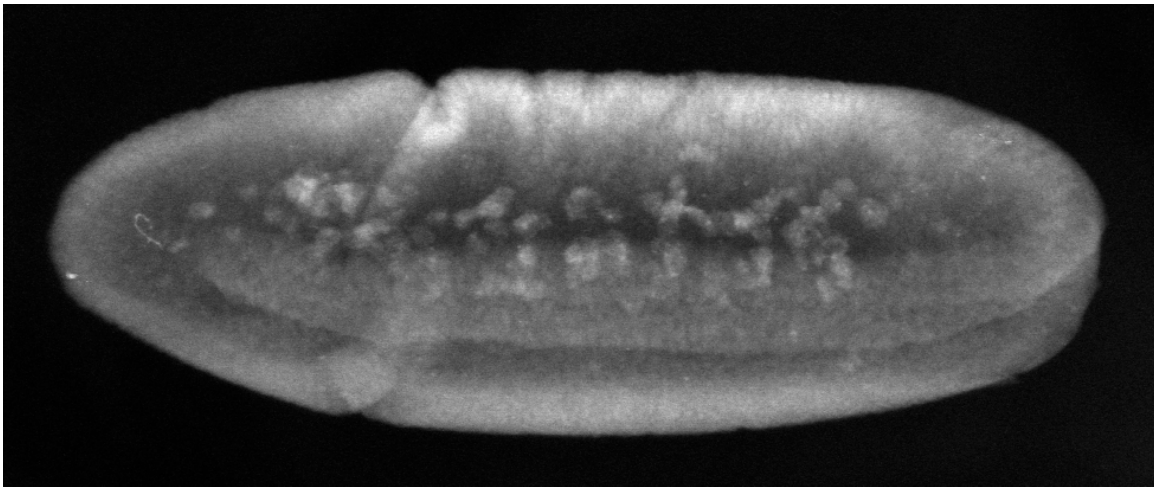

SLP1: The affinity purified SLP1 1 antibody was used for embryo staining. The pattern shown is consistent with the known Slp1 mRNA and protein expression [103-105].

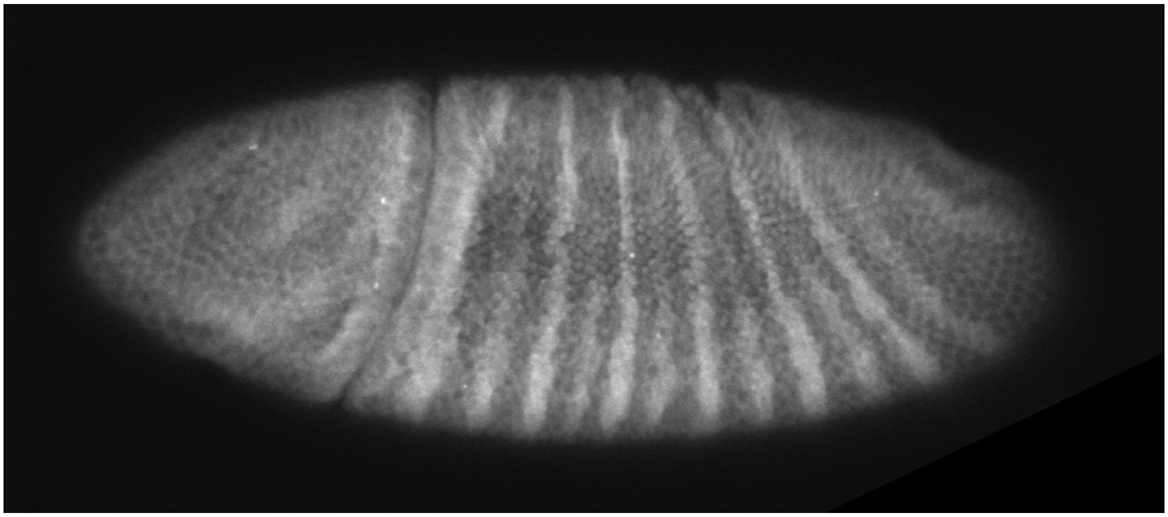

SNA: The anti-SNA serum used for affinity purification of the SNA 1 and SNA 2 antibodies was used for embryo staining. The staining pattern shown in the image is consistent with the known SNA expression pattern, being expressed on the ventral side of the embryo [106-108].

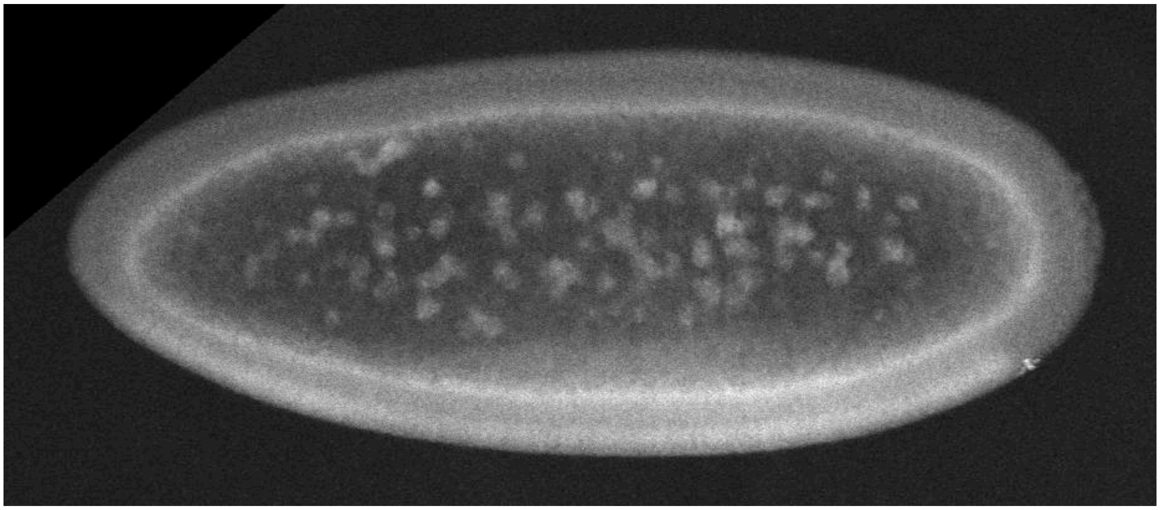

TLL: The affinity purified TLL-I antibody was used for embryo staining. The terminal staining pattern is consistent with the reported TLL protein and mRNA expression patterns [86, 109].

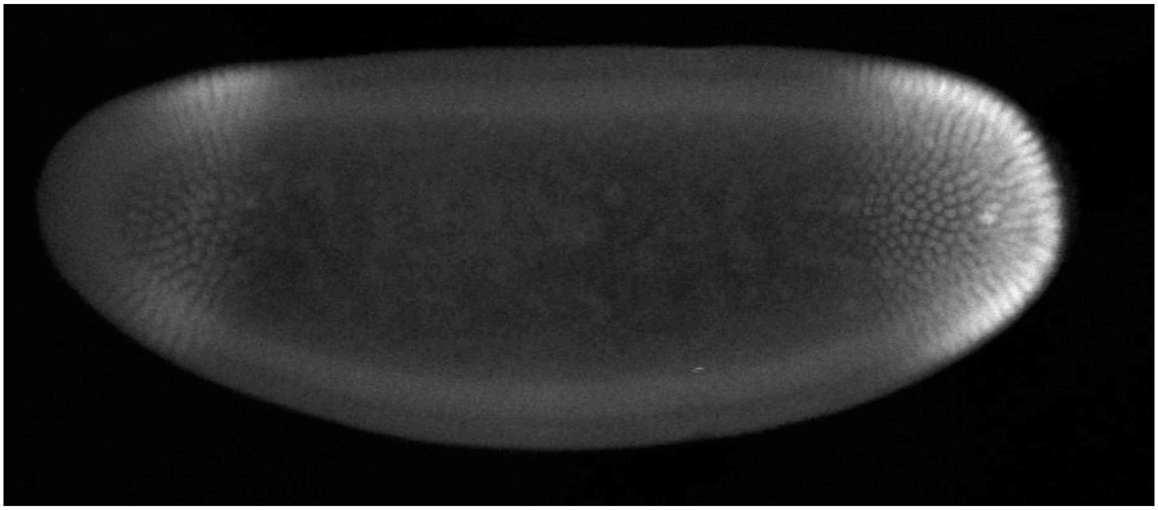

TWI: The affinity purified TWI 2 antibody was used for embryo staining. The expression pattern in the image is consistent with the ventral expression pattern previously shown for this protein [106, 108, 110].

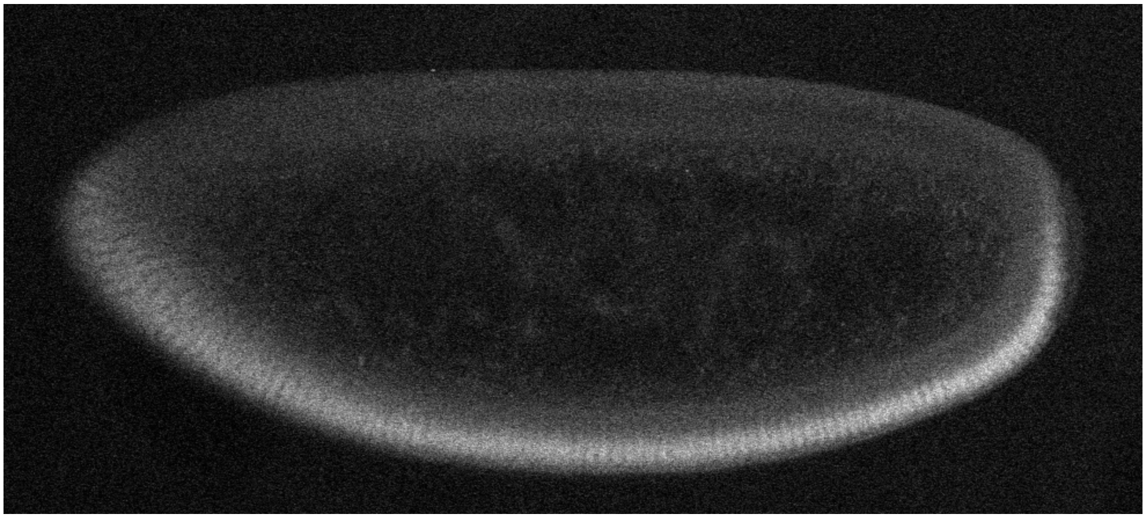

Supplement: Additional data file 1 — The antibodies used specifically recognize the transcription factors they were raised against in the embryo. [file gb-2009-10-7-r80-S1.pdf]
