# Supplementary material for: Developmental roles of 21 Drosophila transcription factors are determined by quantitative differences in binding to an overlapping set of thousands of genomic regions
Source: Genome Biol. 2009 Jul 23;10(7):R80. doi: 10.1186/gb-2009-10-7-r80 (PMC2728534; doi:10.1186/gb-2009-10-7-r80)
Supplement: Additional data file 3 — These are plotted down the ChIP/chip rank list in non-overlapping 200-peak cohorts. [file gb-2009-10-7-r80-S3.pdf]

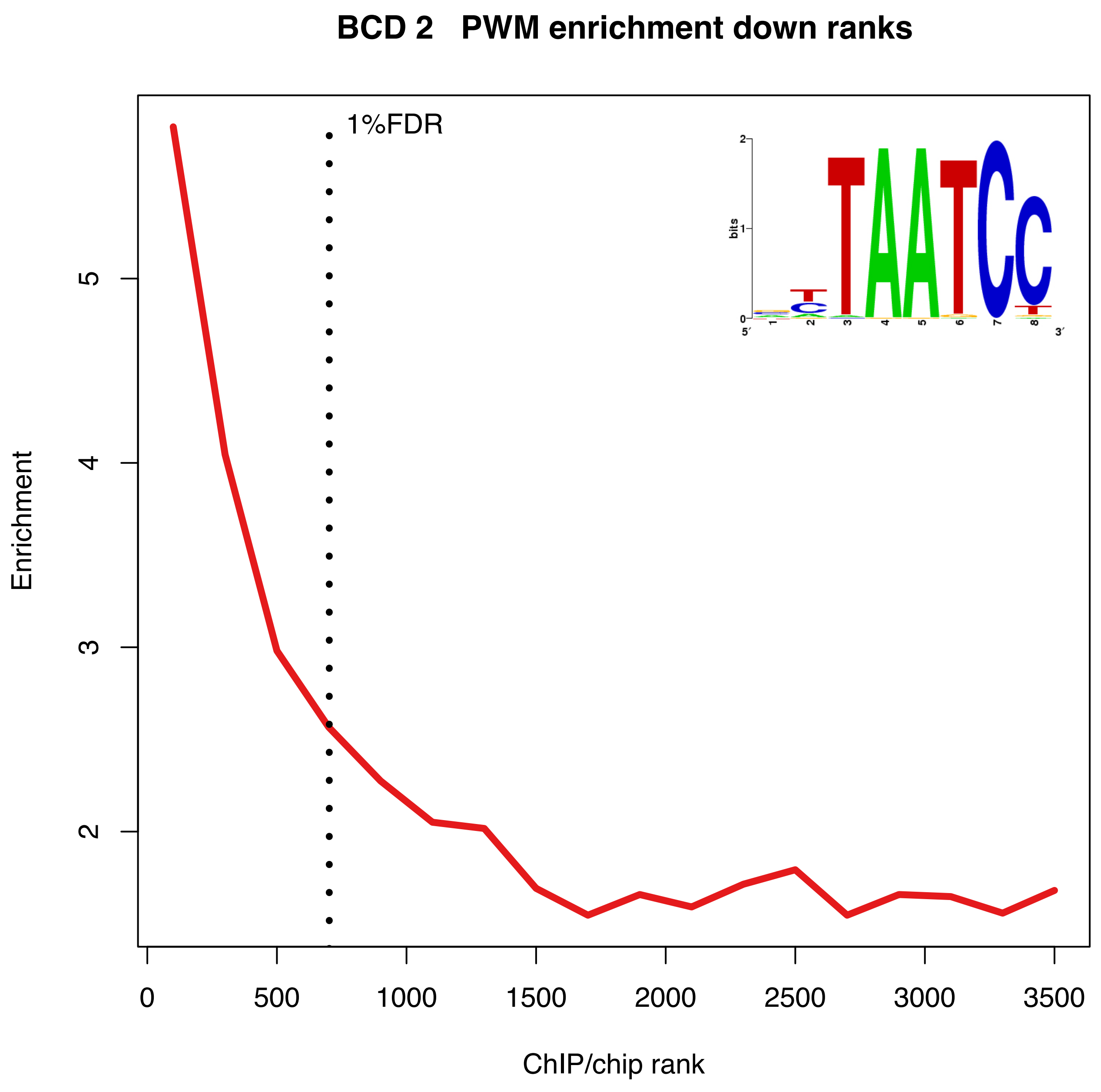
BCD 2 PWM enrichment down ranks
1%FDR
Enrichment
2
3
4
5
0
500
1000
1500
2000
2500
3000
3500
ChIP/chip rank
bits
TAATCc

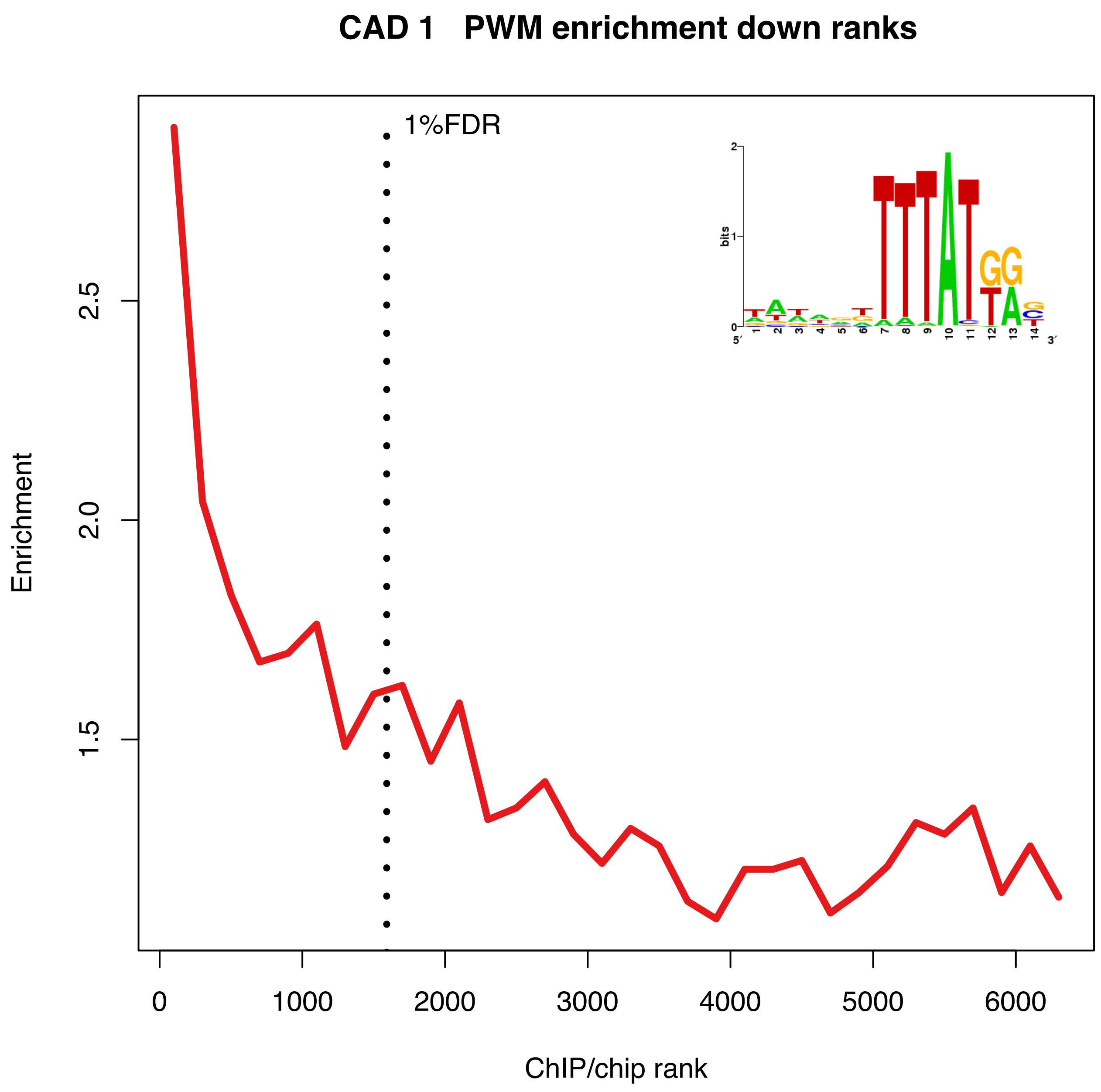
CAD 1   PWM enrichment down ranks
1%FDR
Enrichment
2.5
2.0
1.5
0
1000
2000
3000
4000
5000
6000
ChIP/chip rank
bits

D insufficient in vitro binding data

Da insufficient in vitro binding data

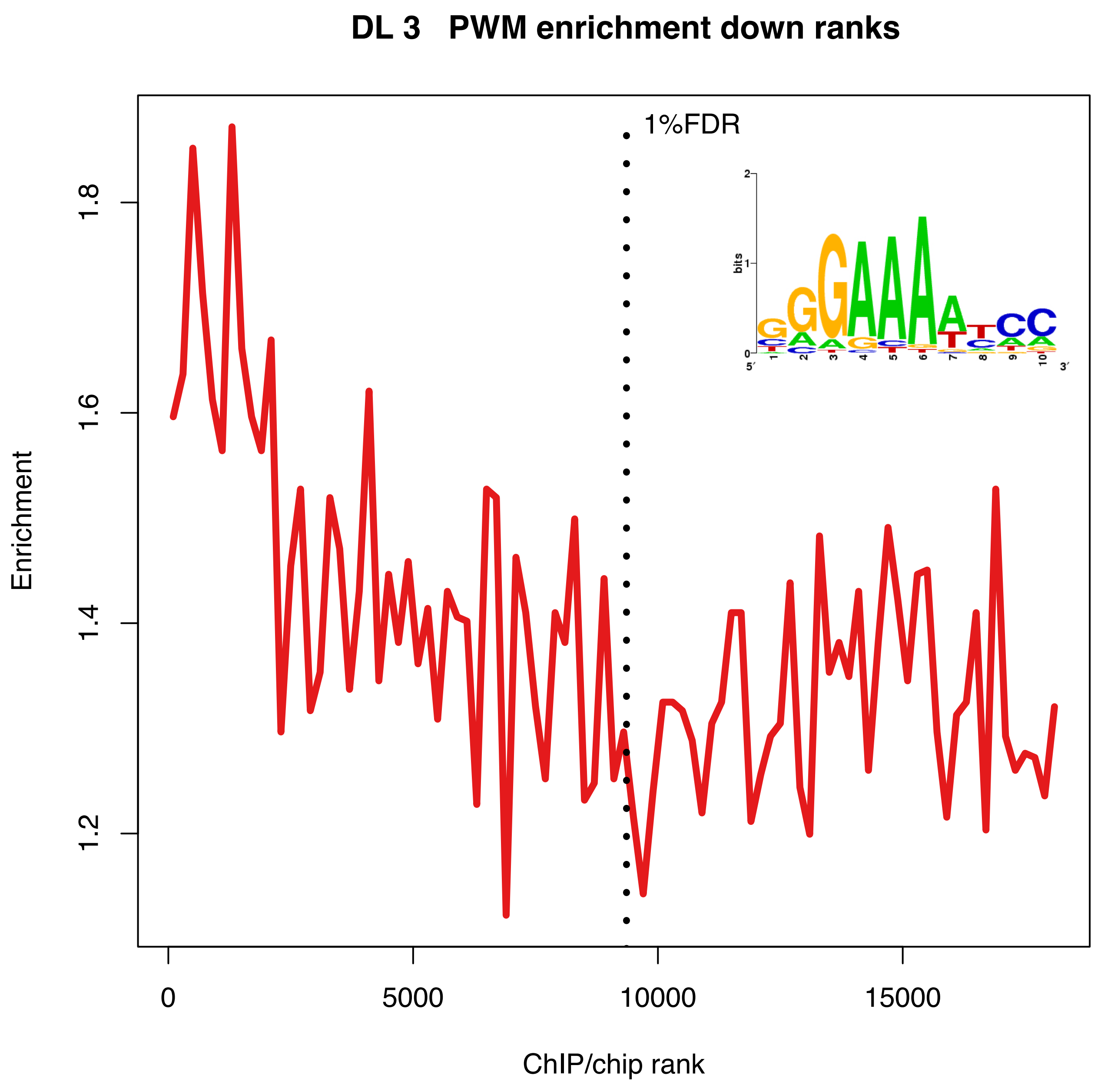
DL 3 PWM enrichment down ranks
1%FDR
Enrichment
1.2
1.4
1.6
1.8
0
5000
10000
15000
ChIP/chip rank
bits

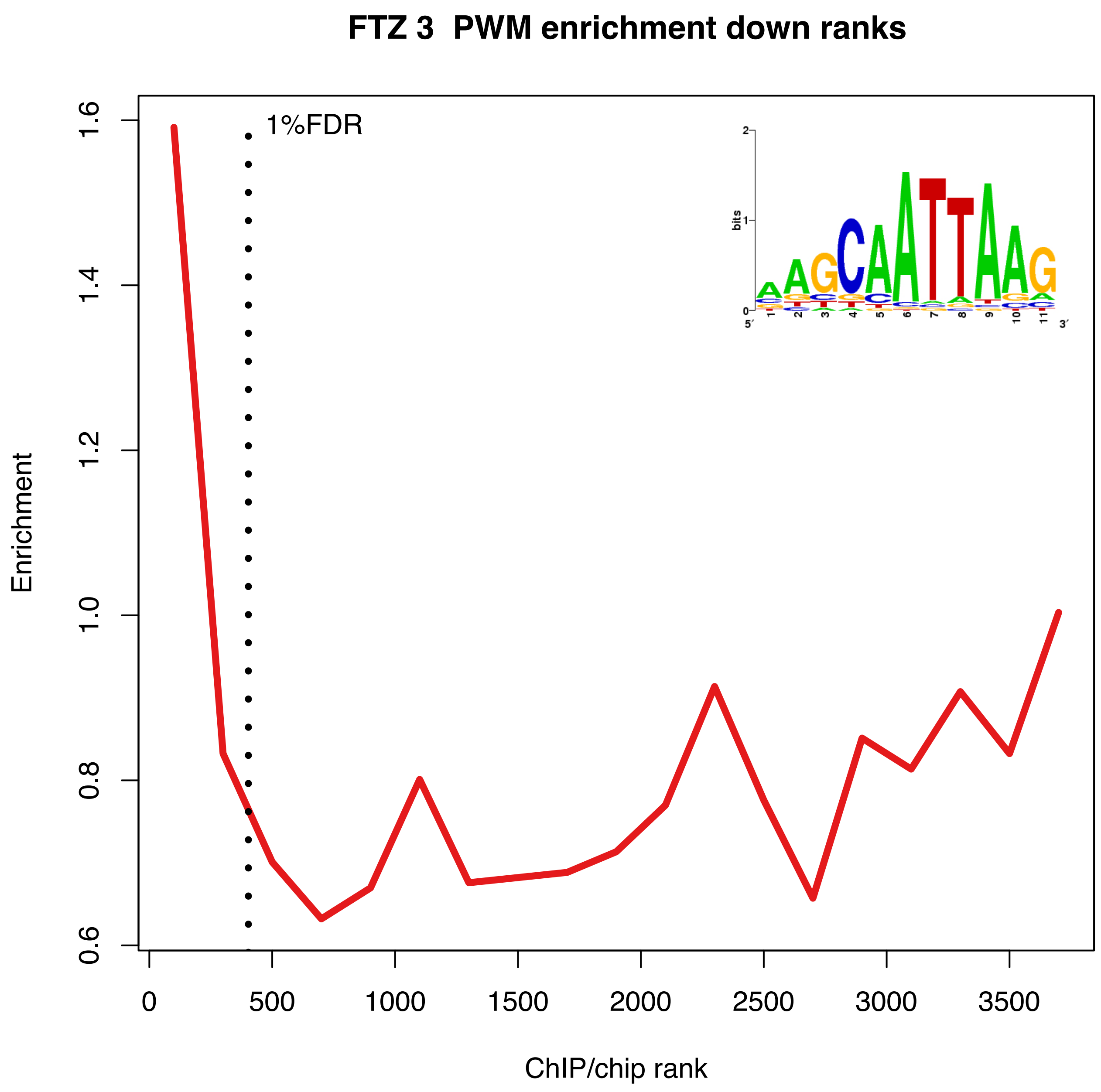
FTZ 3 PWM enrichment down ranks
1%FDR
Enrichment
ChIP/chip rank
1.6
1.4
1.2
1.0
0.8
0.6
0
500
1000
1500
2000
2500
3000
3500
bits

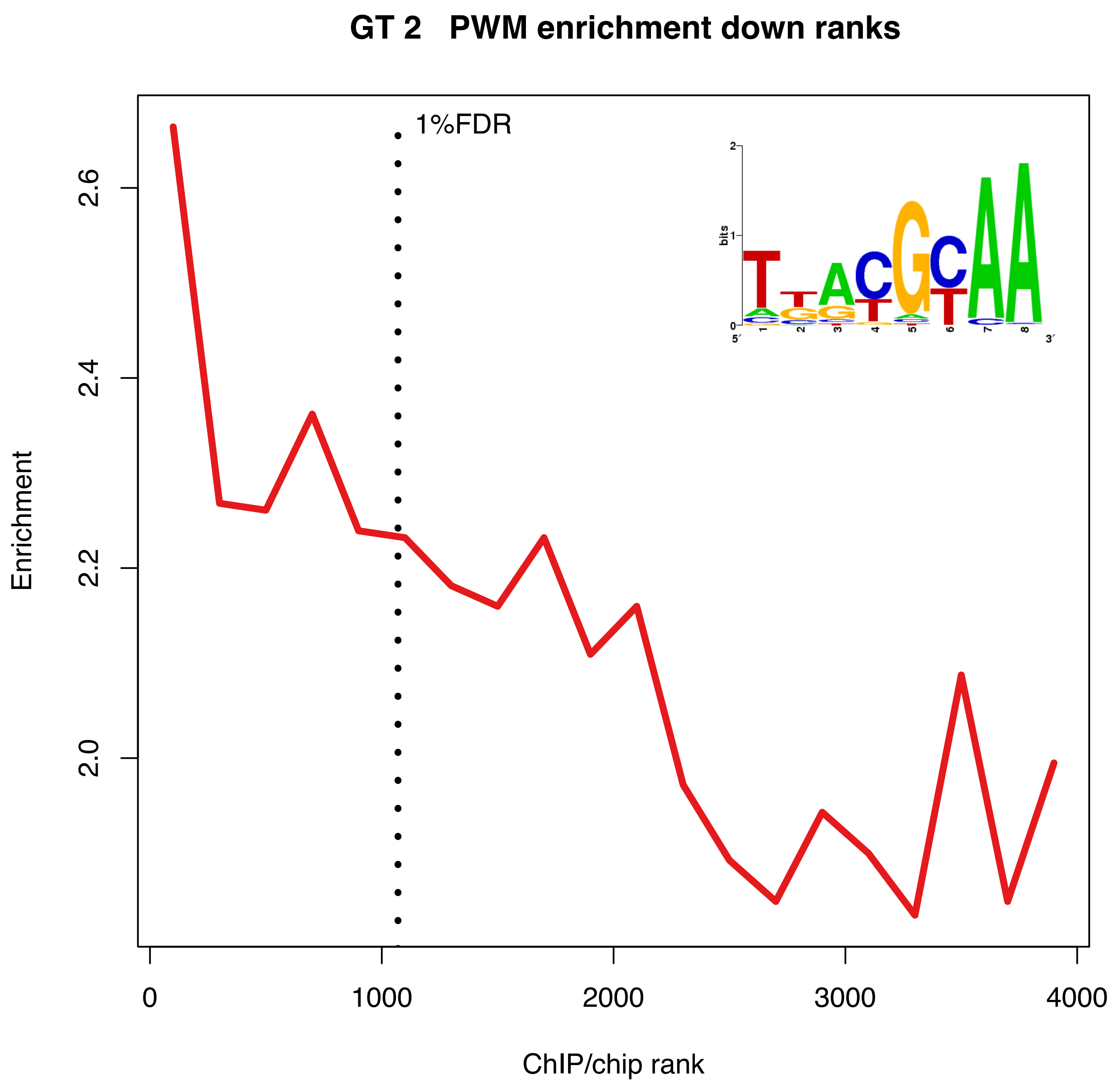
GT 2   PWM enrichment down ranks
1%FDR
Enrichment
2.0
2.2
2.4
2.6
ChIP/chip rank
0
1000
2000
3000
4000
bits

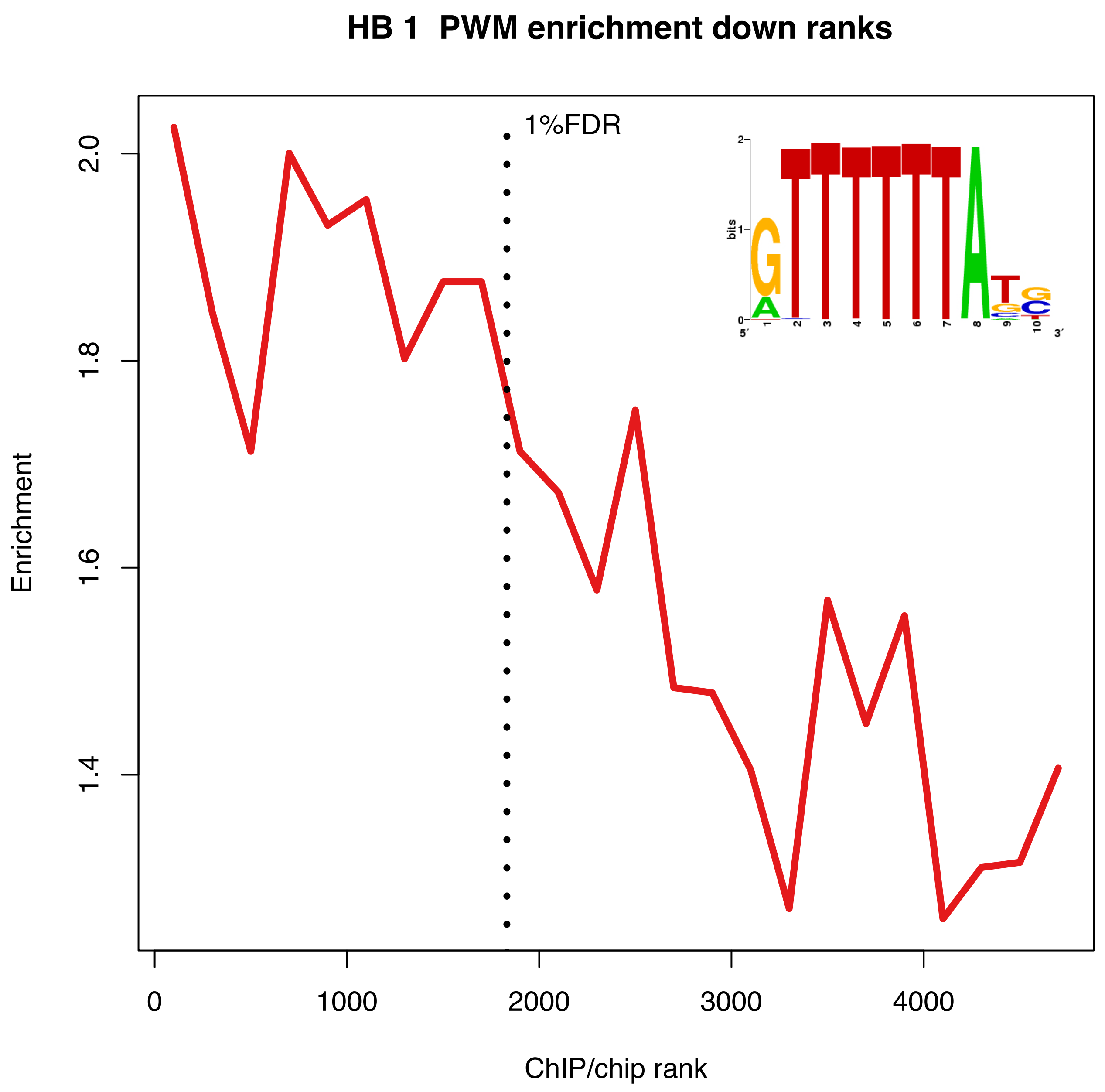
HB 1 PWM enrichment down ranks
1%FDR
Enrichment
ChIP/chip rank
2.0
1.8
1.6
1.4
0
1000
2000
3000
4000
bits

HKB insufficient in vitro binding data

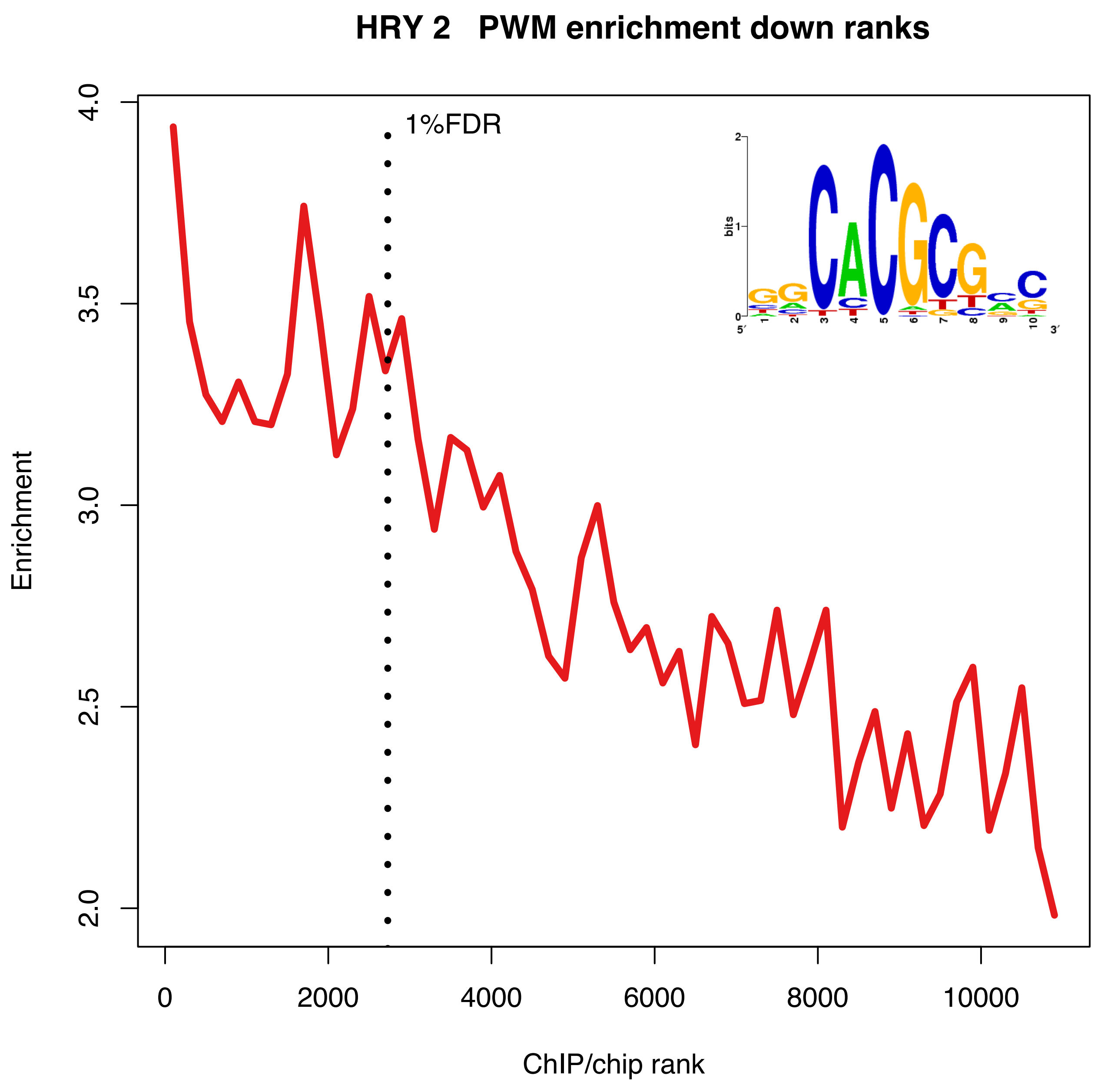
HRY 2   PWM enrichment down ranks
1%FDR
Enrichment
ChIP/chip rank
4.0
3.5
3.0
2.5
2.0
0
2000
4000
6000
8000
10000
bits

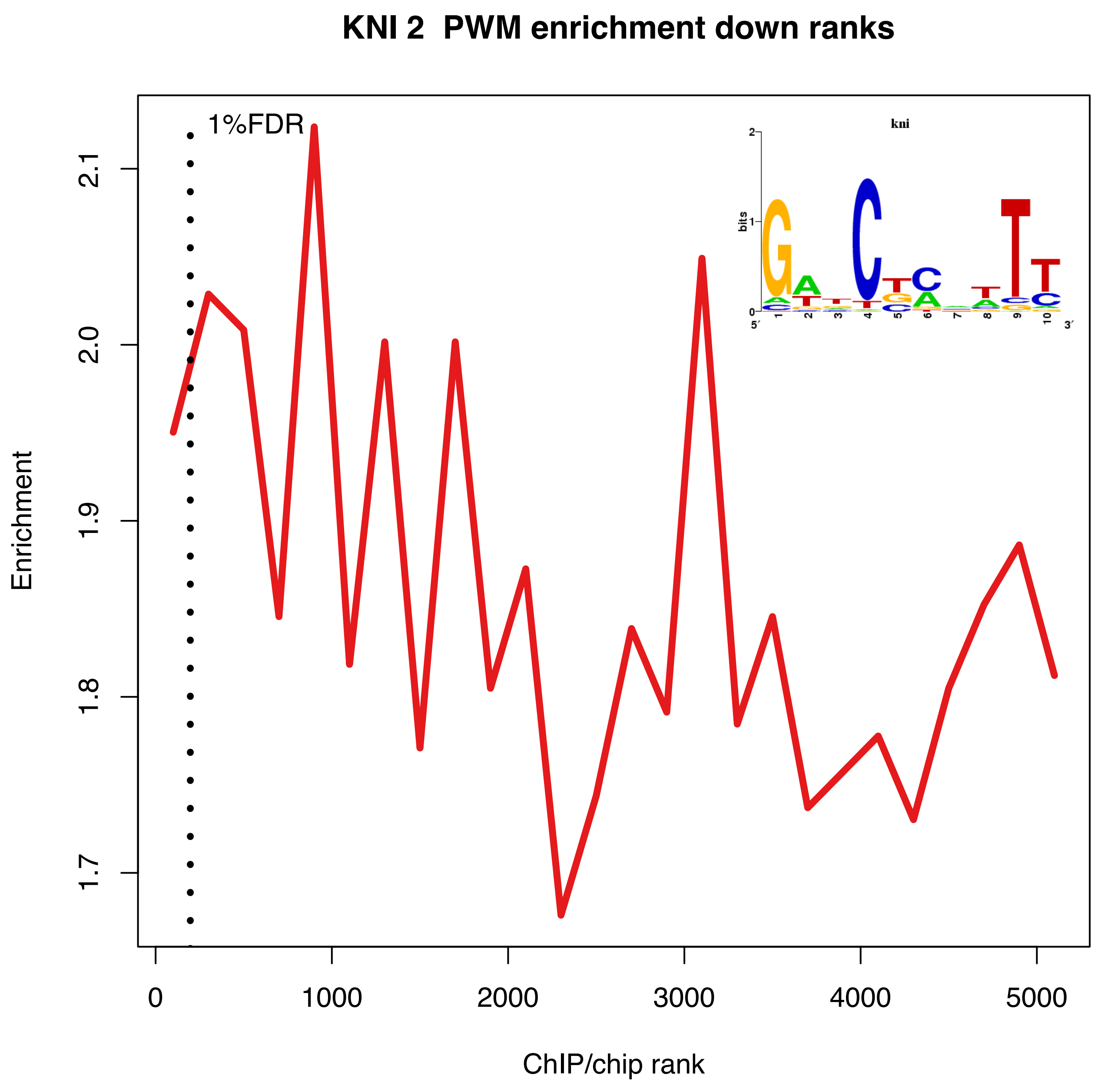
KNI 2 PWM enrichment down ranks
1%FDR
kni
bits
Enrichment
ChIP/chip rank
2.1
2.0
1.9
1.8
1.7
0
1000
2000
3000
4000
5000

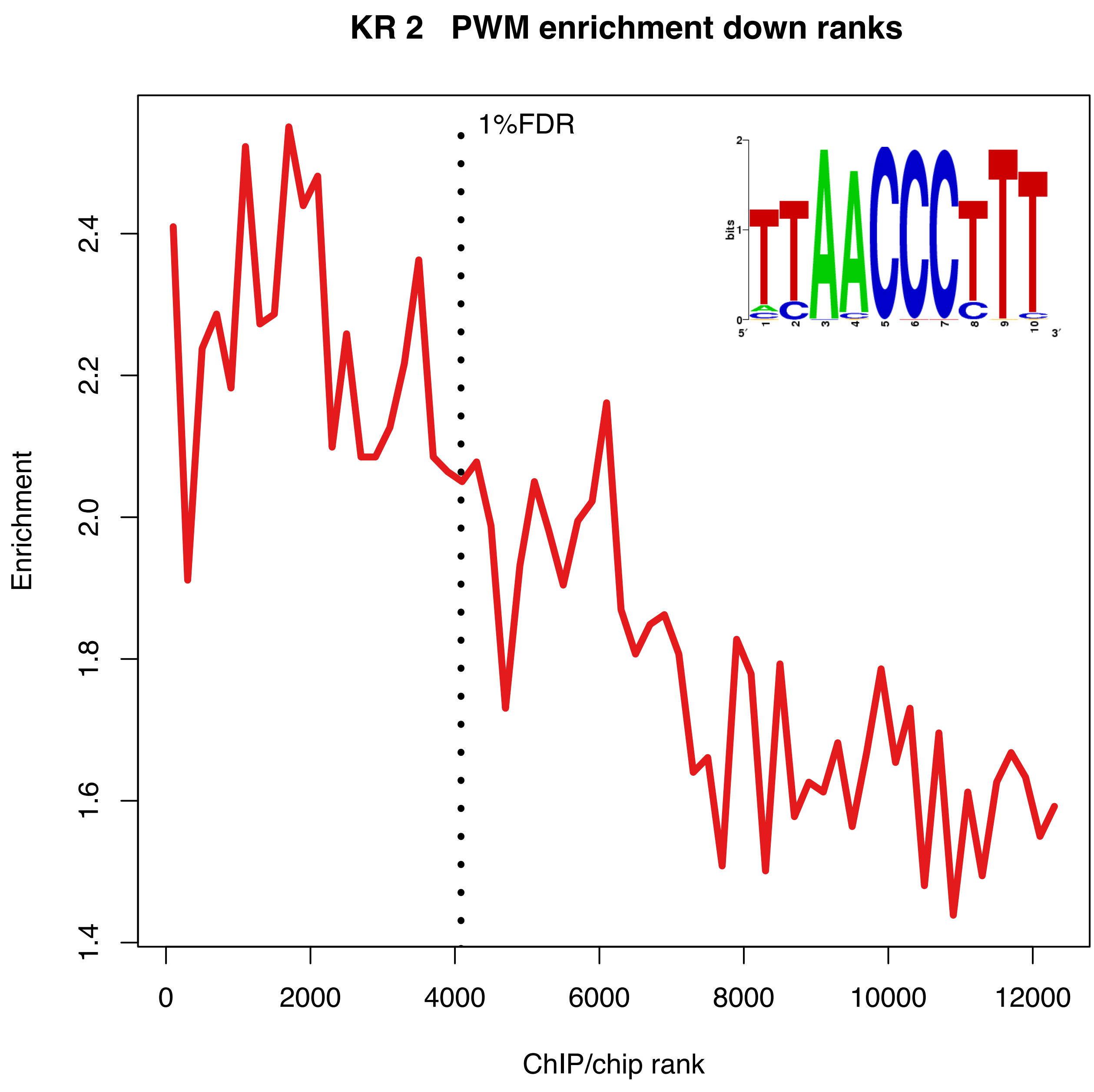
KR 2   PWM enrichment down ranks
1%FDR
Enrichment
ChIP/chip rank
1.4
1.6
1.8
2.0
2.2
2.4
0
2000
4000
6000
8000
10000
12000
bits
0
1
2
5′
3′

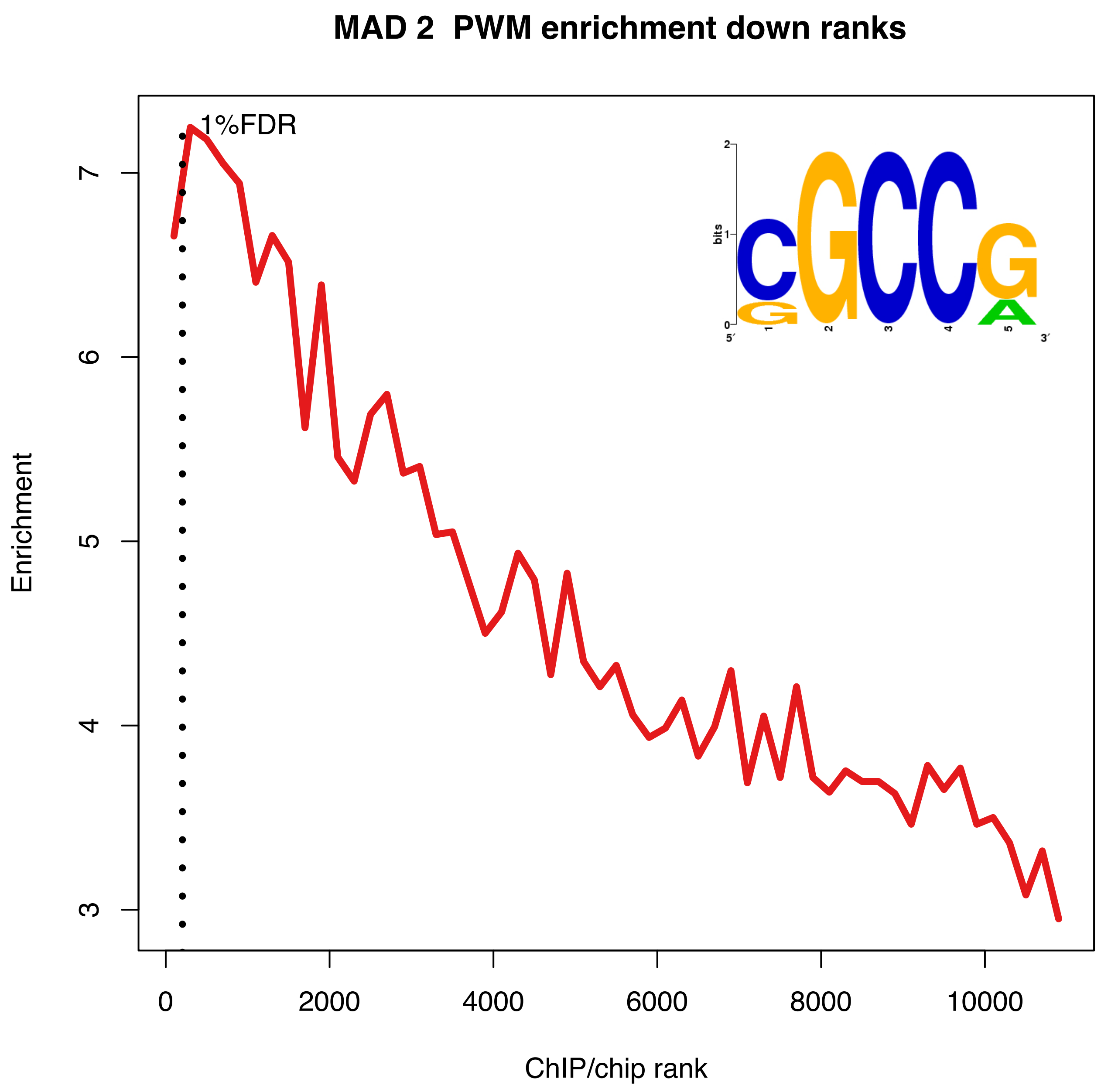
MAD 2 PWM enrichment down ranks
1%FDR
Enrichment
ChIP/chip rank
3
4
5
6
7
0
2000
4000
6000
8000
10000
bits
0
1
2
5′
3′
1
2
3
4
5

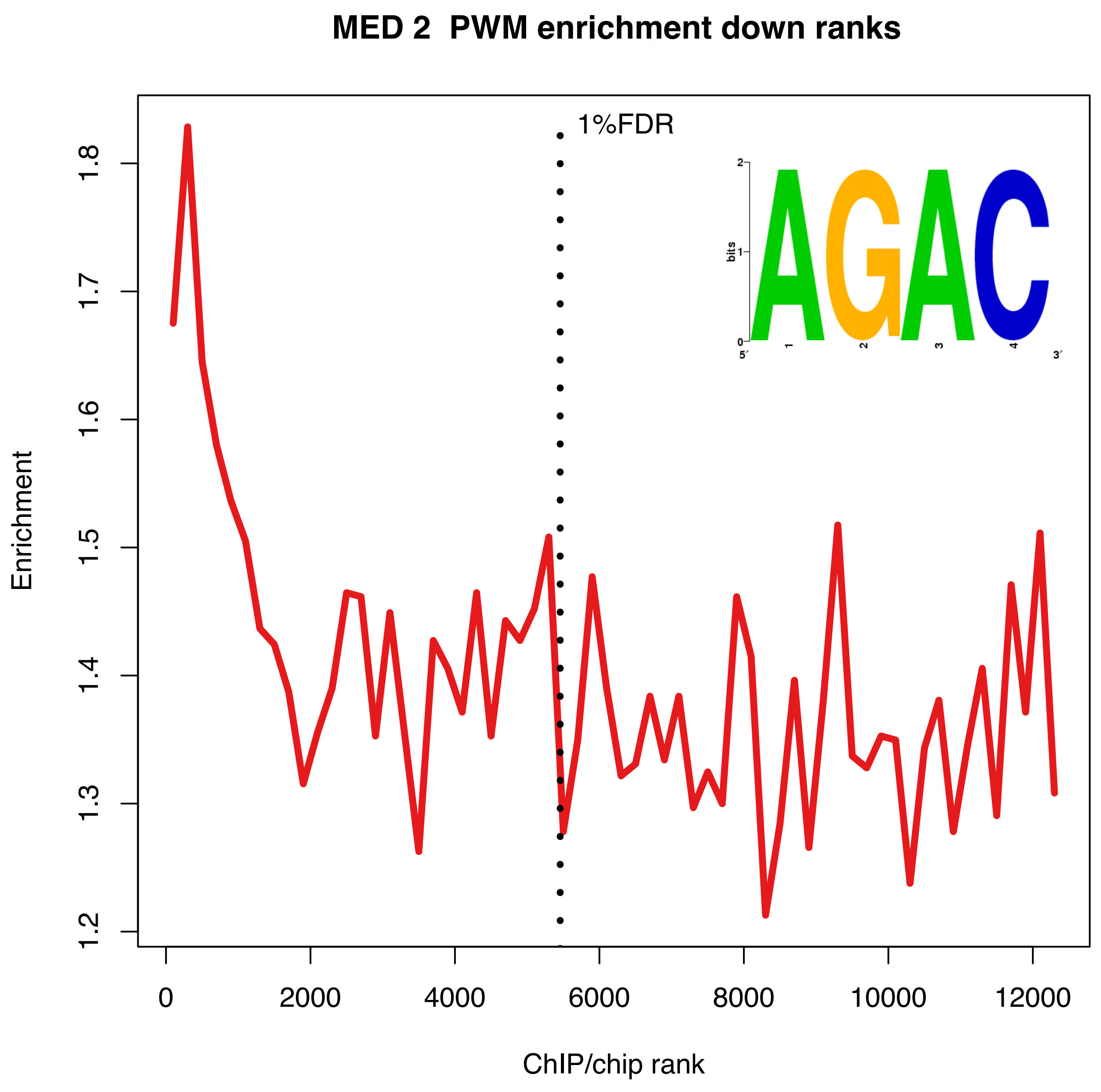
MED 2 PWM enrichment down ranks
1%FDR
Enrichment
1.2
1.3
1.4
1.5
1.6
1.7
1.8
ChIP/chip rank
0
2000
4000
6000
8000
10000
12000
AGAC
bits
0
1
2
5′
3′

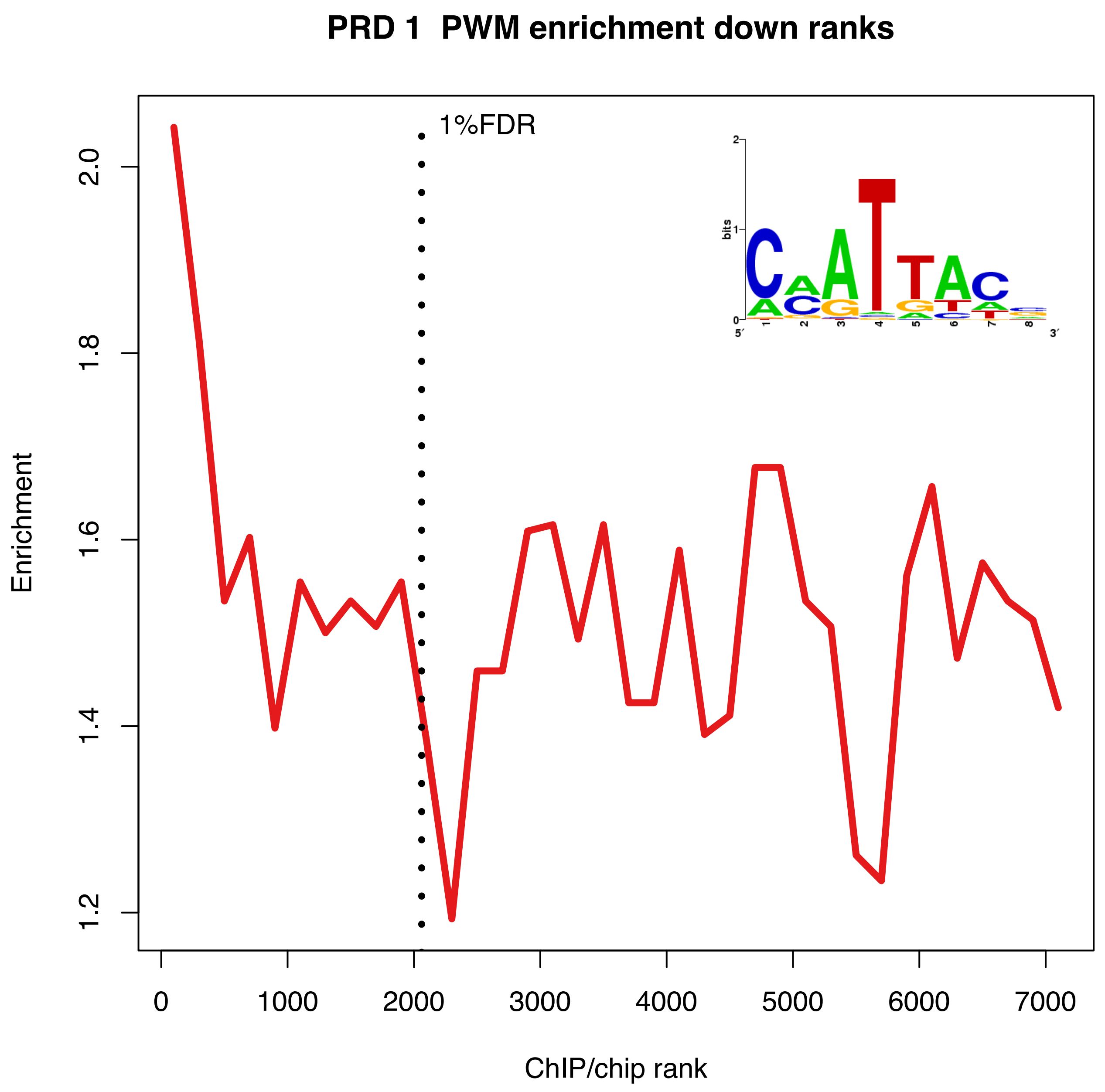
PRD 1 PWM enrichment down ranks
1%FDR
Enrichment
2.0
1.8
1.6
1.4
1.2
0
1000
2000
3000
4000
5000
6000
7000
ChIP/chip rank
bits
5′
3′

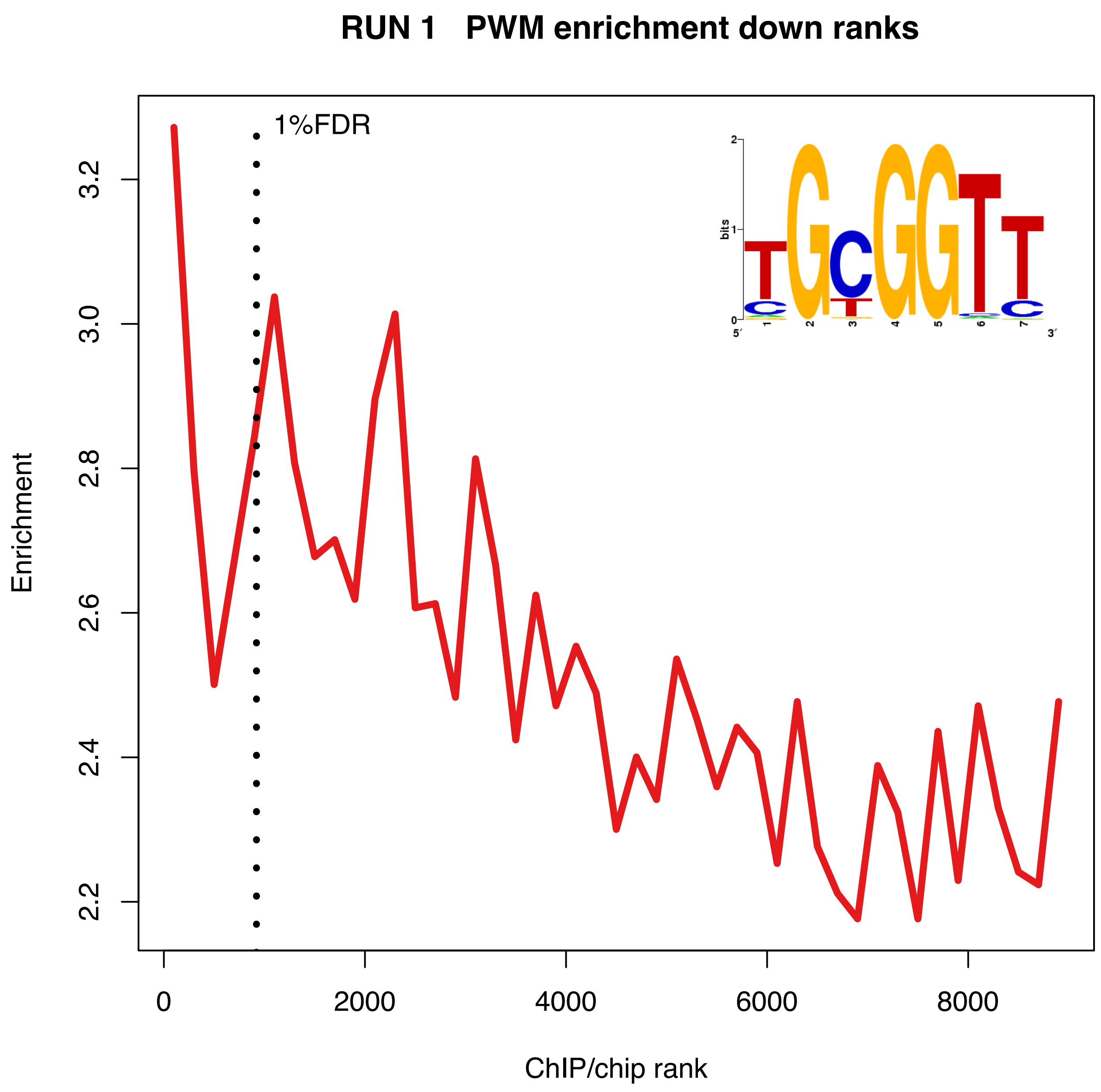
RUN 1 PWM enrichment down ranks
1%FDR
Enrichment
3.2
3.0
2.8
2.6
2.4
2.2
0
2000
4000
6000
8000
ChIP/chip rank
bits
5′
3′

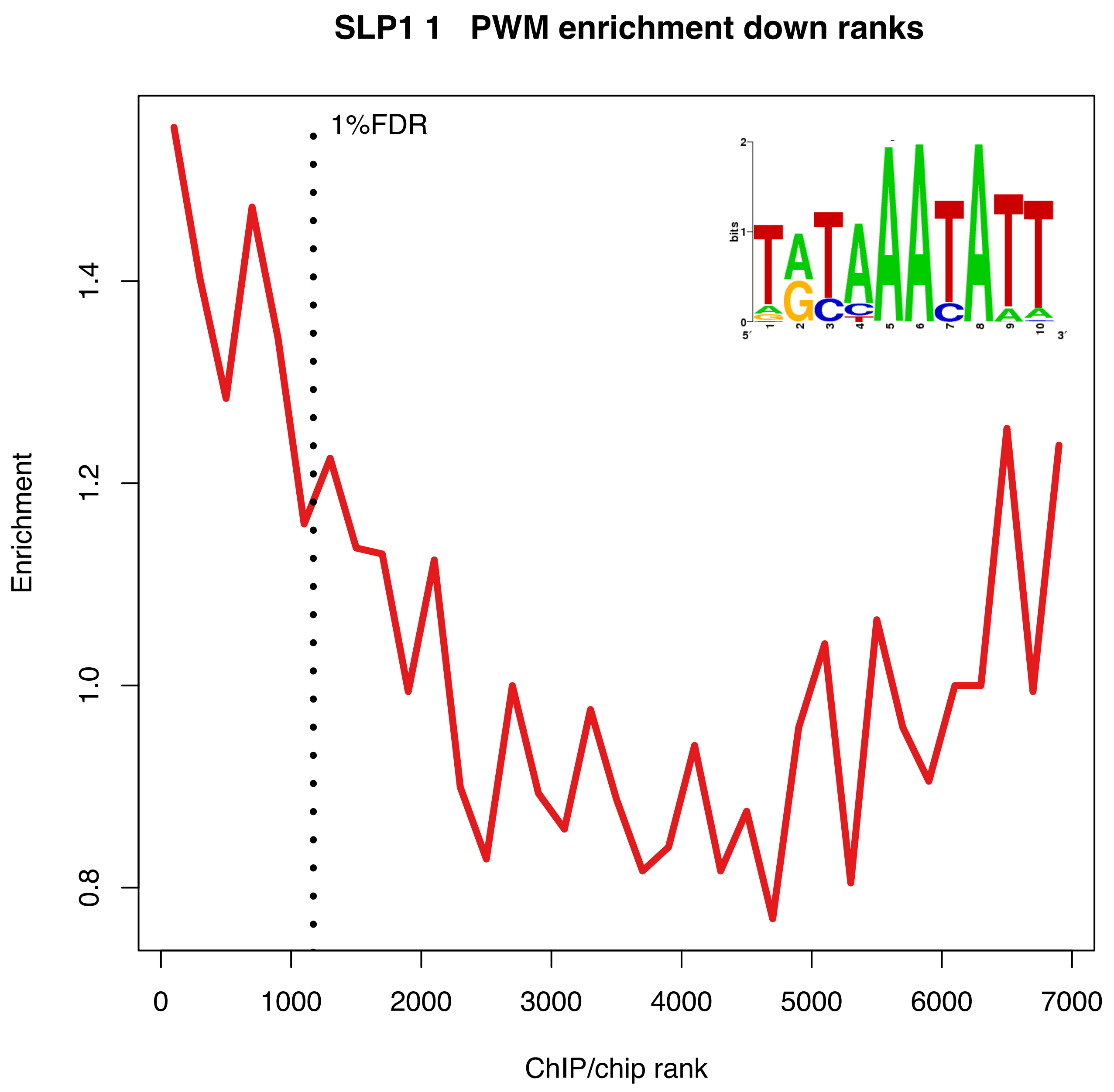
SLP1 1   PWM enrichment down ranks
1%FDR
Enrichment
ChIP/chip rank
0.8
1.0
1.2
1.4
0
1000
2000
3000
4000
5000
6000
7000
bits

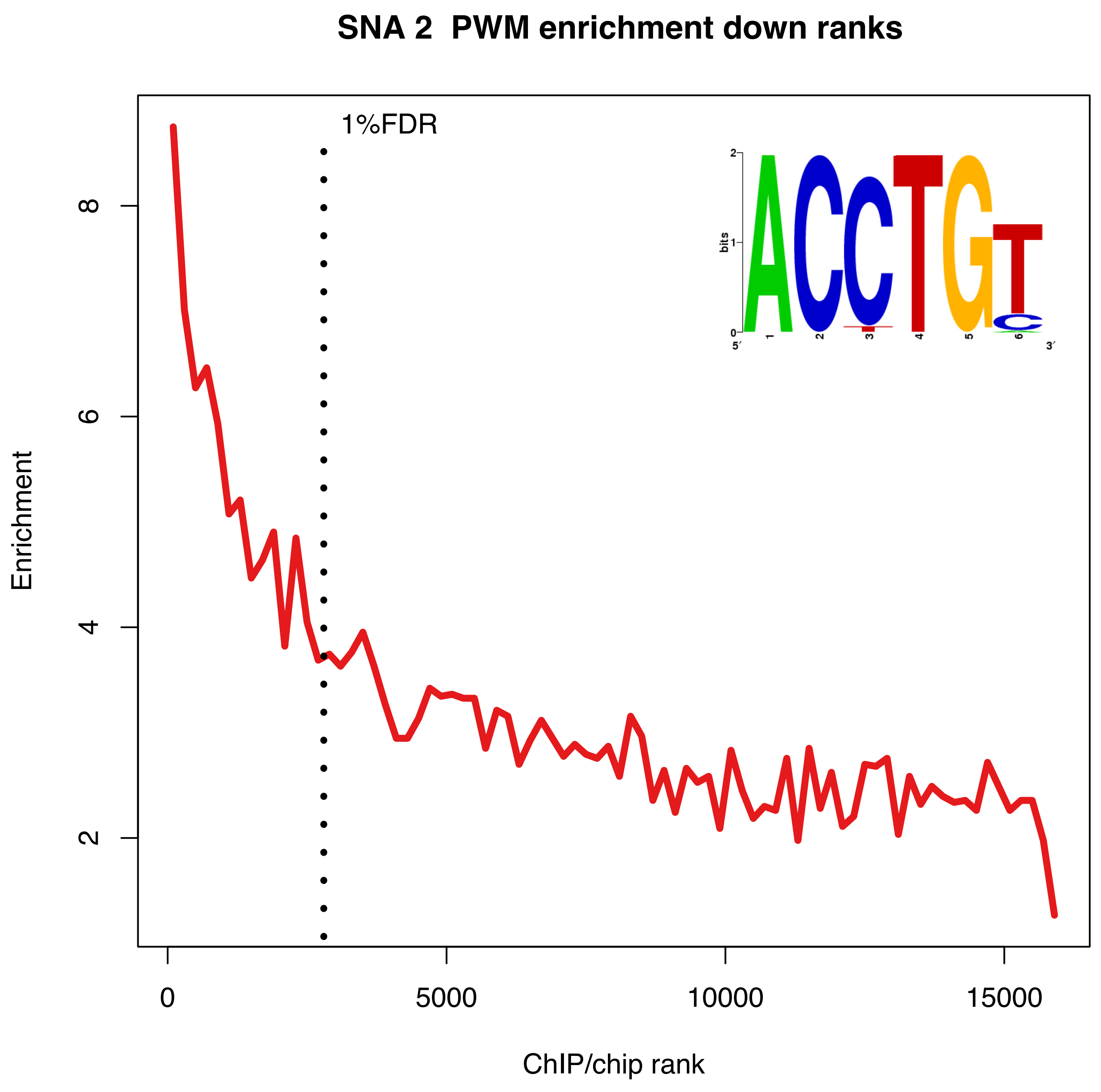
SNA 2 PWM enrichment down ranks
1%FDR
Enrichment
8
6
4
2
0
5000
10000
15000
ChIP/chip rank
bits
5′
3′

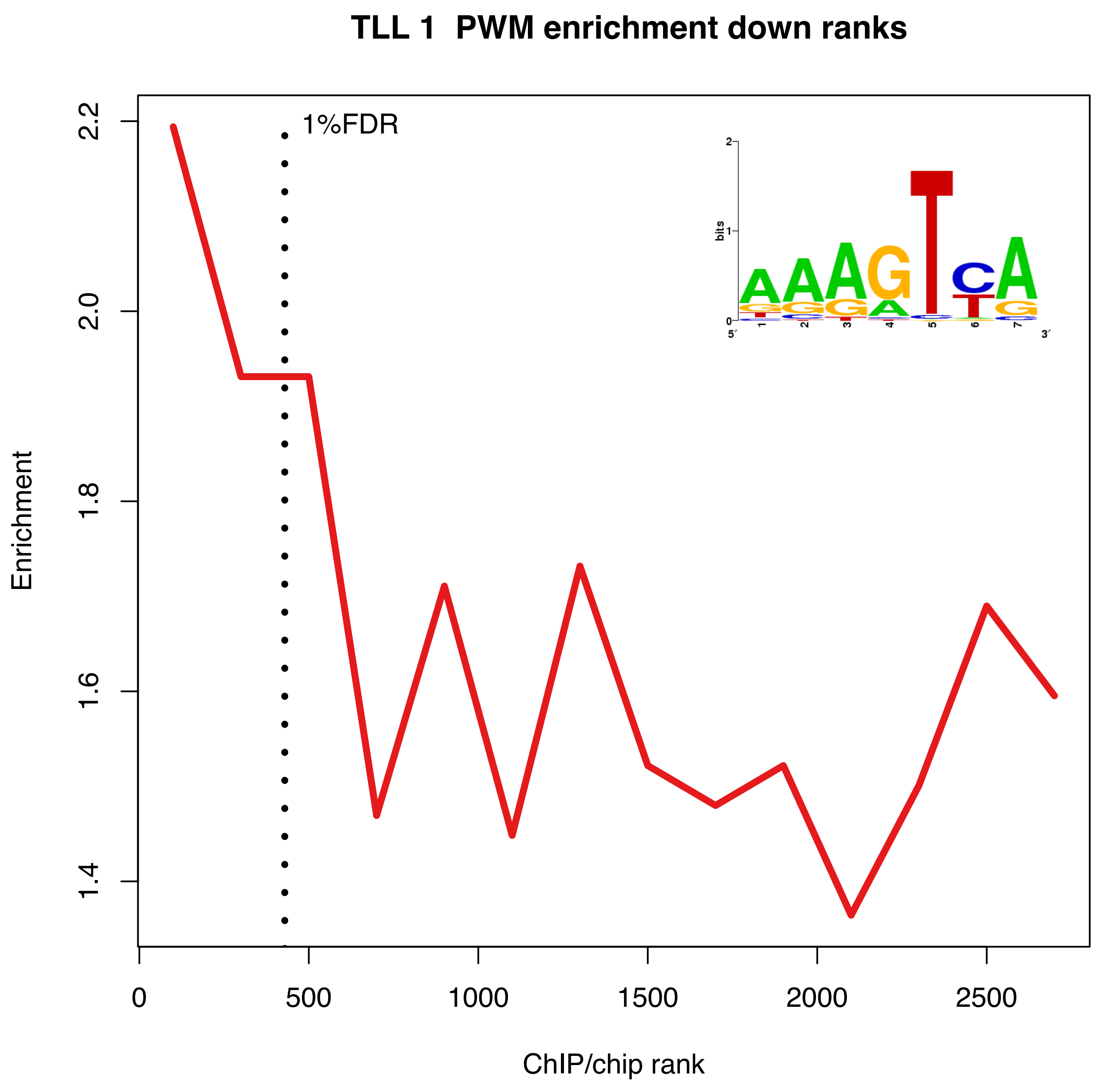
TLL 1 PWM enrichment down ranks
1%FDR
Enrichment
2.2
2.0
1.8
1.6
1.4
0
500
1000
1500
2000
2500
ChIP/chip rank
bits
AAAGTCA

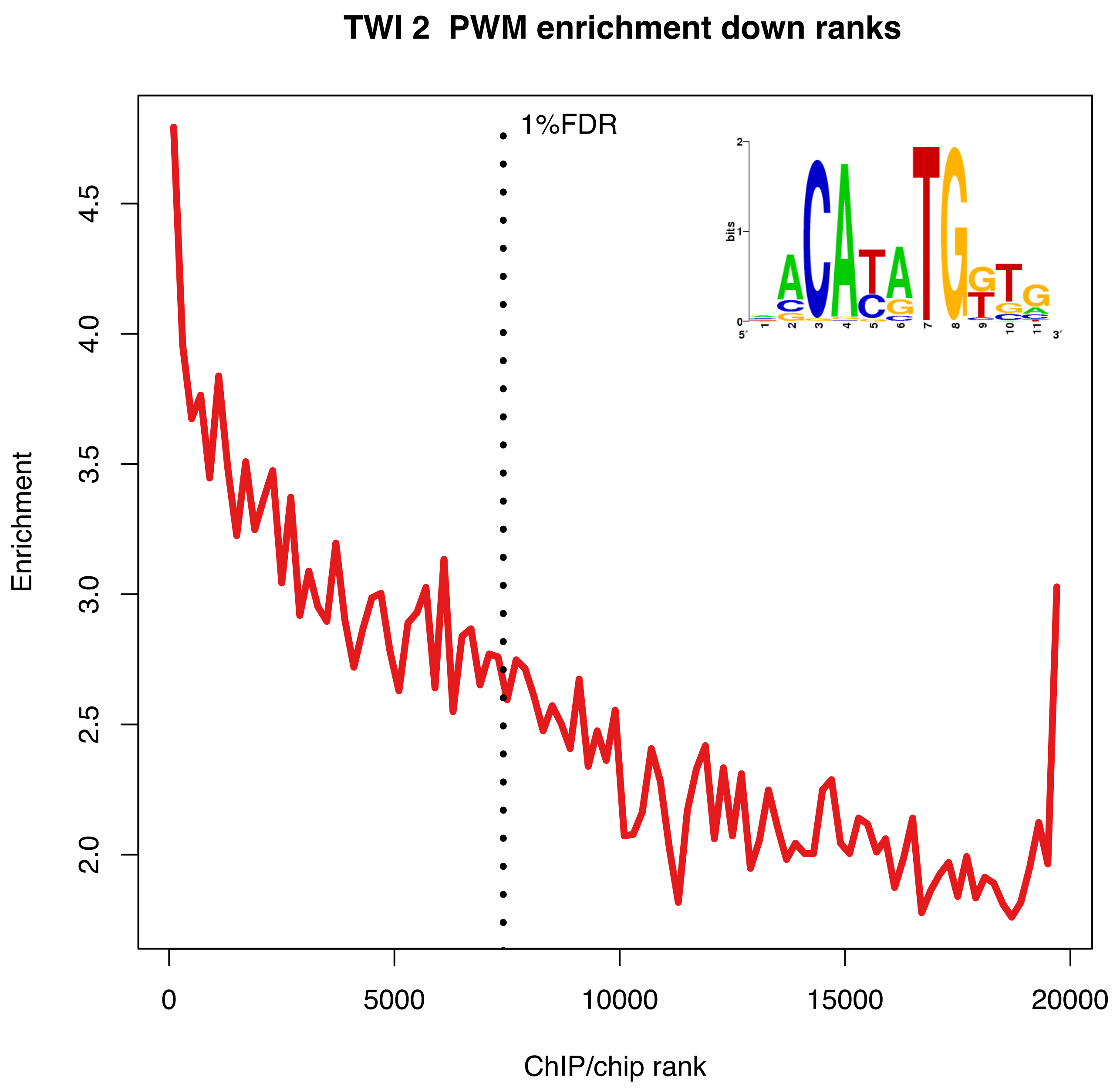
TWI 2 PWM enrichment down ranks
1%FDR
Enrichment
ChIP/chip rank
4.5
4.0
3.5
3.0
2.5
2.0
0
5000
10000
15000
20000
bits
