# Supplementary material for: Developmental roles of 21 Drosophila transcription factors are determined by quantitative differences in binding to an overlapping set of thousands of genomic regions
Source: Genome Biol. 2009 Jul 23;10(7):R80. doi: 10.1186/gb-2009-10-7-r80 (PMC2728534; doi:10.1186/gb-2009-10-7-r80)
Supplement: Additional data file 5 — Relative levels of mean UV crosslinking and mean ChIP/chip scores across a series of highly and poorly bound genomic regions. [file gb-2009-10-7-r80-S5.pdf]

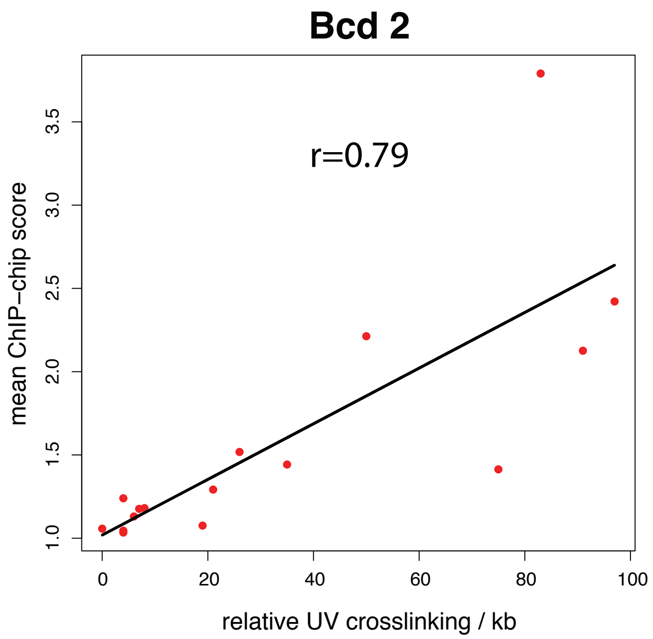

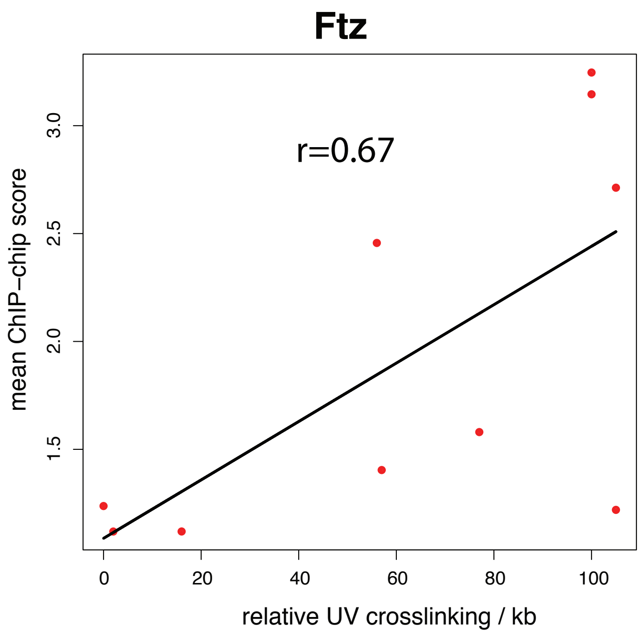

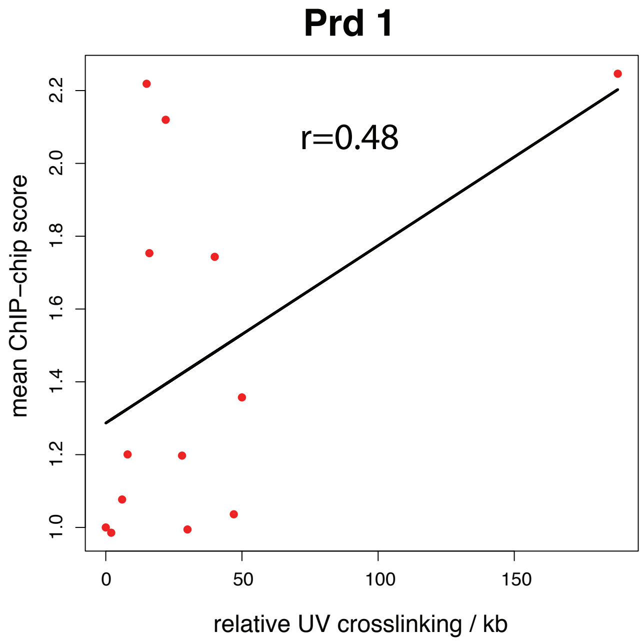

Addtional data file 5. Scatter plots comparing formaldehyde ChIP/chip and southern blot based UV crosslinking data. Relative levels of UV crosslinking per kb were measured to a series of genomic restriction fragments (*x*-axis, Carr and Biggin, 1999 [31]). The mean Chip/chip score across these same genomic regions was calculated (*y*-axis, this paper). The pearson correlation coefficients (r) are given.
