# Supplementary figures and images for: Developmental roles of 21 Drosophila transcription factors are determined by quantitative differences in binding to an overlapping set of thousands of genomic regions
Source: Genome Biol. 2009 Jul 23;10(7):R80. doi: 10.1186/gb-2009-10-7-r80 (PMC2728534; doi:10.1186/gb-2009-10-7-r80)

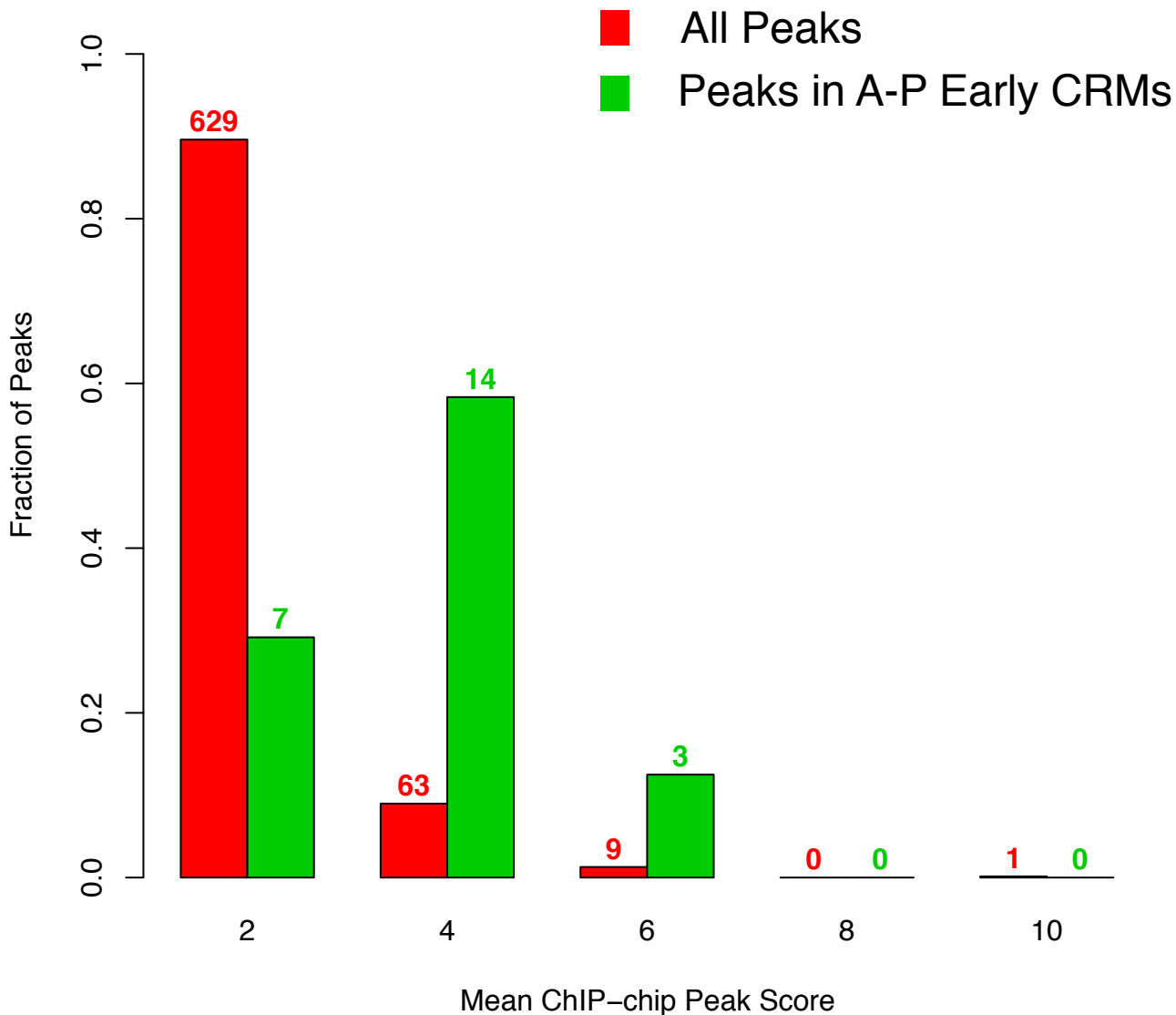

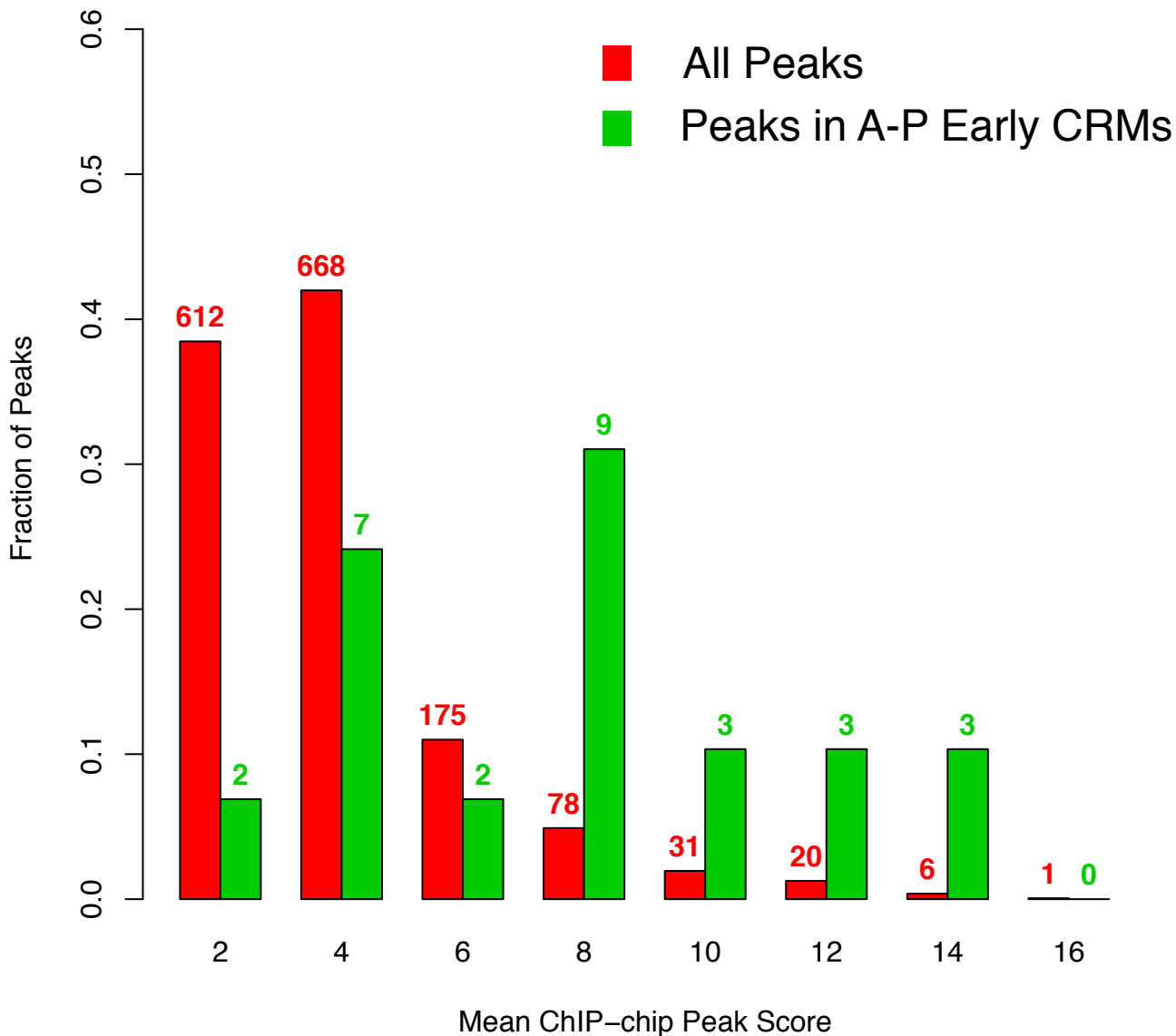

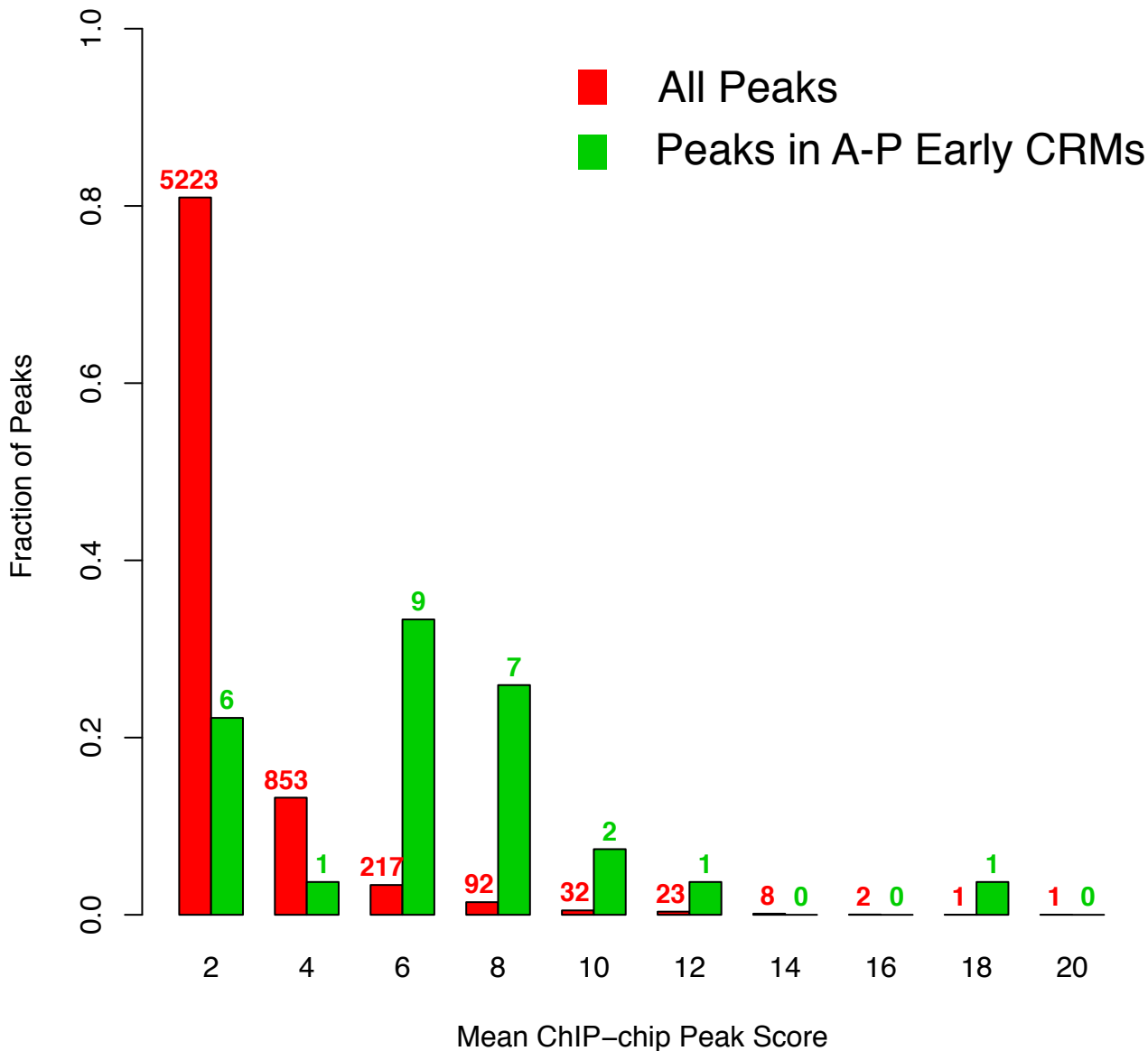

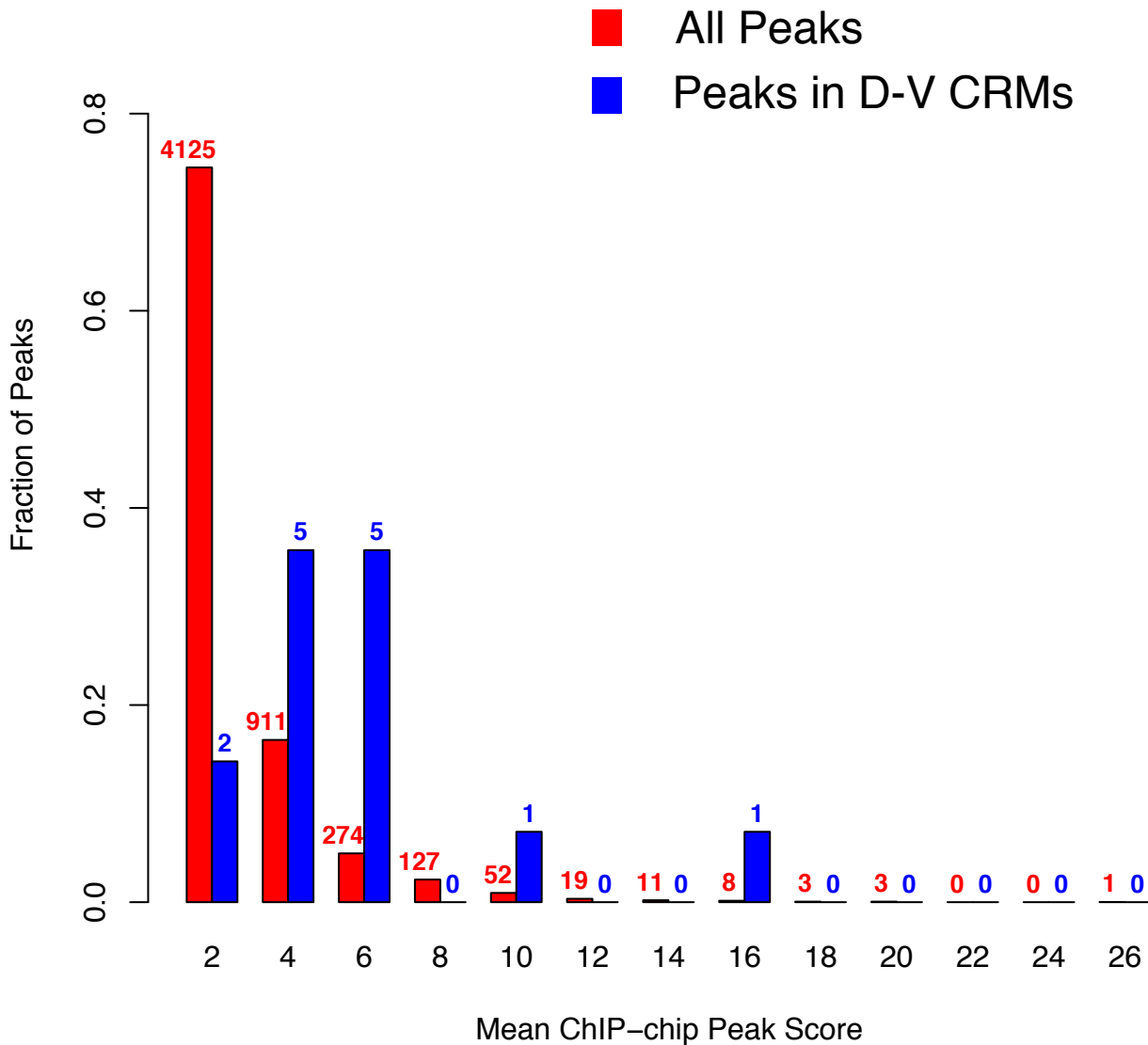

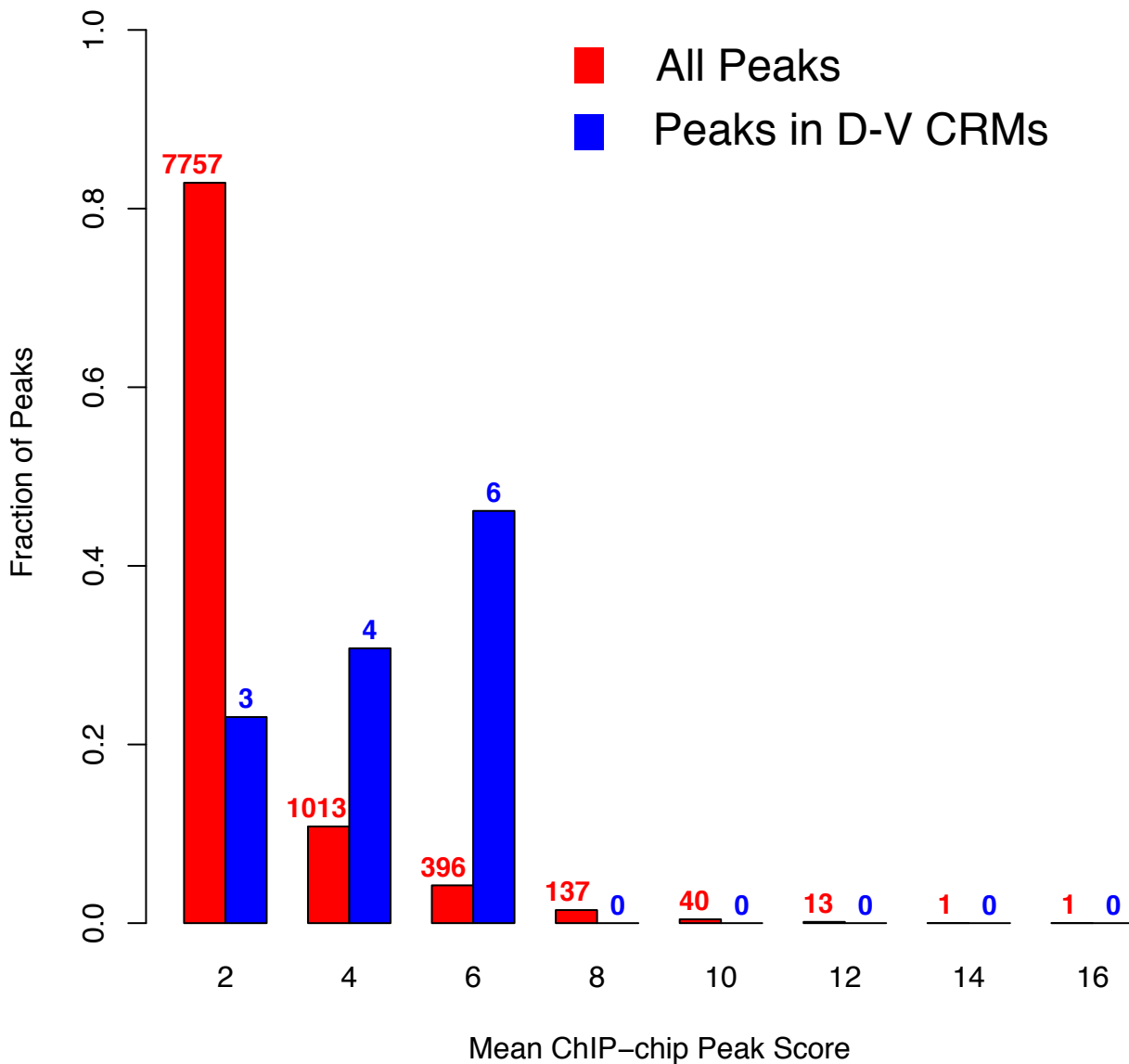

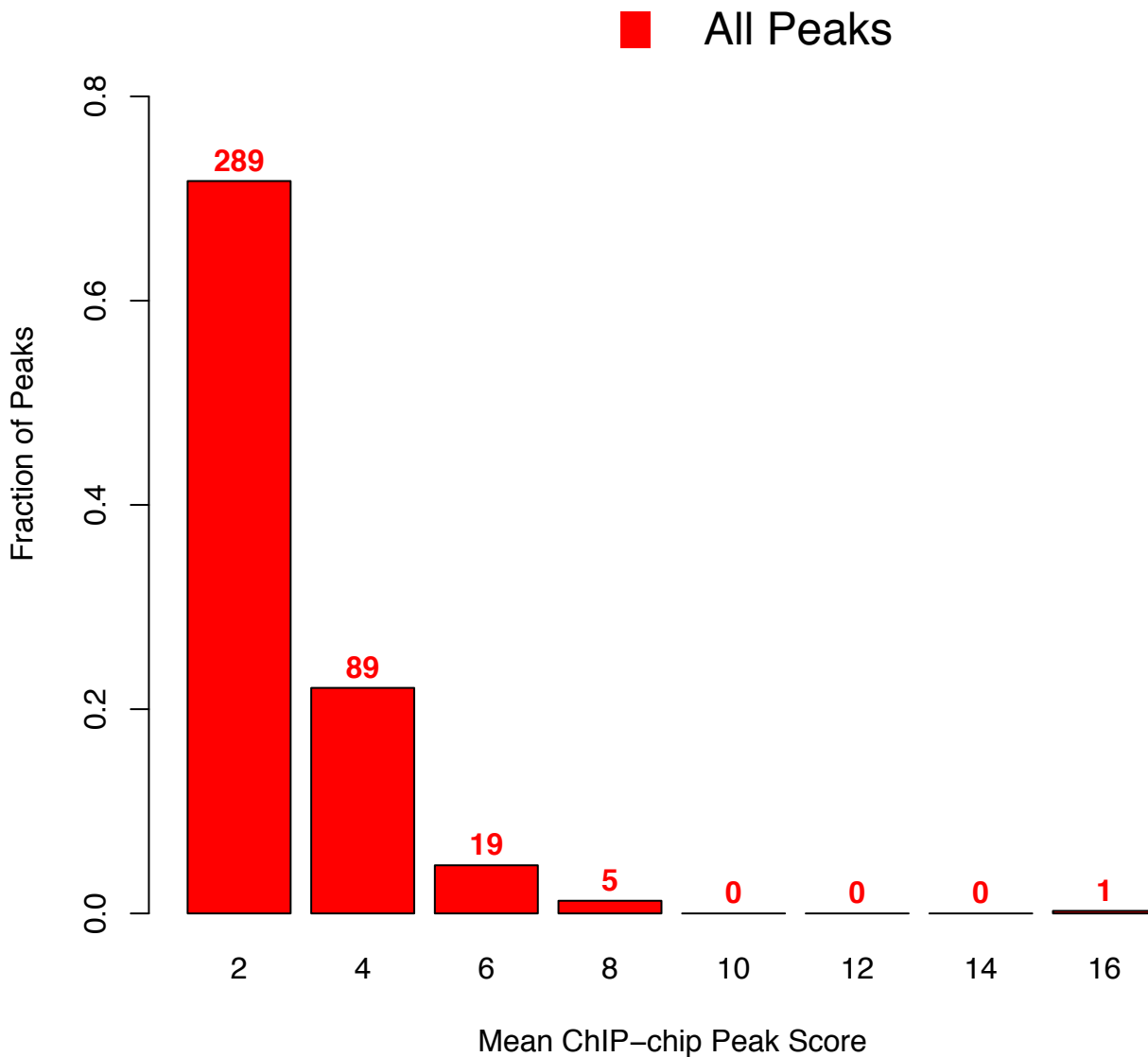

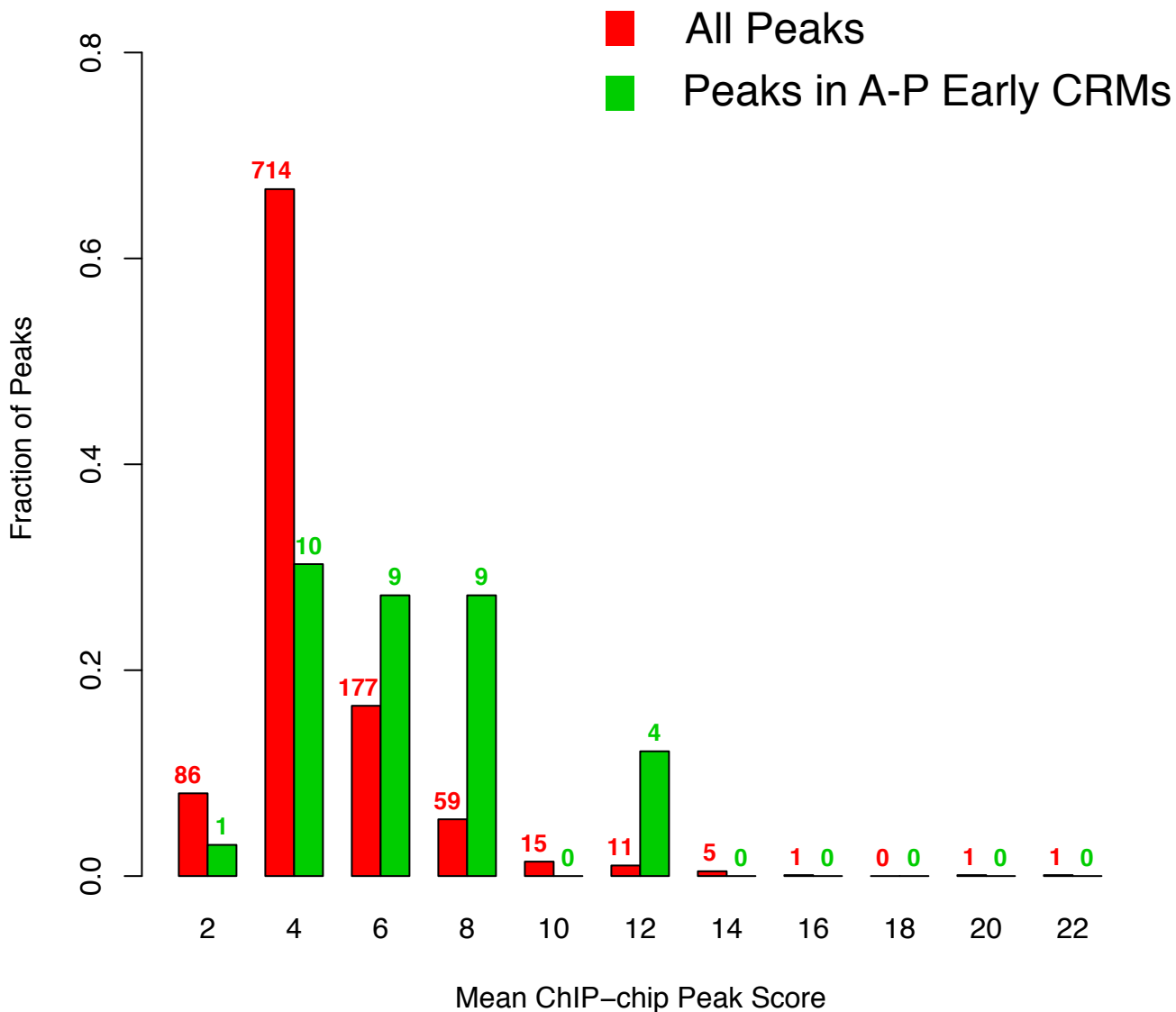

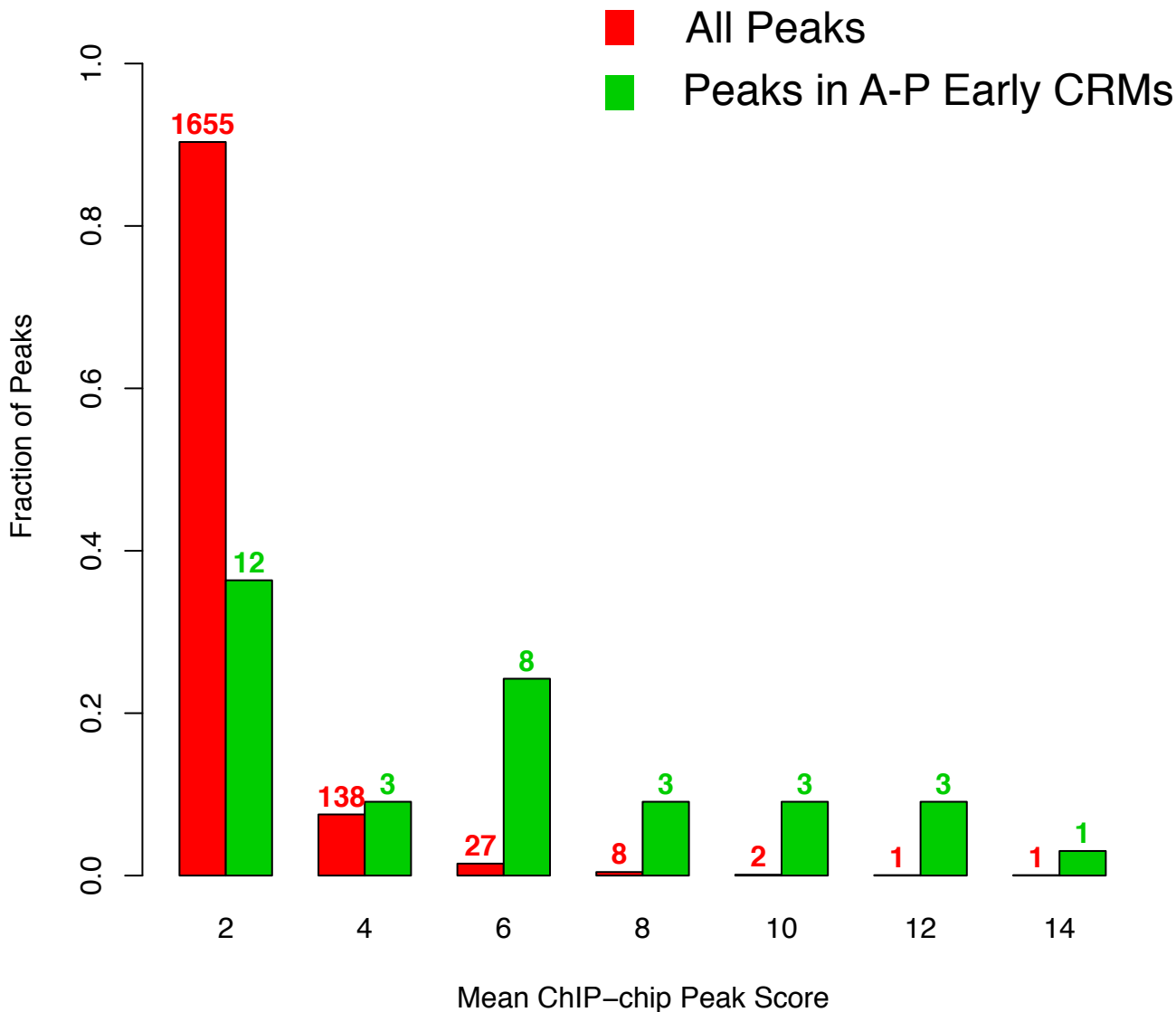

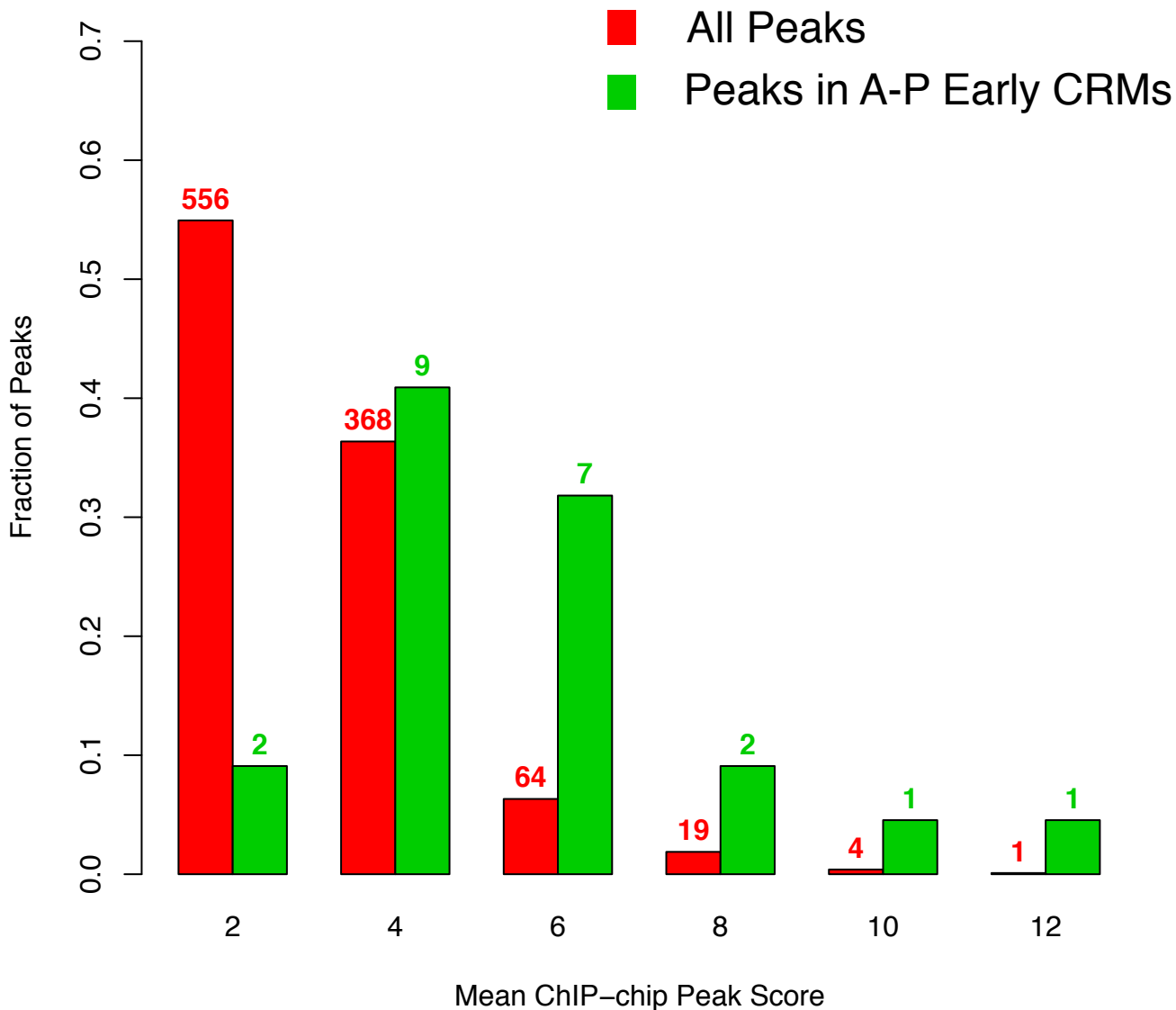

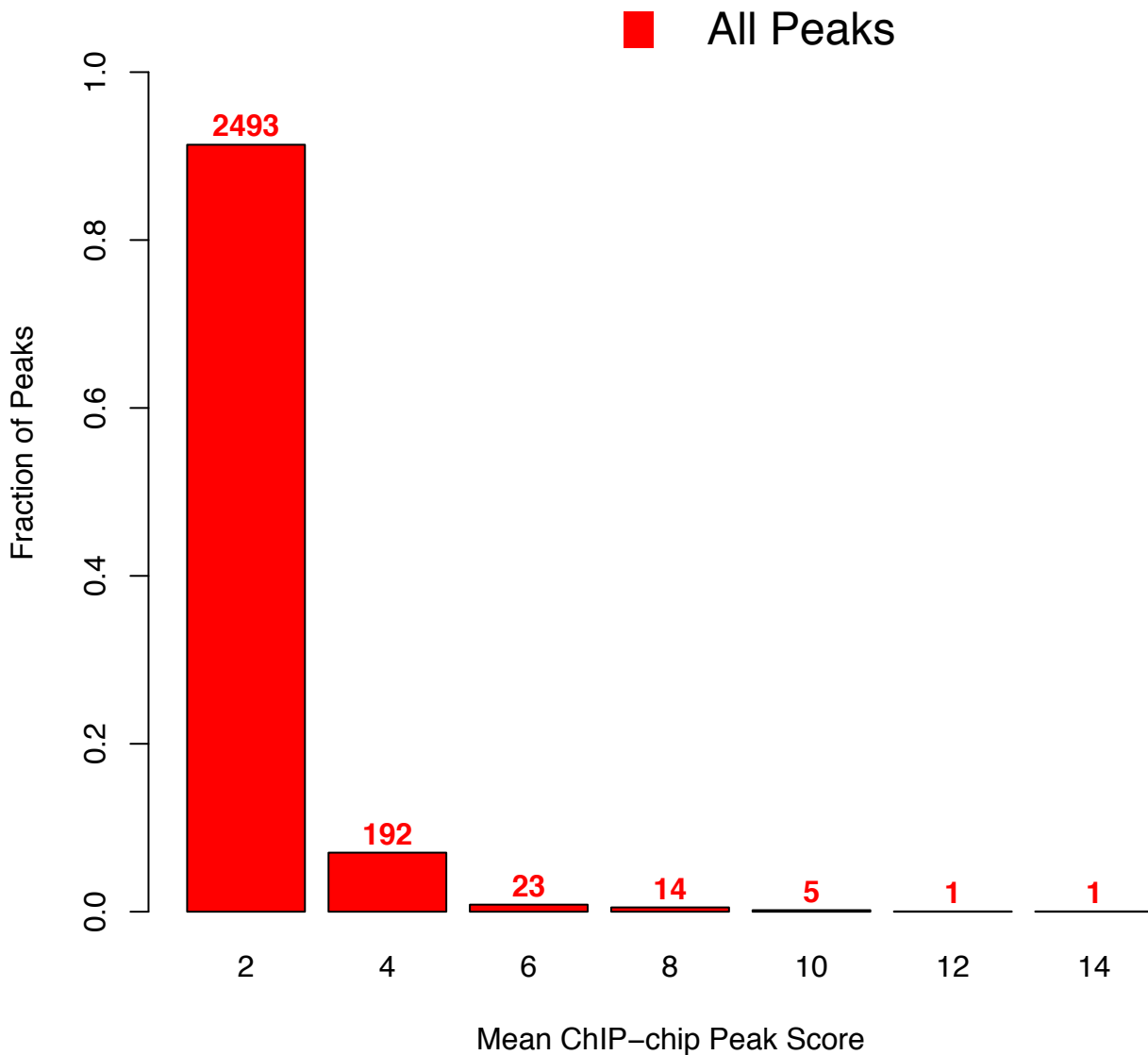

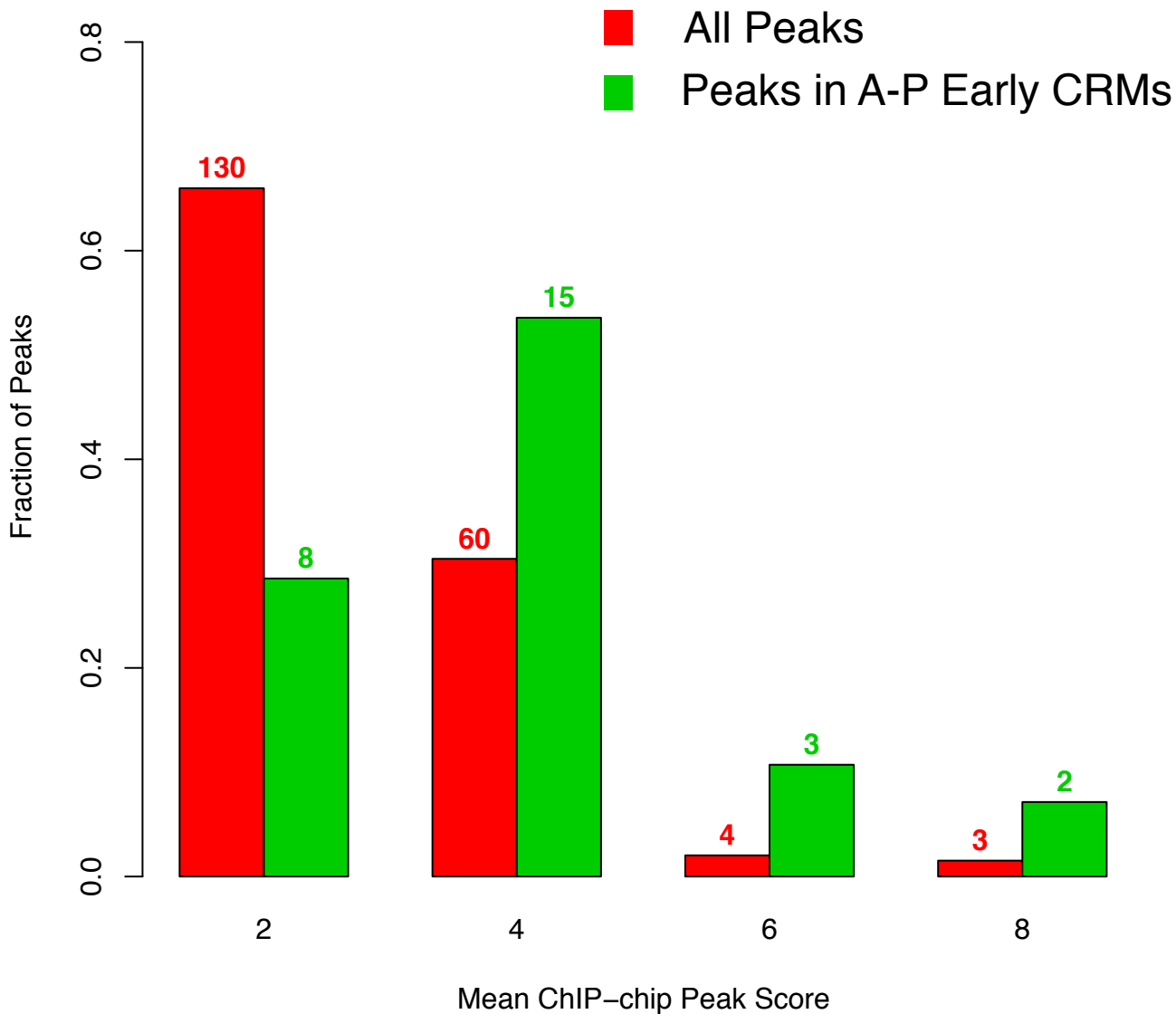

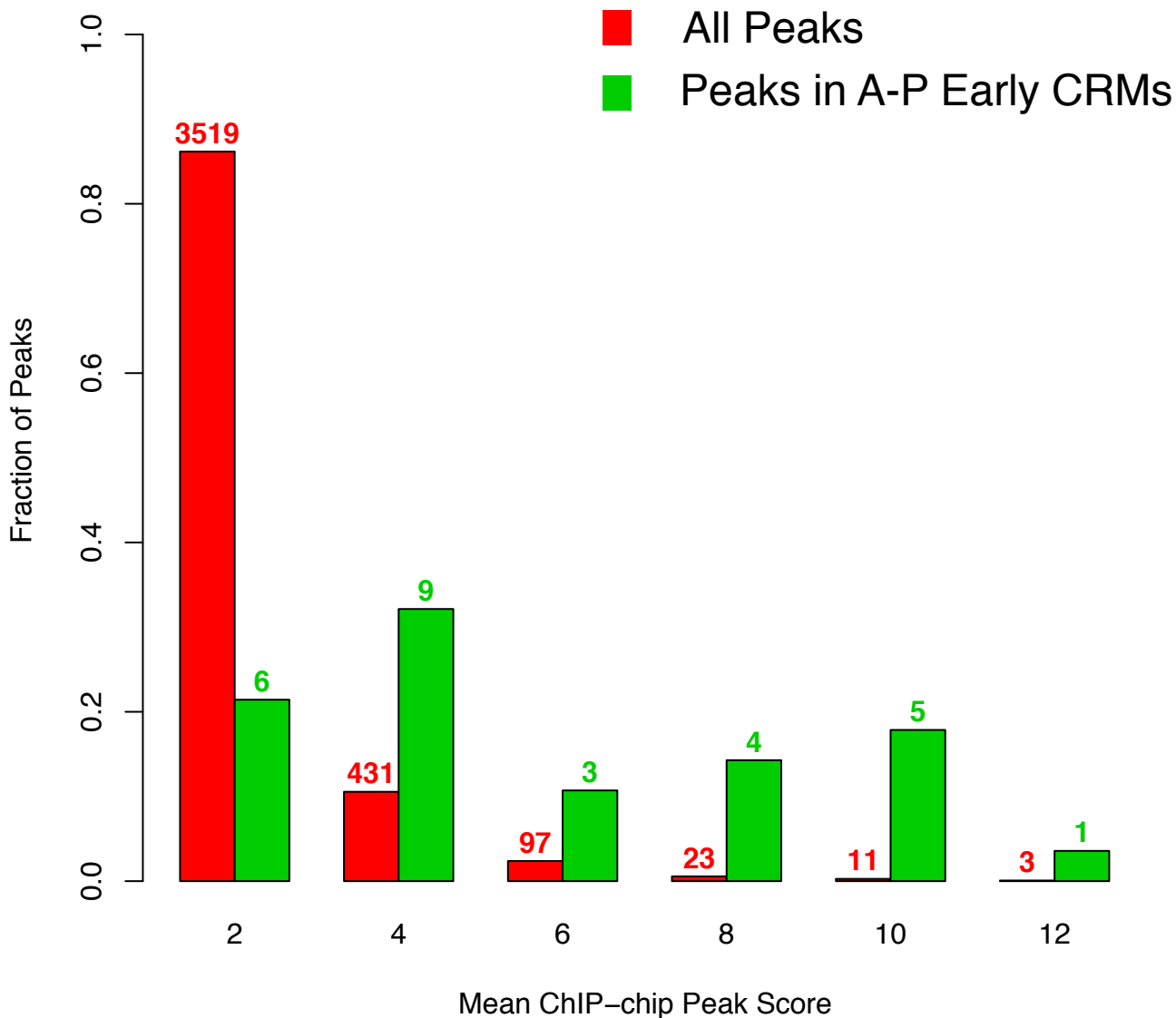

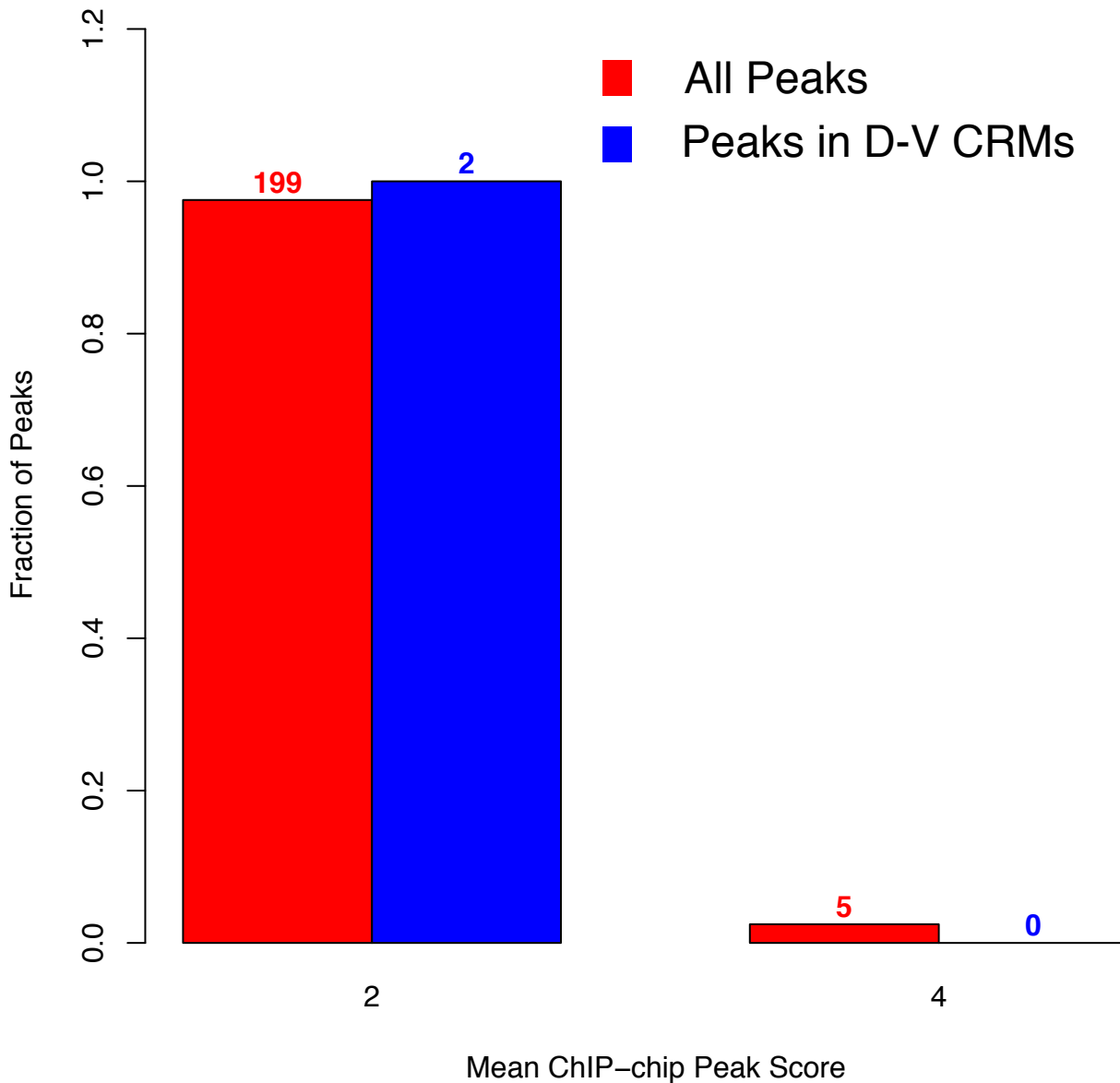

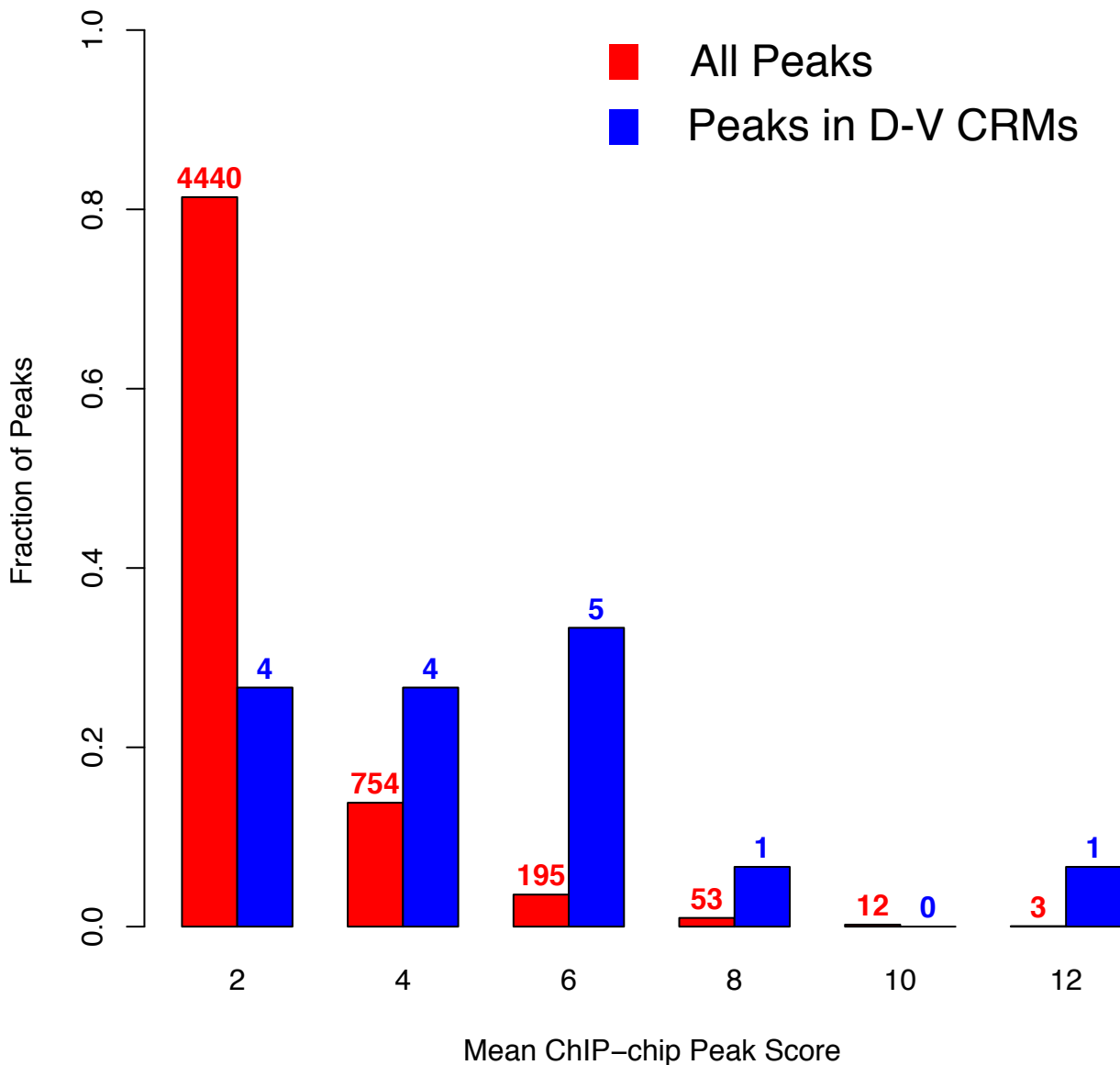

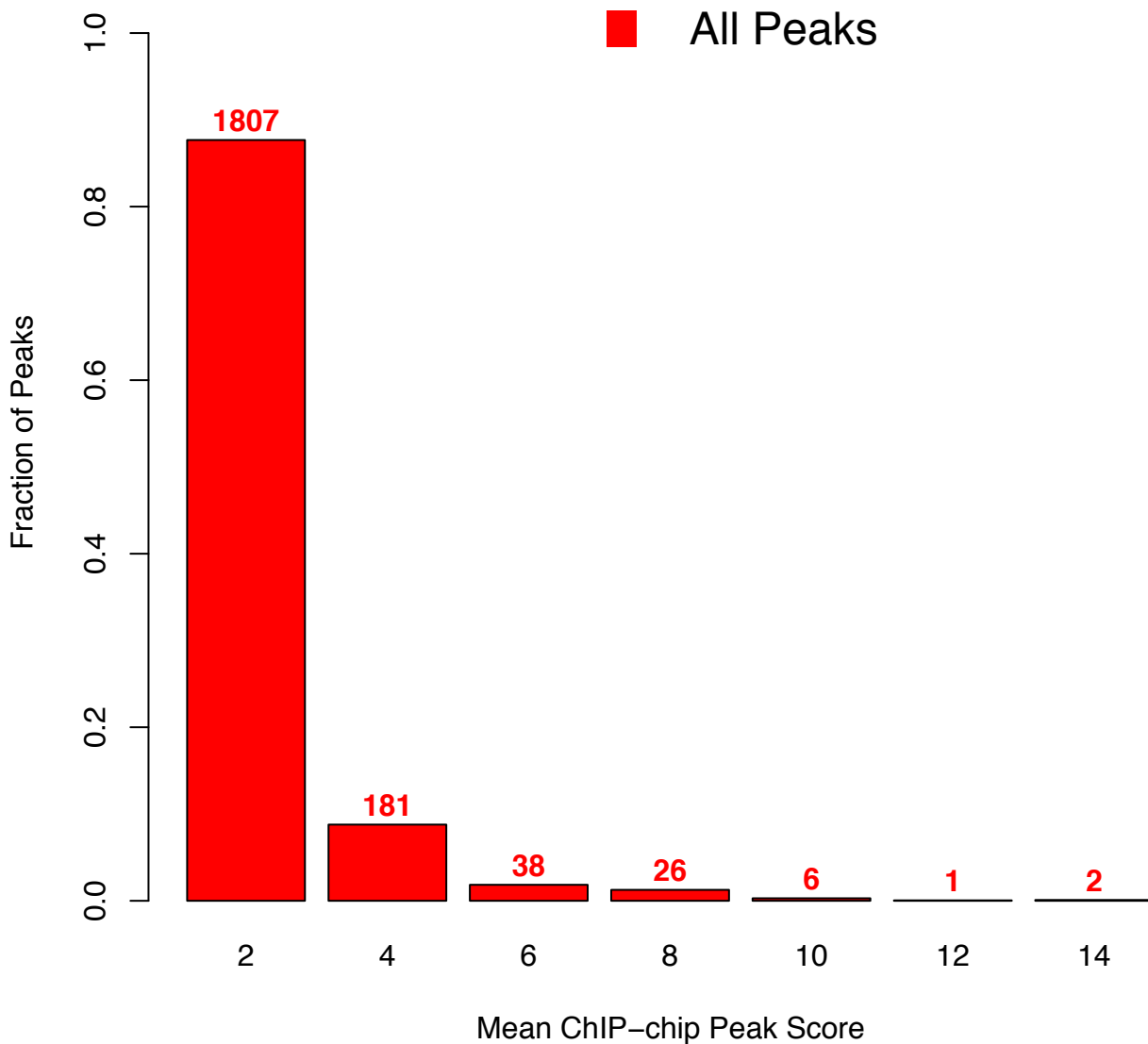

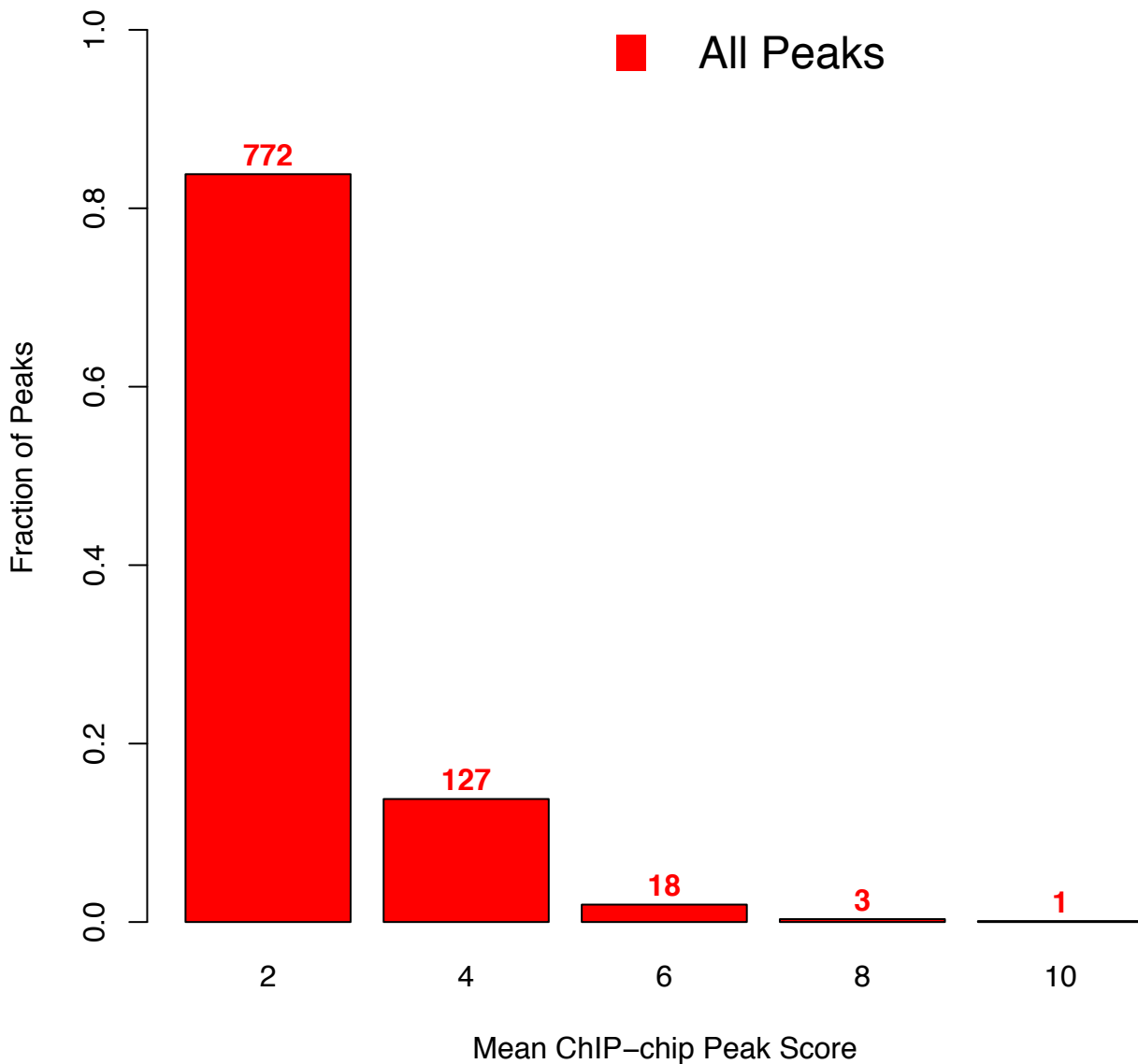

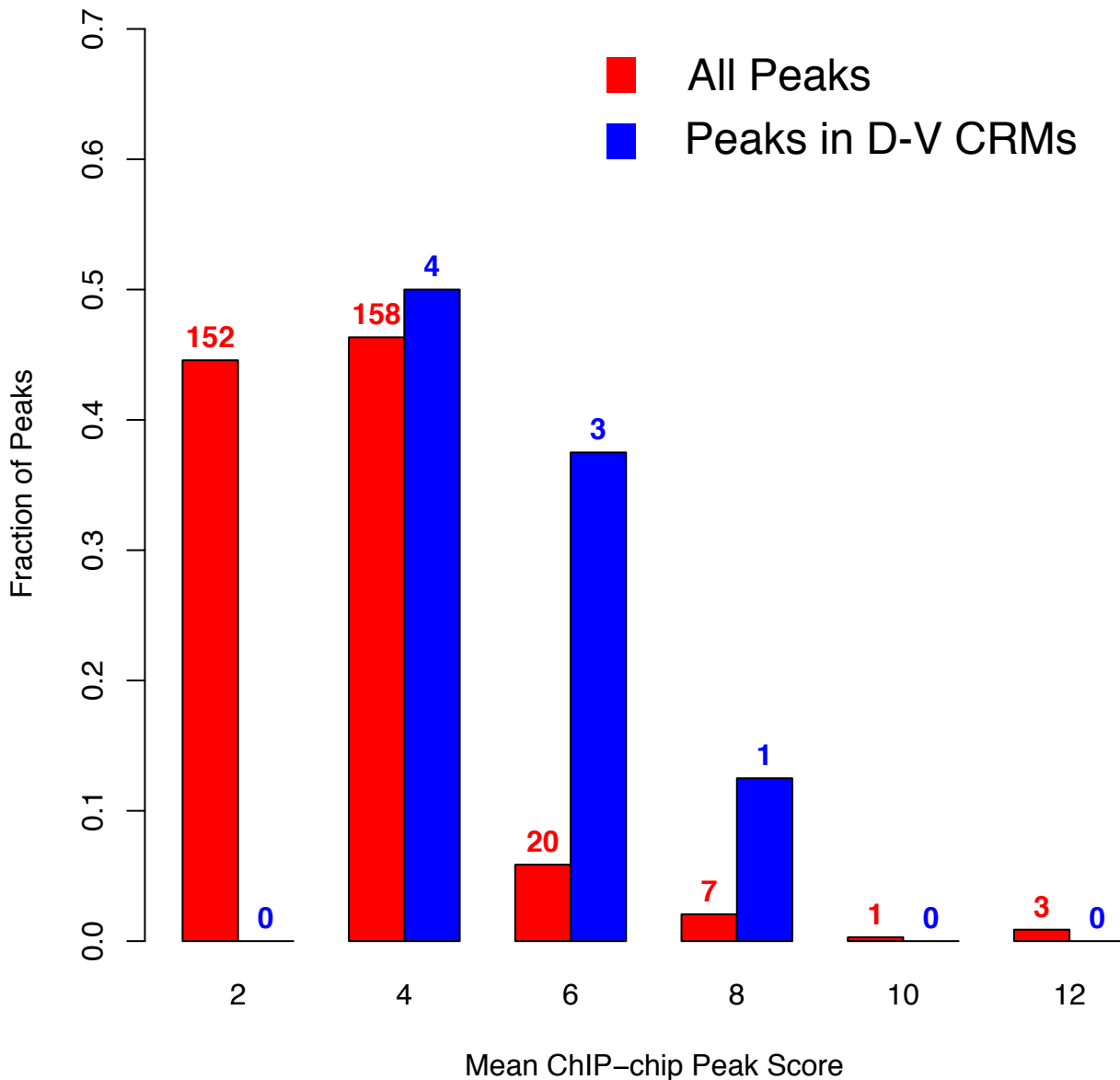

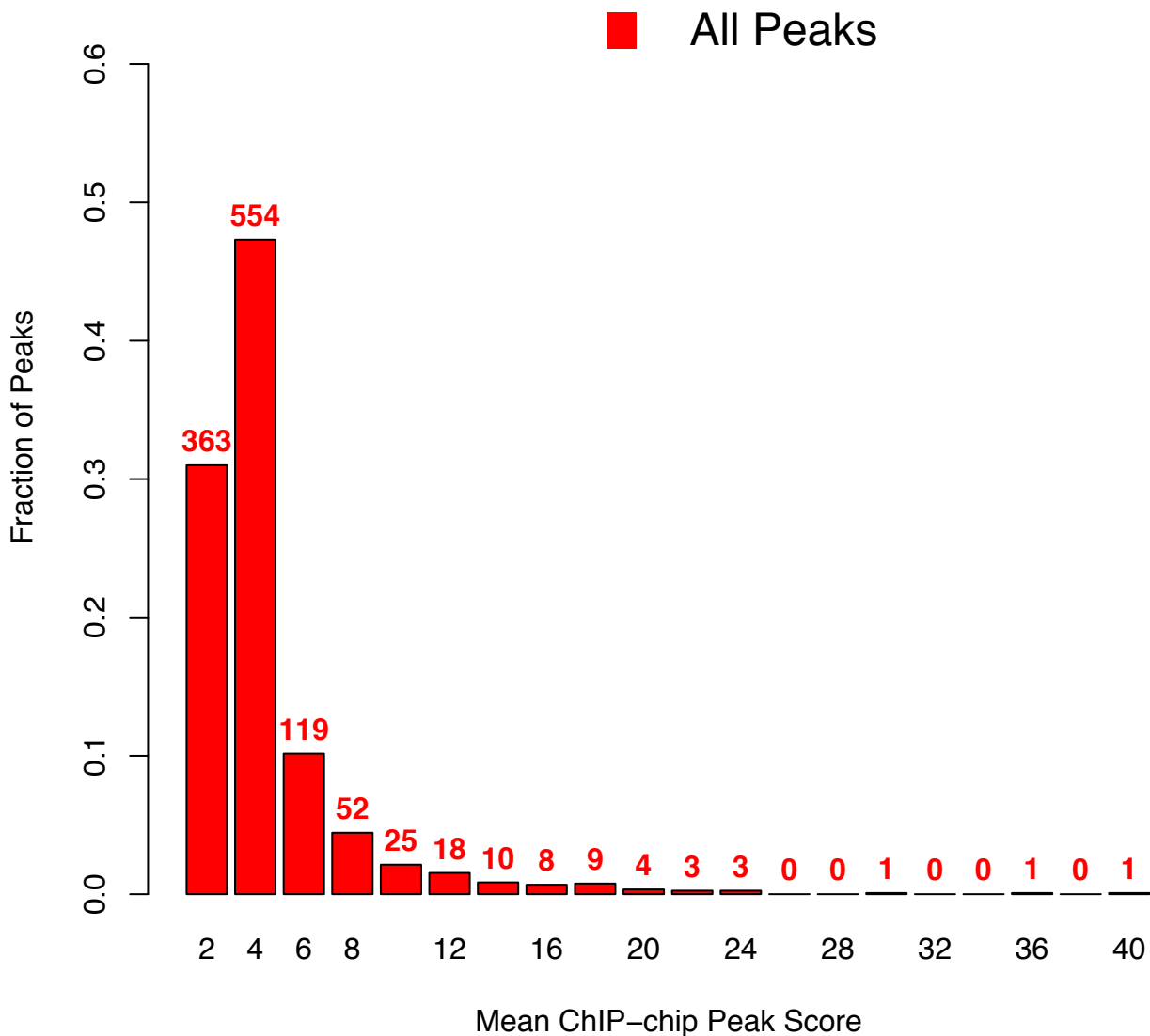

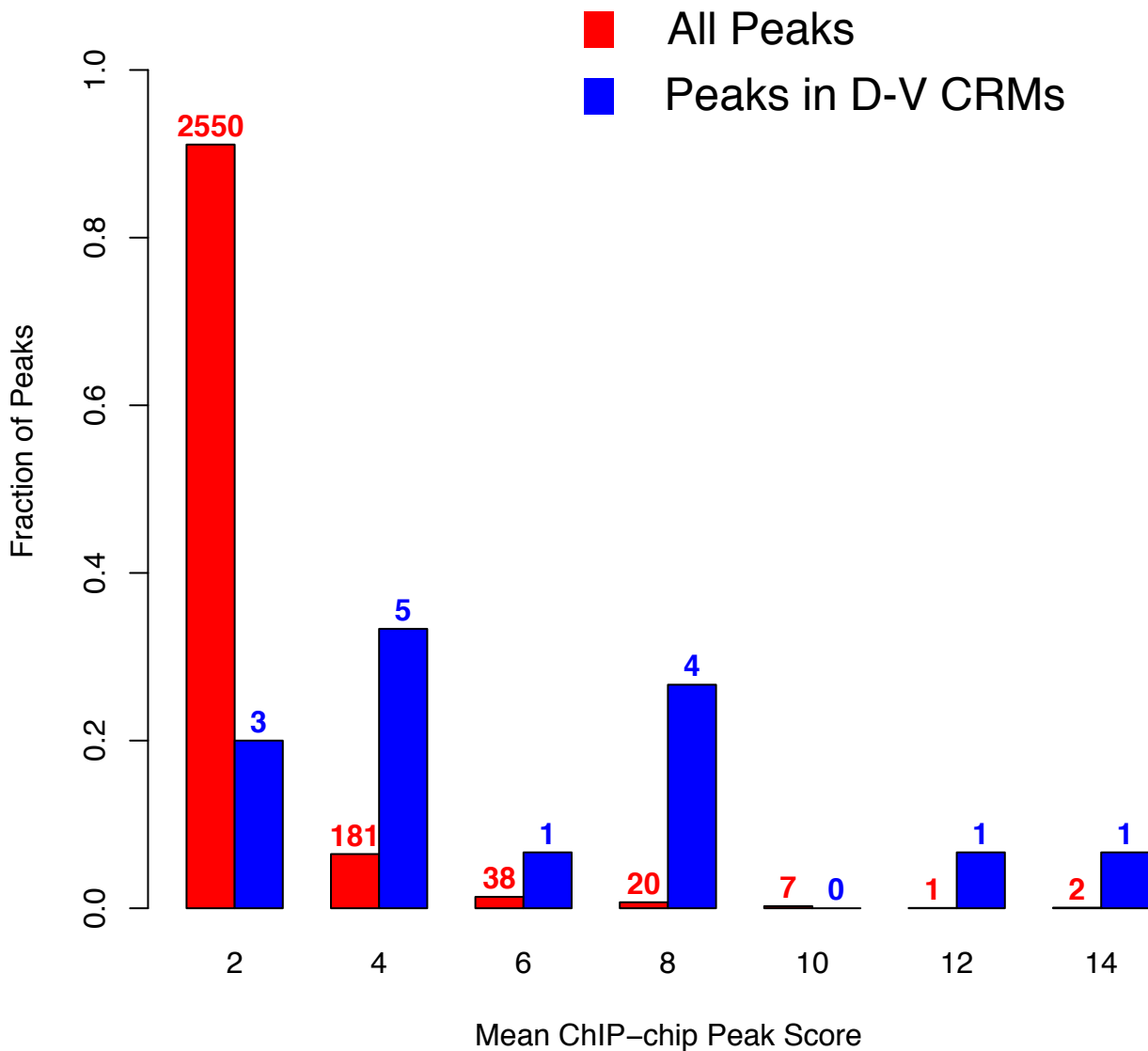

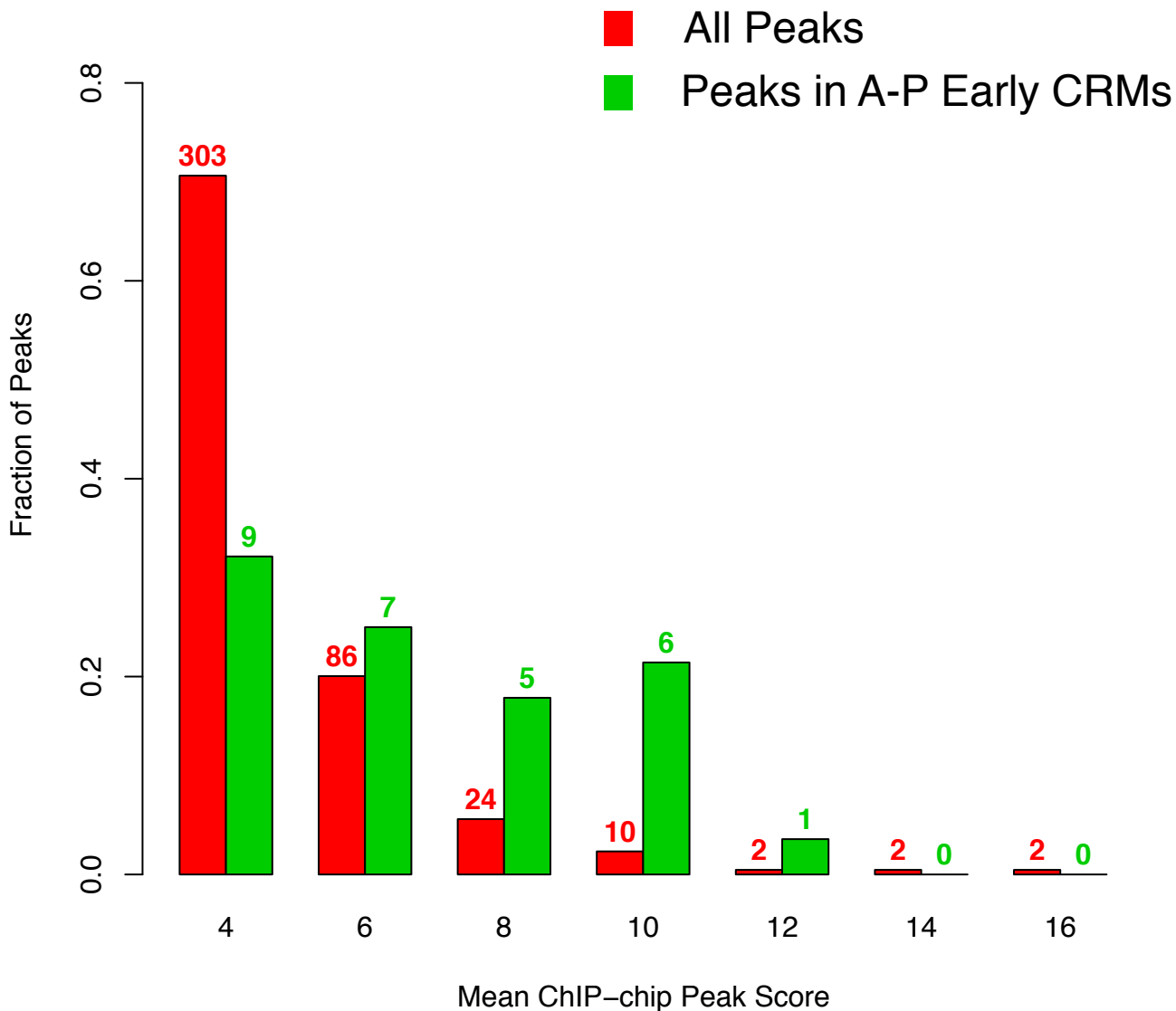

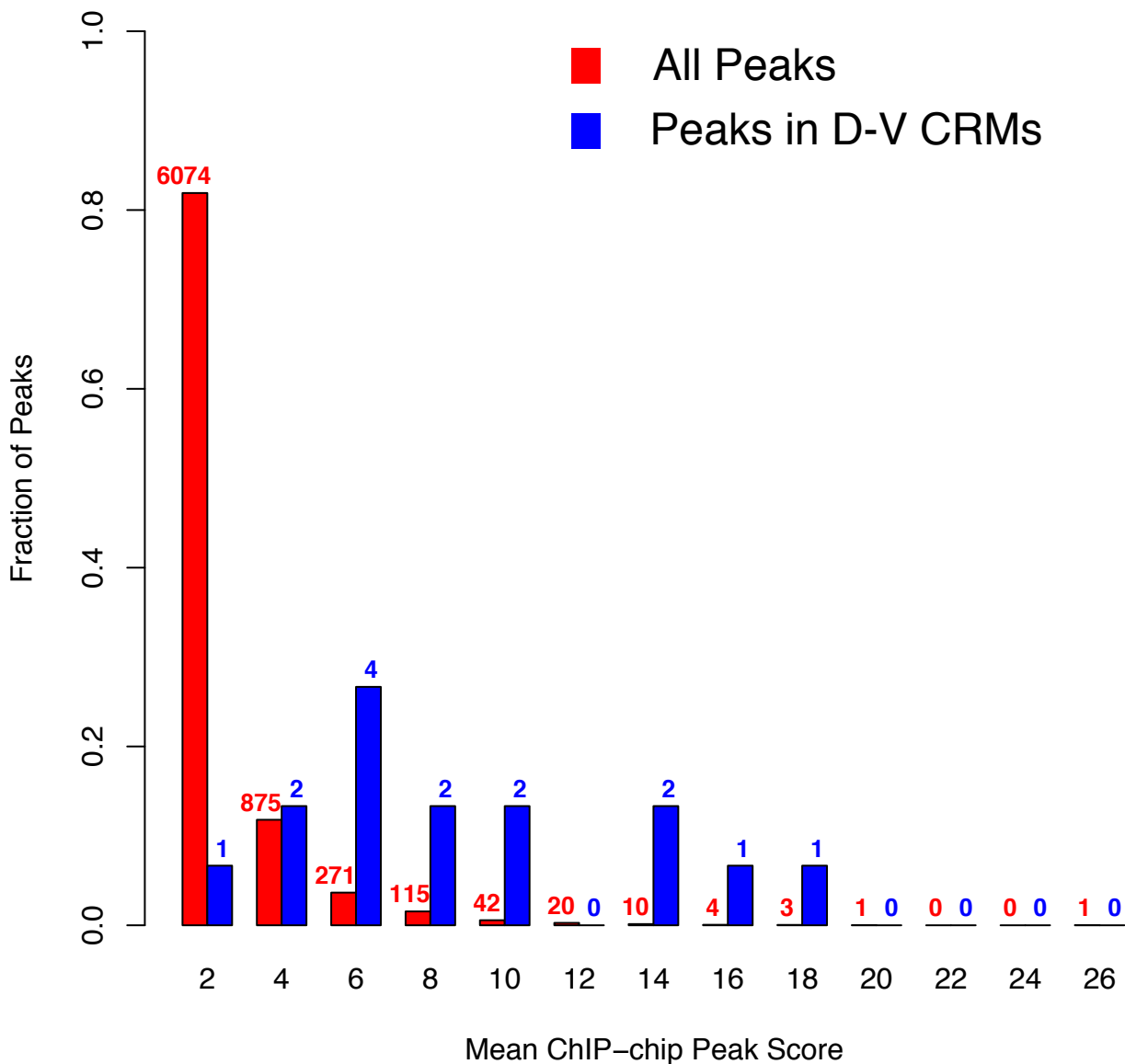

Supplement: Additional data file 8 — Fraction of bound regions in different cohorts distinguished by ChIP/chip score and, for some factors, the fraction of those bound regions that overlap known CRMs, using the conventions shown in Figure 4. [file gb-2009-10-7-r80-S8.pdf]
