# Supplementary material for: Developmental roles of 21 Drosophila transcription factors are determined by quantitative differences in binding to an overlapping set of thousands of genomic regions
Source: Genome Biol. 2009 Jul 23;10(7):R80. doi: 10.1186/gb-2009-10-7-r80 (PMC2728534; doi:10.1186/gb-2009-10-7-r80)
Supplement: Additional data file 9 — These are shown plotted down the ChIP/chip rank list in non-overlapping 200-peak cohorts. [file gb-2009-10-7-r80-S9.pdf]

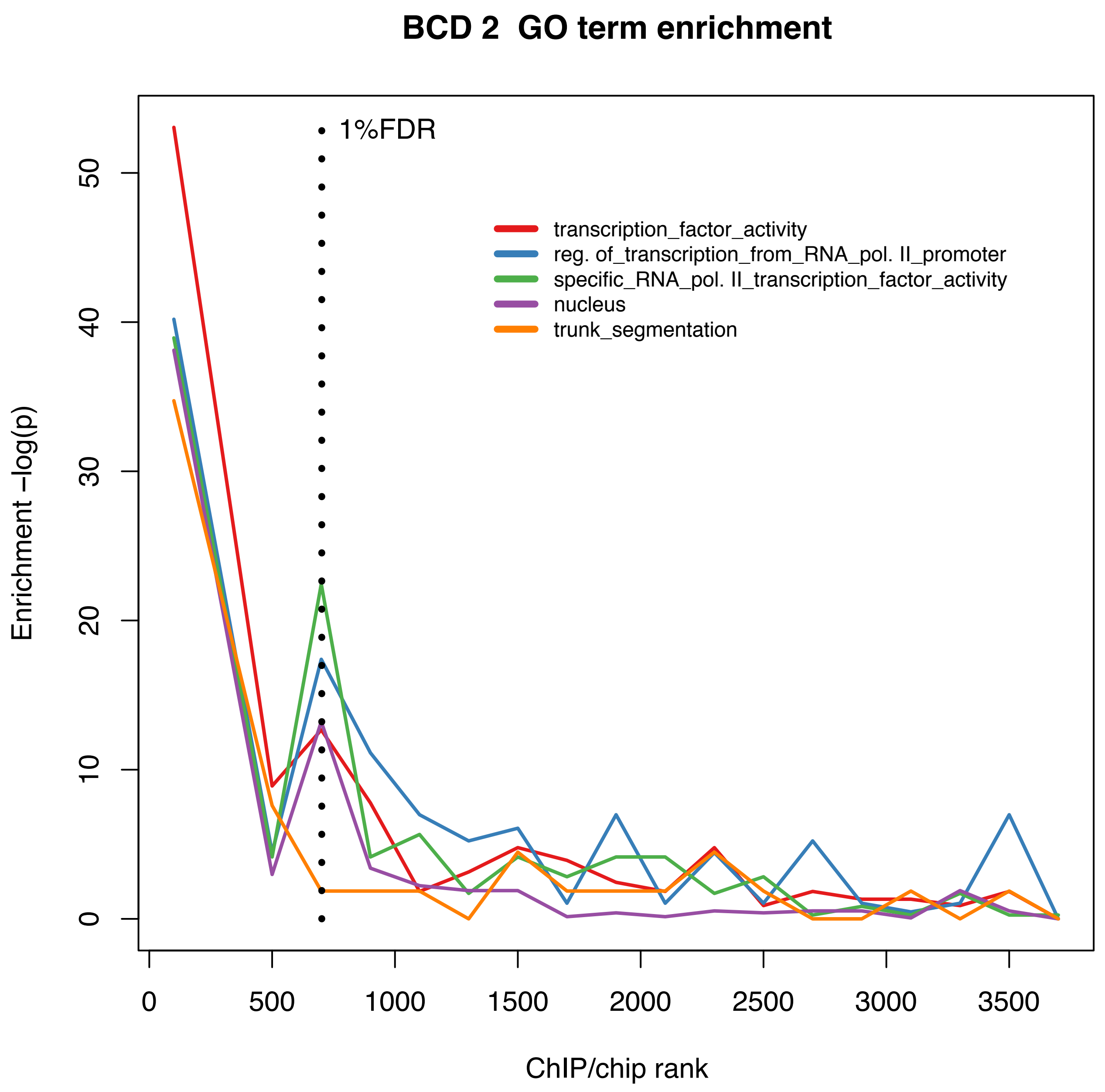
BCD 2 GO term enrichment
1%FDR
transcription_factor_activity
reg. of_transcription_from_RNA_pol. II_promoter
specific_RNA_pol. II_transcription_factor_activity
nucleus
trunk_segmentation
Enrichment −log(p)
0
10
20
30
40
50
0
500
1000
1500
2000
2500
3000
3500
ChIP/chip rank

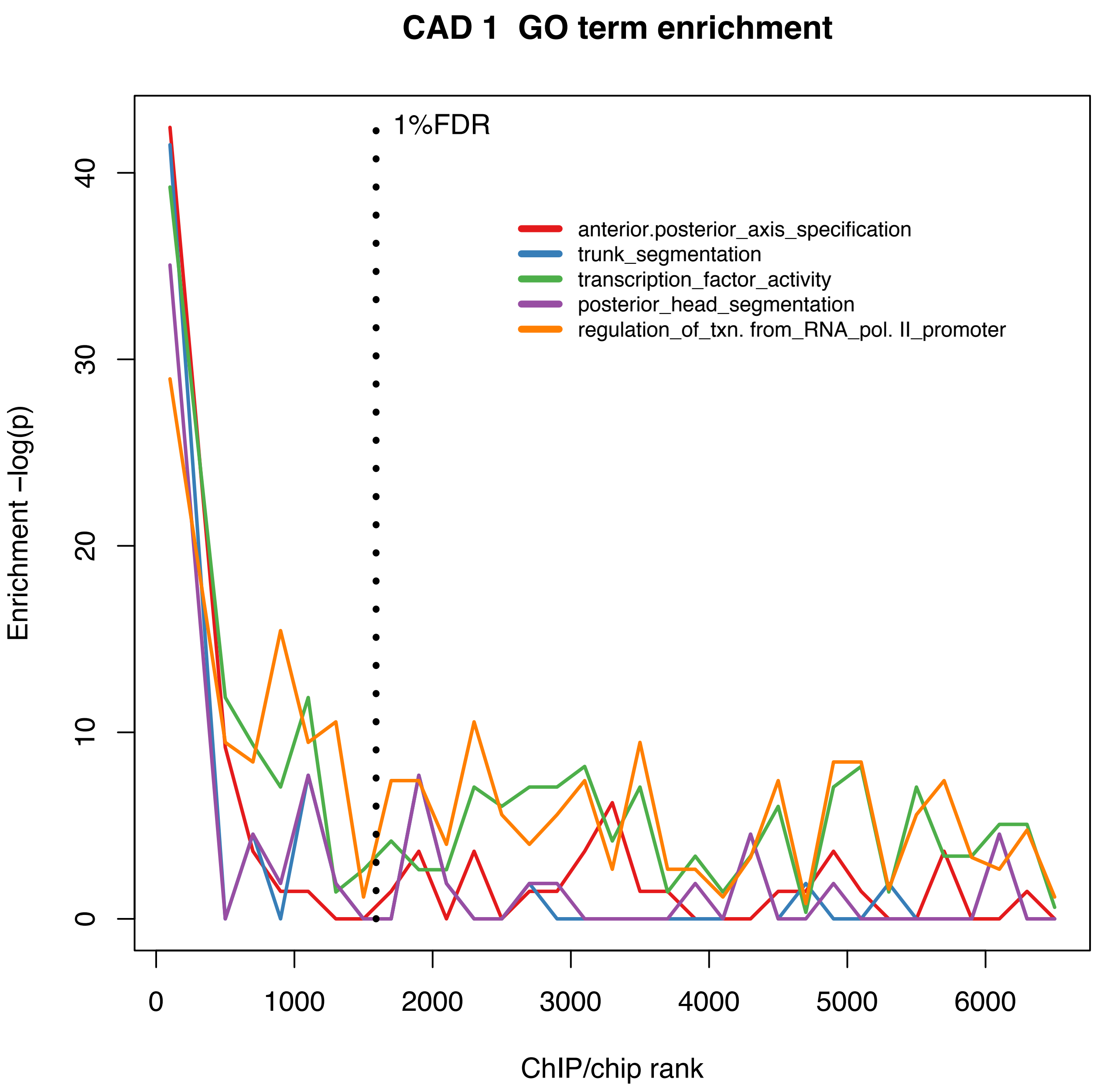
CAD 1 GO term enrichment
1%FDR
anterior.posterior_axis_specification
trunk_segmentation
transcription_factor_activity
posterior_head_segmentation
regulation_of_txn. from_RNA_pol. II_promoter
Enrichment −log(p)
0
10
20
30
40
0
1000
2000
3000
4000
5000
6000
ChIP/chip rank

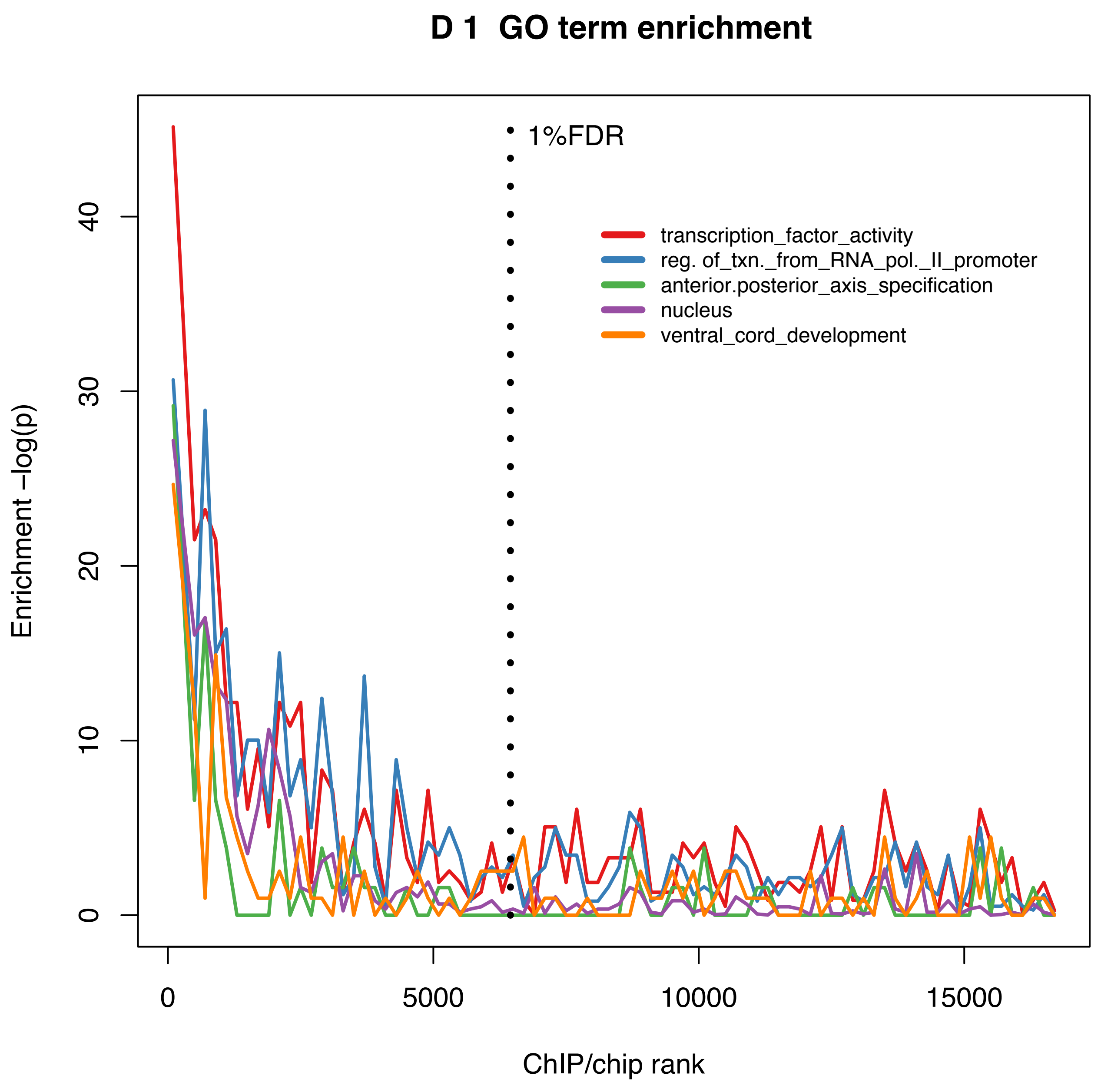
D 1 GO term enrichment
1%FDR
transcription_factor_activity
reg. of_txn._from_RNA_pol._II_promoter
anterior.posterior_axis_specification
nucleus
ventral_cord_development
Enrichment −log(p)
0
10
20
30
40
0
5000
10000
15000
ChIP/chip rank

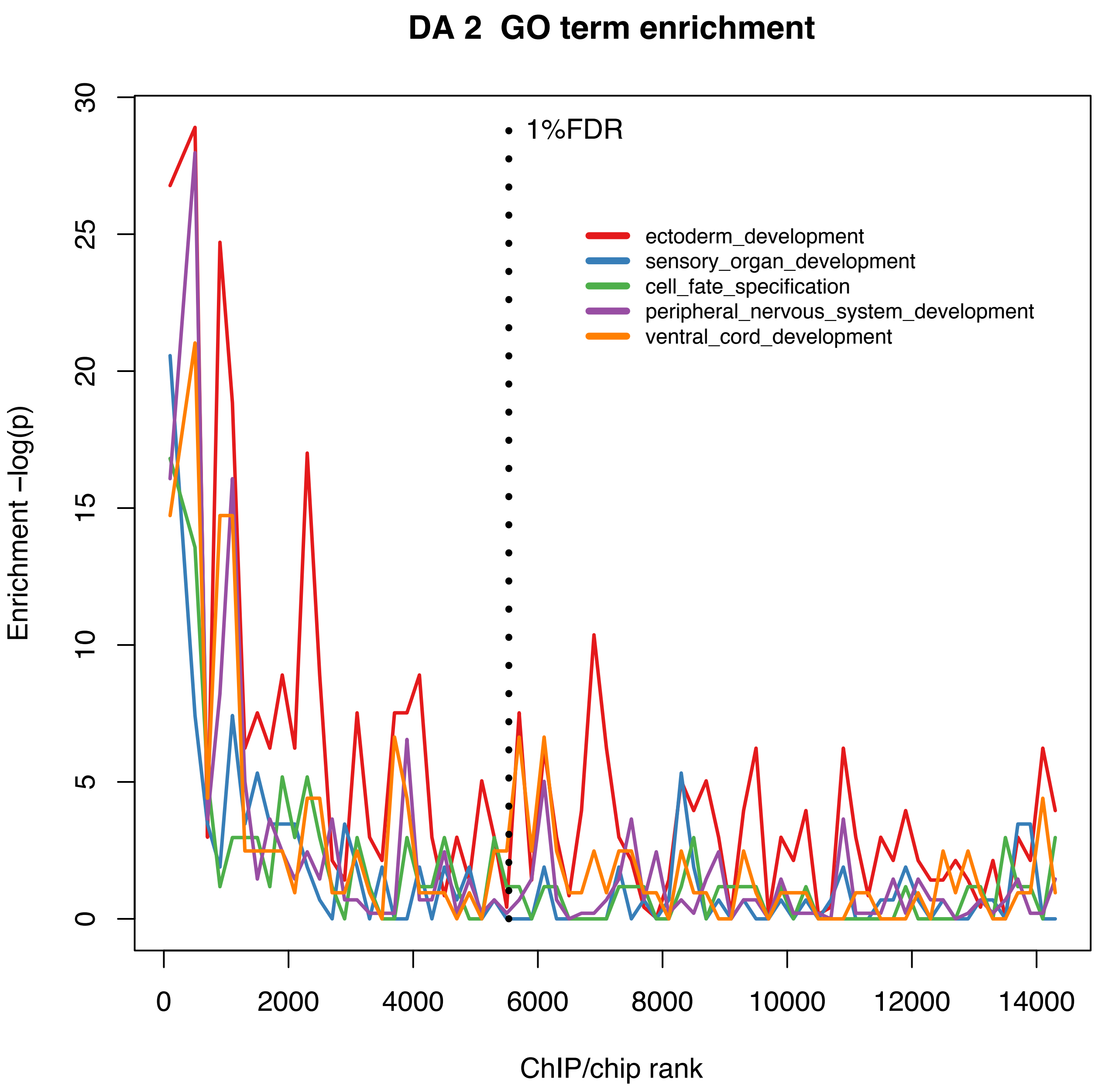
DA 2 GO term enrichment
Enrichment −log(p)
0
5
10
15
20
25
30
1%FDR
ectoderm_development
sensory_organ_development
cell_fate_specification
peripheral_nervous_system_development
ventral_cord_development
0
2000
4000
6000
8000
10000
12000
14000
ChIP/chip rank

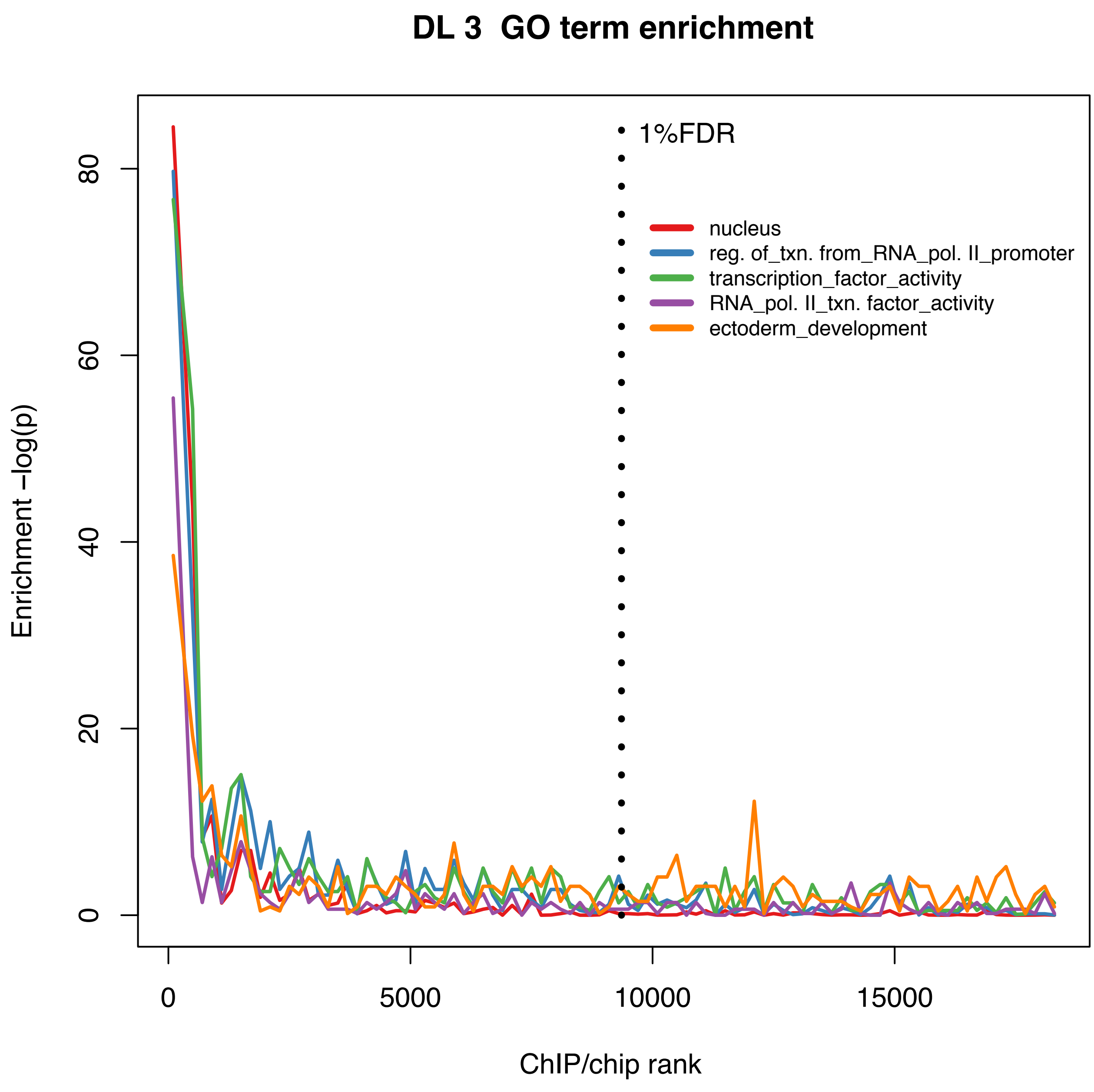
DL 3 GO term enrichment
1%FDR
nucleus
reg. of_txn. from_RNA_pol. II_promoter
transcription_factor_activity
RNA_pol. II_txn. factor_activity
ectoderm_development
Enrichment −log(p)
0
20
40
60
80
0
5000
10000
15000
ChIP/chip rank

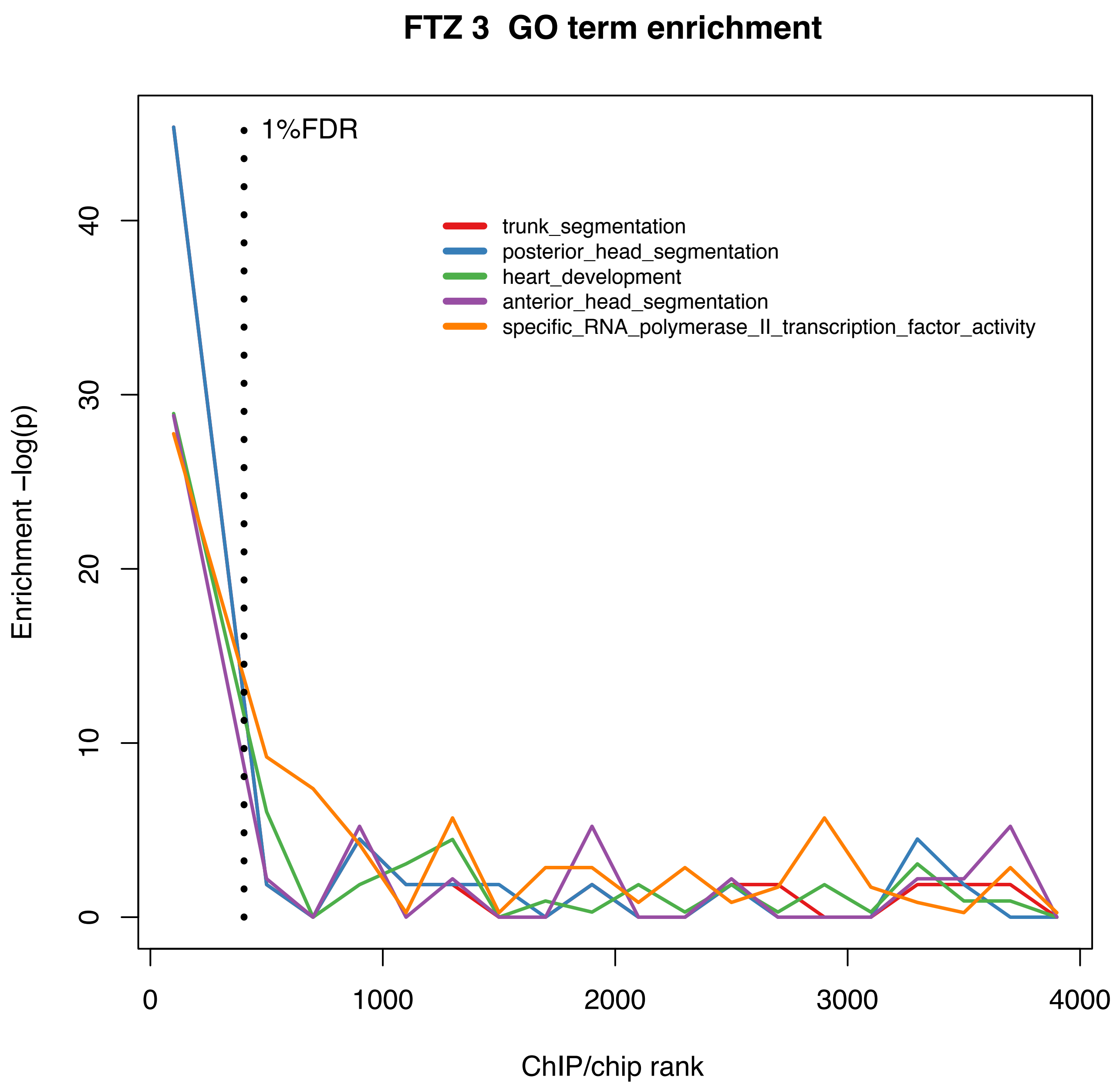
FTZ 3 GO term enrichment
1%FDR
trunk_segmentation
posterior_head_segmentation
heart_development
anterior_head_segmentation
specific_RNA_polymerase_II_transcription_factor_activity
Enrichment −log(p)
0
10
20
30
40
0
1000
2000
3000
4000
ChIP/chip rank

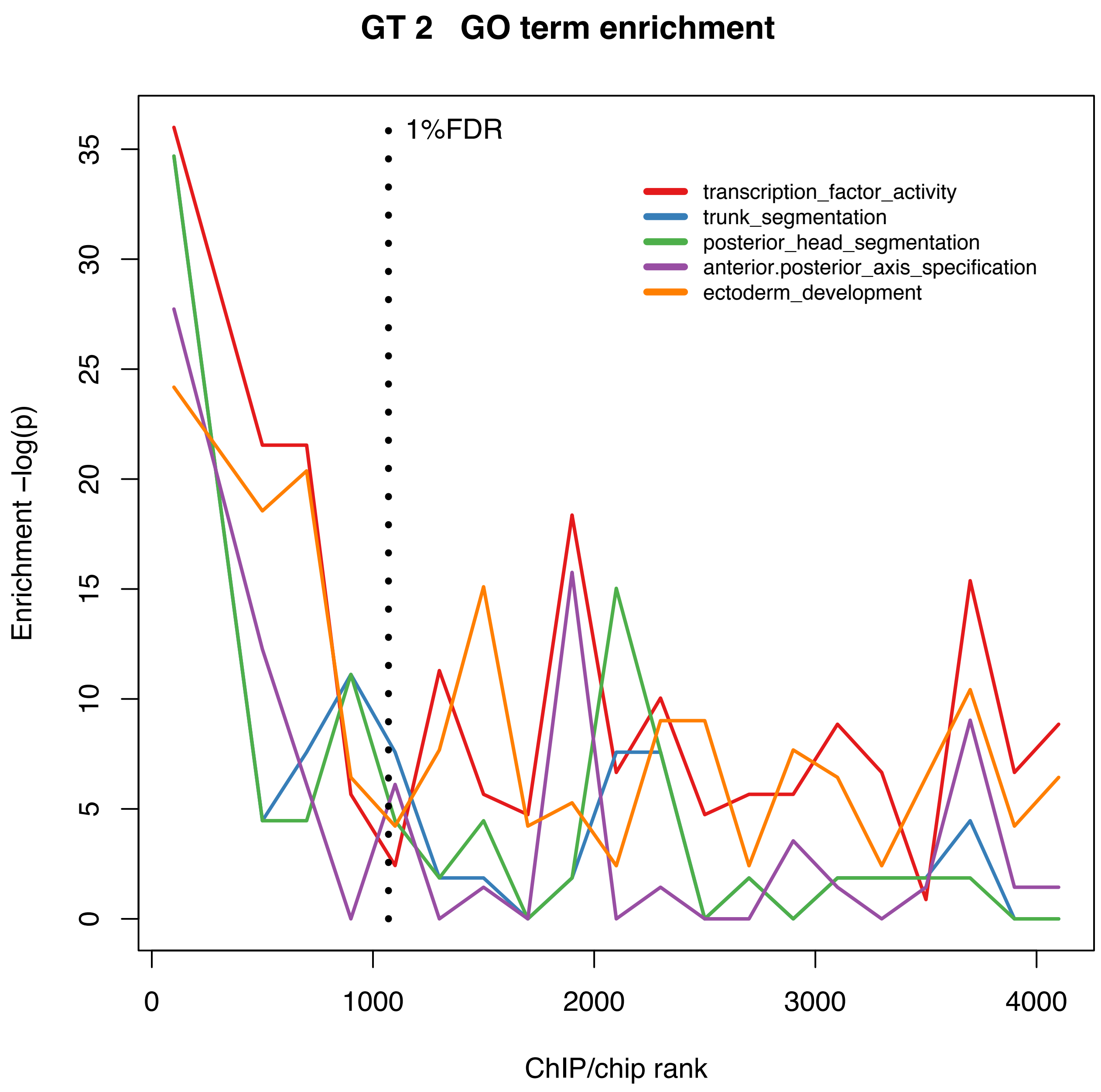
GT 2 GO term enrichment
1%FDR
transcription_factor_activity
trunk_segmentation
posterior_head_segmentation
anterior.posterior_axis_specification
ectoderm_development
Enrichment −log(p)
ChIP/chip rank
0
5
10
15
20
25
30
35
0
1000
2000
3000
4000

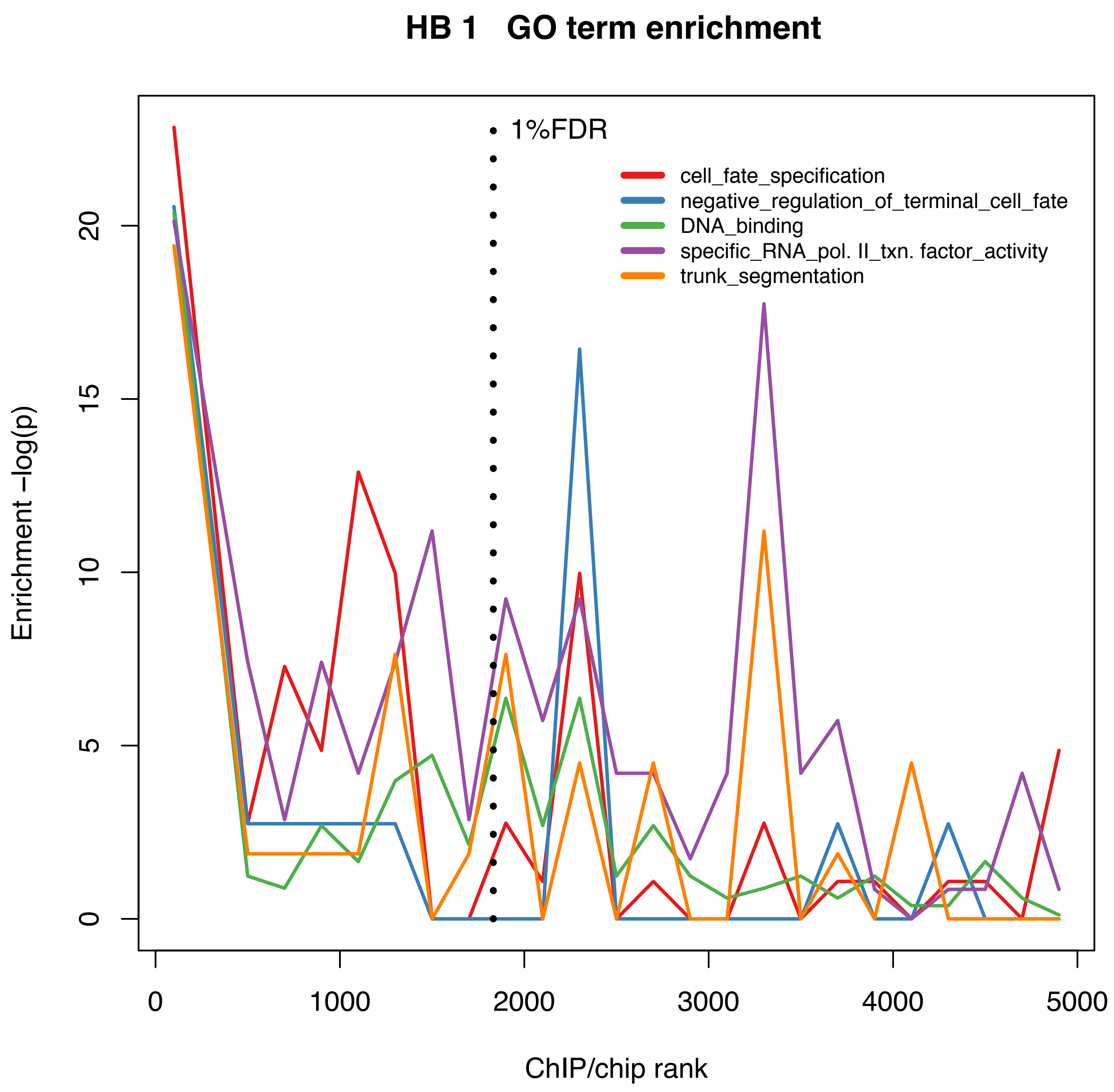
HB 1 GO term enrichment
1%FDR
cell_fate_specification
negative_regulation_of_terminal_cell_fate
DNA_binding
specific_RNA_pol. II_txn. factor_activity
trunk_segmentation
Enrichment −log(p)
0
5
10
15
20
0
1000
2000
3000
4000
5000
ChIP/chip rank

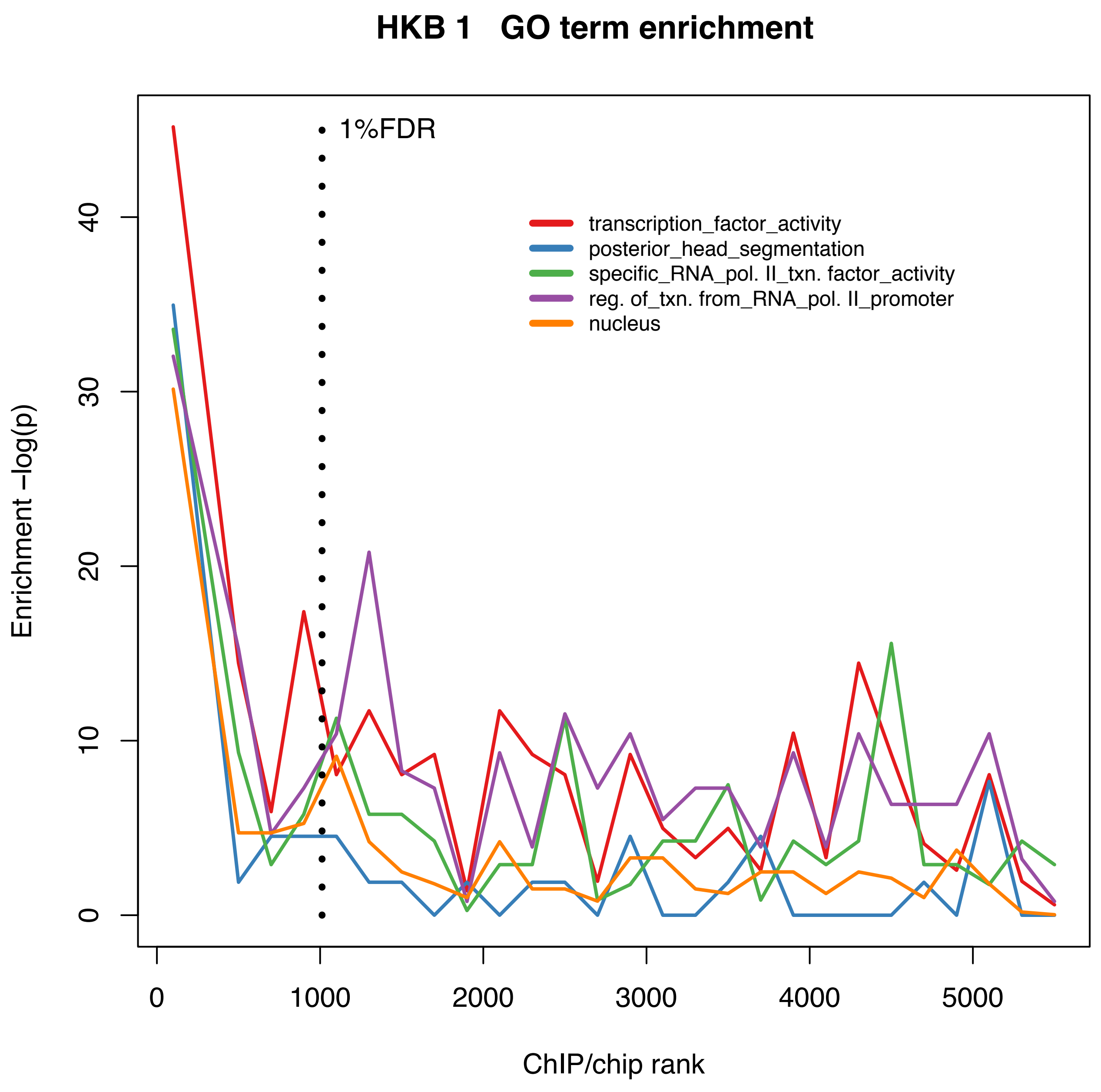
HKB 1   GO term enrichment
1%FDR
transcription_factor_activity
posterior_head_segmentation
specific_RNA_pol. II_txn. factor_activity
reg. of_txn. from_RNA_pol. II_promoter
nucleus
Enrichment −log(p)
0
10
20
30
40
0
1000
2000
3000
4000
5000
ChIP/chip rank

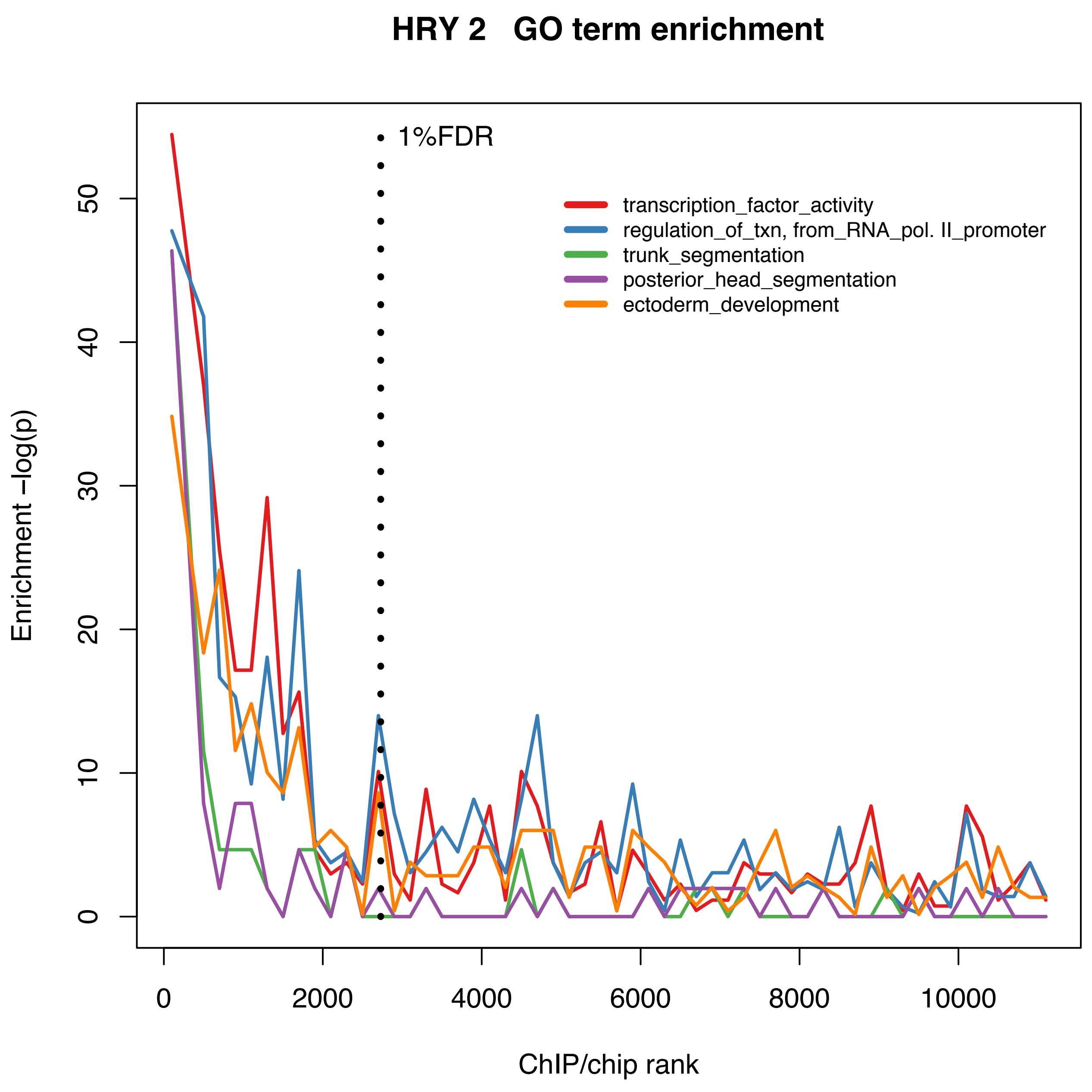
HRY 2 GO term enrichment
1%FDR
transcription_factor_activity
regulation_of_txn, from_RNA_pol. II_promoter
trunk_segmentation
posterior_head_segmentation
ectoderm_development
Enrichment −log(p)
0
10
20
30
40
50
0
2000
4000
6000
8000
10000
ChIP/chip rank

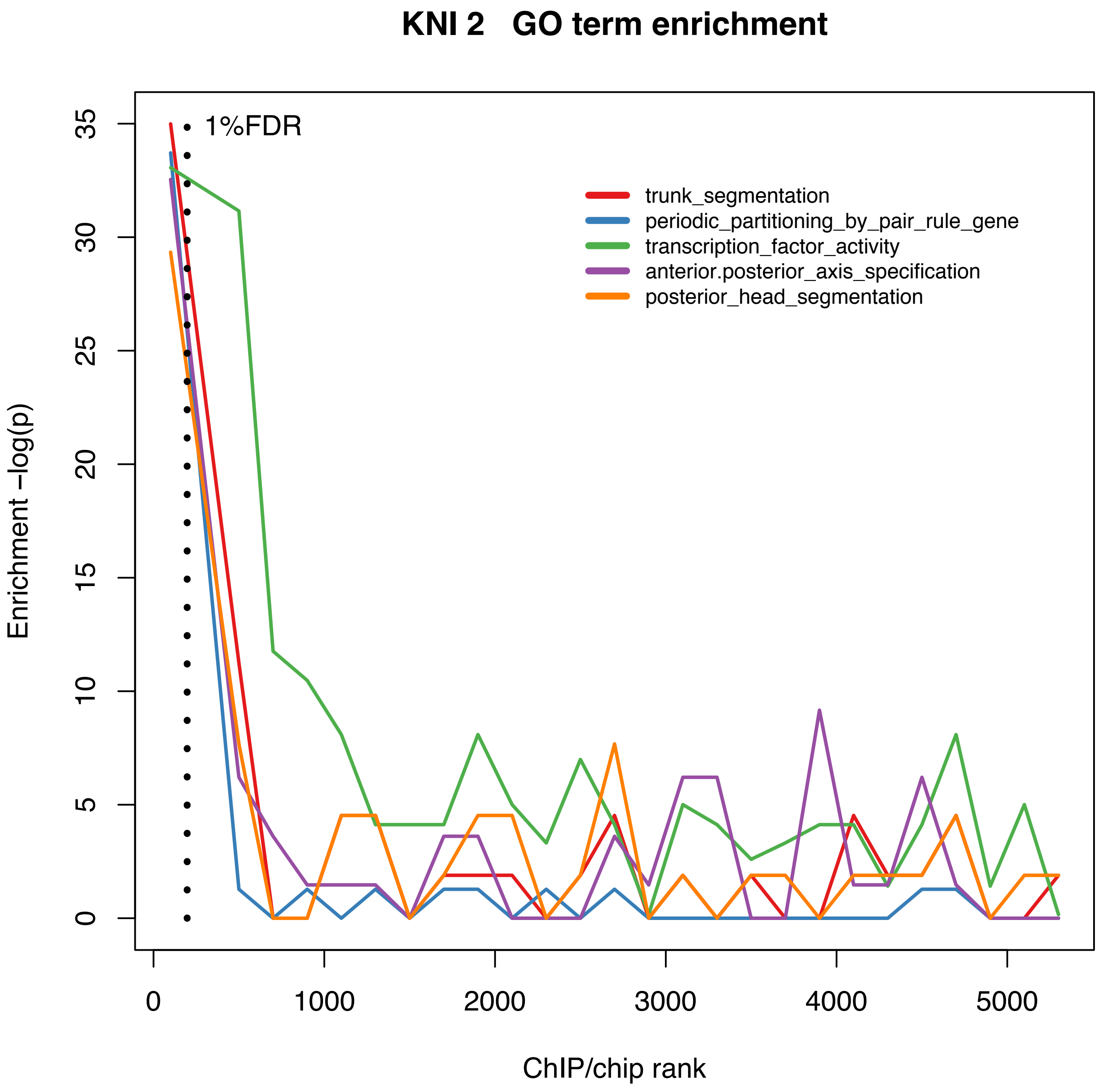
KNI 2   GO term enrichment
1%FDR
trunk_segmentation
periodic_partitioning_by_pair_rule_gene
transcription_factor_activity
anterior.posterior_axis_specification
posterior_head_segmentation
Enrichment −log(p)
0
5
10
15
20
25
30
35
0
1000
2000
3000
4000
5000
ChIP/chip rank

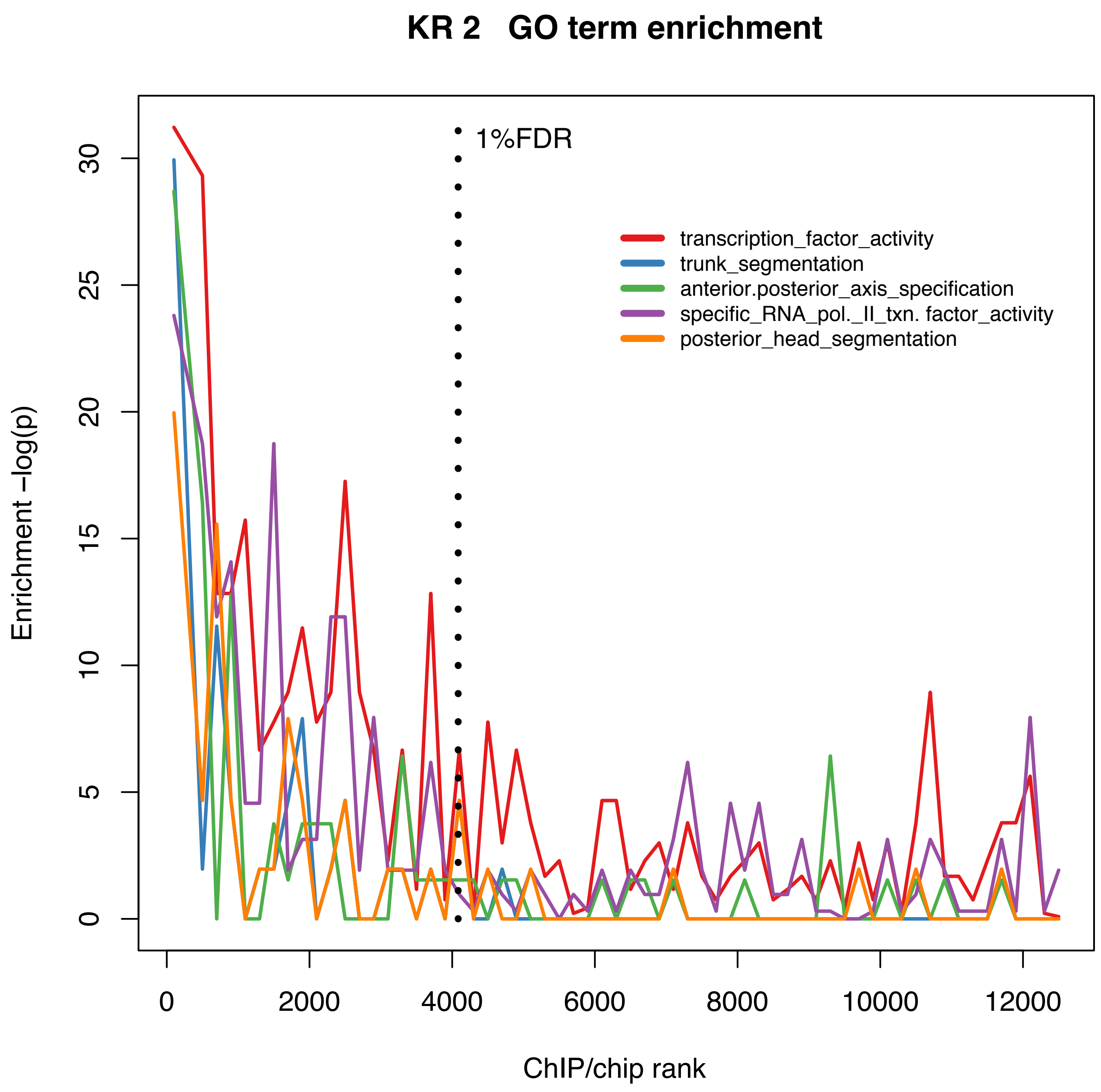
KR 2 GO term enrichment
1%FDR
transcription_factor_activity
trunk_segmentation
anterior.posterior_axis_specification
specific_RNA_pol._II_txn. factor_activity
posterior_head_segmentation
Enrichment −log(p)
0
5
10
15
20
25
30
0
2000
4000
6000
8000
10000
12000
ChIP/chip rank

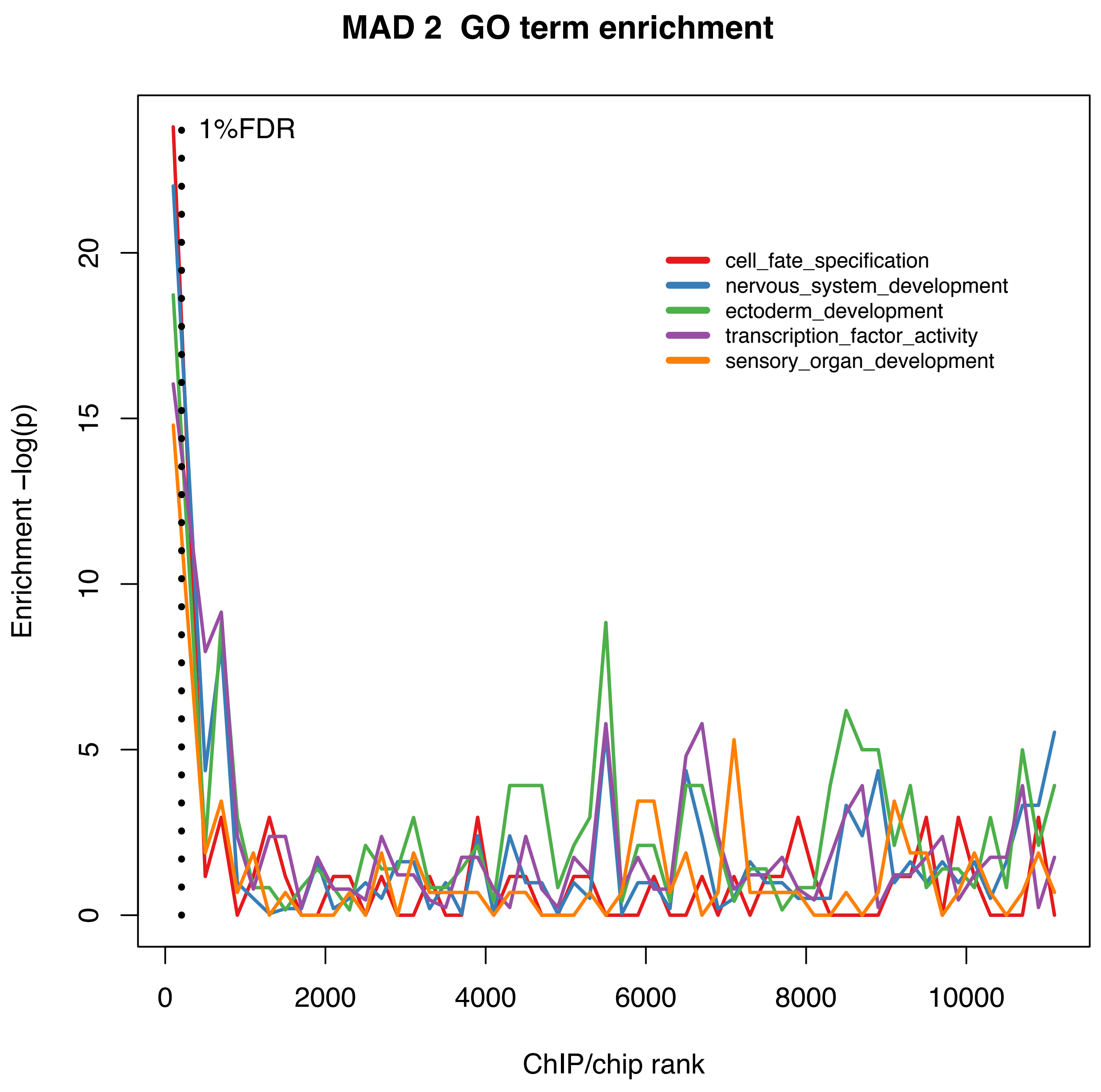
MAD 2 GO term enrichment
1%FDR
cell_fate_specification
nervous_system_development
ectoderm_development
transcription_factor_activity
sensory_organ_development
Enrichment −log(p)
0
5
10
15
20
0
2000
4000
6000
8000
10000
ChIP/chip rank

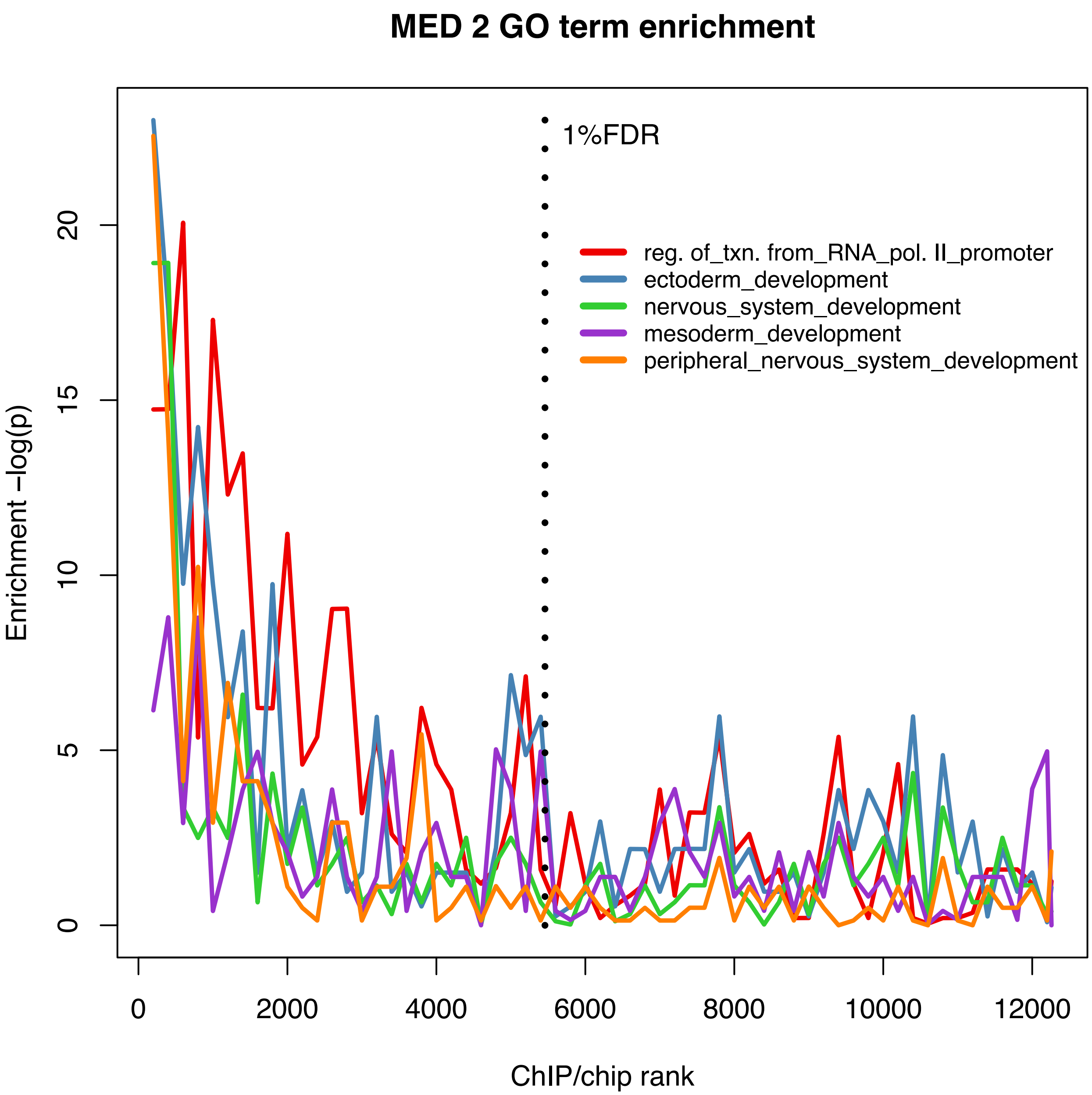
MED 2 GO term enrichment
1%FDR
reg. of_txn. from_RNA_pol. II_promoter
ectoderm_development
nervous_system_development
mesoderm_development
peripheral_nervous_system_development
Enrichment −log(p)
0
5
10
15
20
0
2000
4000
6000
8000
10000
12000
ChIP/chip rank

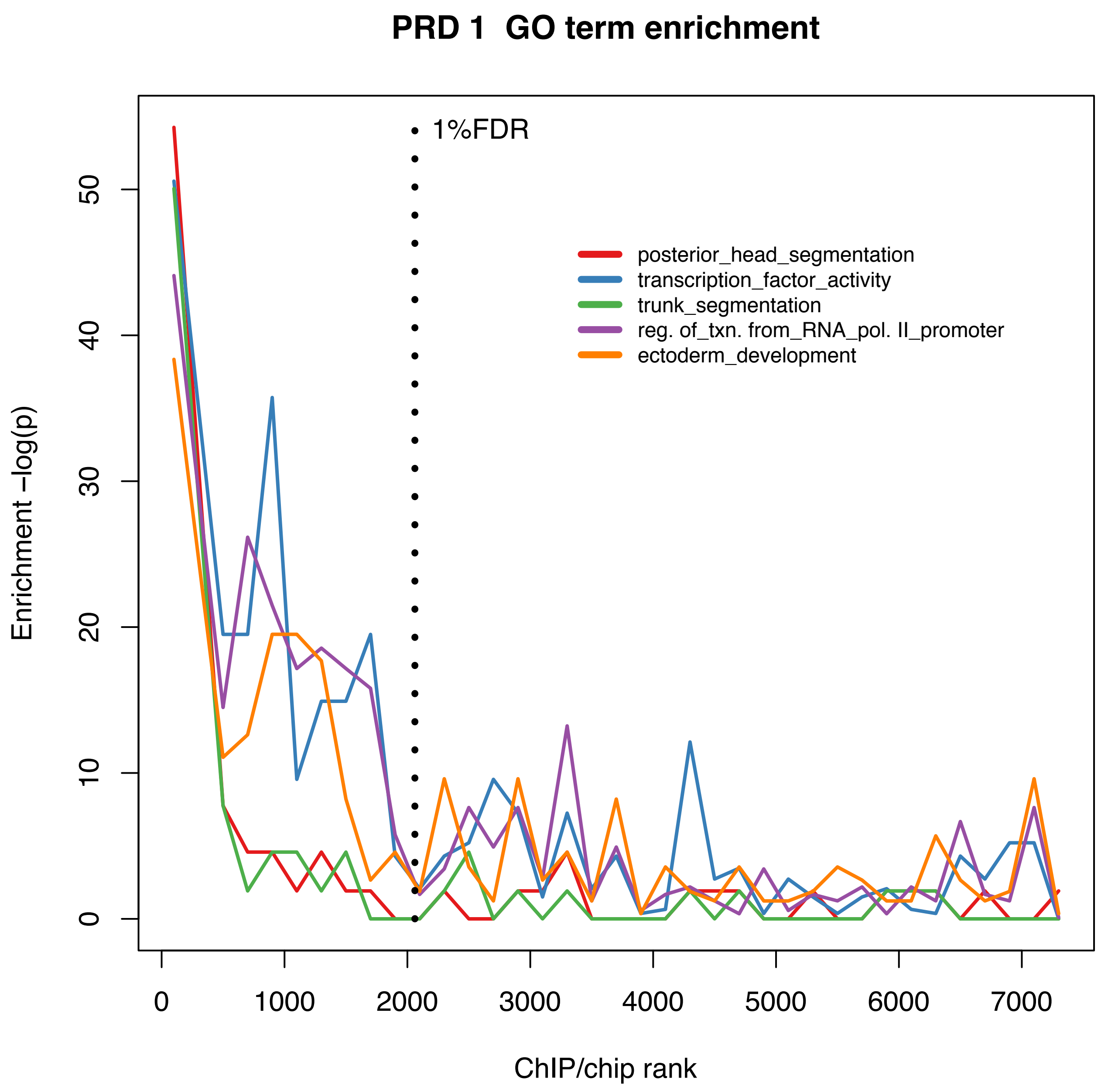
PRD 1 GO term enrichment
1%FDR
posterior_head_segmentation
transcription_factor_activity
trunk_segmentation
reg. of_txn. from_RNA_pol. II_promoter
ectoderm_development
Enrichment −log(p)
0
10
20
30
40
50
0
1000
2000
3000
4000
5000
6000
7000
ChIP/chip rank

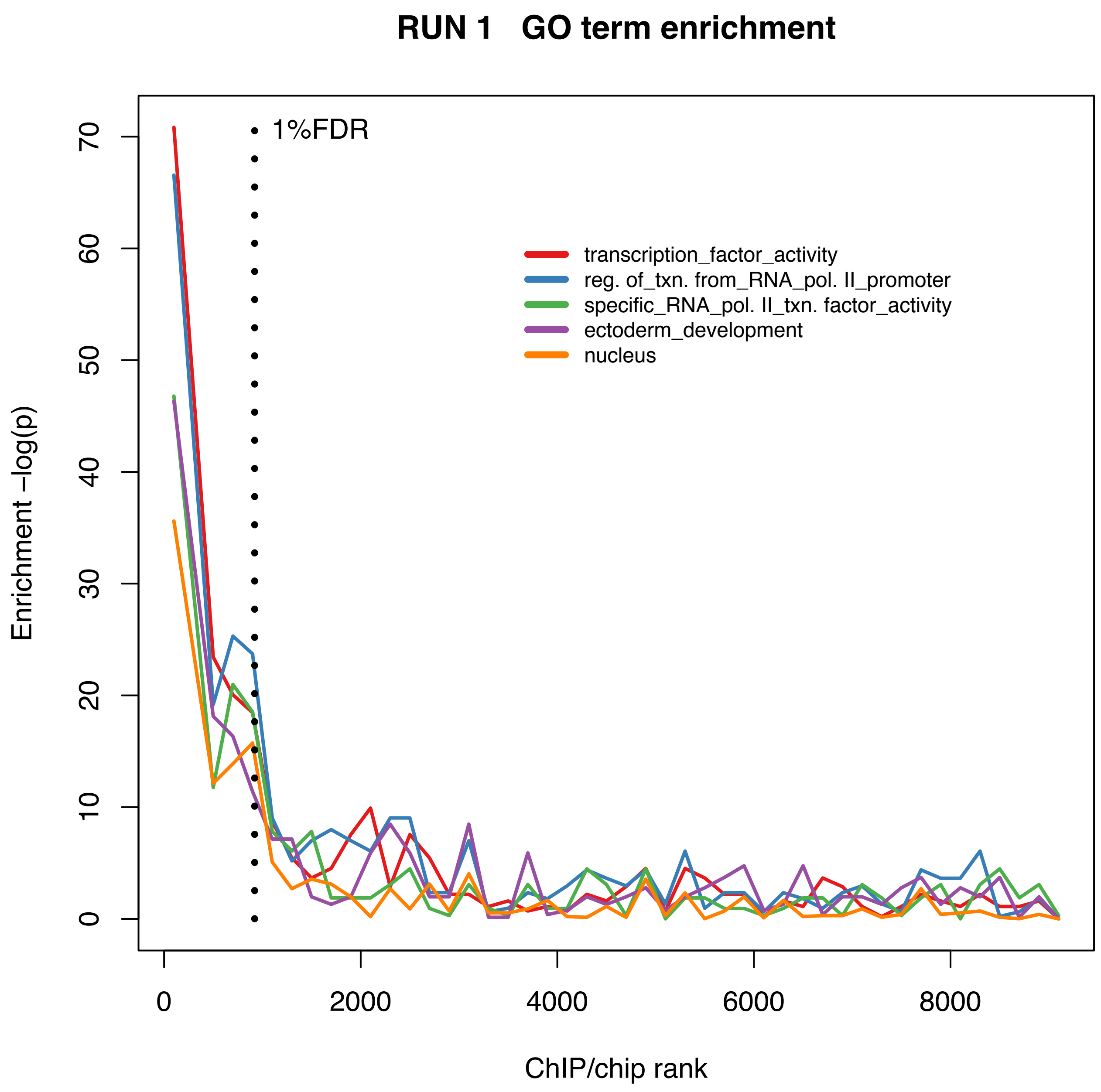
RUN 1 GO term enrichment
1%FDR
transcription_factor_activity
reg. of_txn. from_RNA_pol. II_promoter
specific_RNA_pol. II_txn. factor_activity
ectoderm_development
nucleus
Enrichment −log(p)
0
10
20
30
40
50
60
70
0
2000
4000
6000
8000
ChIP/chip rank

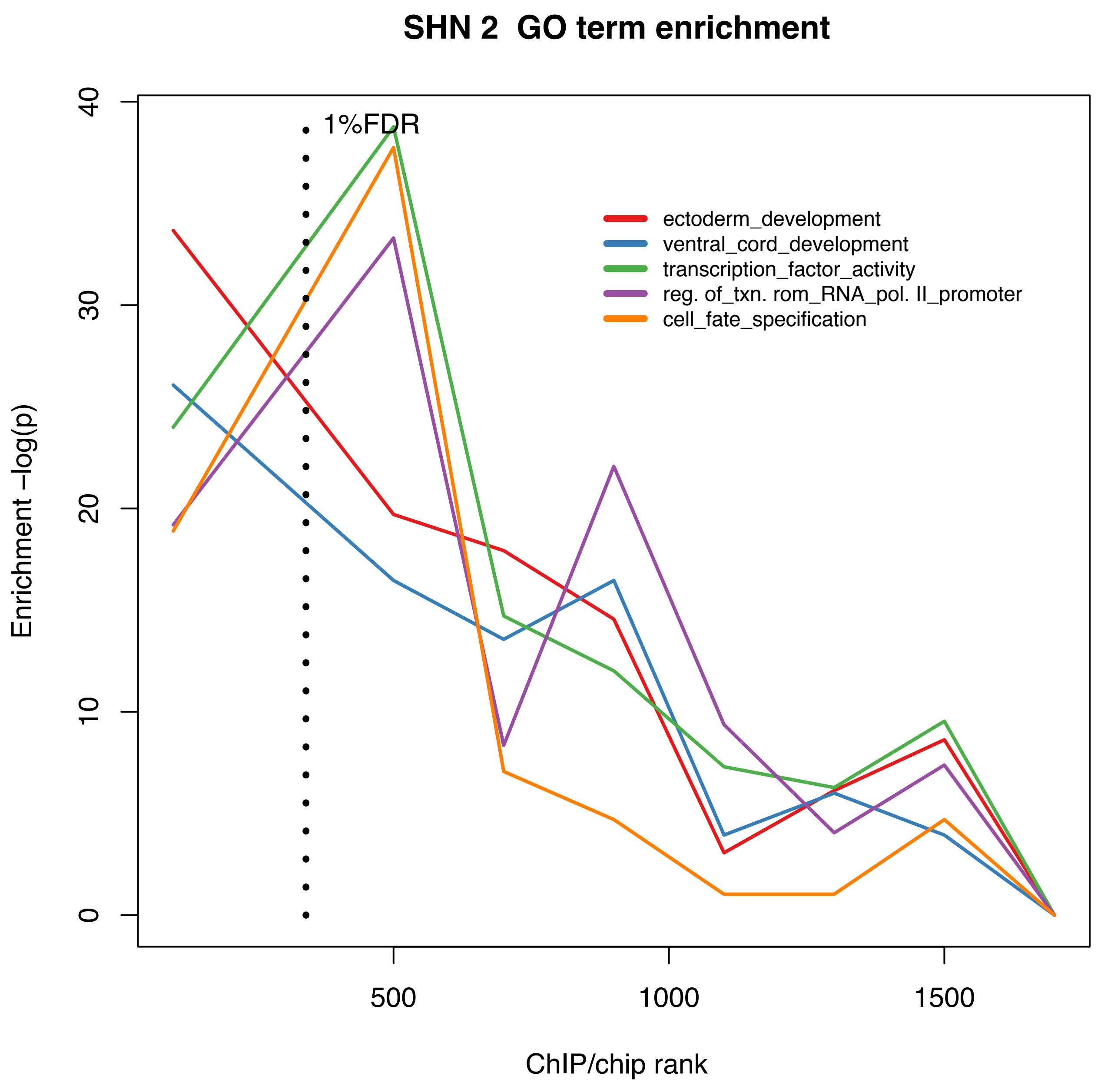
SHN 2 GO term enrichment
1%FDR
ectoderm_development
ventral_cord_development
transcription_factor_activity
reg. of_txn. rom_RNA_pol. II_promoter
cell_fate_specification
Enrichment −log(p)
0
10
20
30
40
500
1000
1500
ChIP/chip rank

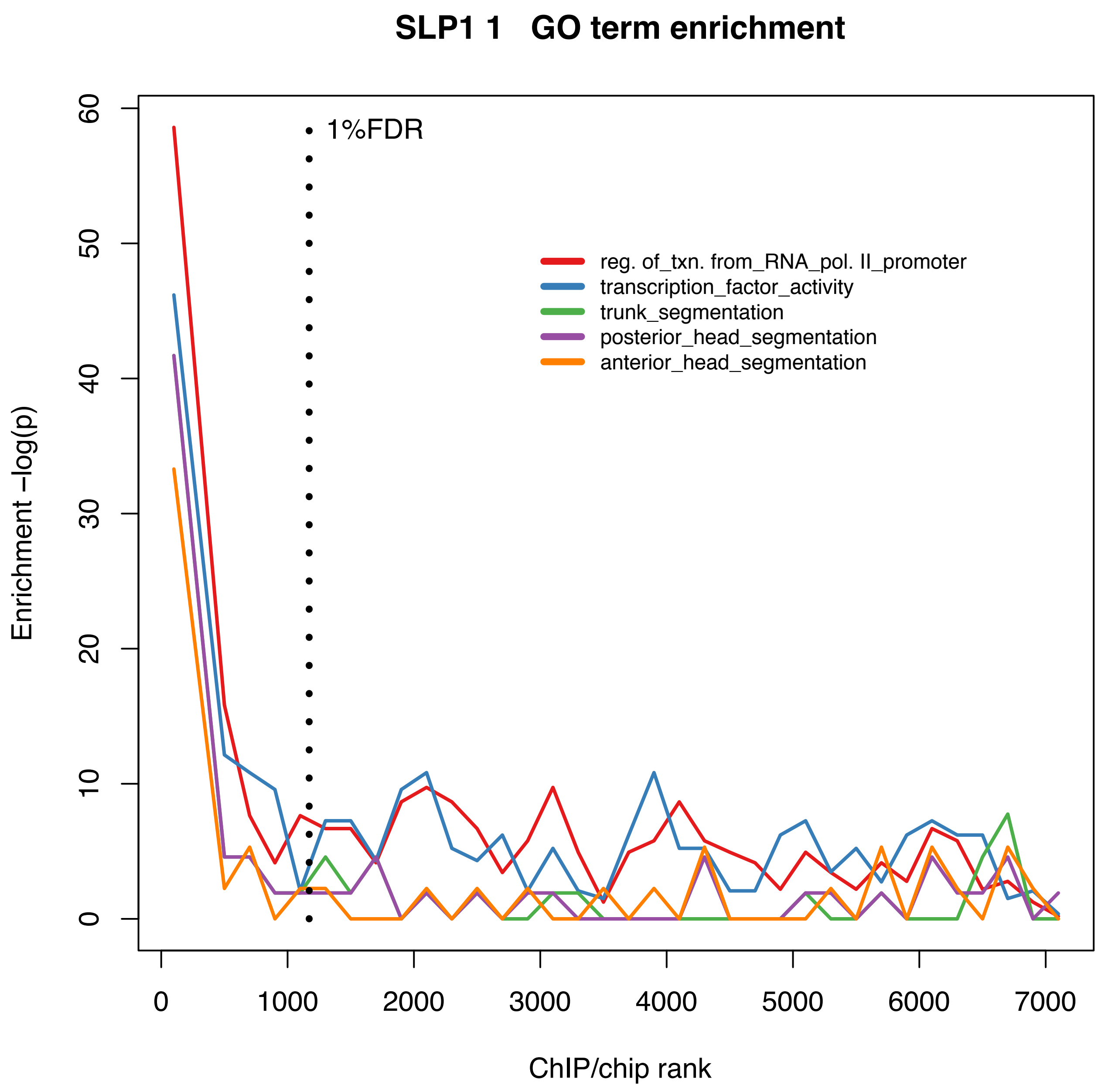
SLP1 1   GO term enrichment
1%FDR
reg. of_txn. from_RNA_pol. II_promoter
transcription_factor_activity
trunk_segmentation
posterior_head_segmentation
anterior_head_segmentation
Enrichment −log(p)
0
10
20
30
40
50
60
0
1000
2000
3000
4000
5000
6000
7000
ChIP/chip rank

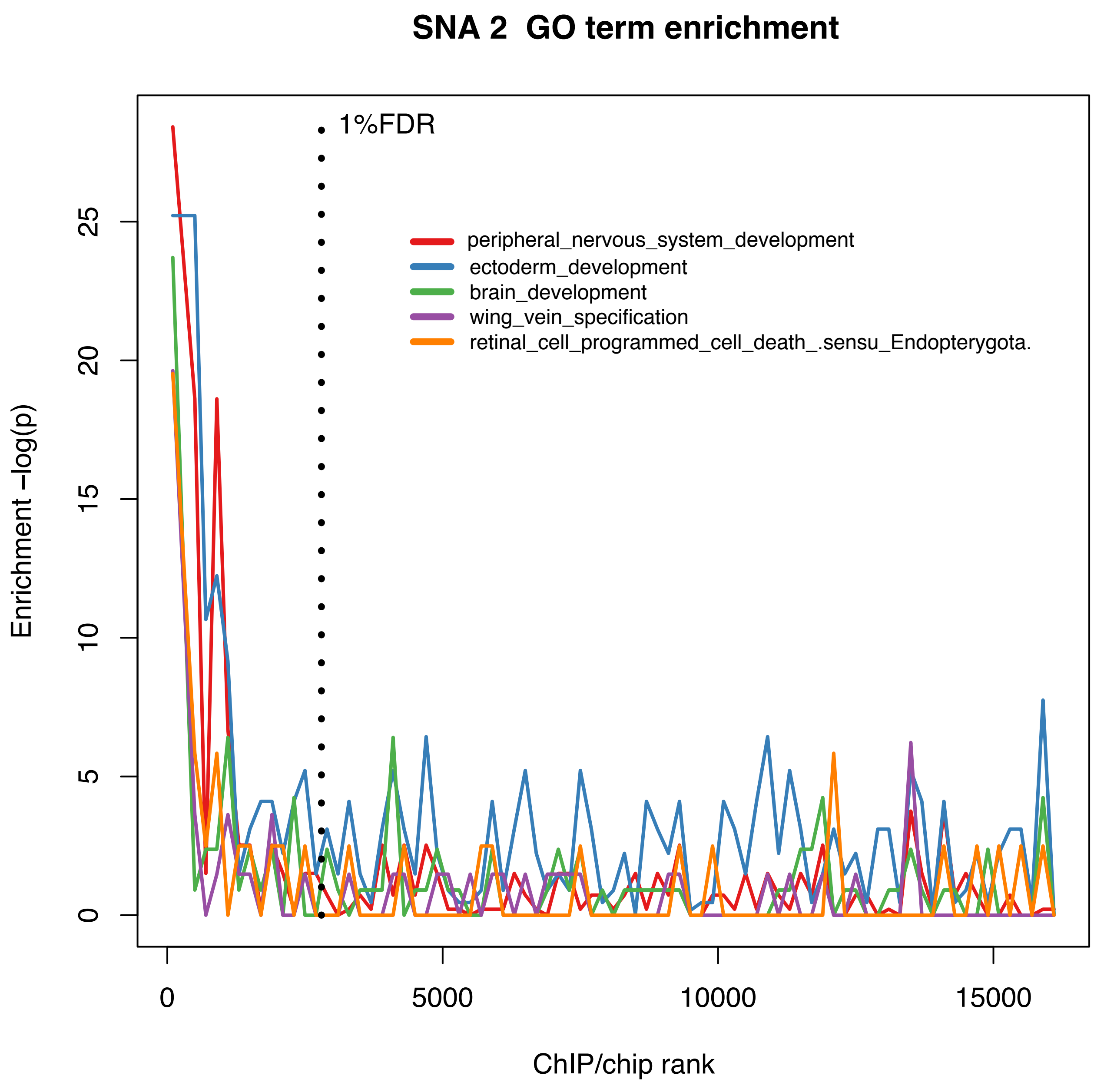
SNA 2 GO term enrichment
1%FDR
peripheral_nervous_system_development
ectoderm_development
brain_development
wing_vein_specification
retinal_cell_programmed_cell_death_.sensu_Endopterygota.
Enrichment −log(p)
0
5
10
15
20
25
0
5000
10000
15000
ChIP/chip rank

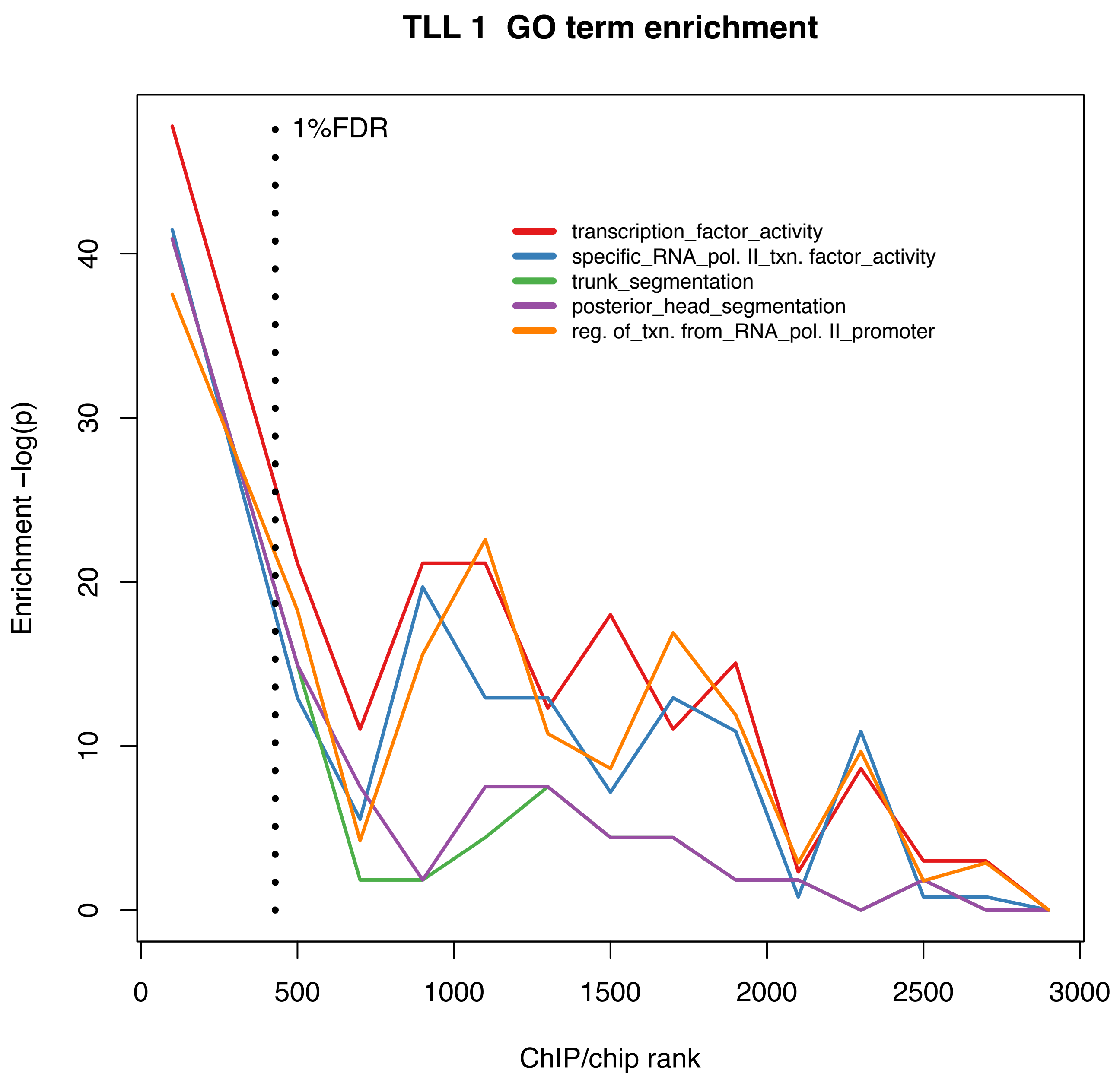
TLL 1 GO term enrichment
1%FDR
transcription_factor_activity
specific_RNA_pol. II_txn. factor_activity
trunk_segmentation
posterior_head_segmentation
reg. of_txn. from_RNA_pol. II_promoter
Enrichment −log(p)
0
10
20
30
40
0
500
1000
1500
2000
2500
3000
ChIP/chip rank

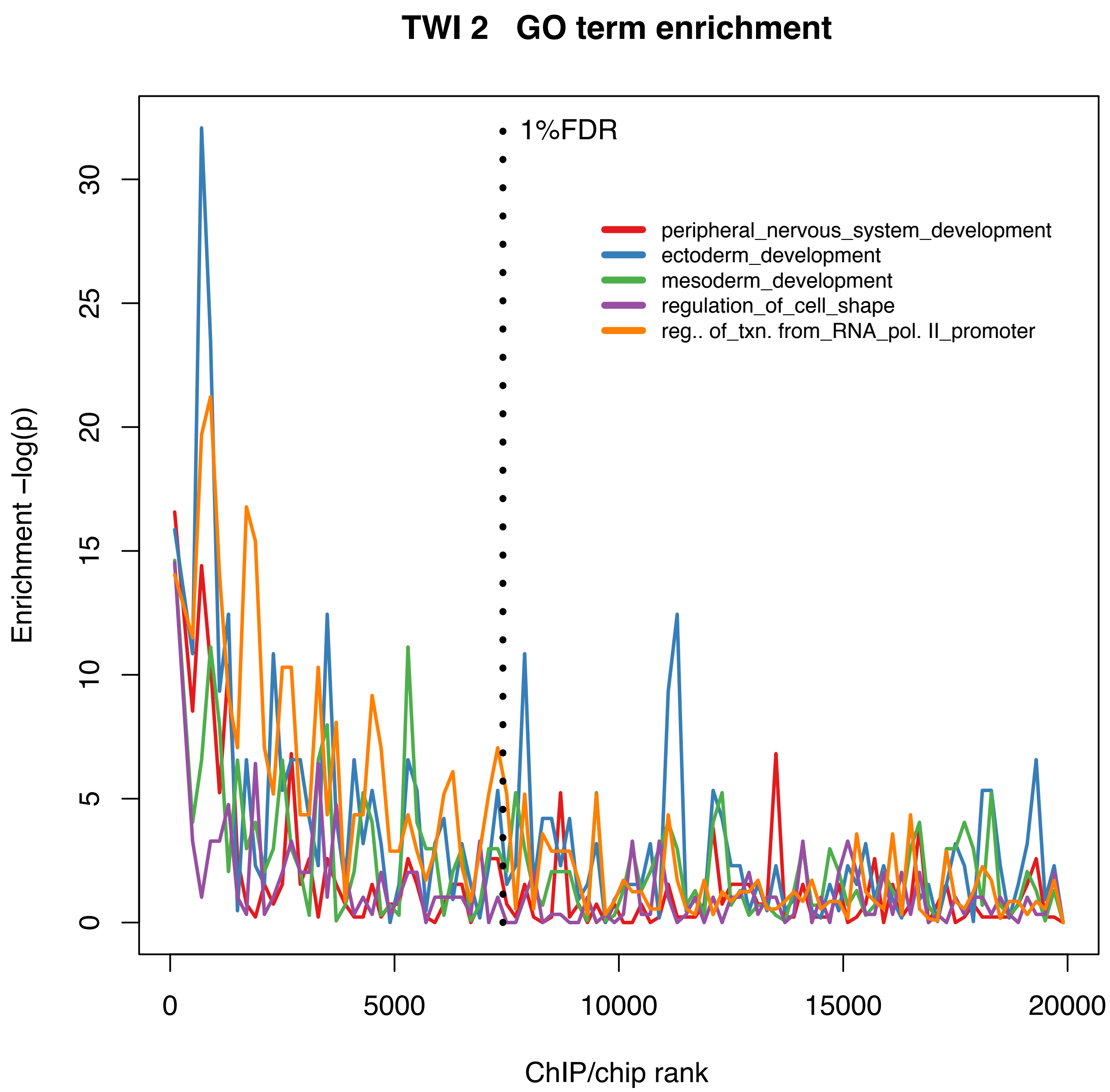
TWI 2   GO term enrichment
1%FDR
peripheral_nervous_system_development
ectoderm_development
mesoderm_development
regulation_of_cell_shape
reg.. of_txn. from_RNA_pol. II_promoter
Enrichment −log(p)
0
5
10
15
20
25
30
0
5000
10000
15000
20000
ChIP/chip rank

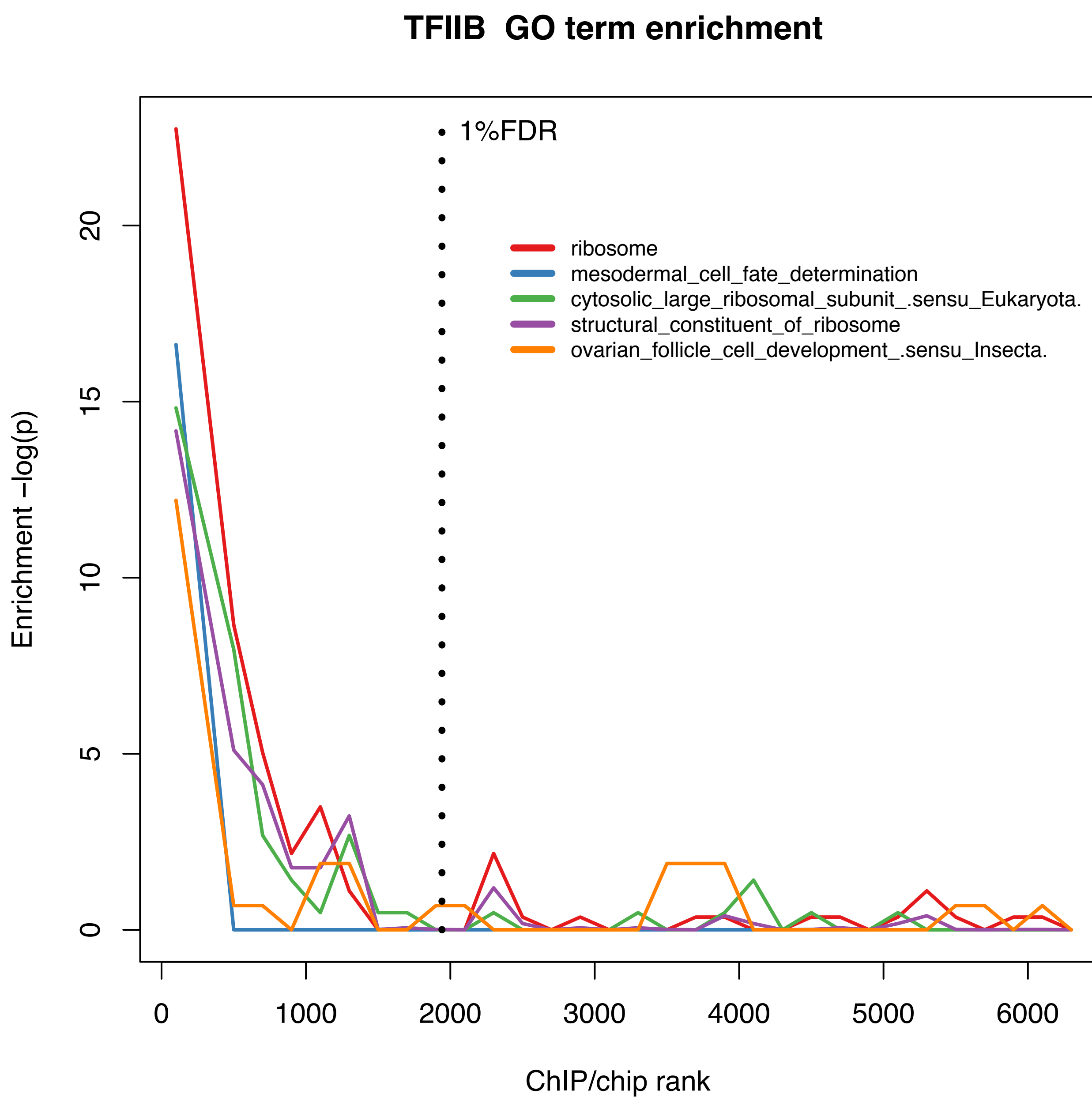
TFIIB GO term enrichment
1%FDR
ribosome
mesodermal_cell_fate_determination
cytosolic_large_ribosomal_subunit_.sensu_Eukaryota.
structural_constituent_of_ribosome
ovarian_follicle_cell_development_.sensu_Insecta.
Enrichment −log(p)
0
5
10
15
20
0
1000
2000
3000
4000
5000
6000
ChIP/chip rank
