# Supplementary material for: Developmental roles of 21 Drosophila transcription factors are determined by quantitative differences in binding to an overlapping set of thousands of genomic regions
Source: Genome Biol. 2009 Jul 23;10(7):R80. doi: 10.1186/gb-2009-10-7-r80 (PMC2728534; doi:10.1186/gb-2009-10-7-r80)
Supplement: Additional data file 10 — These are plotted down the ChIP/chip rank list in non-overlapping 200-peak cohorts. [file gb-2009-10-7-r80-S10.pdf]

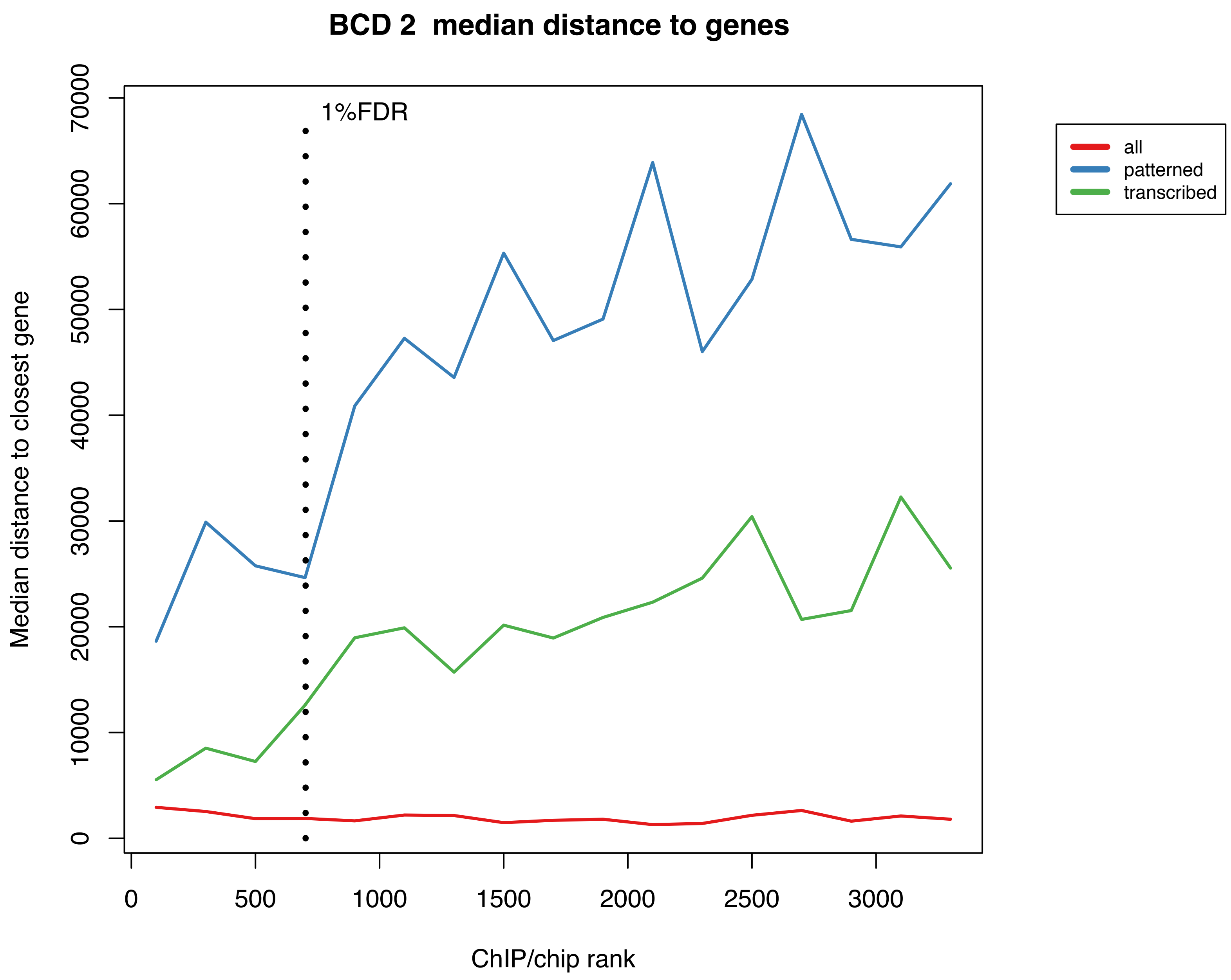
BCD 2 median distance to genes
1%FDR
all
patterned
transcribed
Median distance to closest gene
0
10000
20000
30000
40000
50000
60000
70000
0
500
1000
1500
2000
2500
3000
ChIP/chip rank

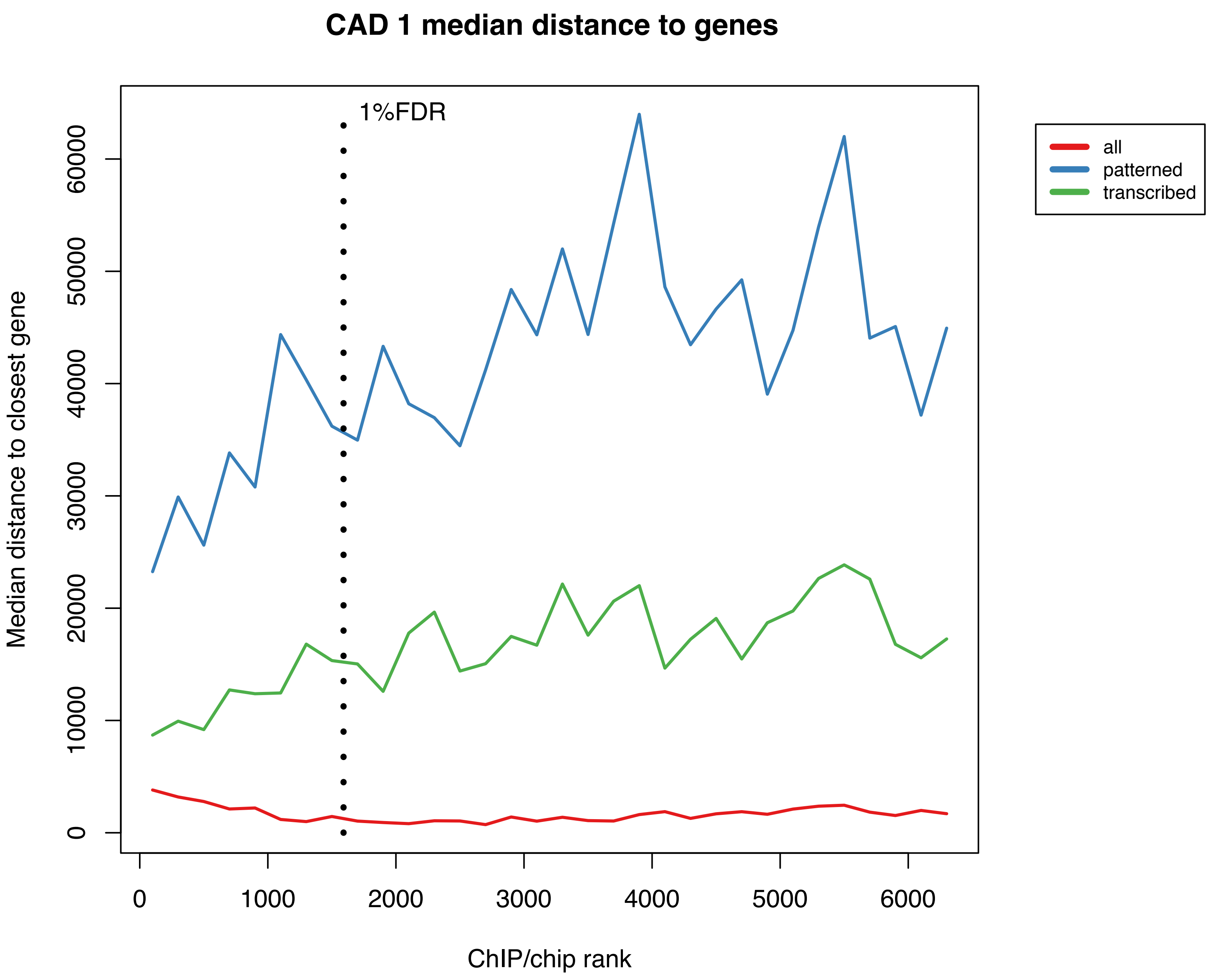
CAD 1 median distance to genes
1%FDR
all
patterned
transcribed
Median distance to closest gene
0
10000
20000
30000
40000
50000
60000
0
1000
2000
3000
4000
5000
6000
ChIP/chip rank

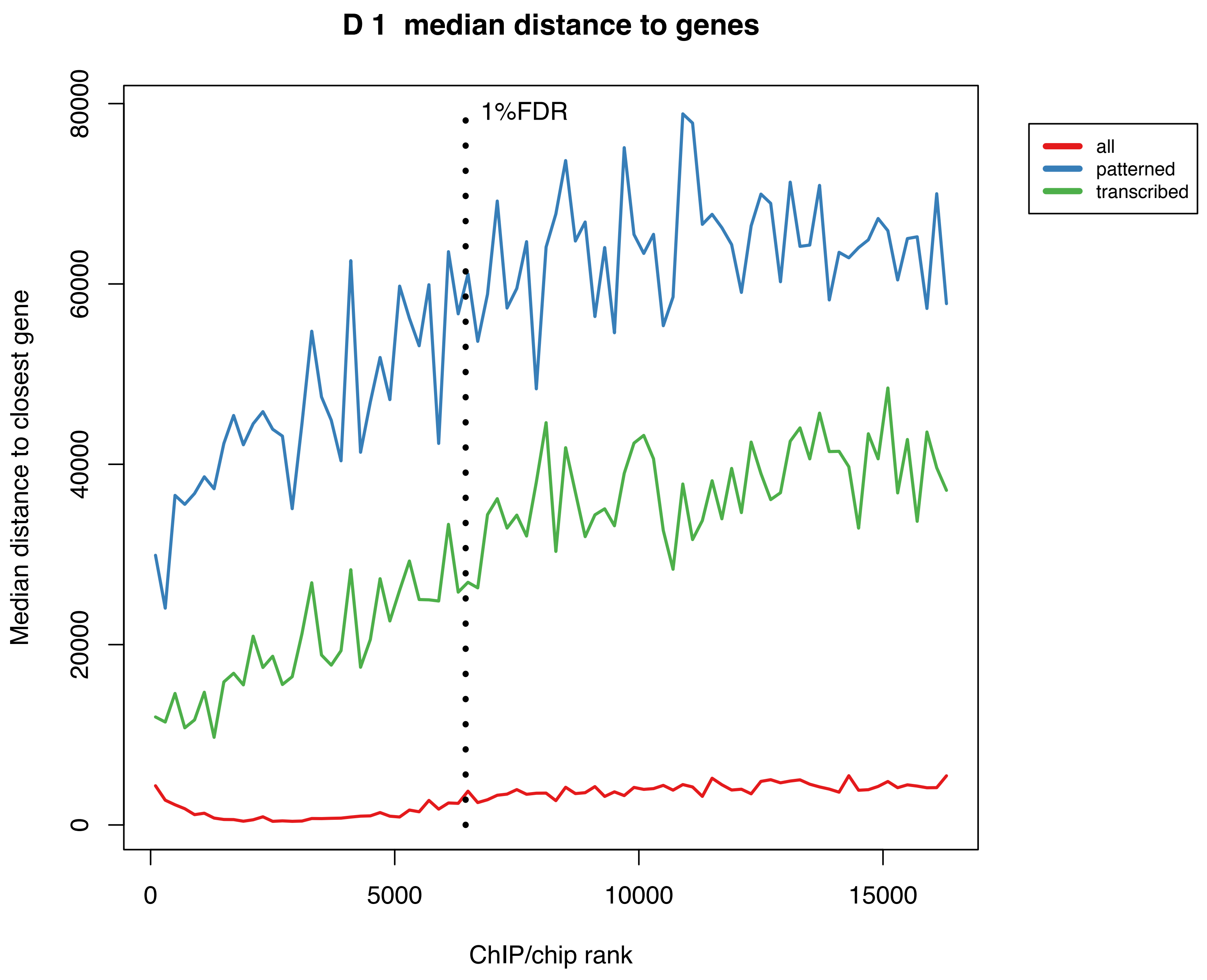
D 1 median distance to genes
Median distance to closest gene
0
20000
40000
60000
80000
0
5000
10000
15000
ChIP/chip rank
1%FDR
all
patterned
transcribed

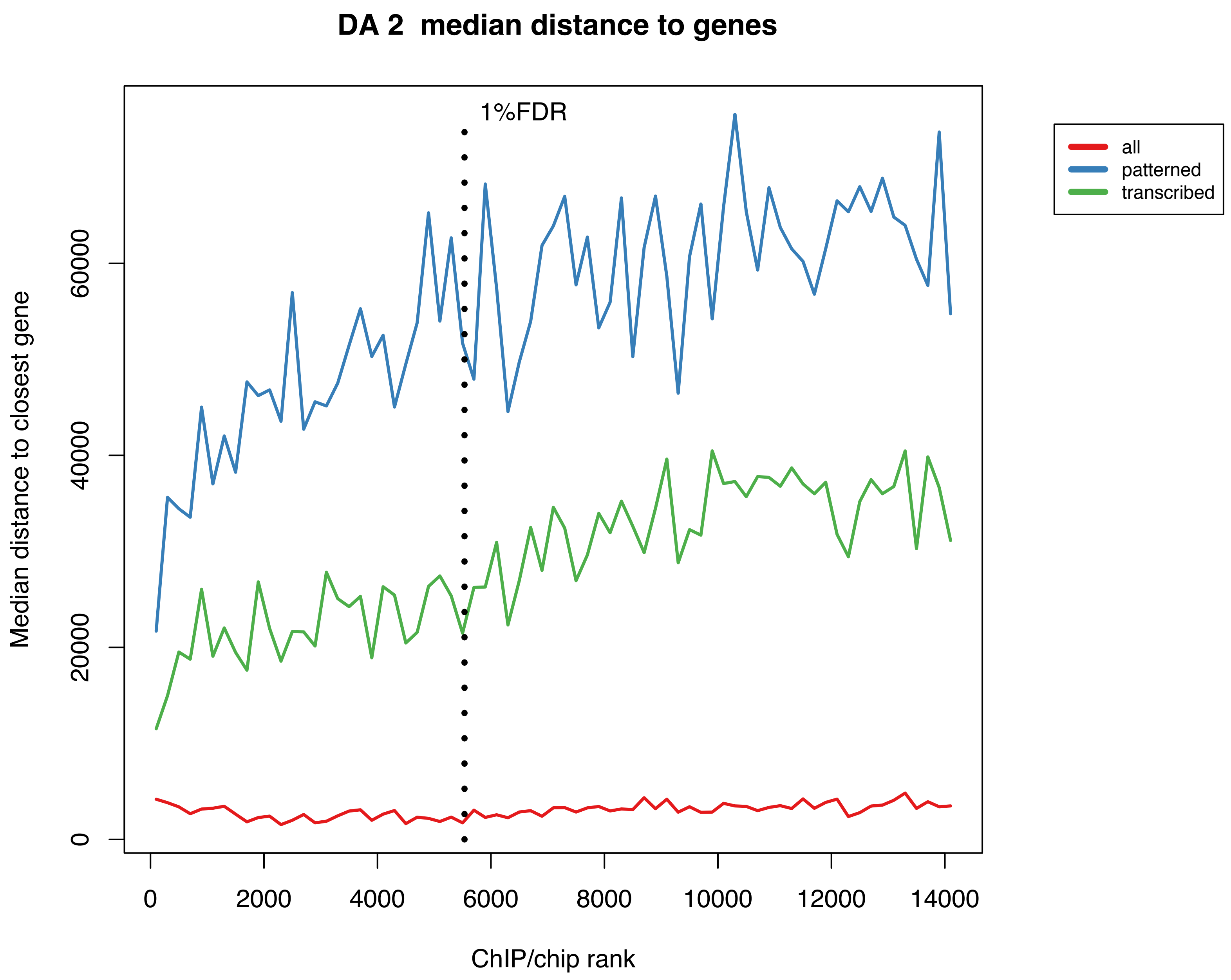
DA 2 median distance to genes
1%FDR
all
patterned
transcribed
Median distance to closest gene
0
20000
40000
60000
0
2000
4000
6000
8000
10000
12000
14000
ChIP/chip rank

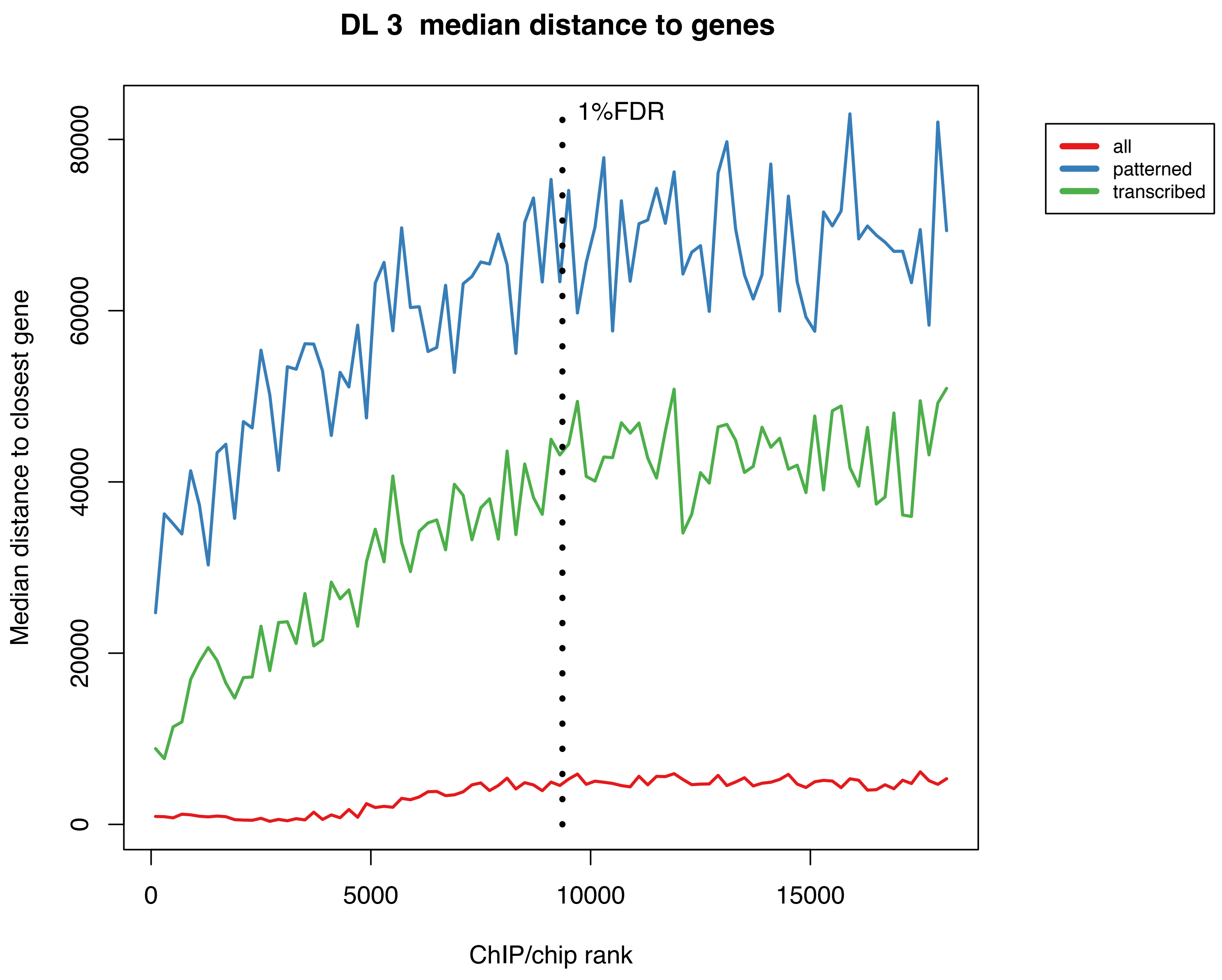
DL 3 median distance to genes
1%FDR
all
patterned
transcribed
Median distance to closest gene
0
20000
40000
60000
80000
0
5000
10000
15000
ChIP/chip rank

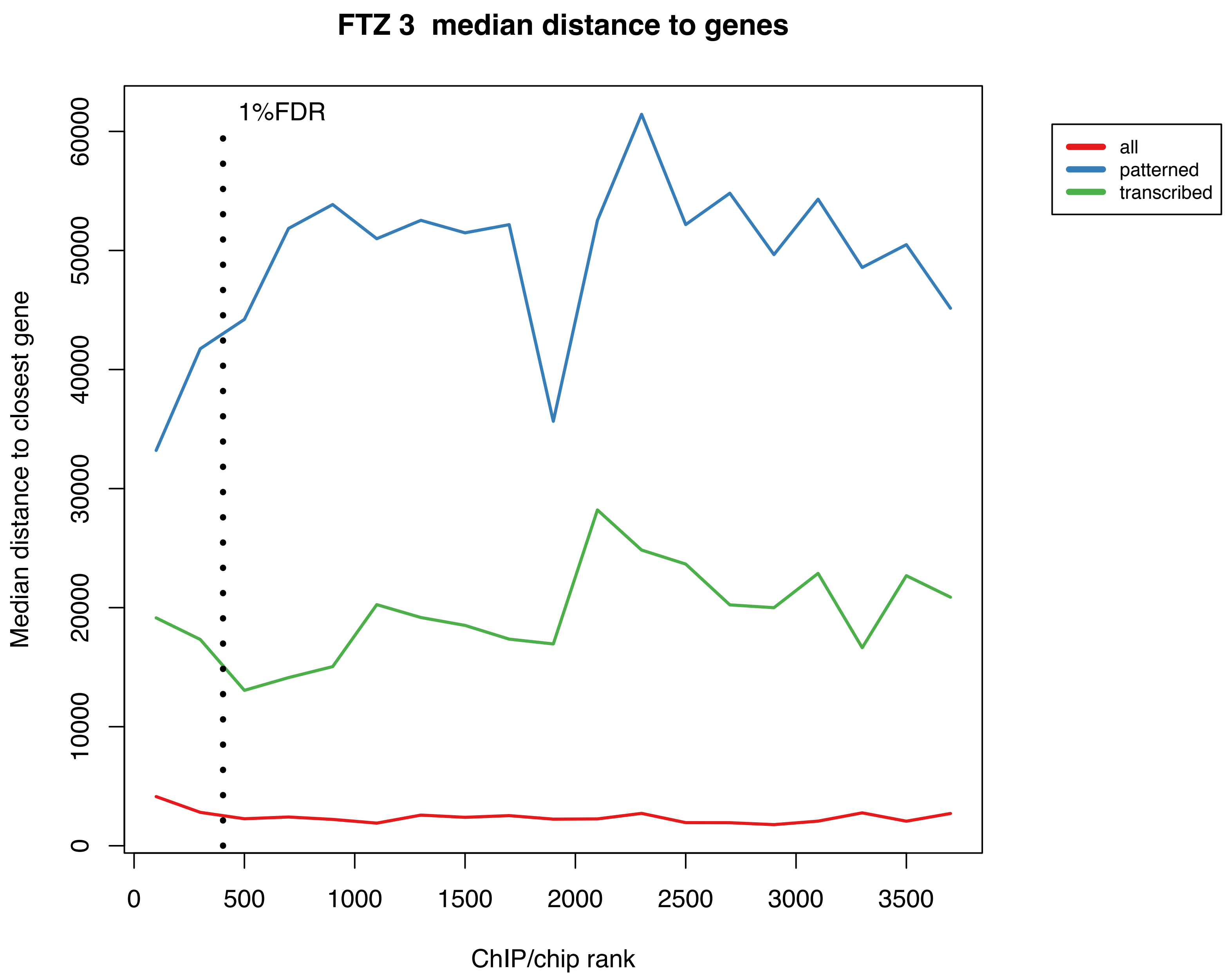
FTZ 3 median distance to genes
1%FDR
all
patterned
transcribed
Median distance to closest gene
ChIP/chip rank
0
10000
20000
30000
40000
50000
60000
0
500
1000
1500
2000
2500
3000
3500

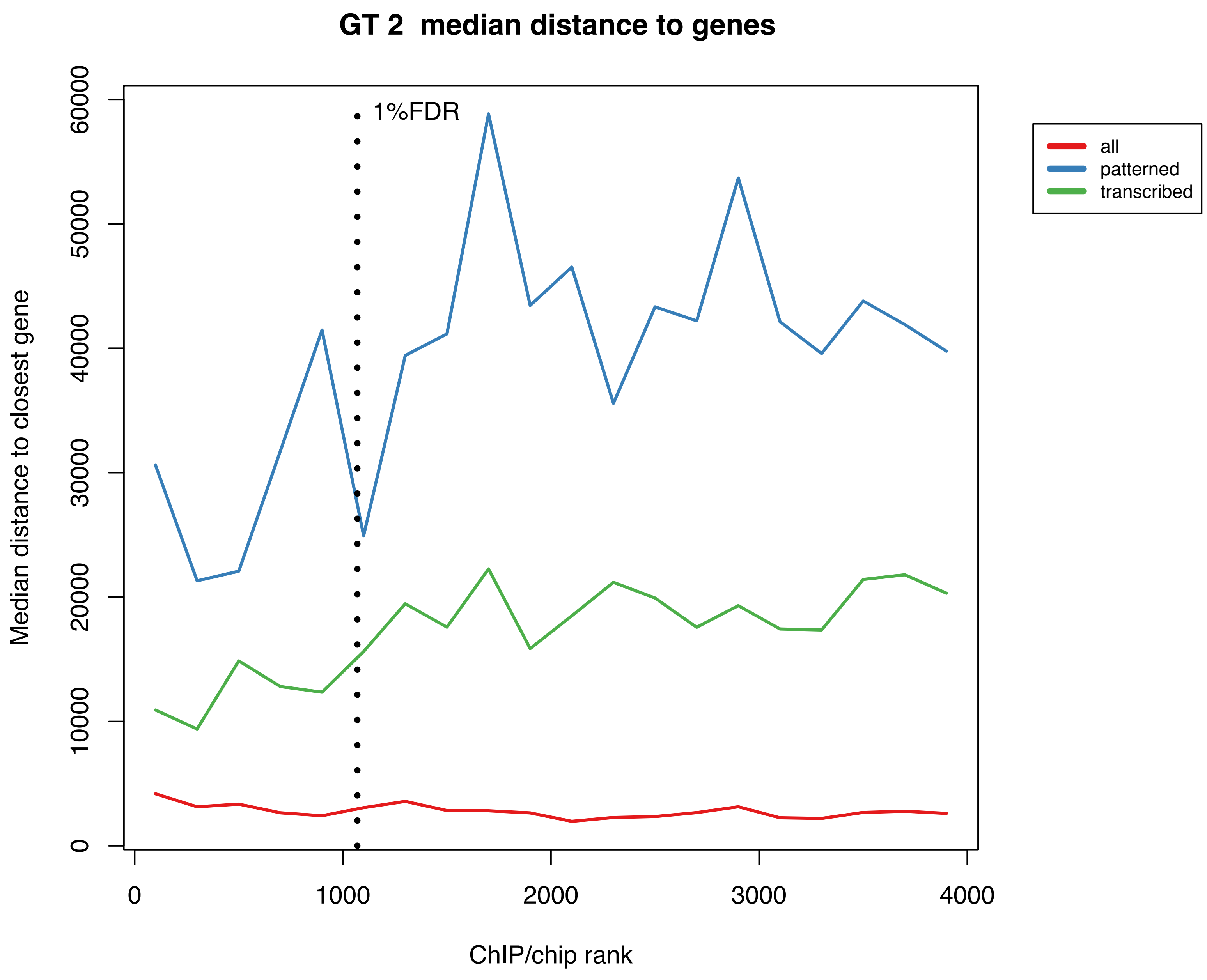
GT 2 median distance to genes
Median distance to closest gene
ChIP/chip rank
1%FDR
all
patterned
transcribed
0
10000
20000
30000
40000
50000
60000
0
1000
2000
3000
4000

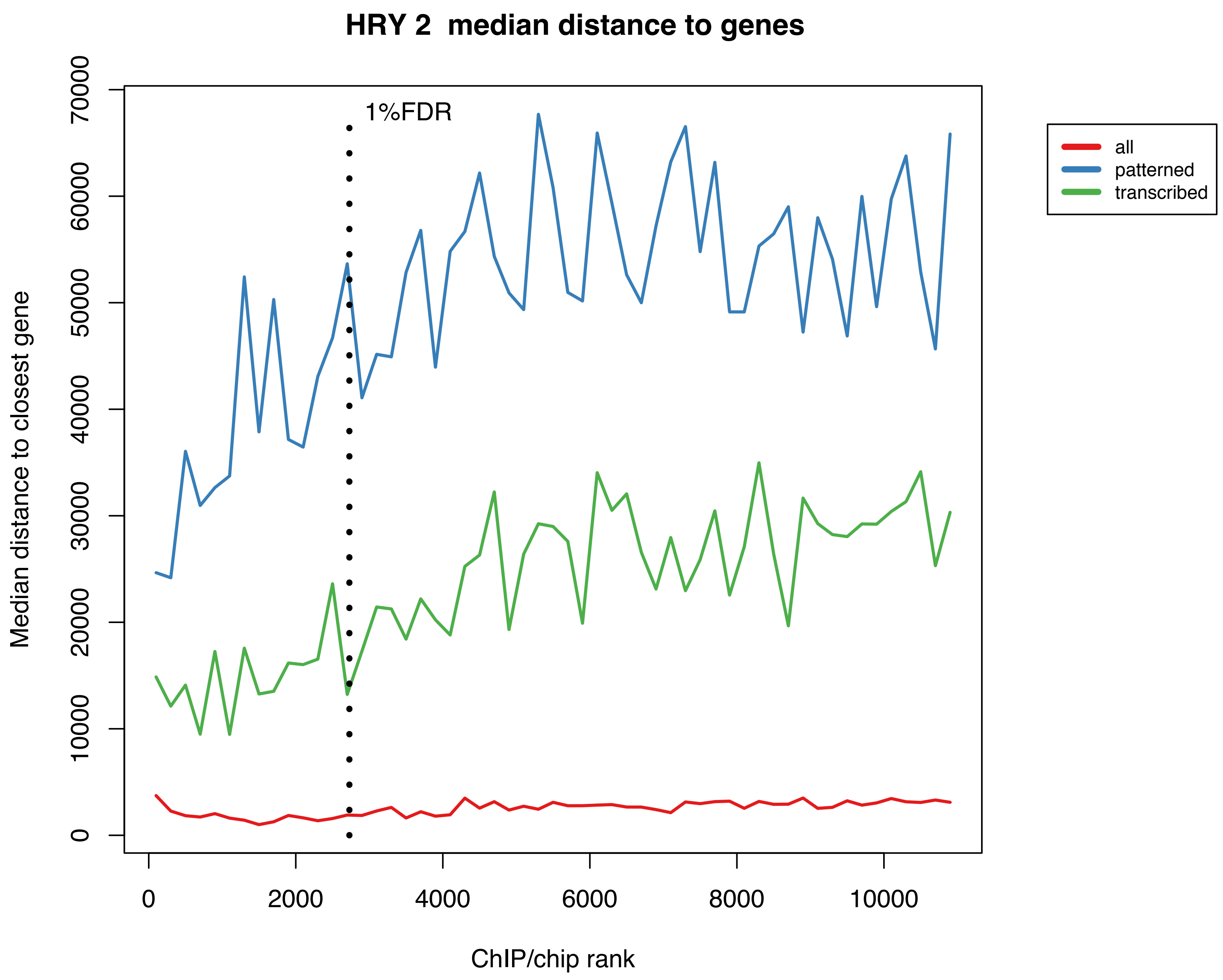
HRY 2 median distance to genes
1%FDR
all
patterned
transcribed
Median distance to closest gene
0
10000
20000
30000
40000
50000
60000
70000
0
2000
4000
6000
8000
10000
ChIP/chip rank

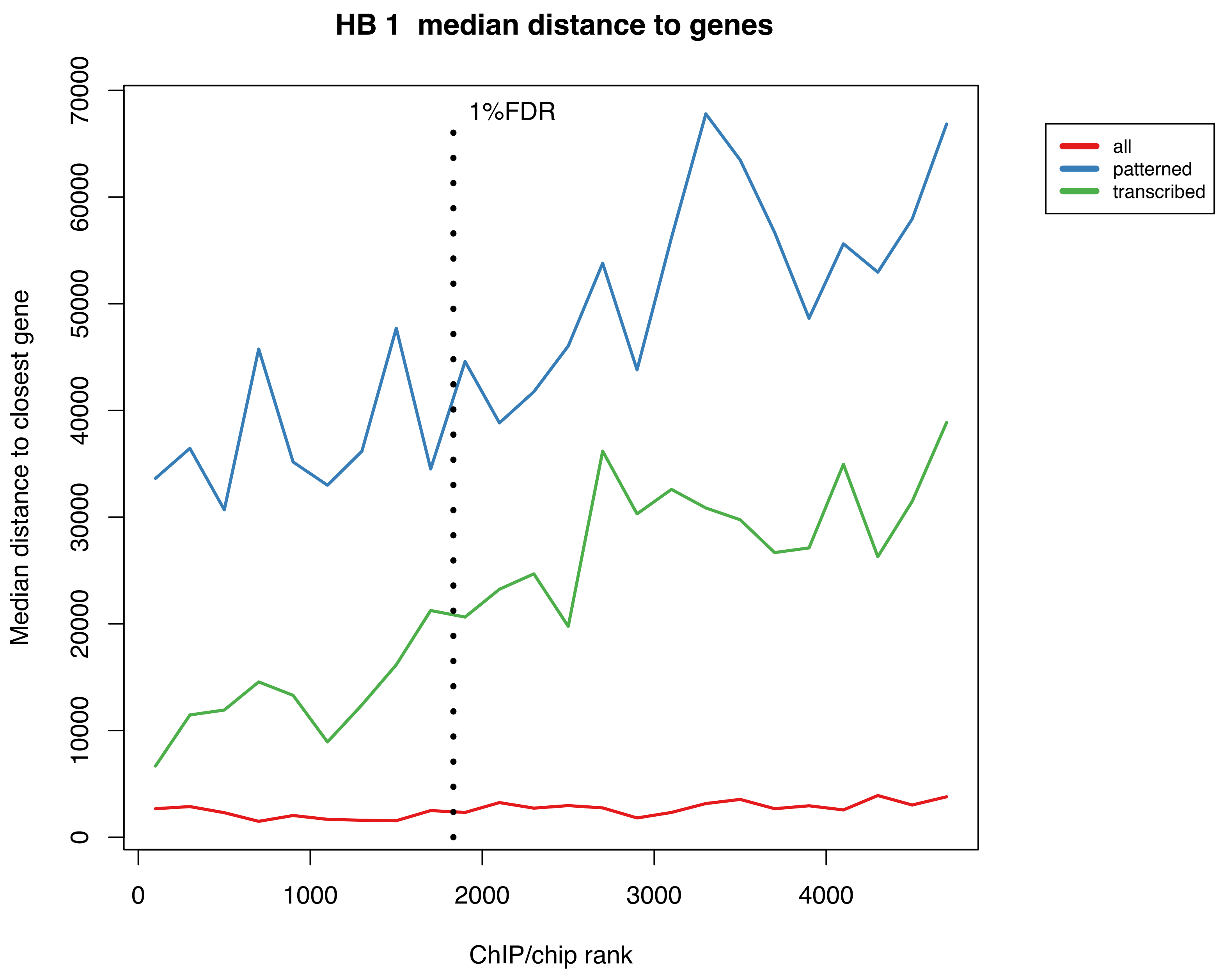
HB 1 median distance to genes
1%FDR
all
patterned
transcribed
Median distance to closest gene
0
10000
20000
30000
40000
50000
60000
70000
0
1000
2000
3000
4000
ChIP/chip rank

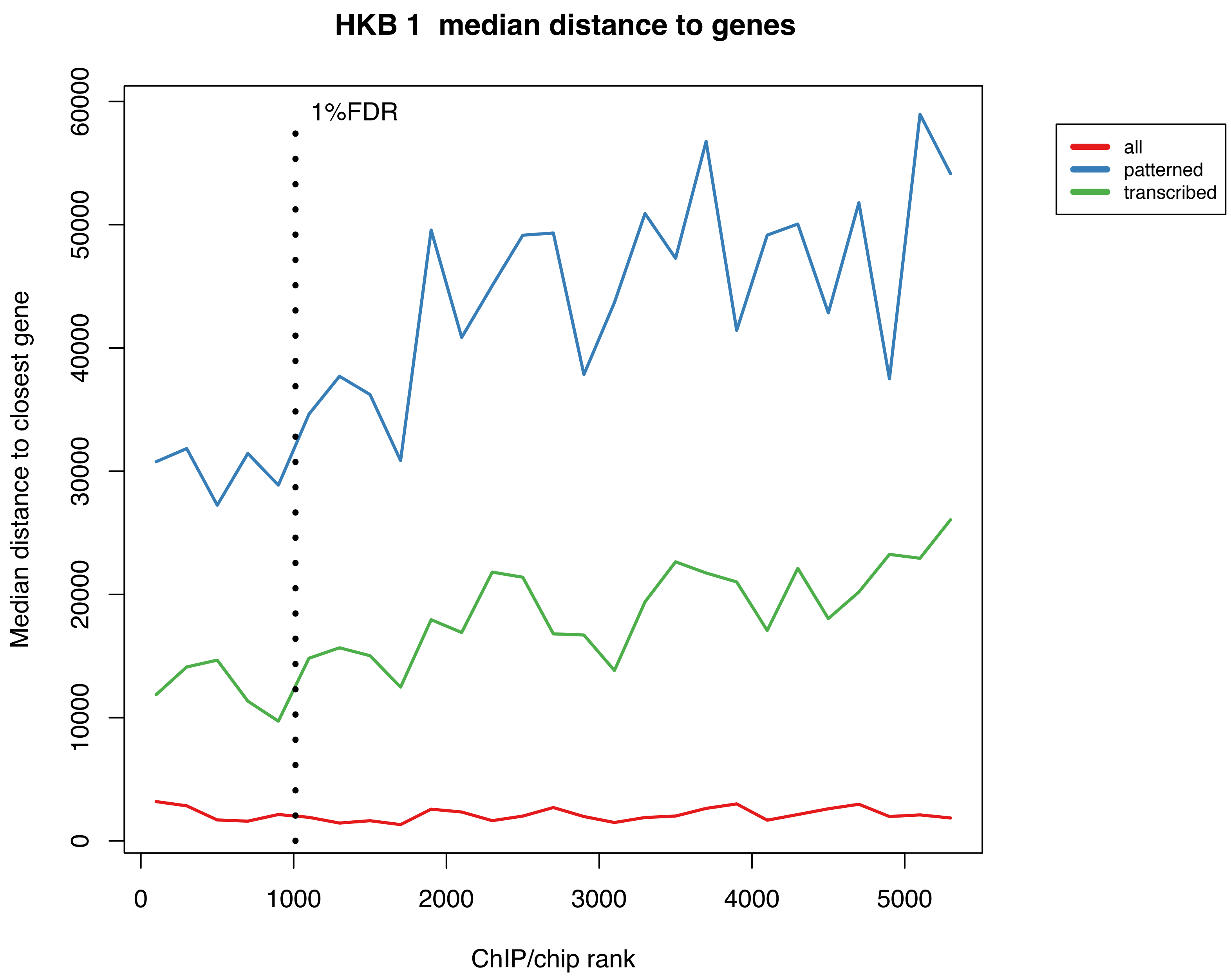
HKB 1 median distance to genes
1%FDR
Median distance to closest gene
0
10000
20000
30000
40000
50000
60000
0
1000
2000
3000
4000
5000
ChIP/chip rank
all
patterned
transcribed

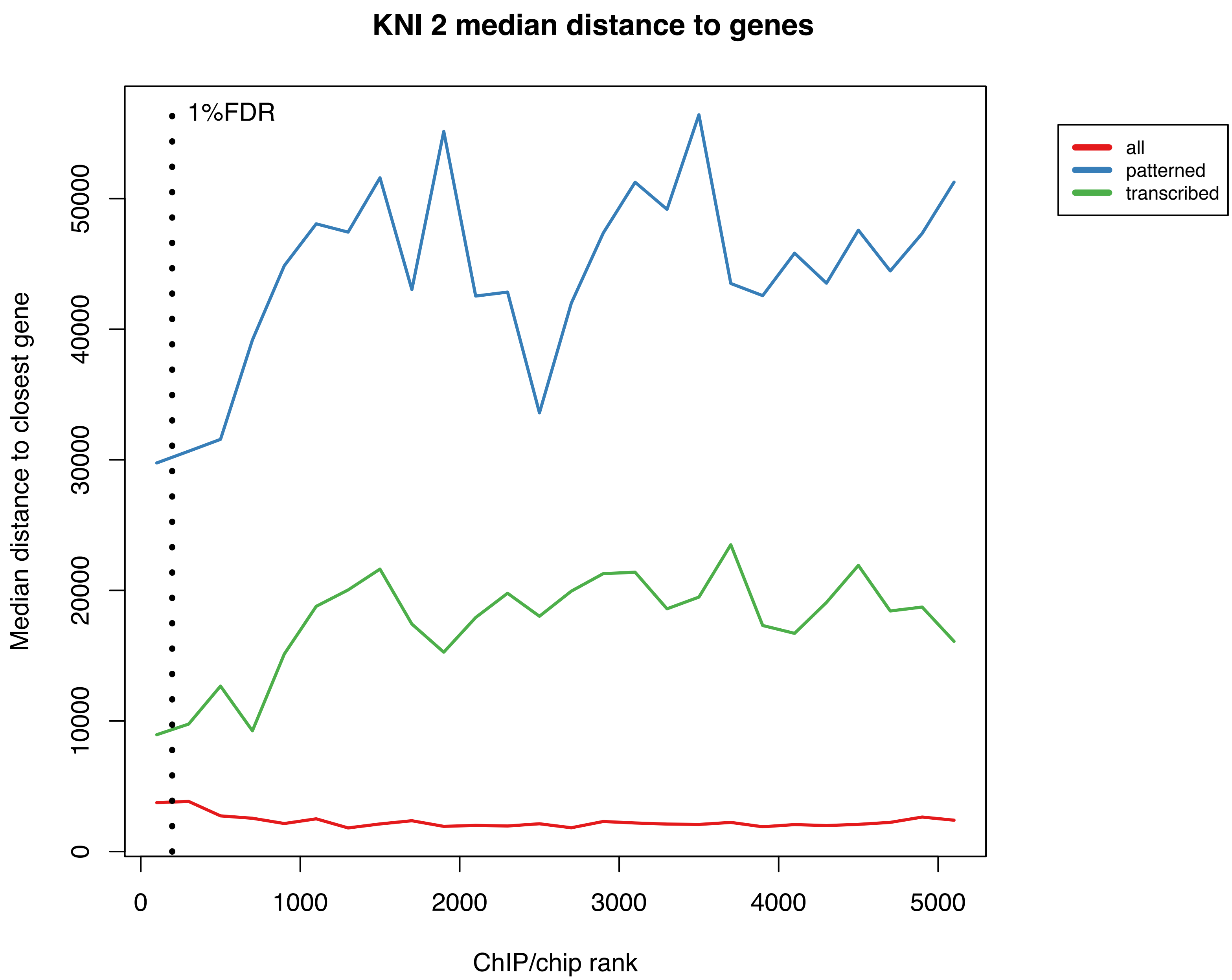
KNI 2 median distance to genes
1%FDR
all
patterned
transcribed
Median distance to closest gene
0
10000
20000
30000
40000
50000
0
1000
2000
3000
4000
5000
ChIP/chip rank

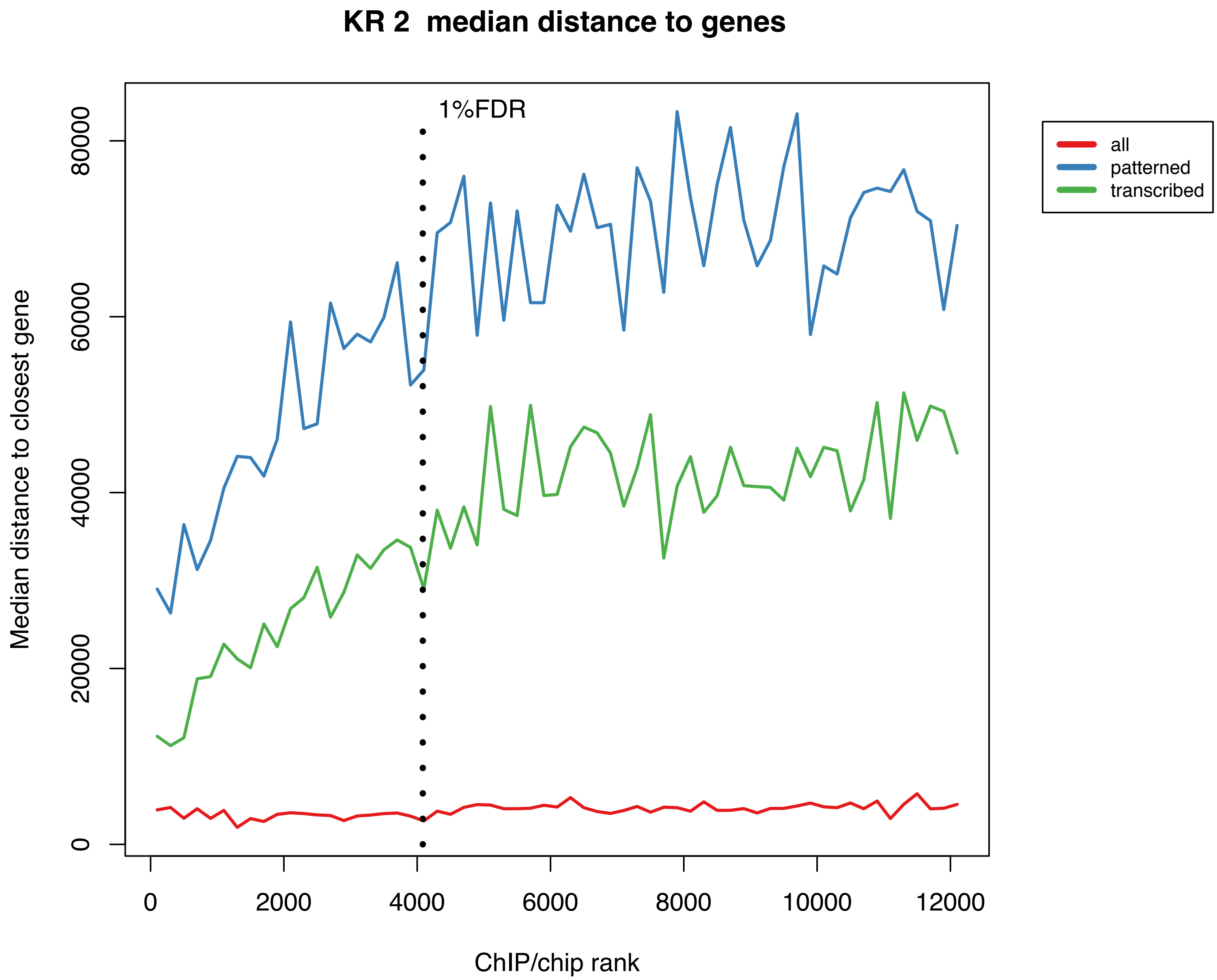
KR 2 median distance to genes
1%FDR
Median distance to closest gene
0
20000
40000
60000
80000
0
2000
4000
6000
8000
10000
12000
ChIP/chip rank
all
patterned
transcribed

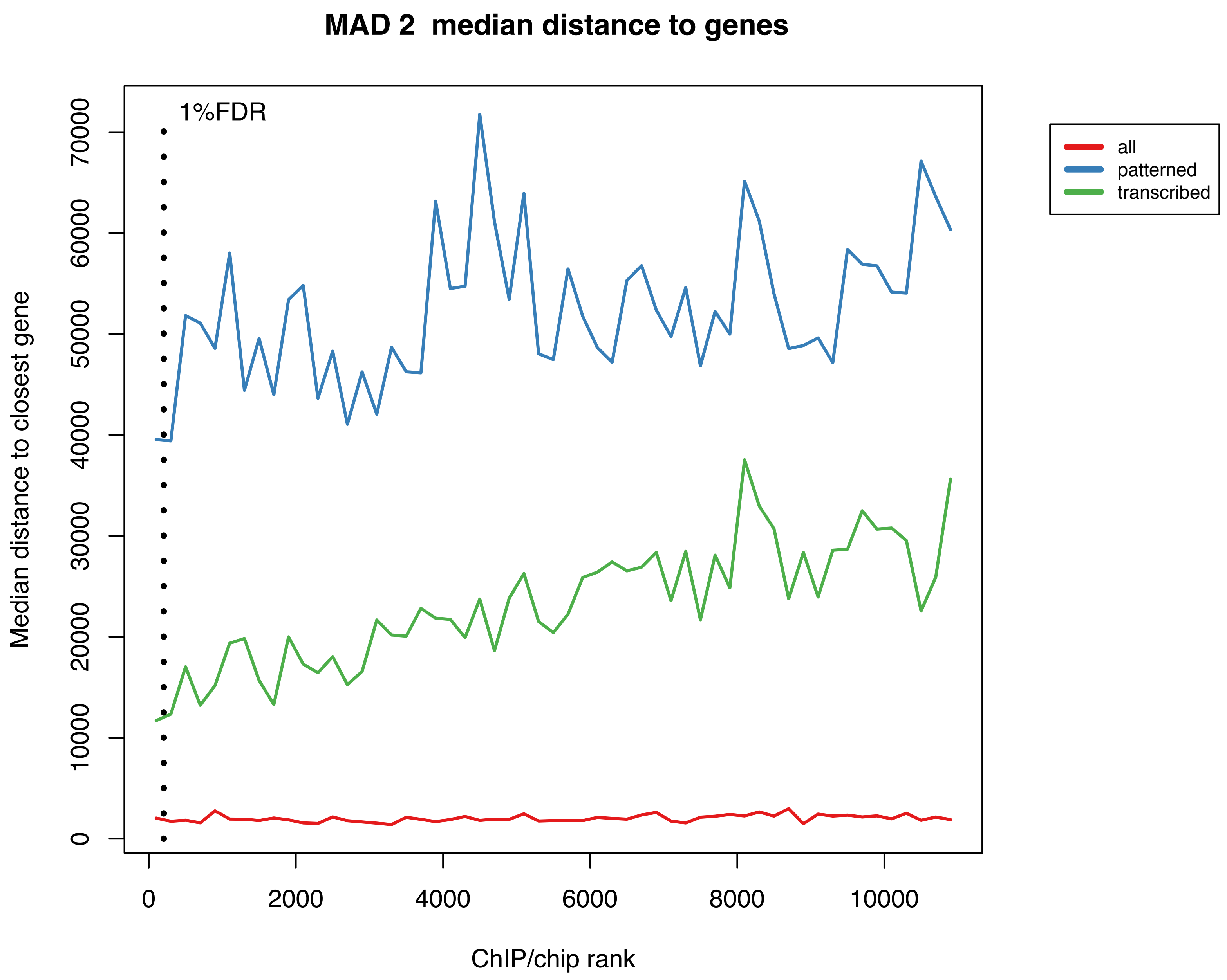
MAD 2 median distance to genes
1%FDR
all
patterned
transcribed
Median distance to closest gene
0
10000
20000
30000
40000
50000
60000
70000
ChIP/chip rank
0
2000
4000
6000
8000
10000

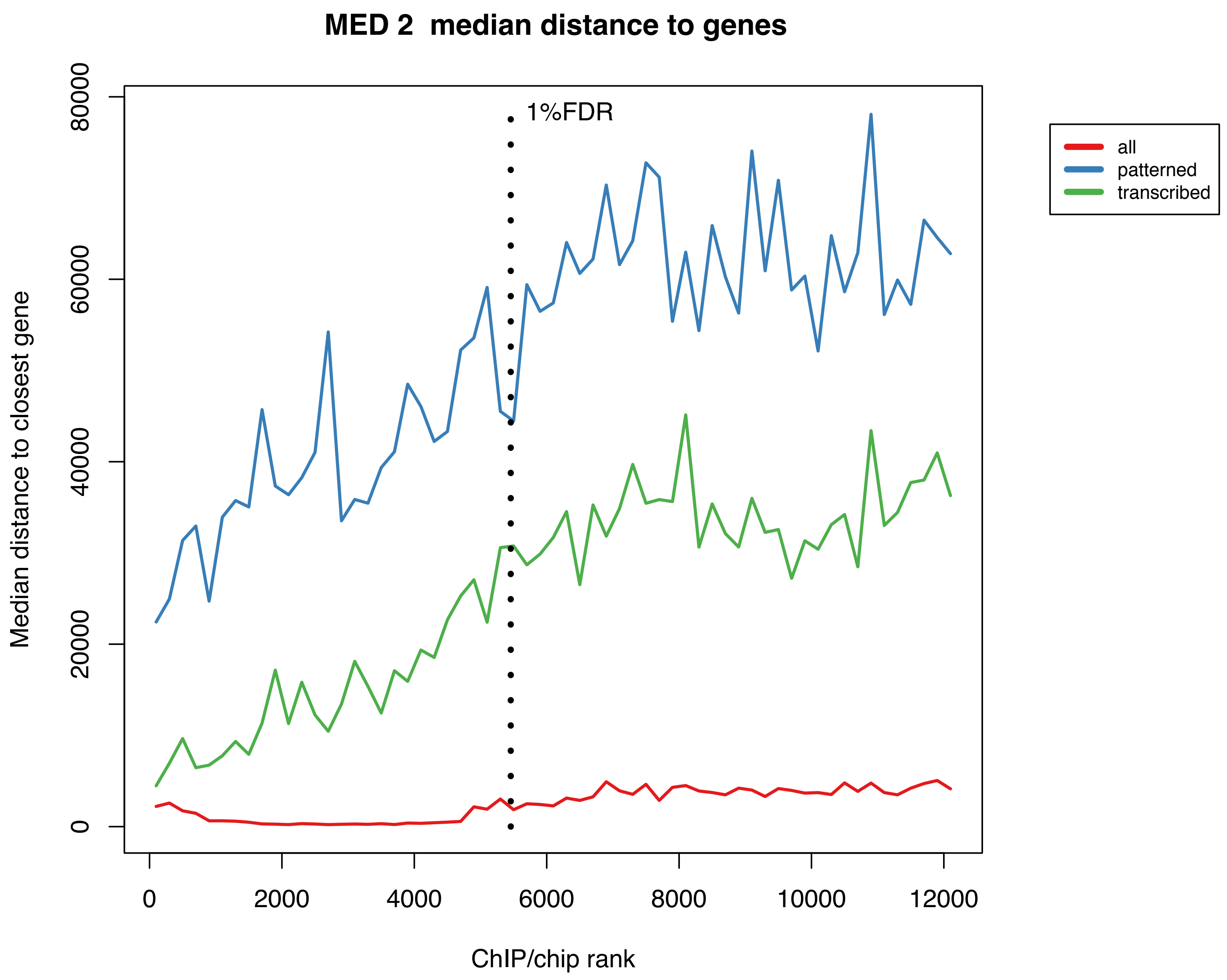
MED 2 median distance to genes
Median distance to closest gene
0
20000
40000
60000
80000
ChIP/chip rank
0
2000
4000
6000
8000
10000
12000
1%FDR
all
patterned
transcribed

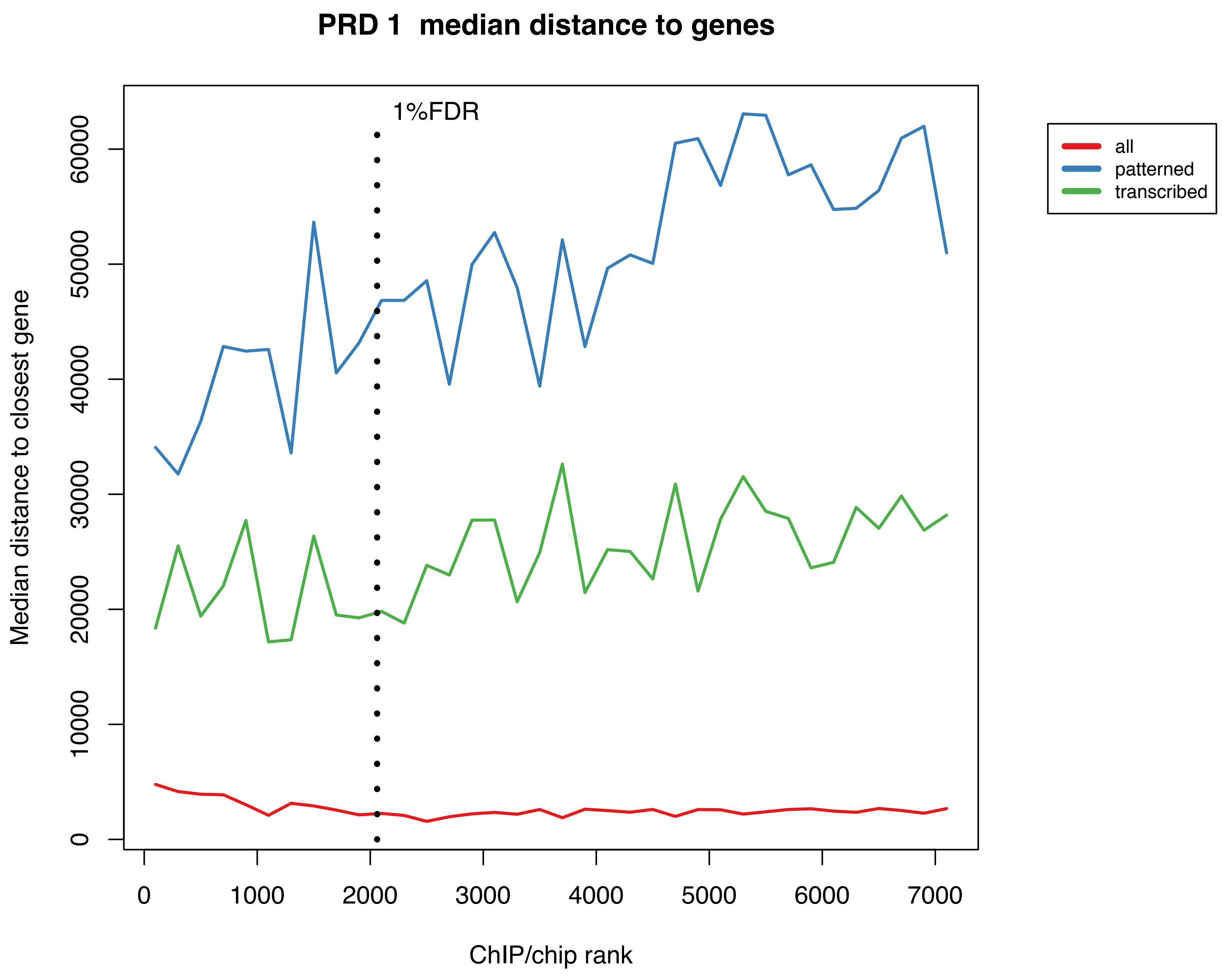
PRD 1 median distance to genes
1%FDR
all
patterned
transcribed
Median distance to closest gene
0
10000
20000
30000
40000
50000
60000
0
1000
2000
3000
4000
5000
6000
7000
ChIP/chip rank

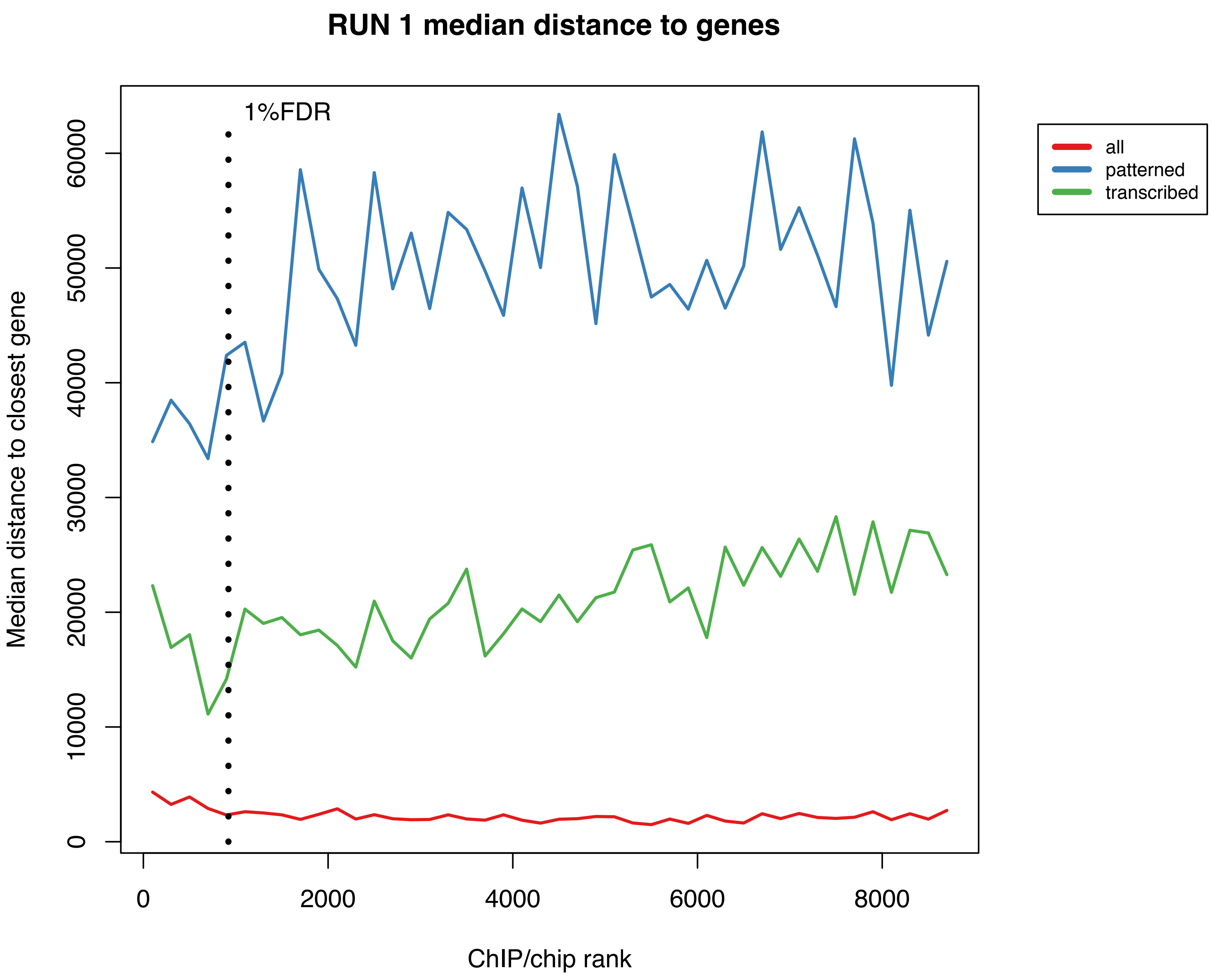
RUN 1 median distance to genes
1%FDR
Median distance to closest gene
0
10000
20000
30000
40000
50000
60000
0
2000
4000
6000
8000
ChIP/chip rank
all
patterned
transcribed

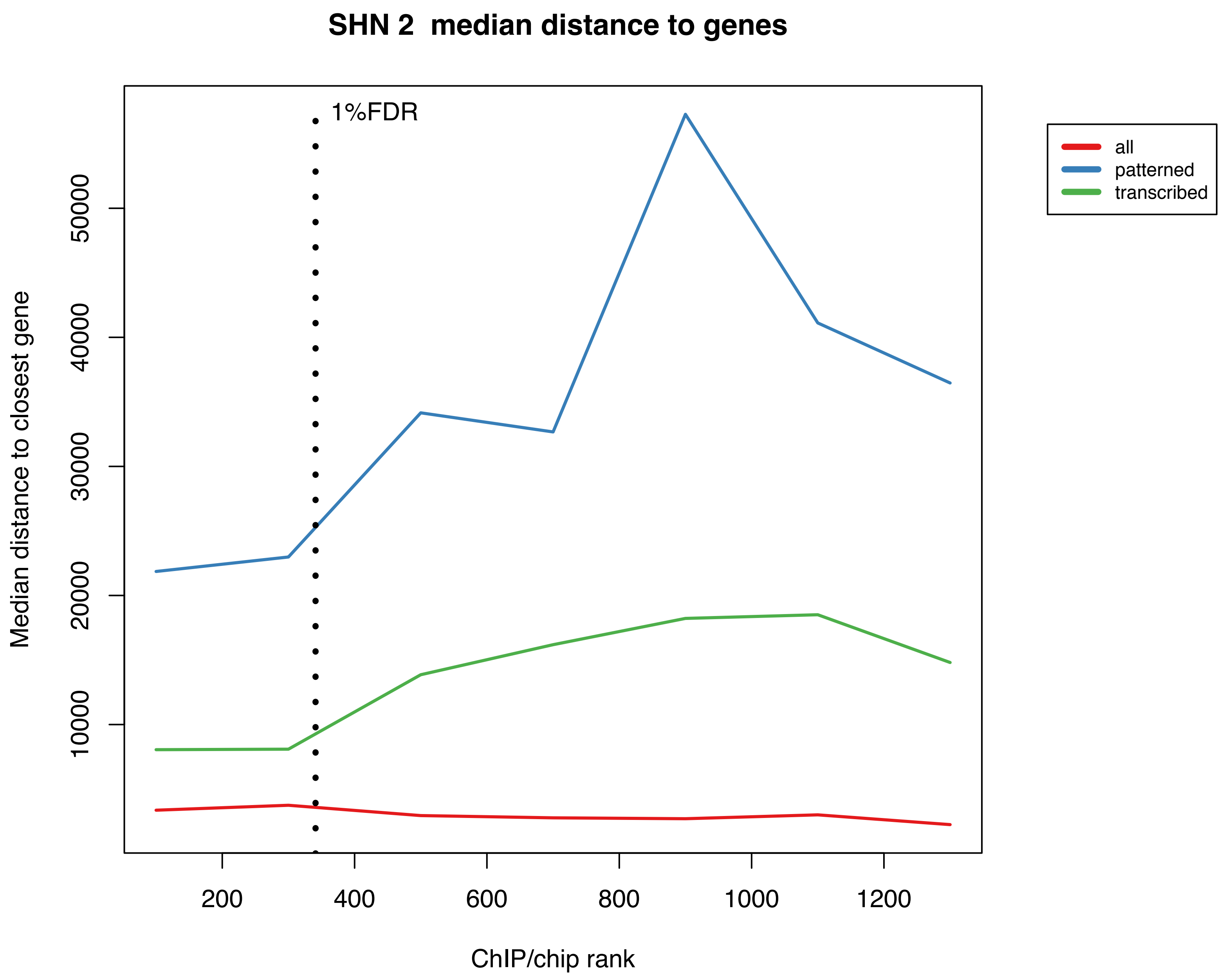
SHN 2 median distance to genes
1%FDR
all
patterned
transcribed
Median distance to closest gene
10000
20000
30000
40000
50000
200
400
600
800
1000
1200
ChIP/chip rank

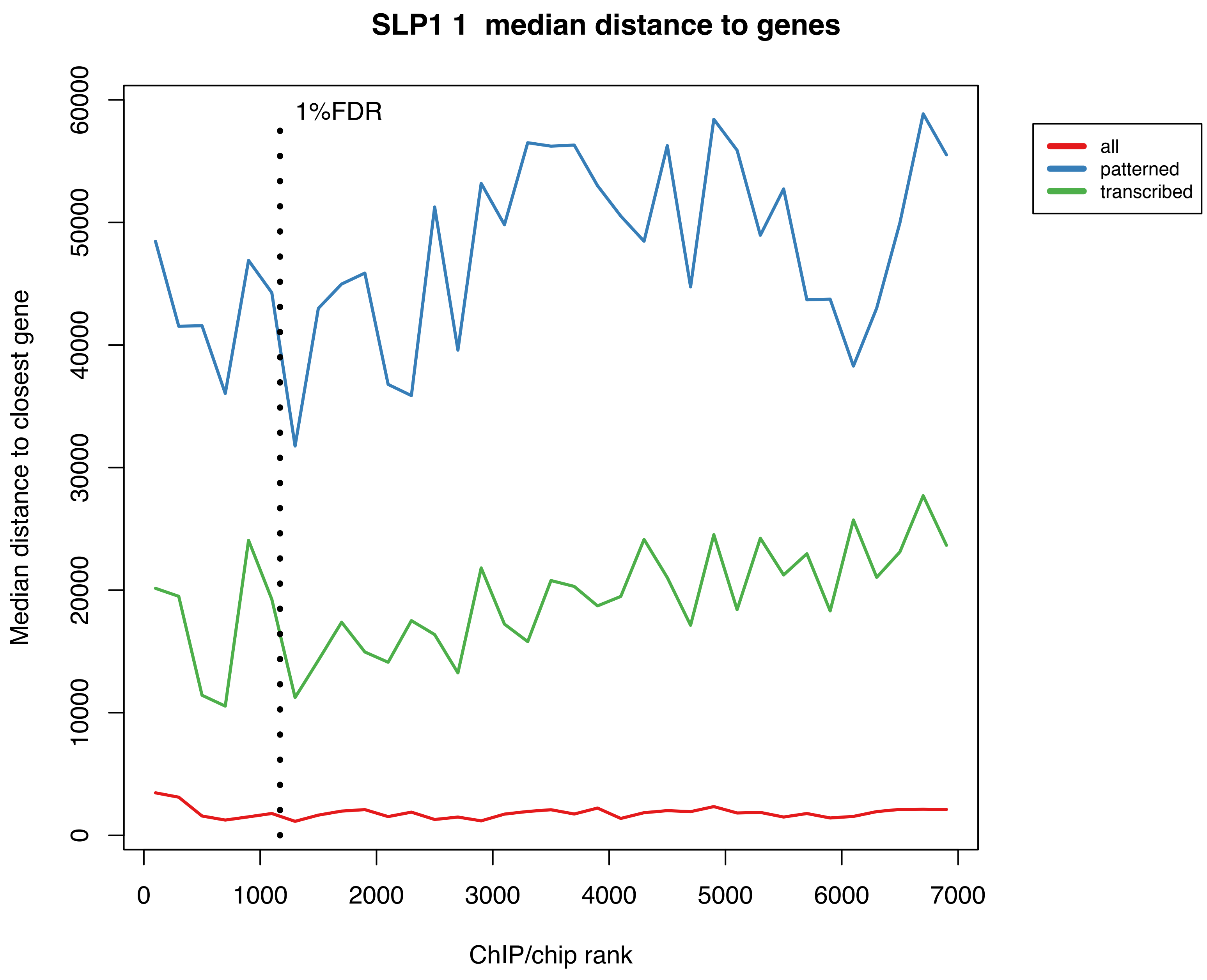
SLP1 1 median distance to genes
1%FDR
Median distance to closest gene
ChIP/chip rank
0
10000
20000
30000
40000
50000
60000
0
1000
2000
3000
4000
5000
6000
7000
all
patterned
transcribed

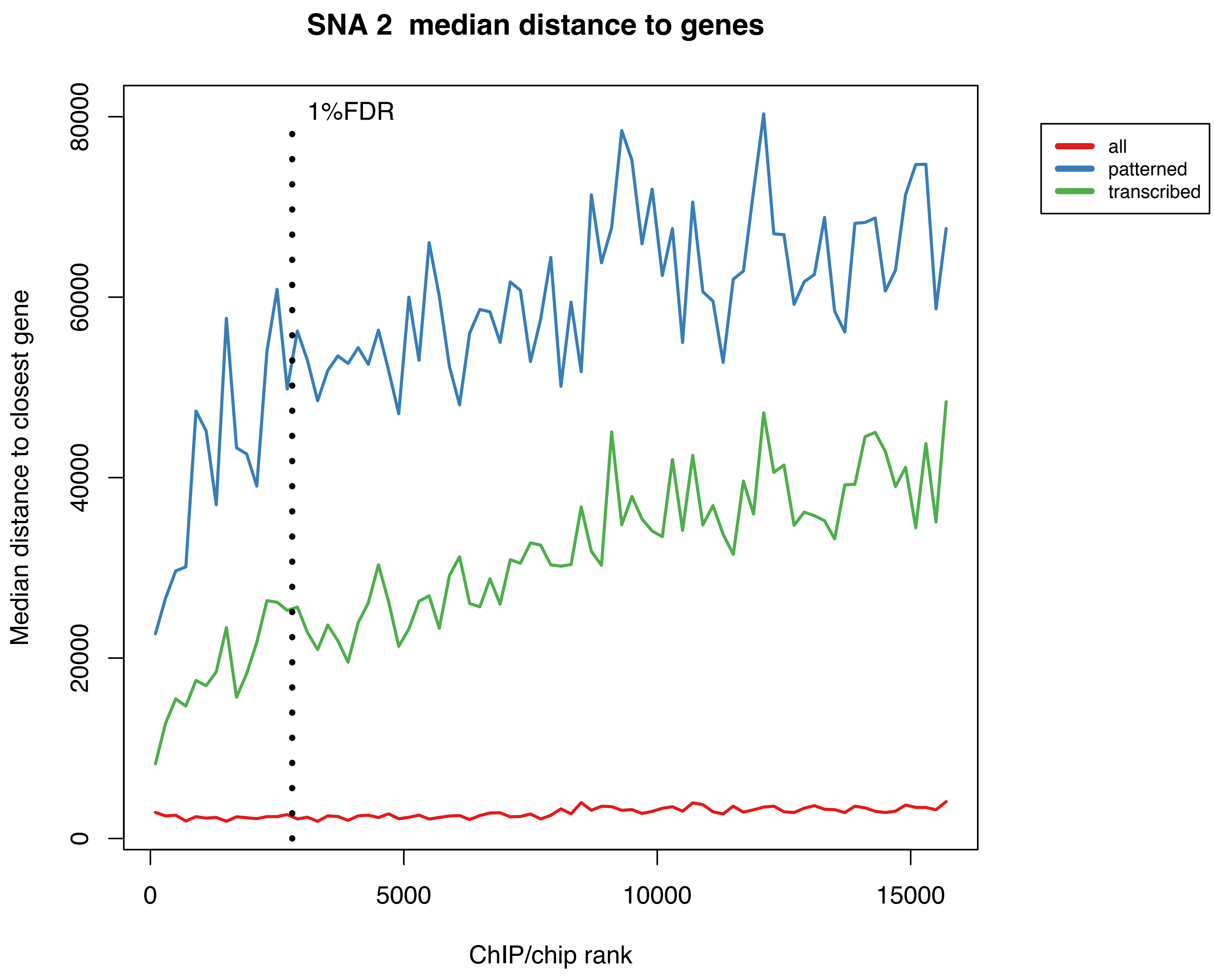
SNA 2 median distance to genes
1%FDR
Median distance to closest gene
0
20000
40000
60000
80000
0
5000
10000
15000
ChIP/chip rank
all
patterned
transcribed

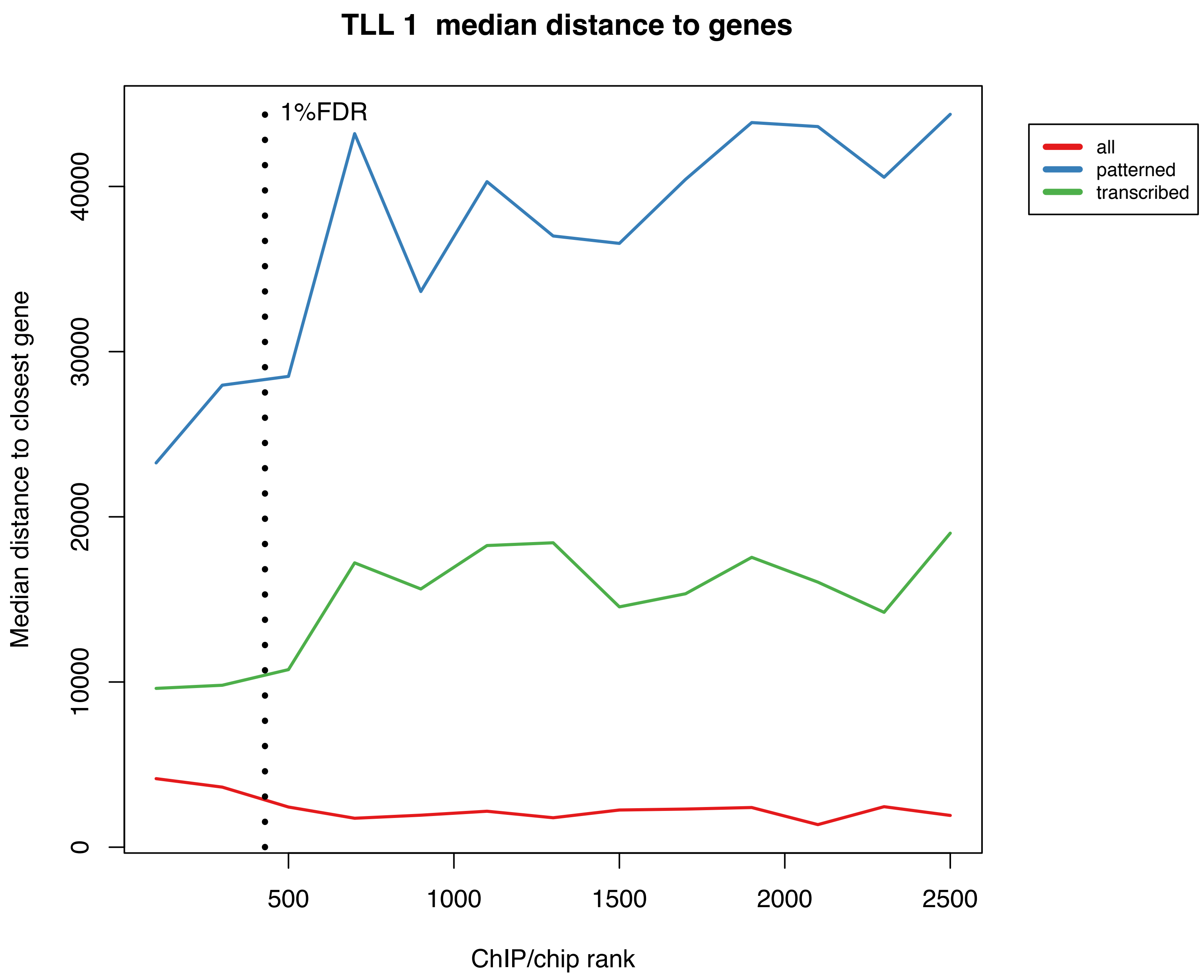
TLL 1 median distance to genes
1%FDR
all
patterned
transcribed
Median distance to closest gene
0
10000
20000
30000
40000
500
1000
1500
2000
2500
ChIP/chip rank

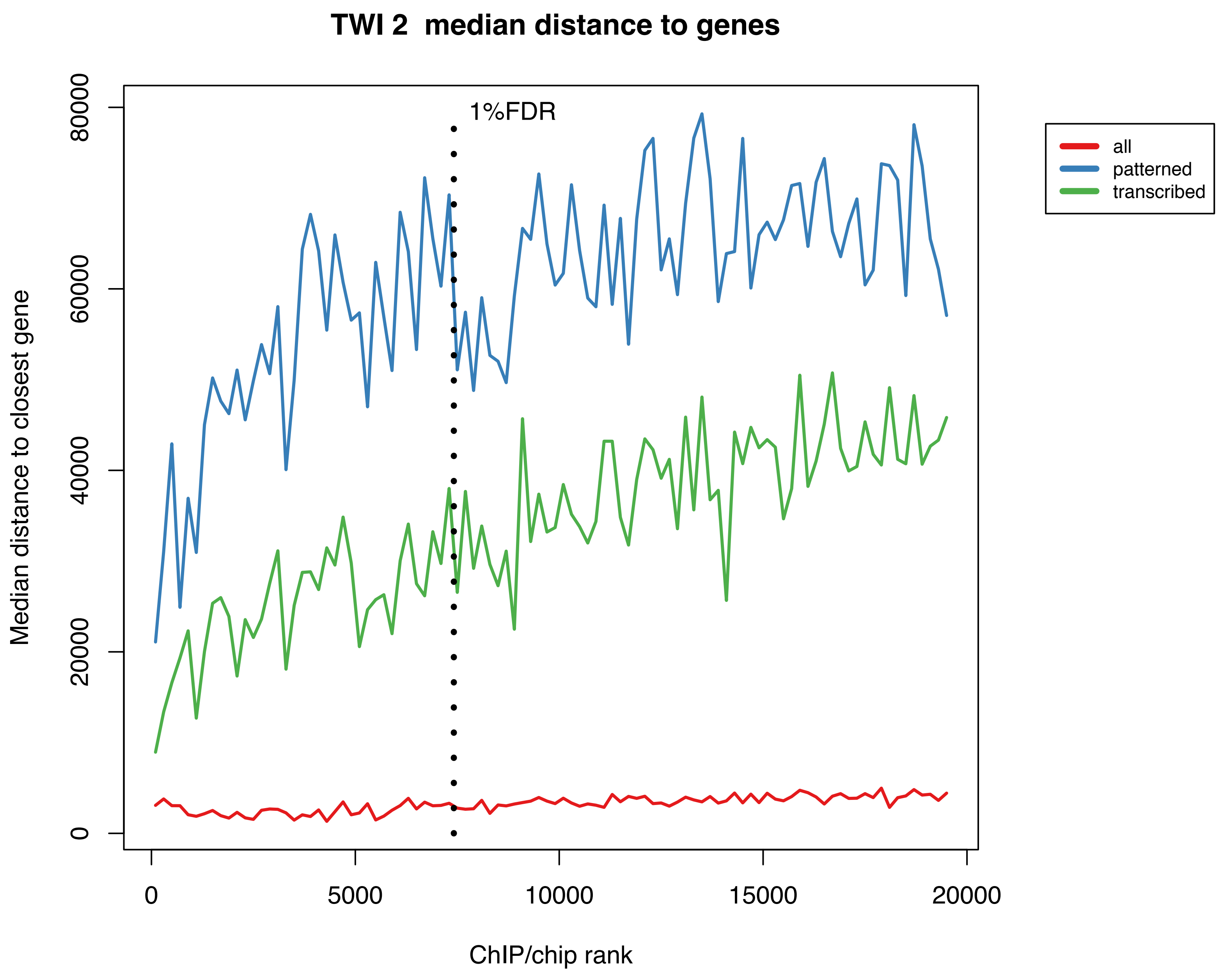
TWI 2 median distance to genes
Median distance to closest gene
0
20000
40000
60000
80000
1%FDR
0
5000
10000
15000
20000
ChIP/chip rank
all
patterned
transcribed

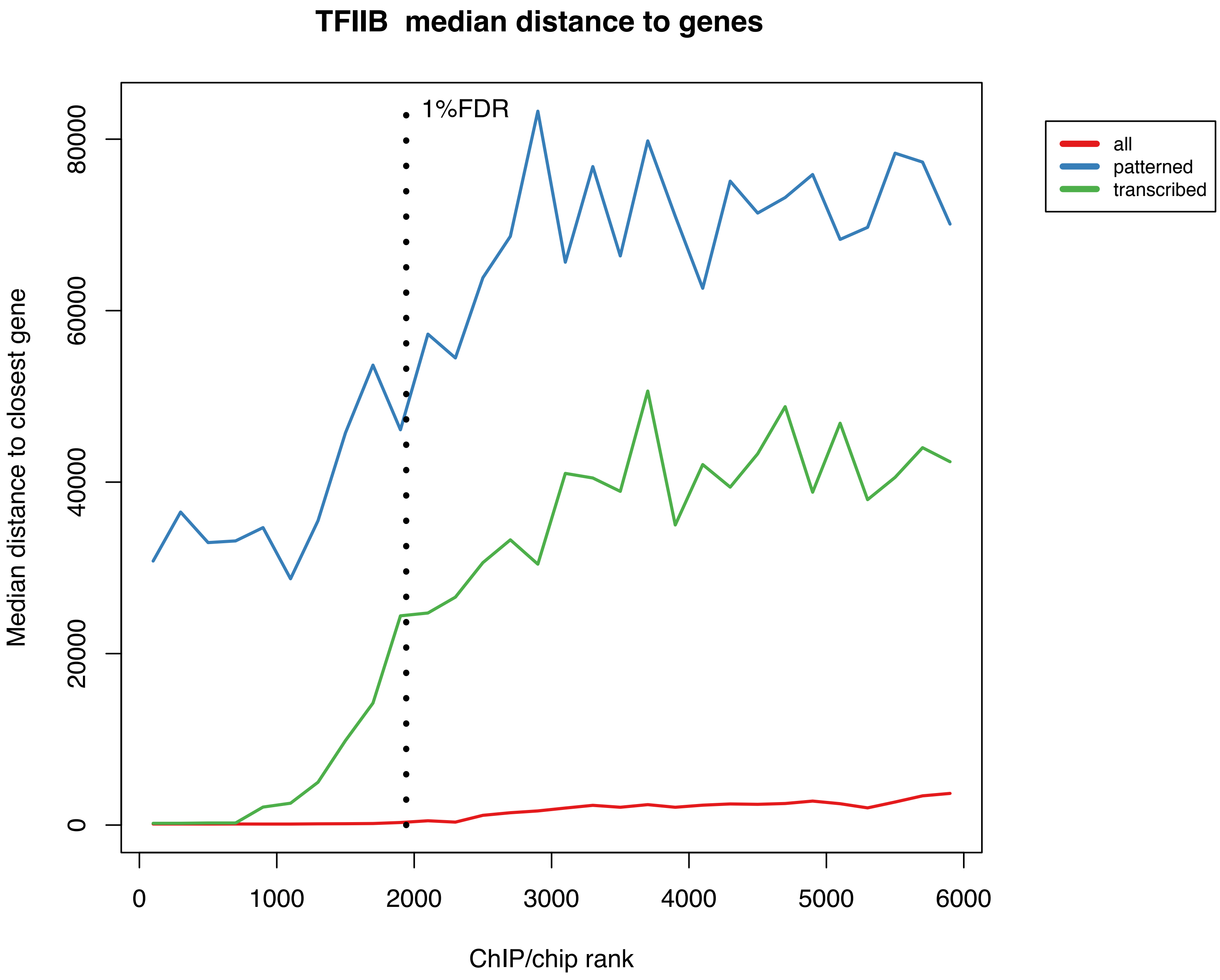
TFIIB median distance to genes
1%FDR
all
patterned
transcribed
Median distance to closest gene
0
20000
40000
60000
80000
0
1000
2000
3000
4000
5000
6000
ChIP/chip rank
