# Supplementary material for: Developmental roles of 21 Drosophila transcription factors are determined by quantitative differences in binding to an overlapping set of thousands of genomic regions
Source: Genome Biol. 2009 Jul 23;10(7):R80. doi: 10.1186/gb-2009-10-7-r80 (PMC2728534; doi:10.1186/gb-2009-10-7-r80)
Supplement: Additional data file 11 — These are plotted down the ChIP/chip rank list in non-overlapping 200-peak cohorts. [file gb-2009-10-7-r80-S11.pdf]

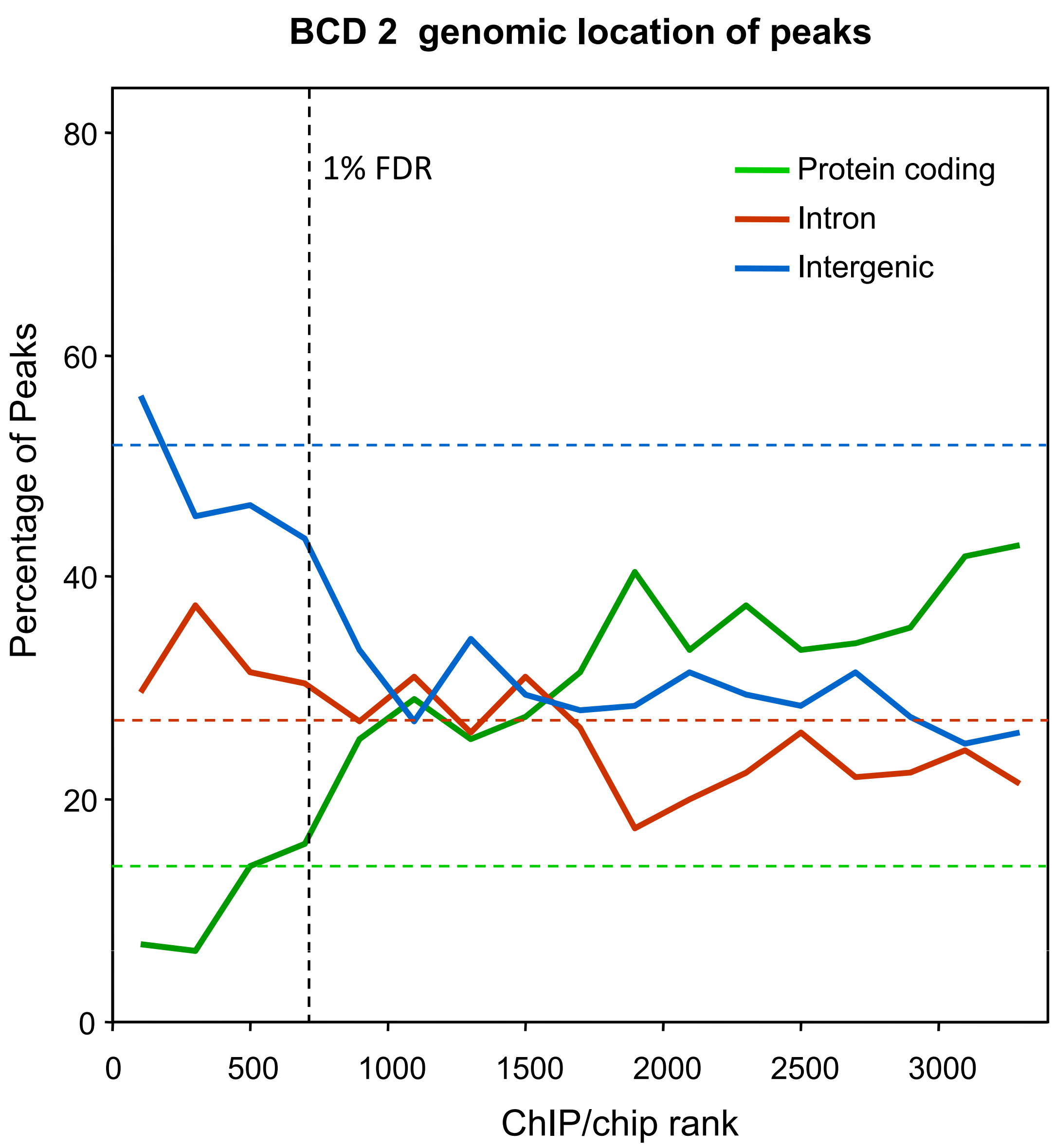
BCD 2 genomic location of peaks
1% FDR
Protein coding
Intron
Intergenic
Percentage of Peaks
0
20
40
60
80
0
500
1000
1500
2000
2500
3000
ChIP/chip rank

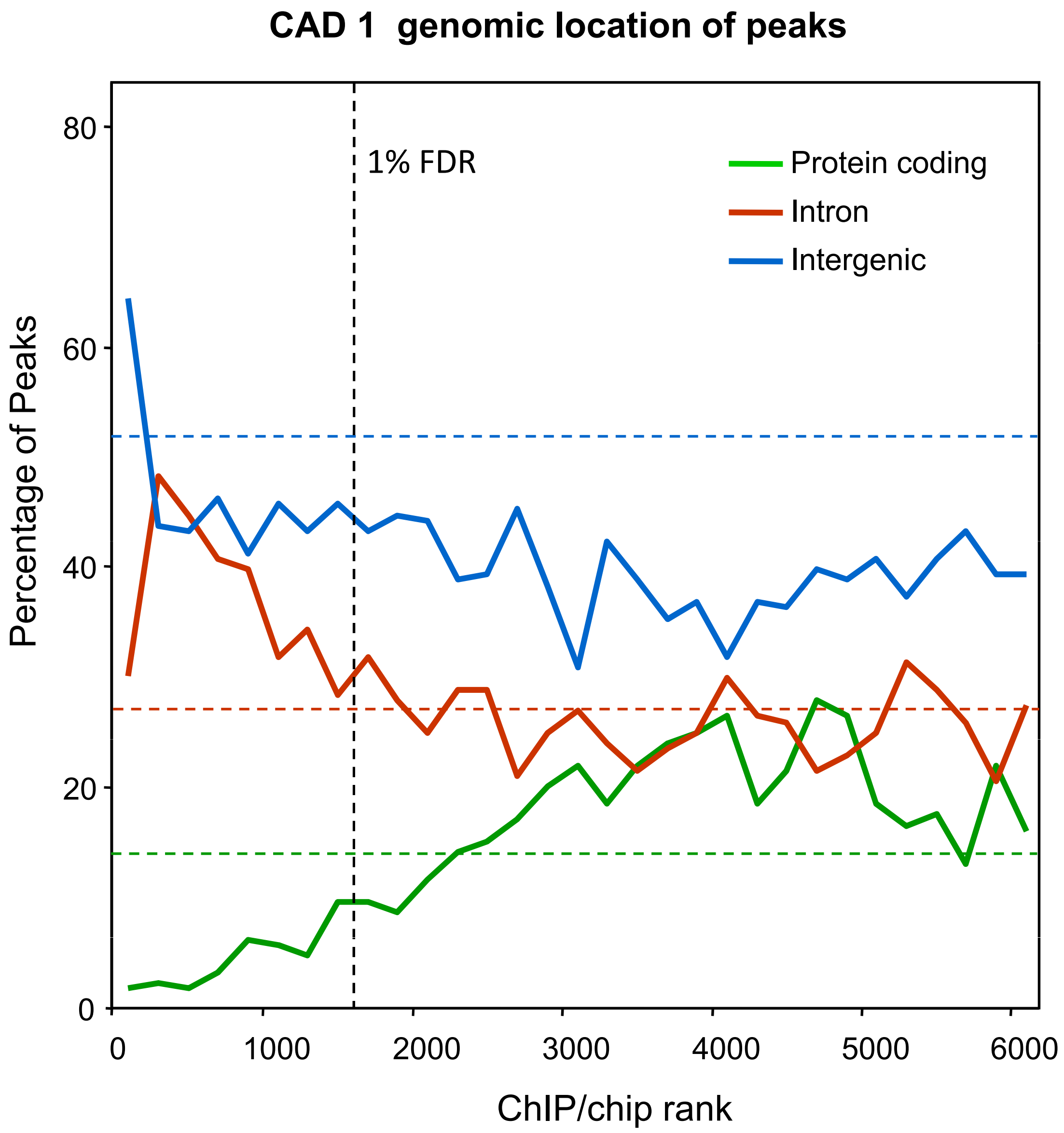

CAD 1 genomic location of peaks
Percentage of Peaks
0
20
40
60
80
0
1000
2000
3000
4000
5000
6000
ChIP/chip rank
1% FDR
Protein coding
Intron
Intergenic

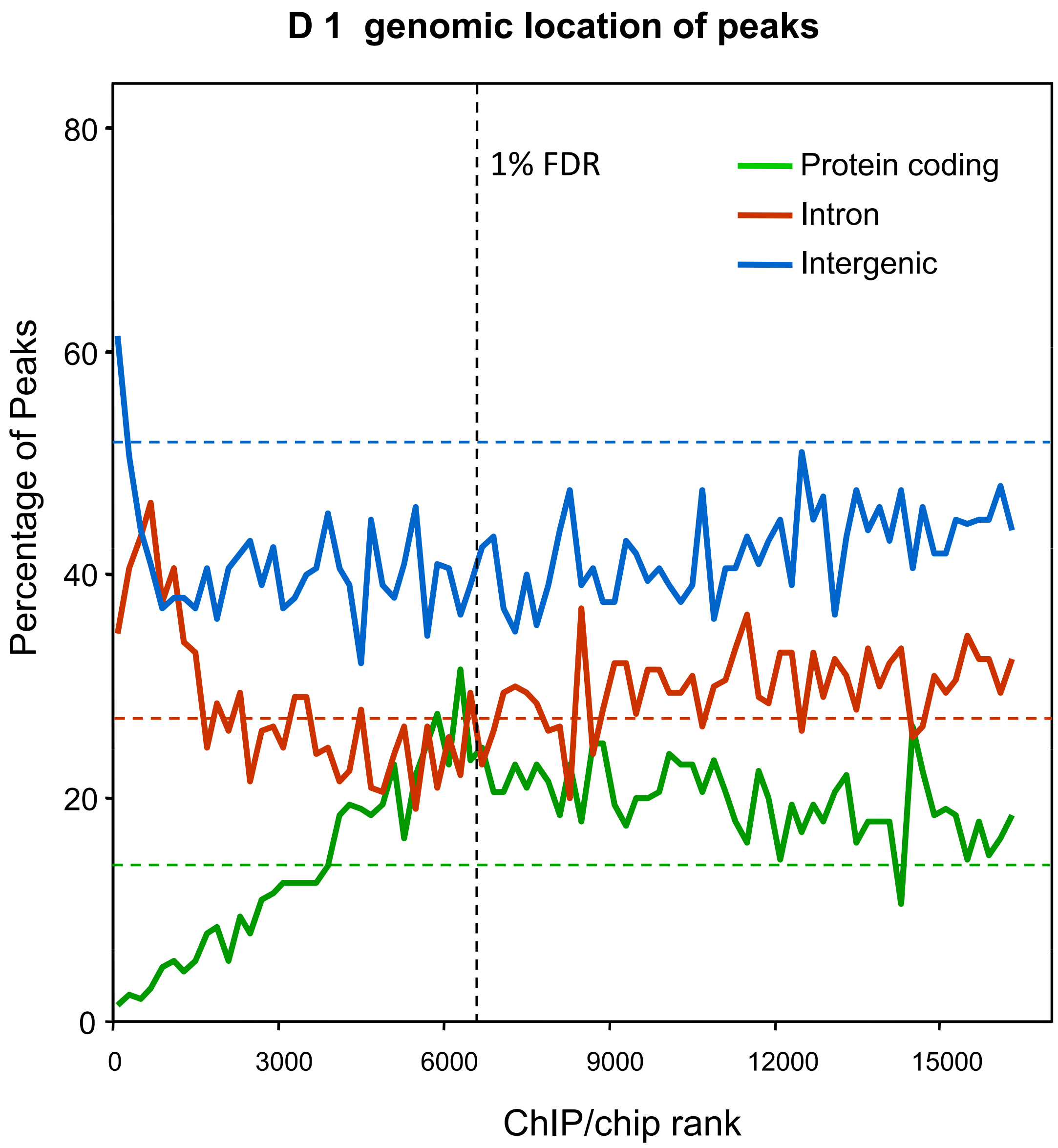
D 1 genomic location of peaks
1% FDR
Protein coding
Intron
Intergenic
Percentage of Peaks
0
20
40
60
80
0
3000
6000
9000
12000
15000
ChIP/chip rank

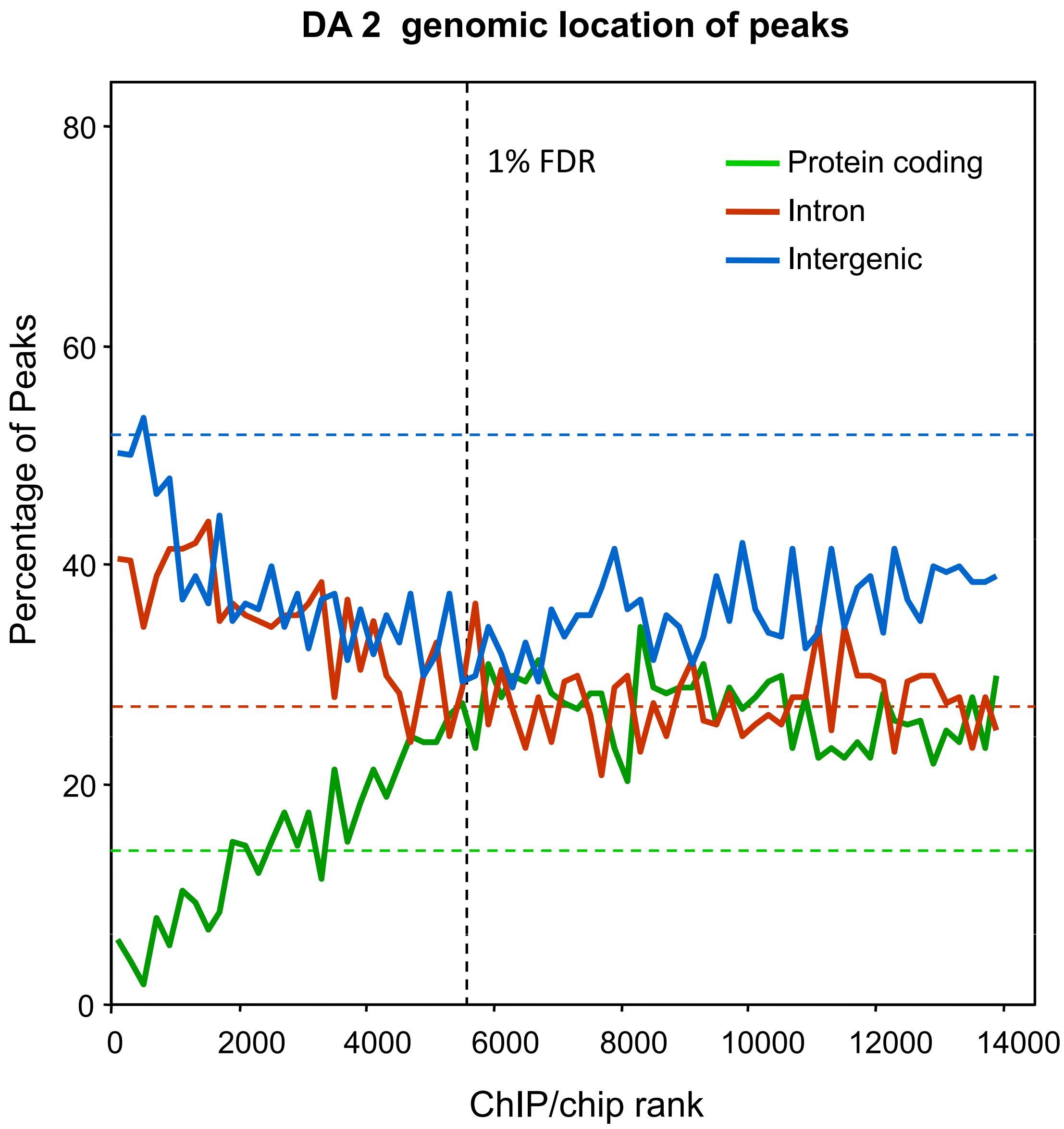
DA 2 genomic location of peaks
1% FDR
Protein coding
Intron
Intergenic
Percentage of Peaks
0
20
40
60
80
ChIP/chip rank
0
2000
4000
6000
8000
10000
12000
14000

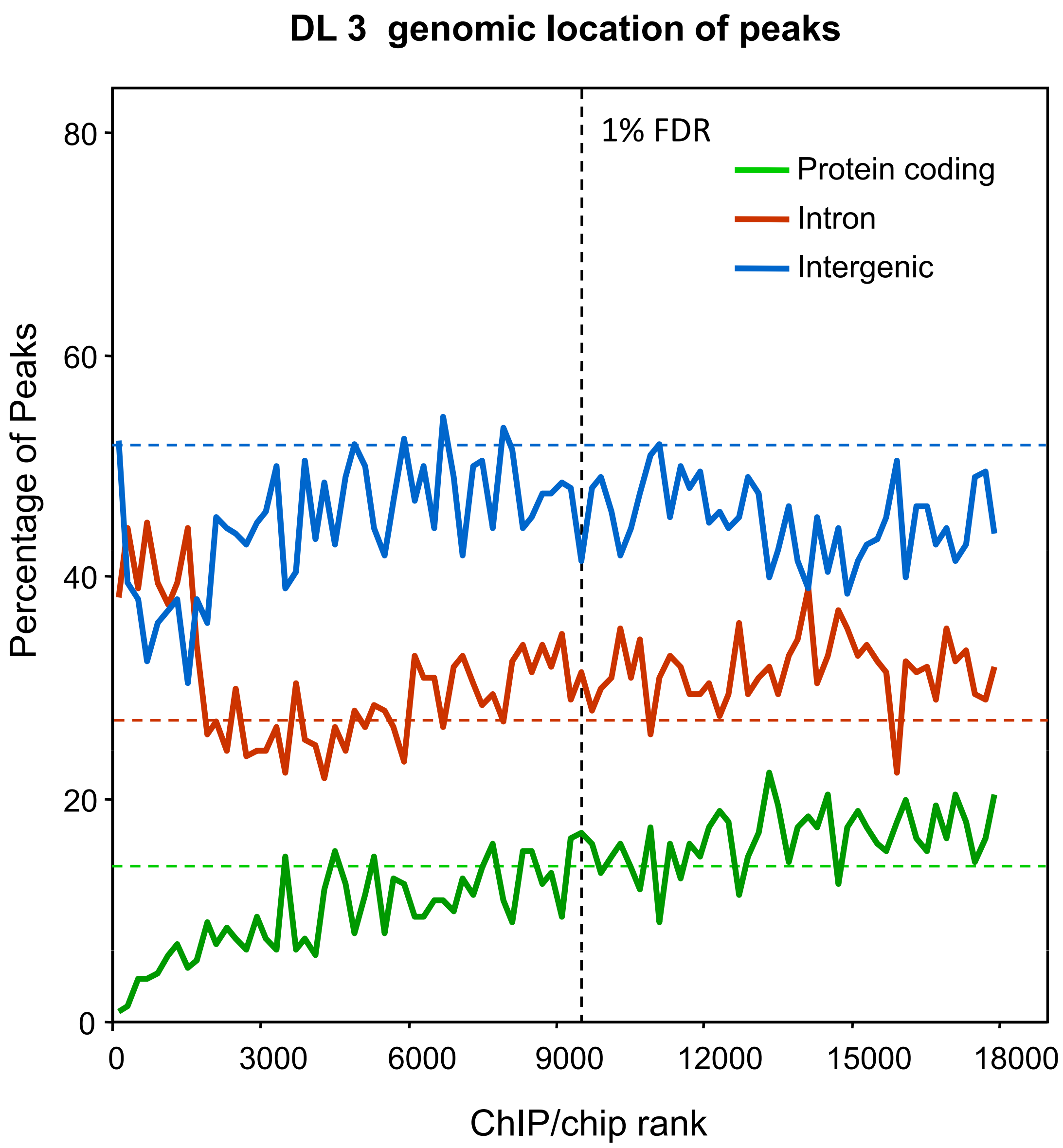
DL 3 genomic location of peaks
1% FDR
Protein coding
Intron
Intergenic
Percentage of Peaks
0
20
40
60
80
0
3000
6000
9000
12000
15000
18000
ChIP/chip rank

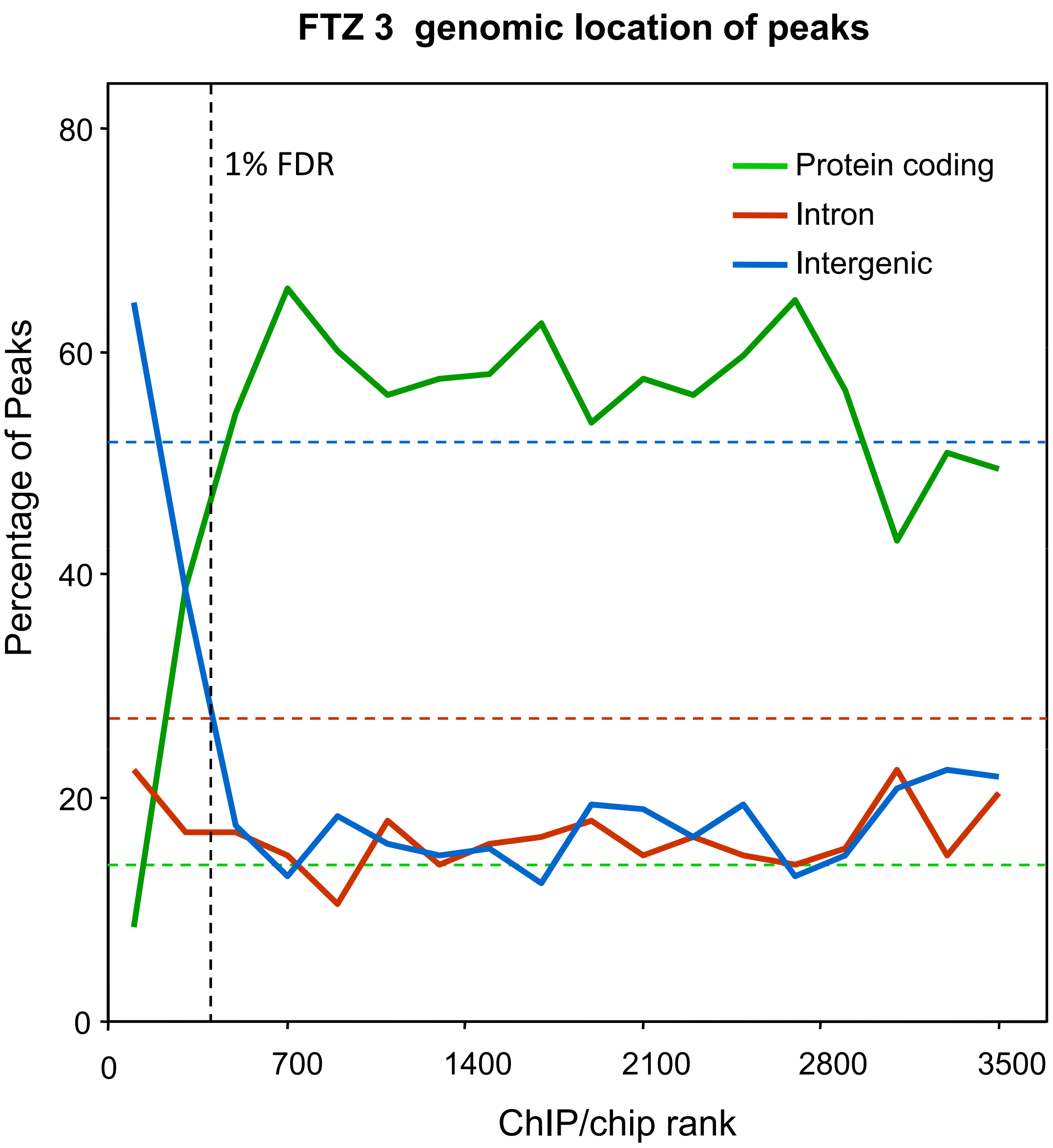
FTZ 3 genomic location of peaks
1% FDR
Protein coding
Intron
Intergenic
Percentage of Peaks
0
20
40
60
80
0
700
1400
2100
2800
3500
ChIP/chip rank

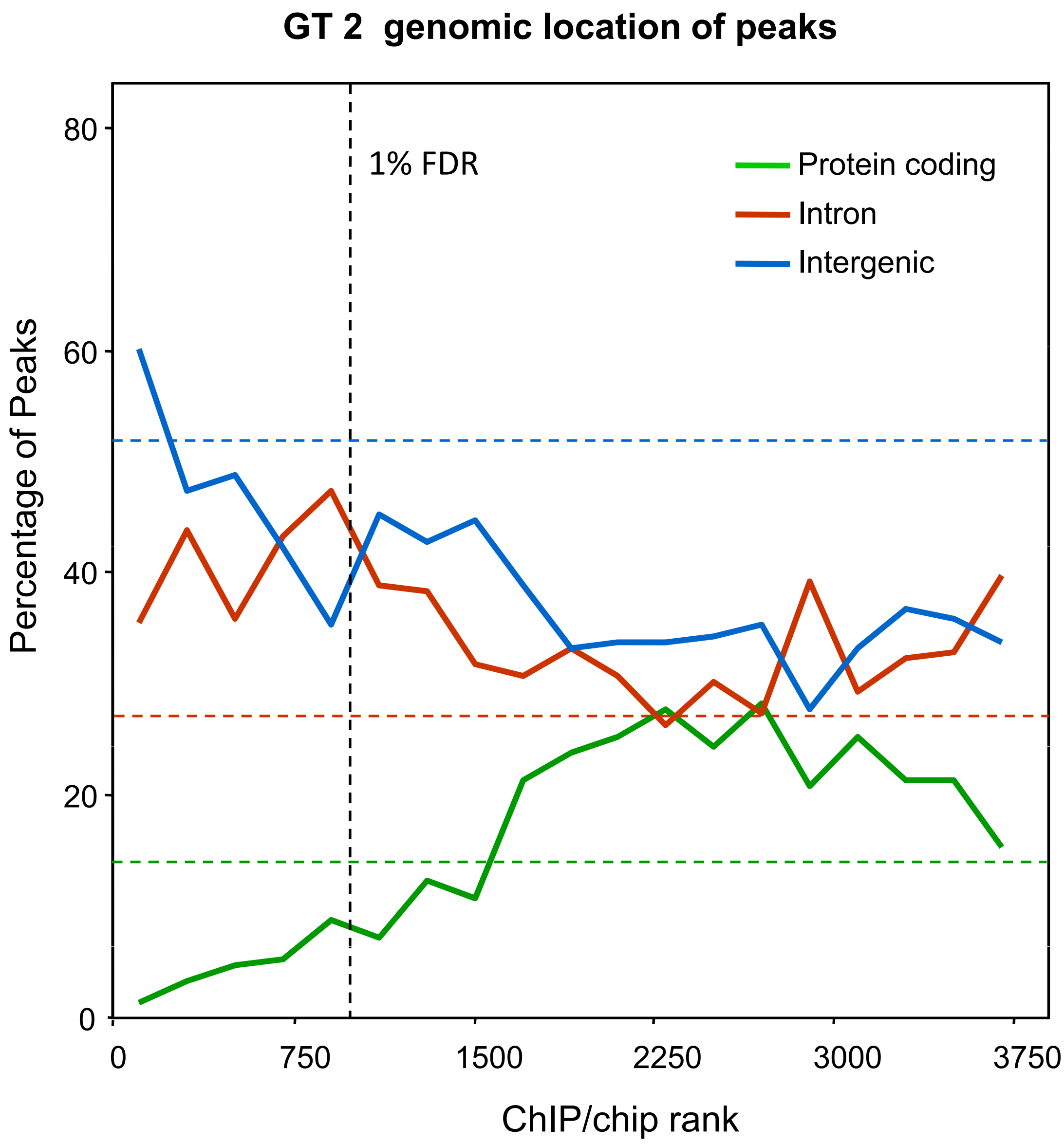
GT 2 genomic location of peaks
1% FDR
Protein coding
Intron
Intergenic
Percentage of Peaks
0
20
40
60
80
0
750
1500
2250
3000
3750
ChIP/chip rank

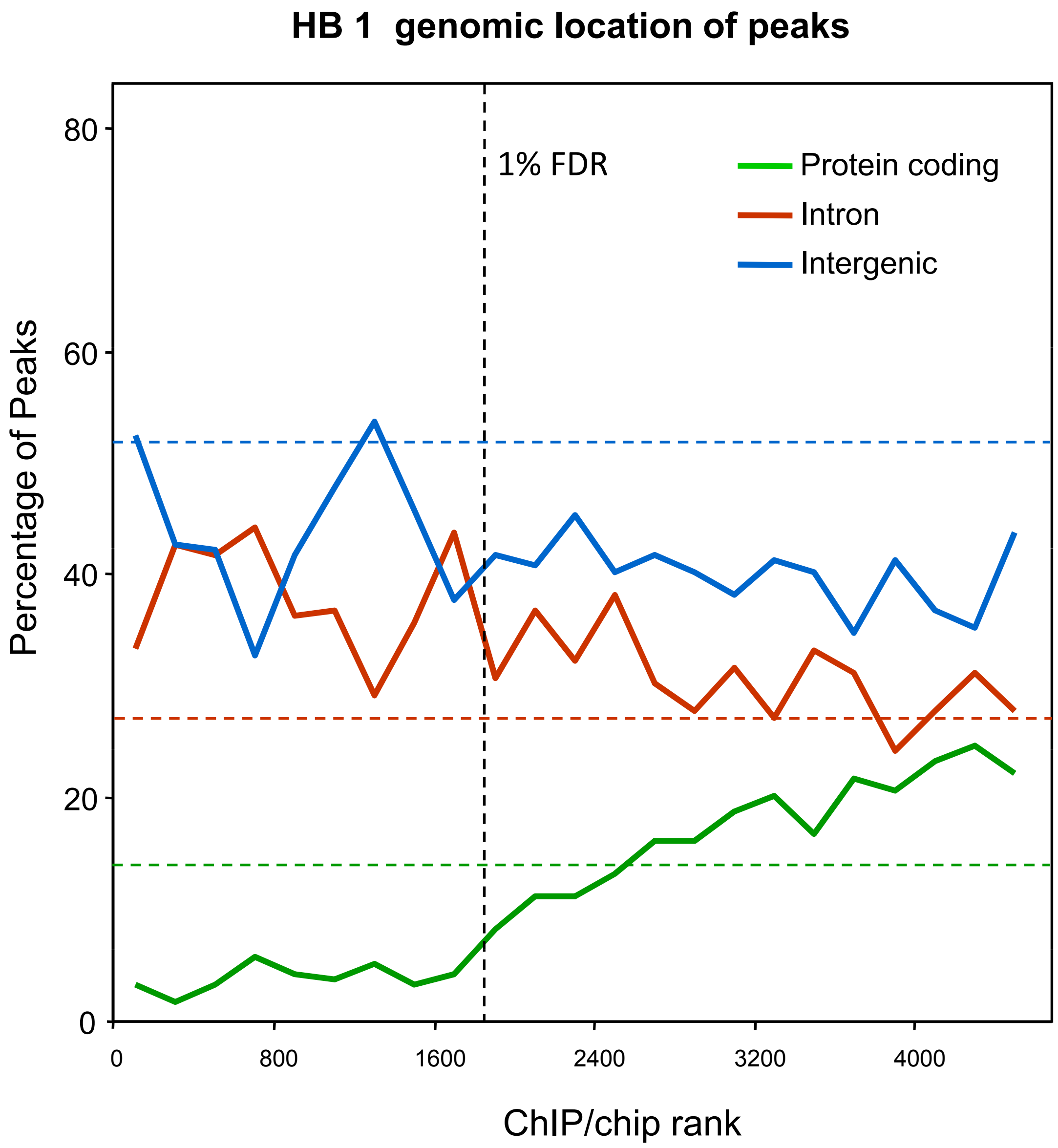
HB 1 genomic location of peaks
1% FDR
Protein coding
Intron
Intergenic
Percentage of Peaks
0
20
40
60
80
0
800
1600
2400
3200
4000
ChIP/chip rank

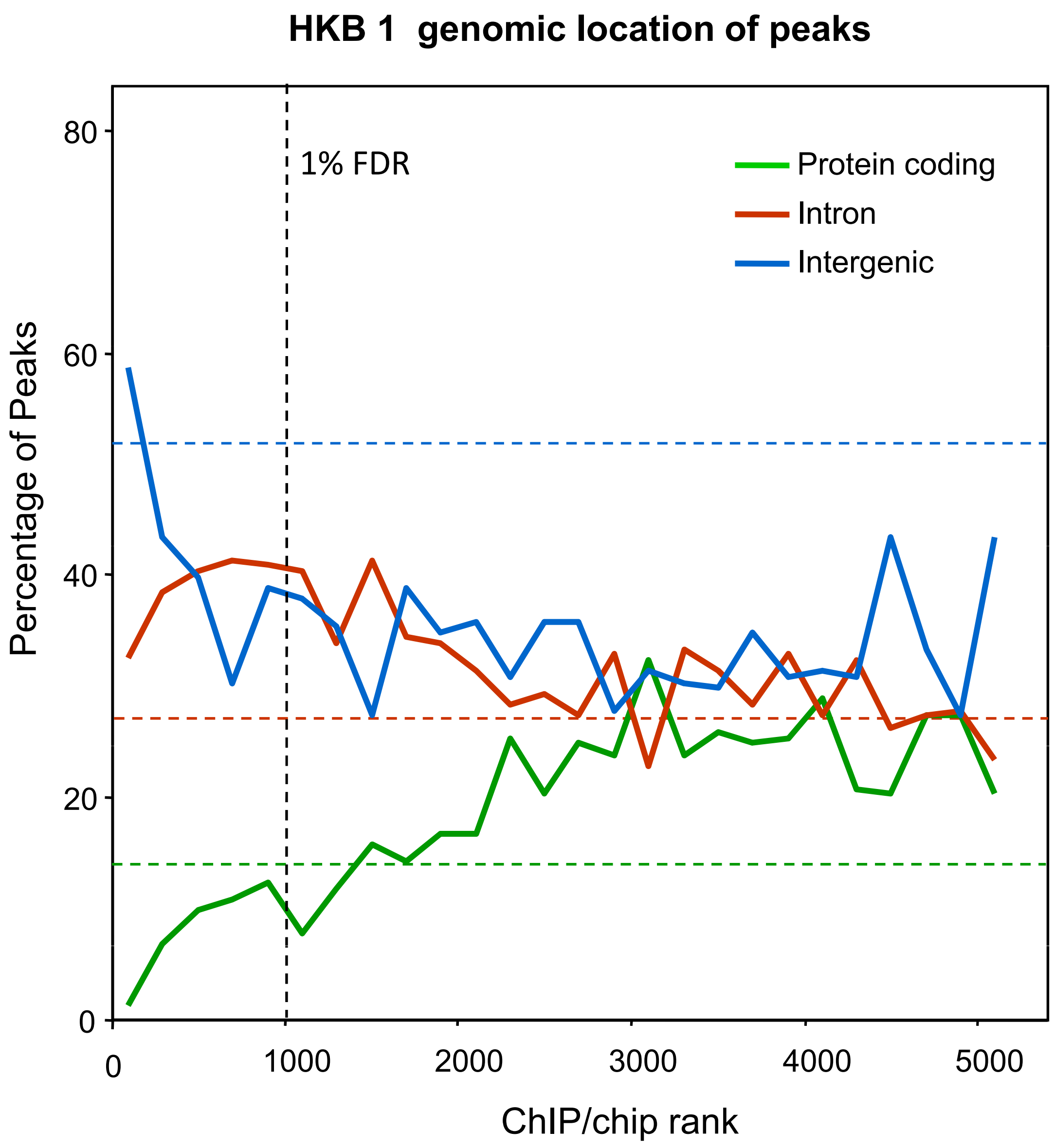
HKB 1 genomic location of peaks
1% FDR
Protein coding
Intron
Intergenic
Percentage of Peaks
0
20
40
60
80
0
1000
2000
3000
4000
5000
ChIP/chip rank

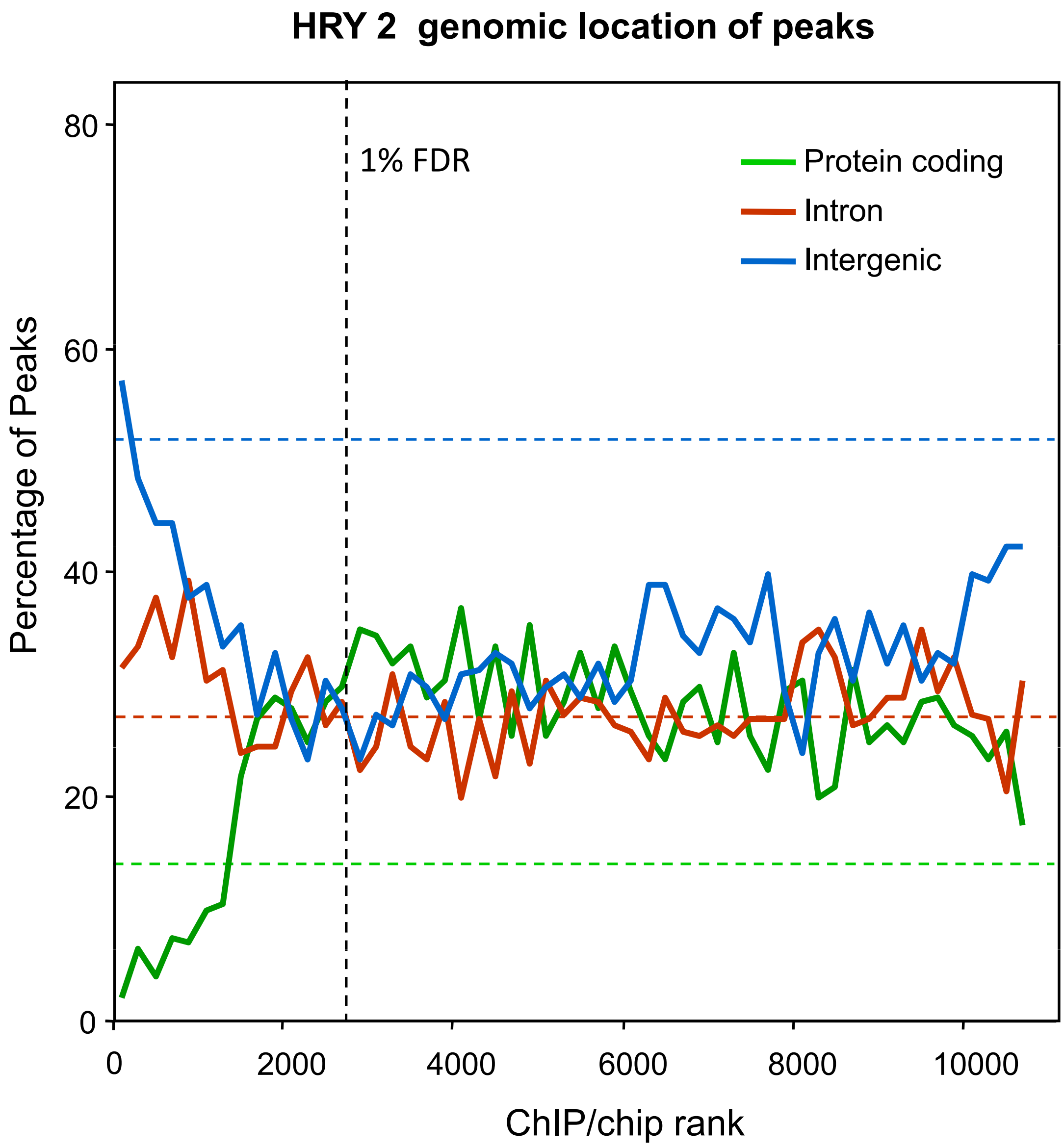
HRY 2 genomic location of peaks
1% FDR
Protein coding
Intron
Intergenic
Percentage of Peaks
0
20
40
60
80
0
2000
4000
6000
8000
10000
ChIP/chip rank

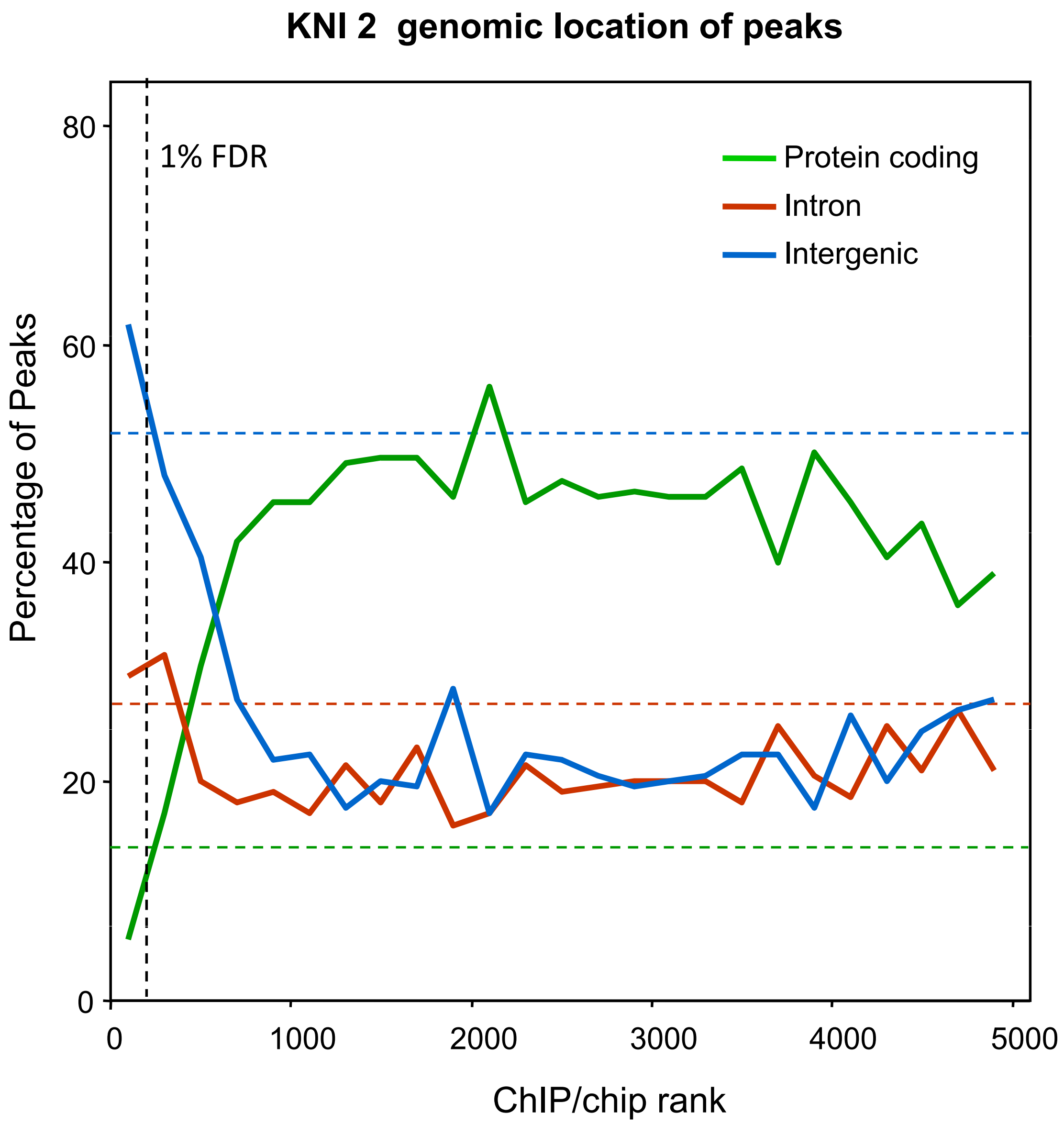
KNI 2 genomic location of peaks
1% FDR
Protein coding
Intron
Intergenic
Percentage of Peaks
0
20
40
60
80
0
1000
2000
3000
4000
5000
ChIP/chip rank

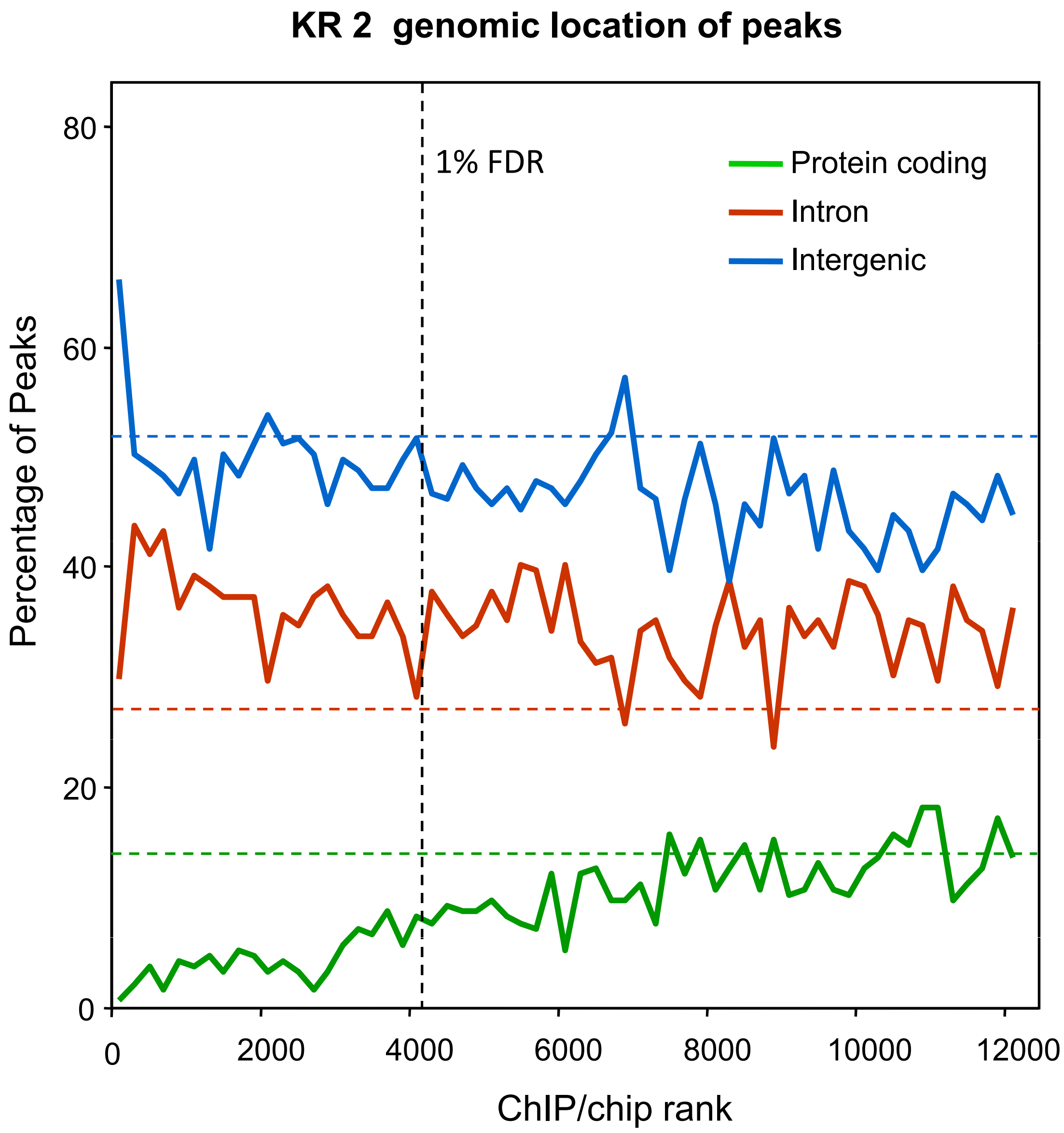
KR 2 genomic location of peaks
1% FDR
Protein coding
Intron
Intergenic
Percentage of Peaks
0
20
40
60
80
0
2000
4000
6000
8000
10000
12000
ChIP/chip rank

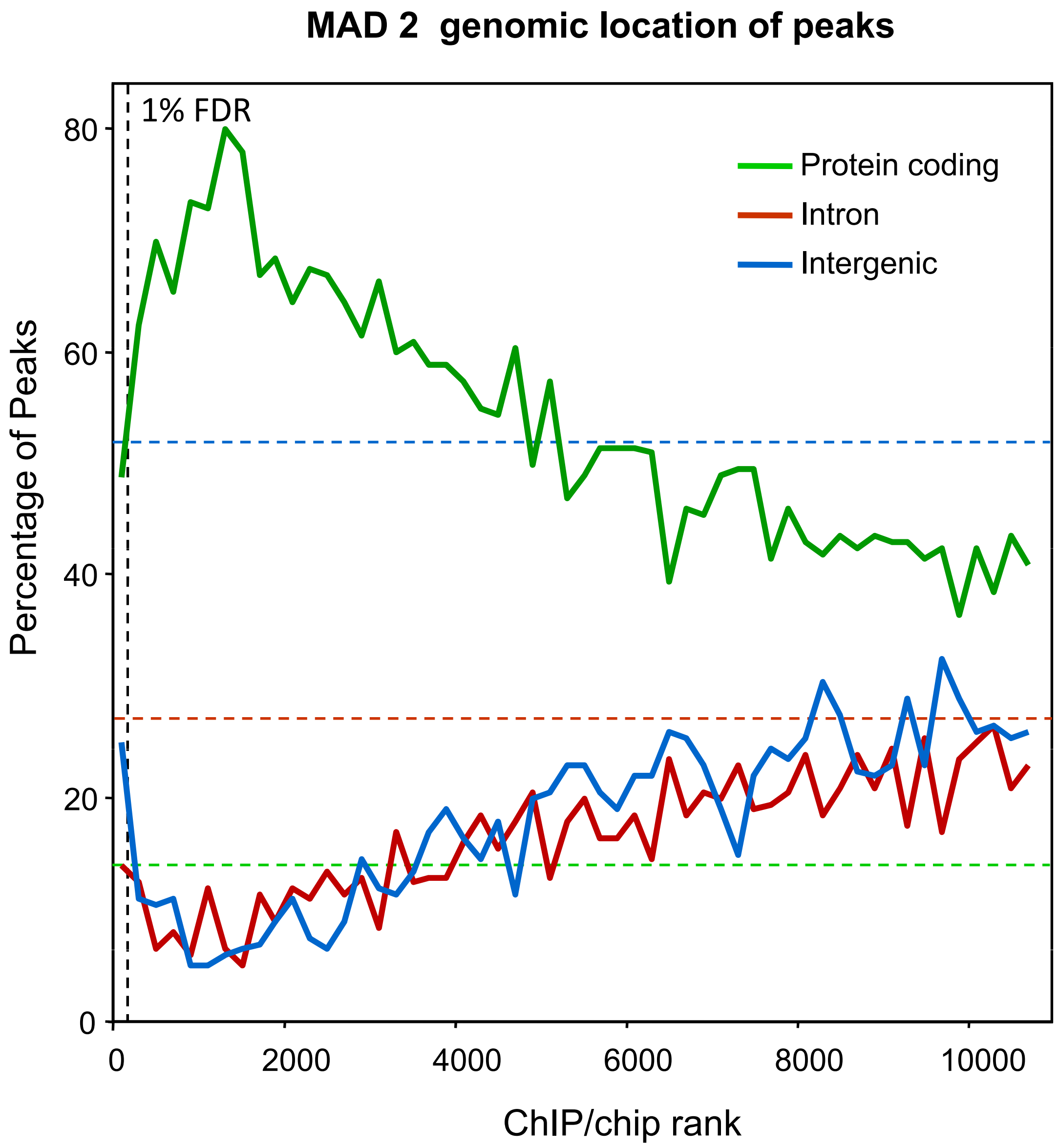
MAD 2 genomic location of peaks
1% FDR
Protein coding
Intron
Intergenic
Percentage of Peaks
ChIP/chip rank
0
20
40
60
80
0
2000
4000
6000
8000
10000

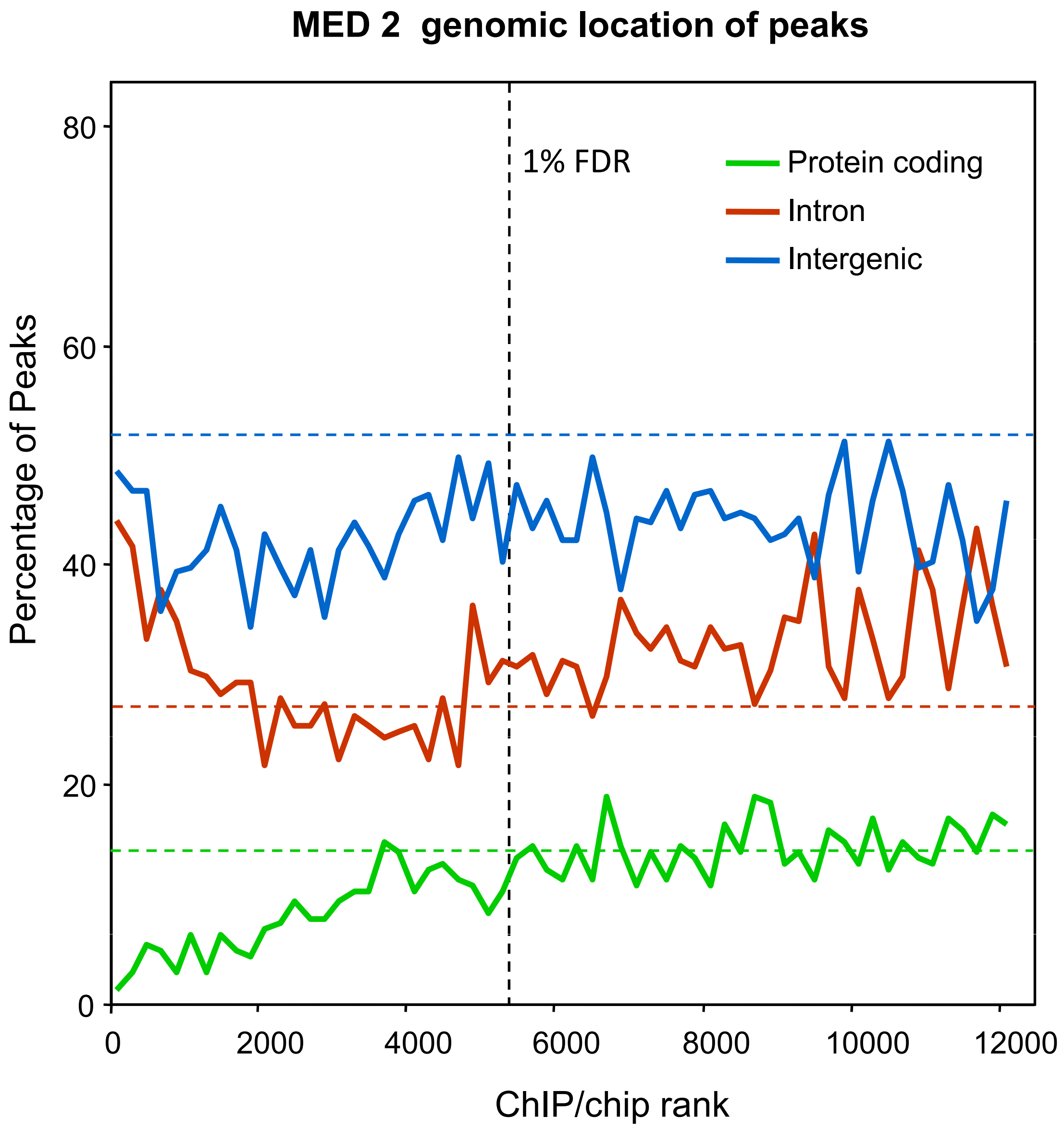
MED 2 genomic location of peaks
1% FDR
Protein coding
Intron
Intergenic
Percentage of Peaks
0
20
40
60
80
0
2000
4000
6000
8000
10000
12000
ChIP/chip rank

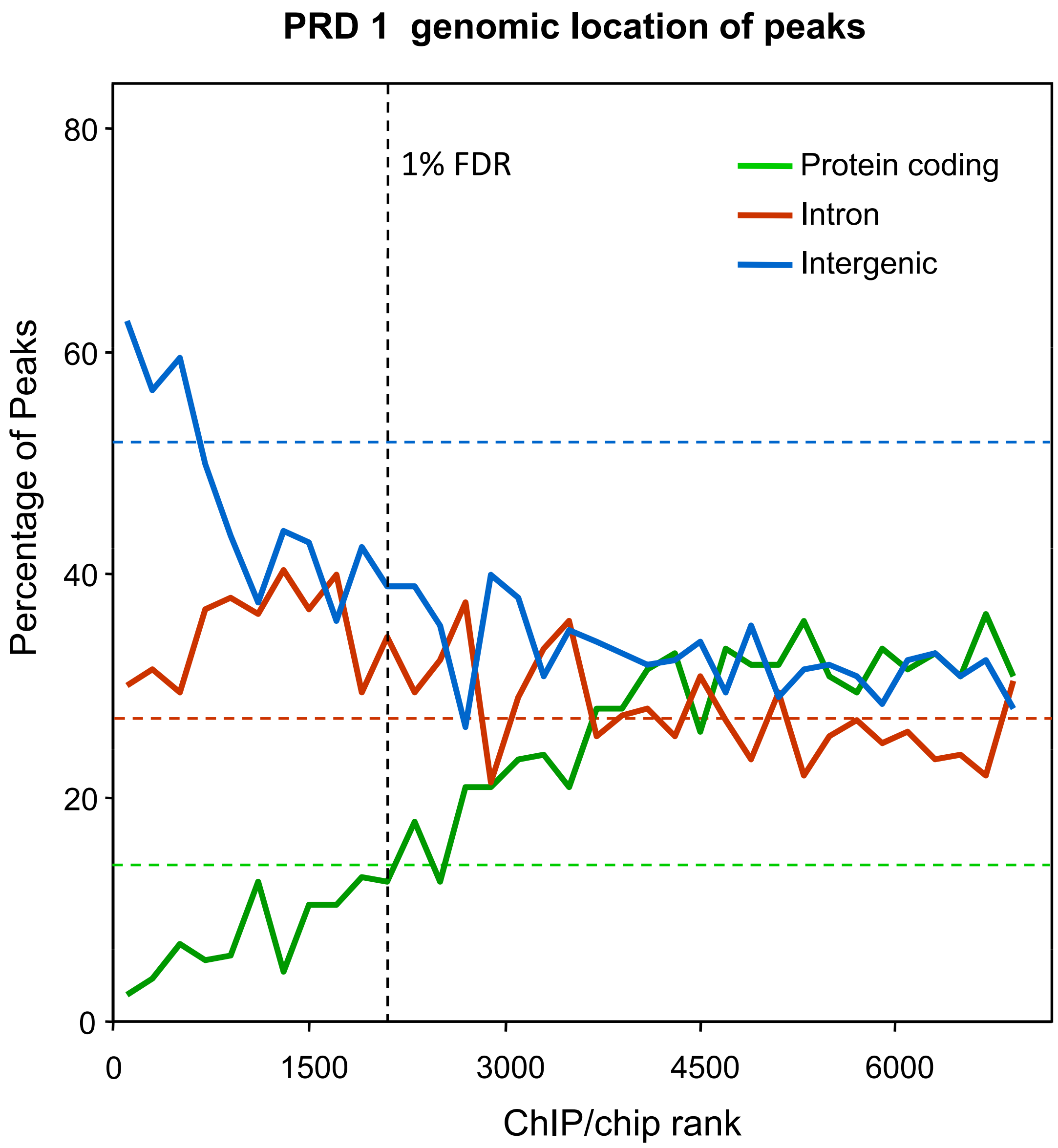
PRD 1 genomic location of peaks
1% FDR
Protein coding
Intron
Intergenic
Percentage of Peaks
0
20
40
60
80
0
1500
3000
4500
6000
ChIP/chip rank

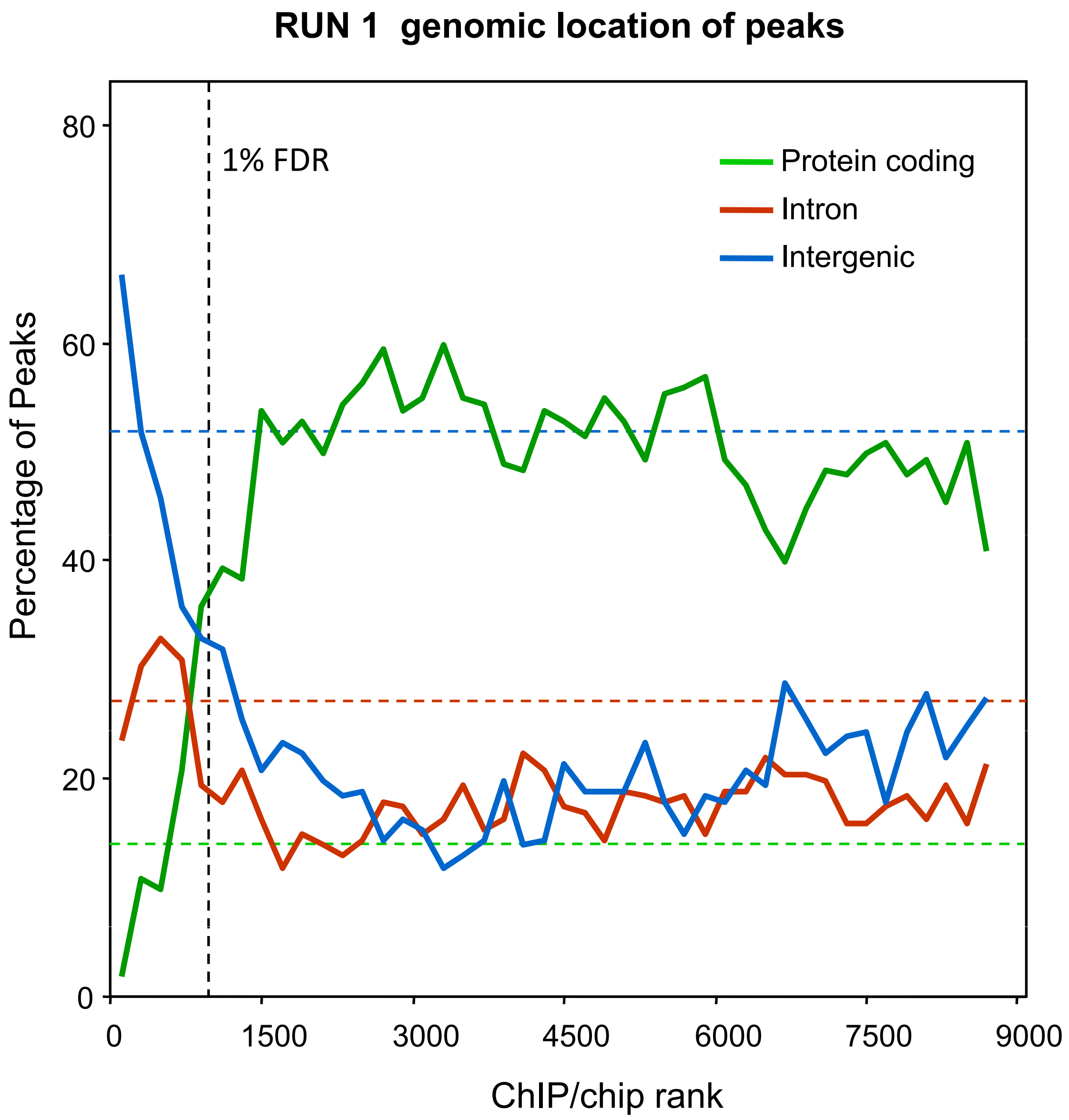
RUN 1 genomic location of peaks
Percentage of Peaks
80
60
40
20
0
0
1500
3000
4500
6000
7500
9000
ChIP/chip rank
1% FDR
Protein coding
Intron
Intergenic

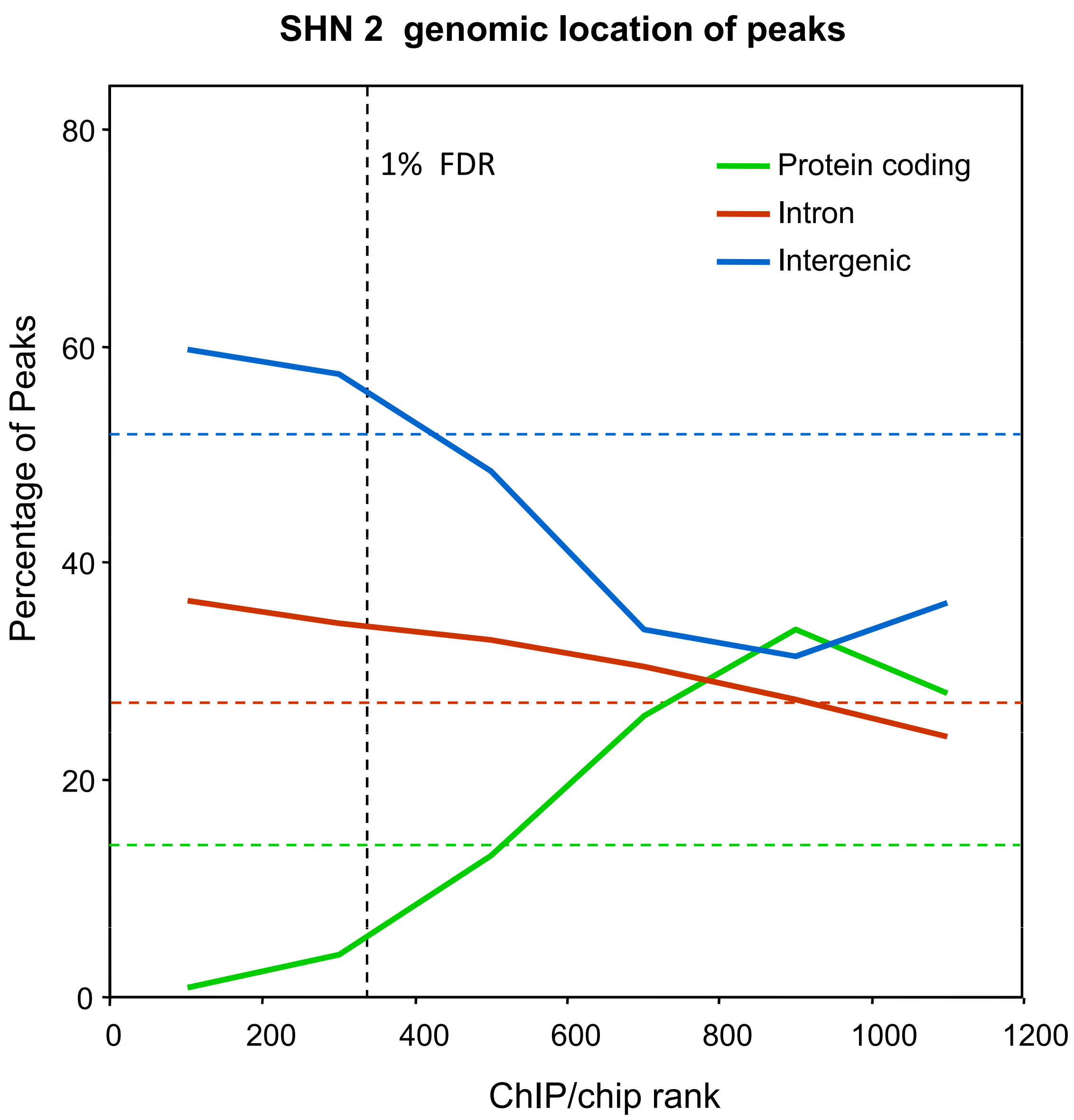
SHN 2 genomic location of peaks
1% FDR
Protein coding
Intron
Intergenic
Percentage of Peaks
0
20
40
60
80
0
200
400
600
800
1000
1200
ChIP/chip rank

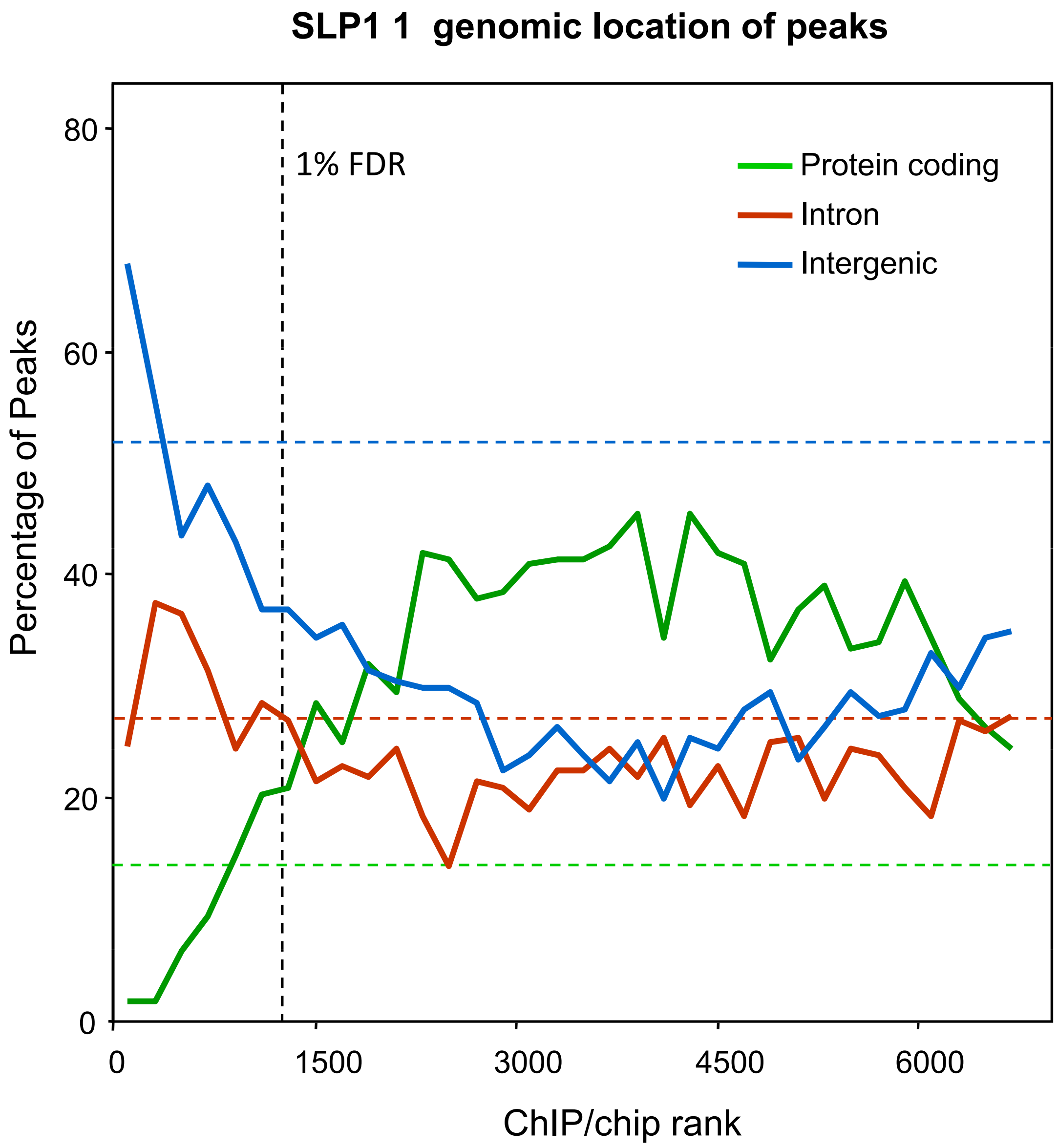
SLP1 1 genomic location of peaks
80
60
40
20
0
Percentage of Peaks
1% FDR
Protein coding
Intron
Intergenic
0
1500
3000
4500
6000
ChIP/chip rank

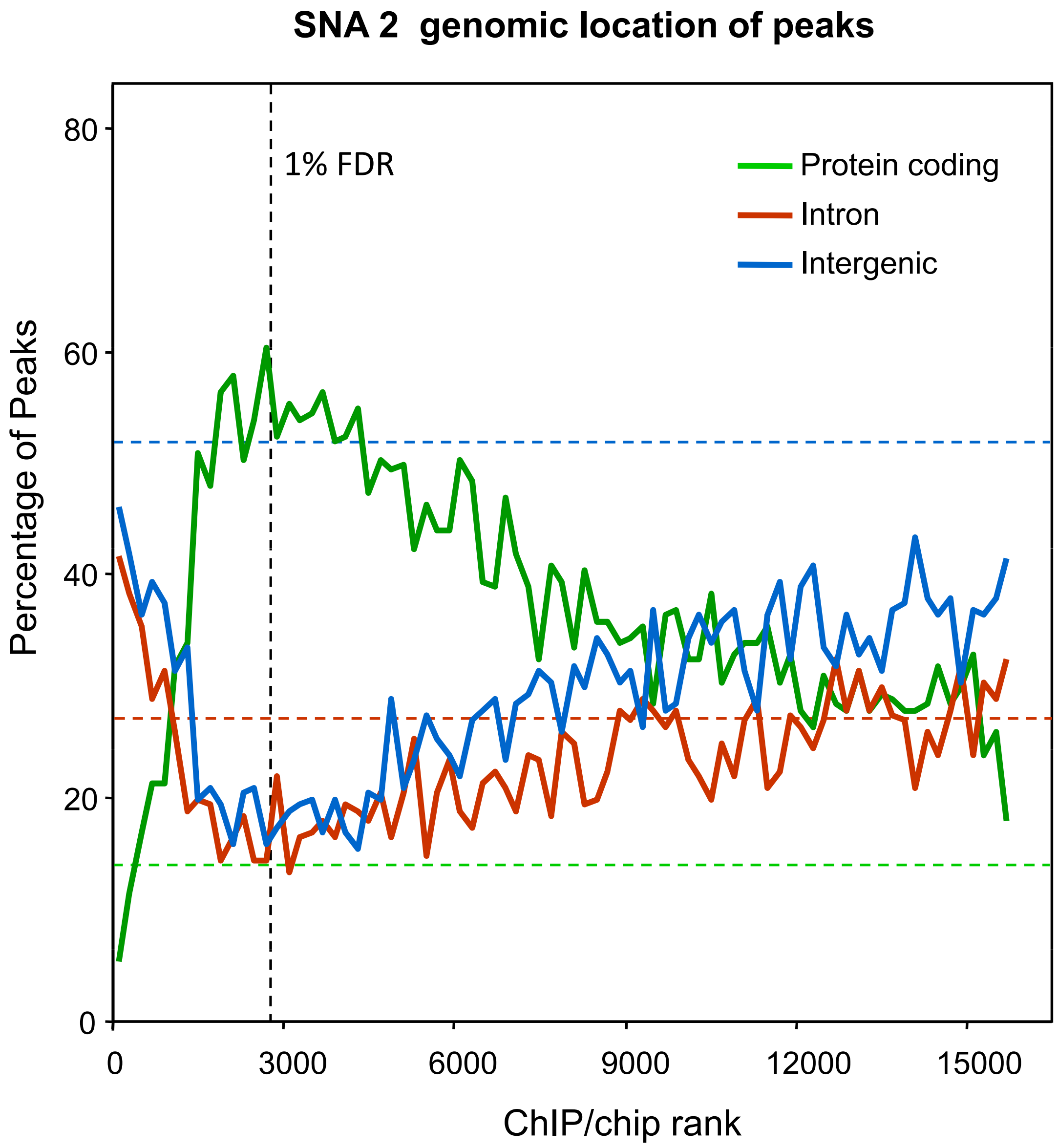
SNA 2 genomic location of peaks
1% FDR
Protein coding
Intron
Intergenic
Percentage of Peaks
0
20
40
60
80
0
3000
6000
9000
12000
15000
ChIP/chip rank

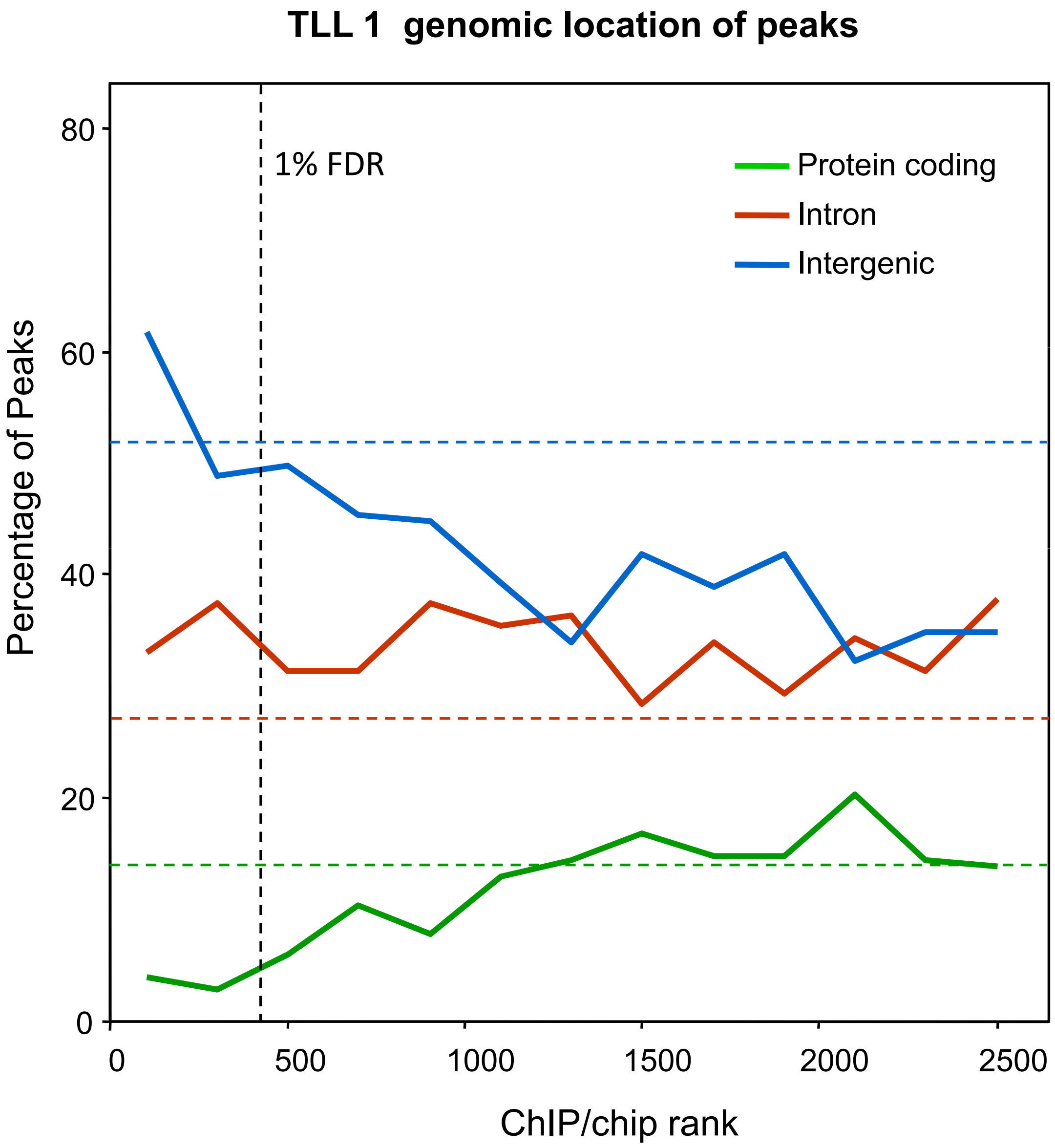
TLL 1 genomic location of peaks
1% FDR
Protein coding
Intron
Intergenic
Percentage of Peaks
ChIP/chip rank
0
20
40
60
80
0
500
1000
1500
2000
2500

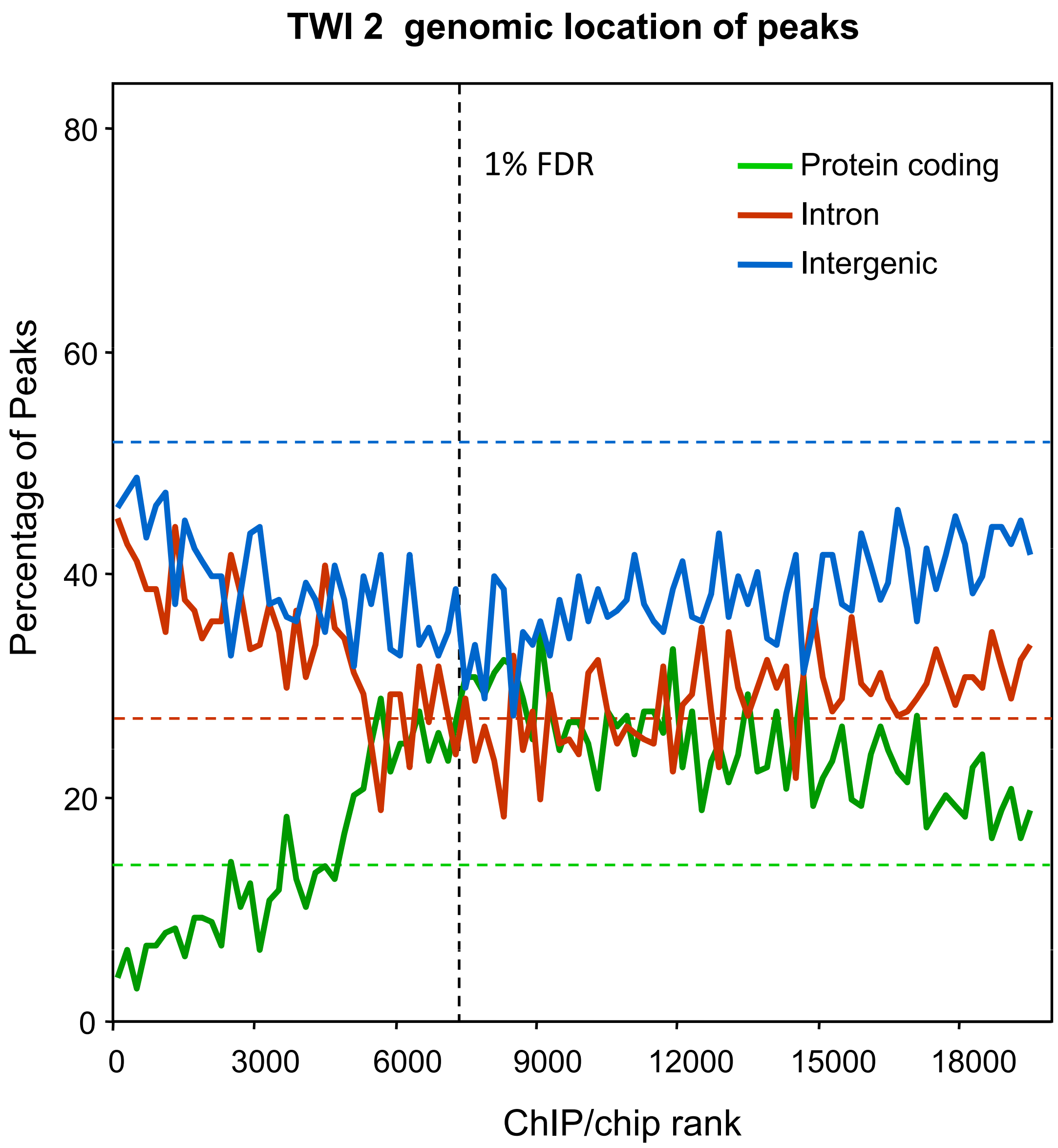
TWI 2 genomic location of peaks
1% FDR
Protein coding
Intron
Intergenic
Percentage of Peaks
0
20
40
60
80
0
3000
6000
9000
12000
15000
18000
ChIP/chip rank
