# Supplementary material for: Developmental roles of 21 Drosophila transcription factors are determined by quantitative differences in binding to an overlapping set of thousands of genomic regions
Source: Genome Biol. 2009 Jul 23;10(7):R80. doi: 10.1186/gb-2009-10-7-r80 (PMC2728534; doi:10.1186/gb-2009-10-7-r80)
Supplement: Additional data file 12 — These are plotted down the ChIP/chip rank list in non-overlapping 200-peak cohorts. [file gb-2009-10-7-r80-S12.pdf]

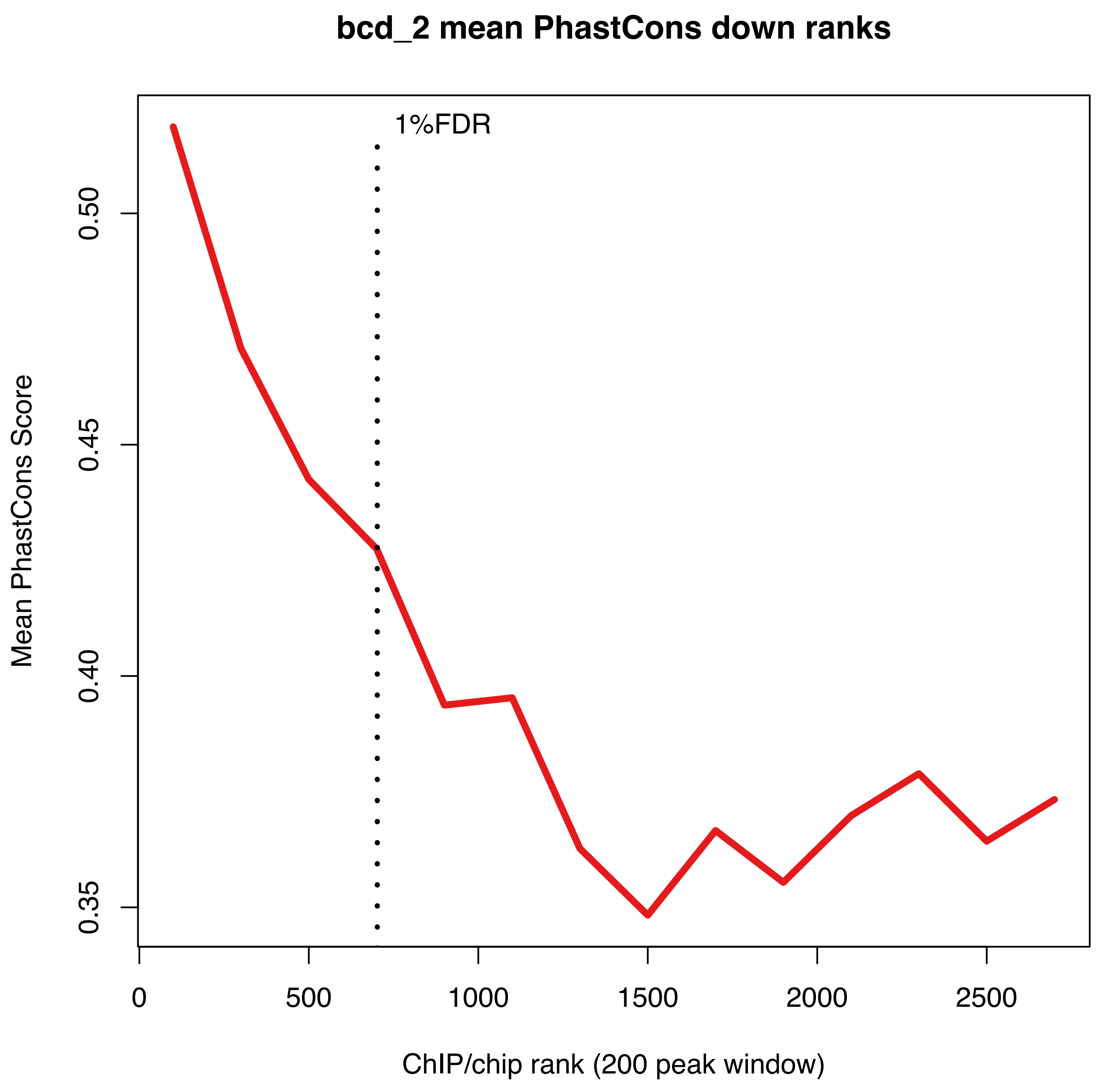
bcd_2 mean PhastCons down ranks
1%FDR
Mean PhastCons Score
0.35
0.40
0.45
0.50
0
500
1000
1500
2000
2500
ChIP/chip rank (200 peak window)

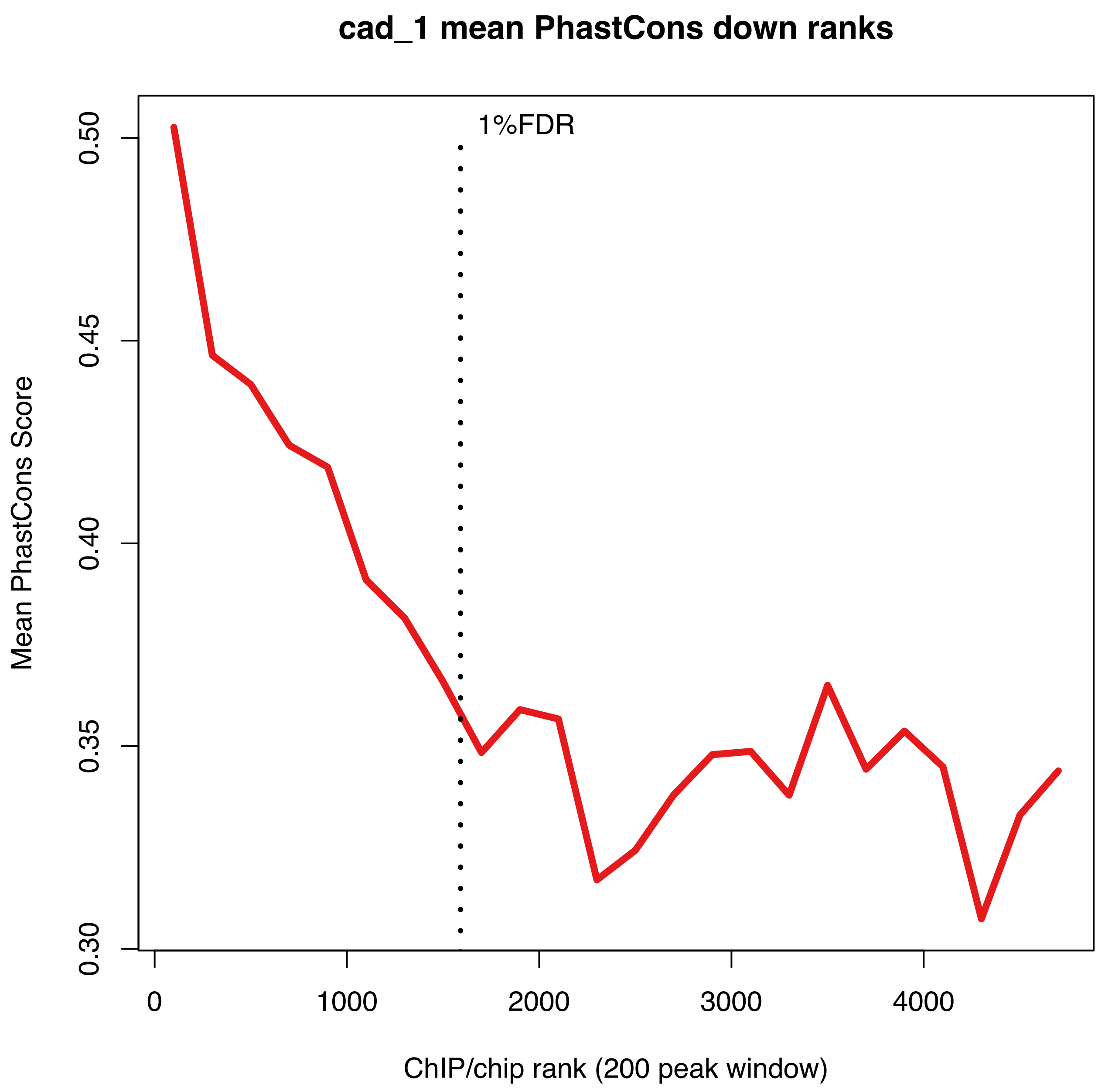
cad_1 mean PhastCons down ranks
1%FDR
Mean PhastCons Score
0.30
0.35
0.40
0.45
0.50
0
1000
2000
3000
4000
ChIP/chip rank (200 peak window)

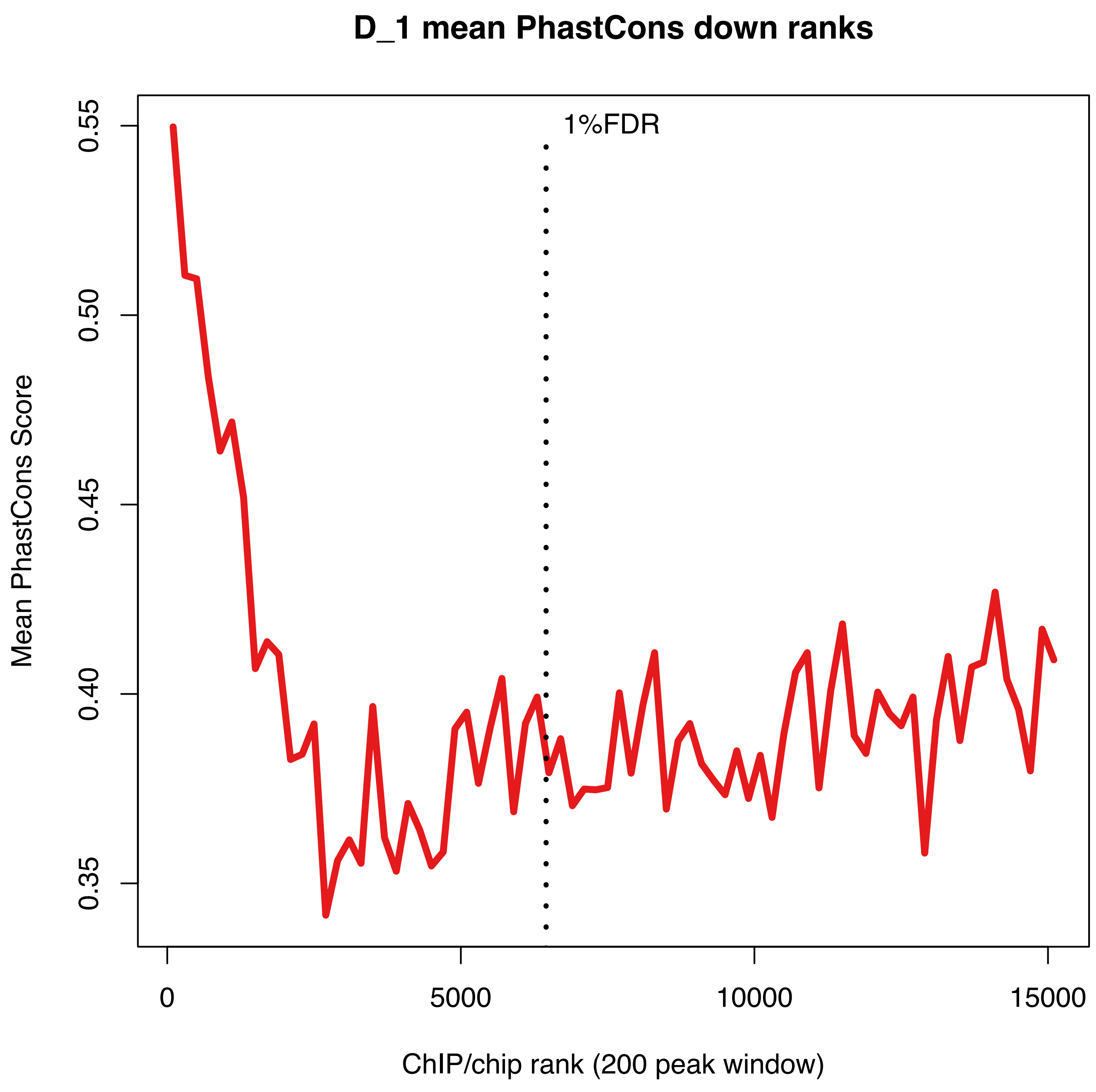
D_1 mean PhastCons down ranks
1%FDR
Mean PhastCons Score
0.35
0.40
0.45
0.50
0.55
0
5000
10000
15000
ChIP/chip rank (200 peak window)

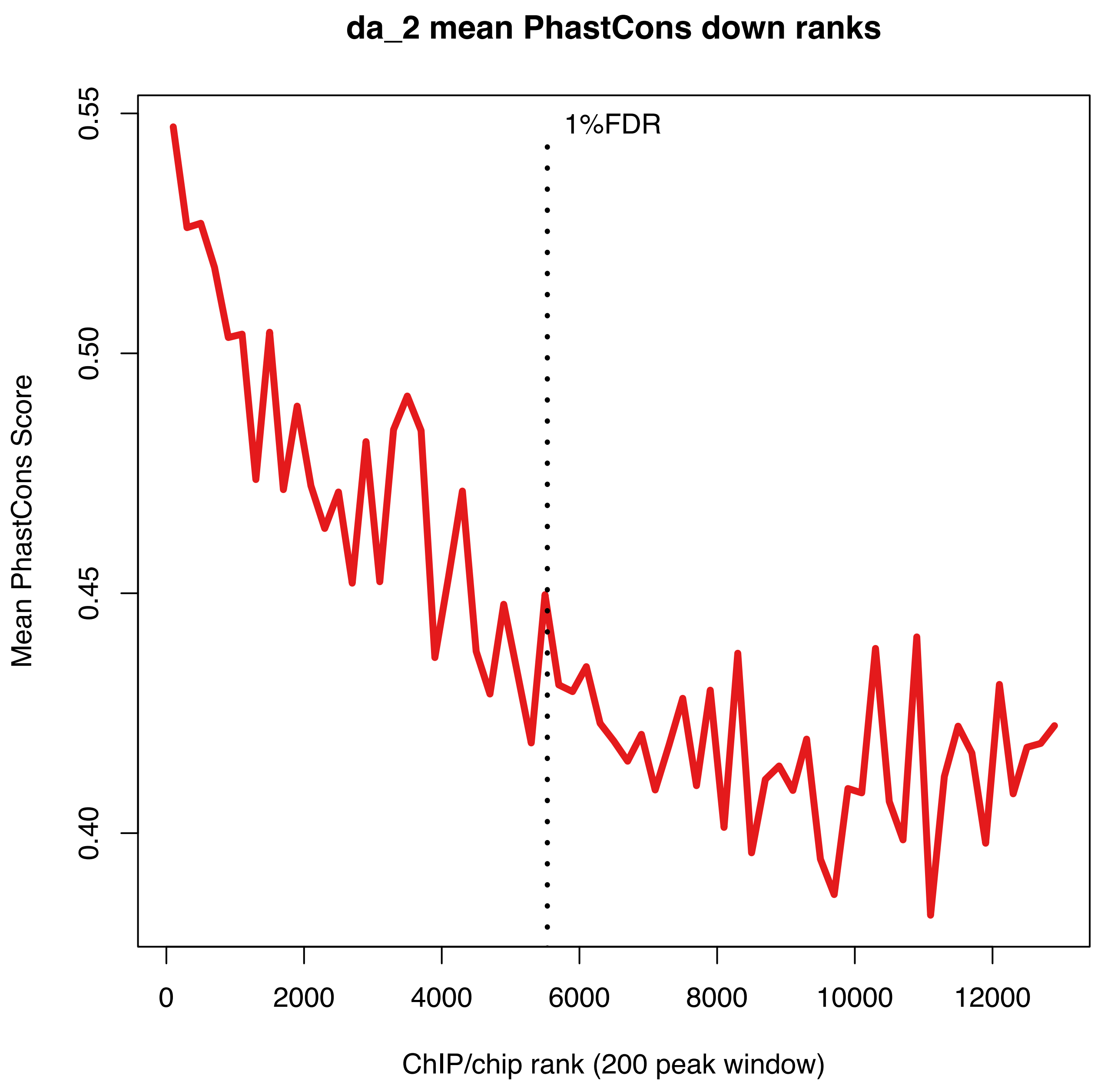
da_2 mean PhastCons down ranks
1%FDR
Mean PhastCons Score
0.40
0.45
0.50
0.55
0
2000
4000
6000
8000
10000
12000
ChIP/chip rank (200 peak window)

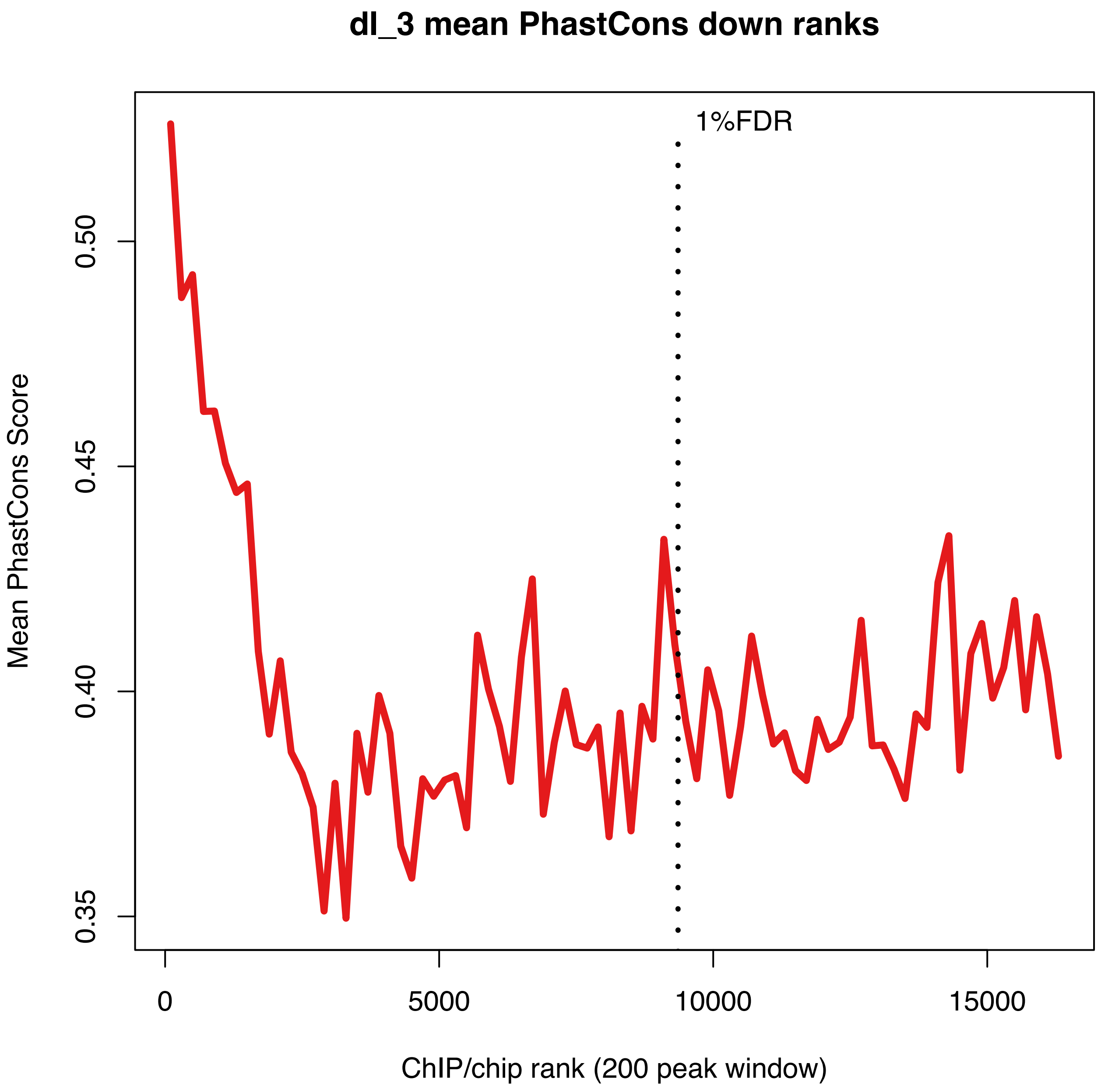
dl_3 mean PhastCons down ranks
1%FDR
Mean PhastCons Score
0.35
0.40
0.45
0.50
0
5000
10000
15000
ChIP/chip rank (200 peak window)

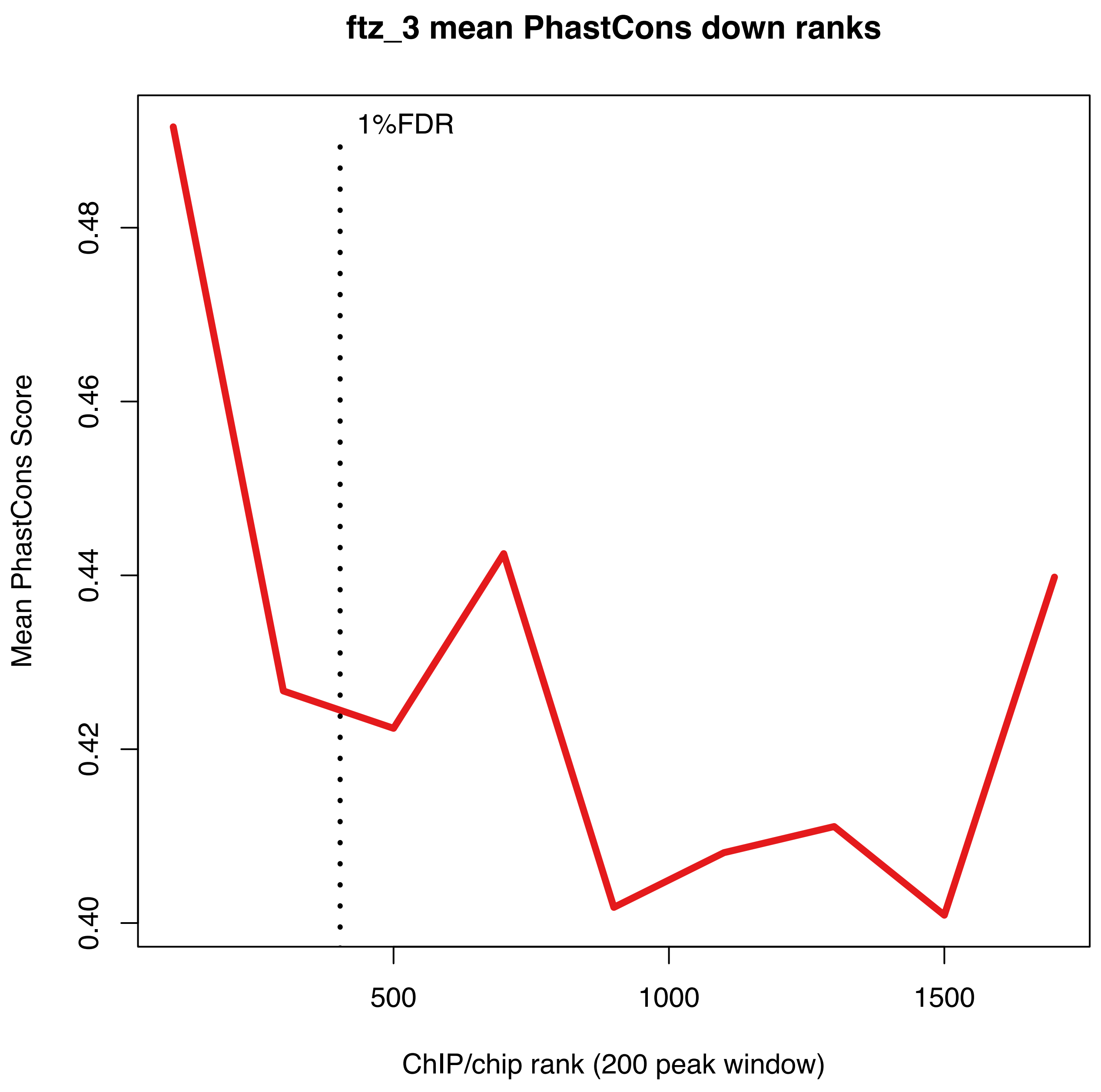
ftz_3 mean PhastCons down ranks
1%FDR
Mean PhastCons Score
0.40
0.42
0.44
0.46
0.48
500
1000
1500
ChIP/chip rank (200 peak window)

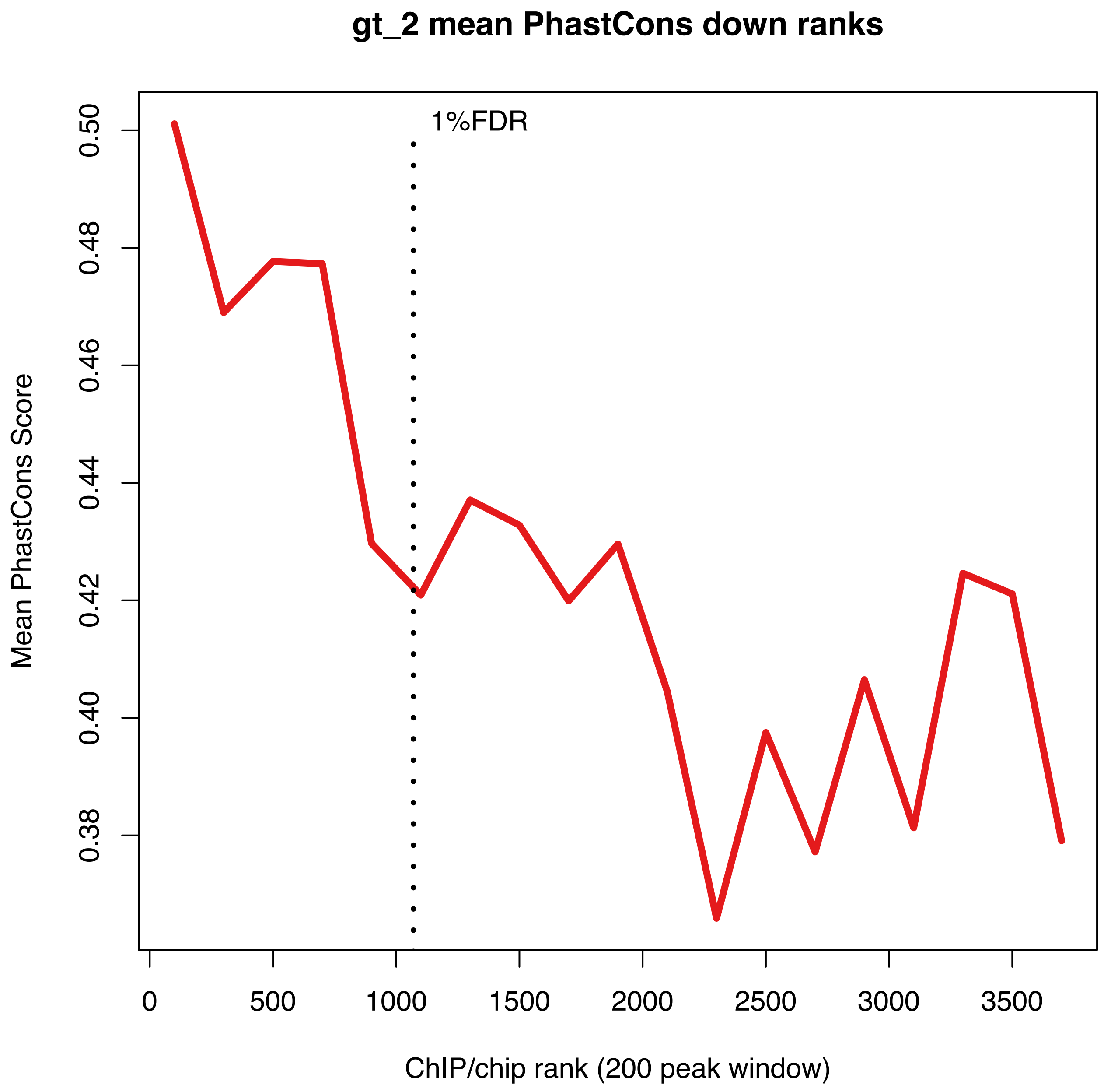
gt_2 mean PhastCons down ranks
1%FDR
Mean PhastCons Score
0.38
0.40
0.42
0.44
0.46
0.48
0.50
0
500
1000
1500
2000
2500
3000
3500
ChIP/chip rank (200 peak window)

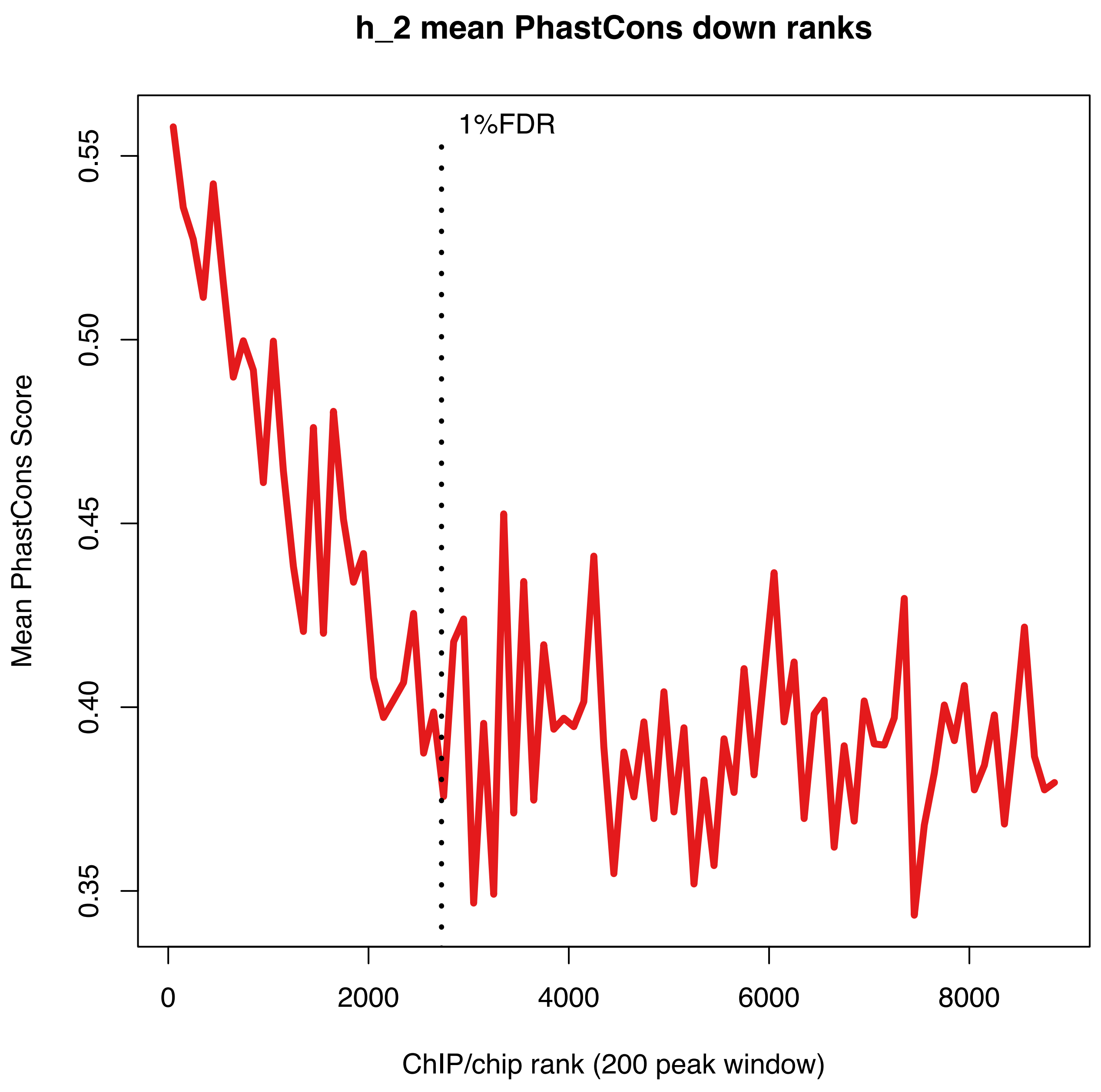
h_2 mean PhastCons down ranks
1%FDR
Mean PhastCons Score
0.35
0.40
0.45
0.50
0.55
0
2000
4000
6000
8000
ChIP/chip rank (200 peak window)

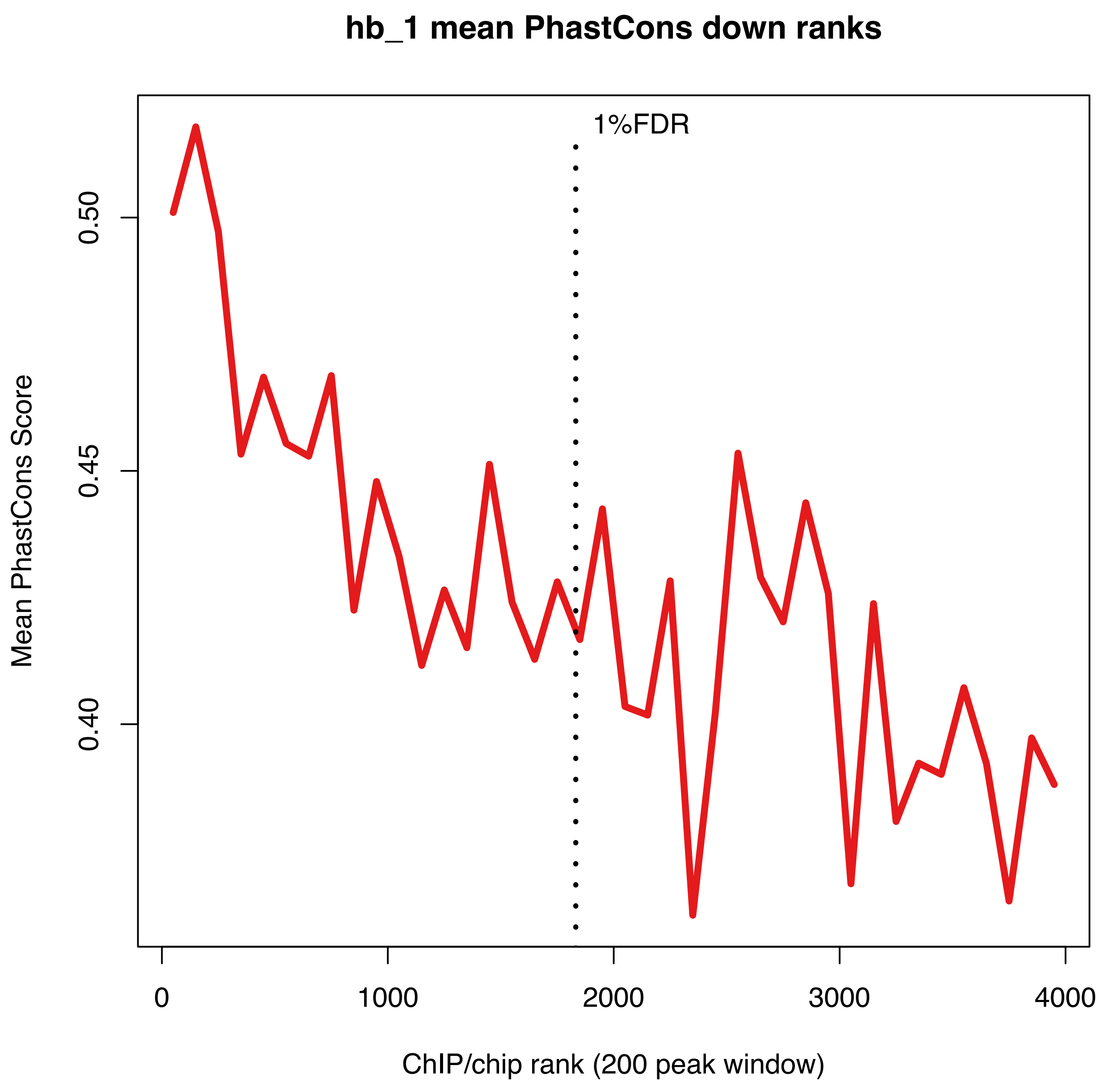
hb_1 mean PhastCons down ranks
1%FDR
Mean PhastCons Score
0.40
0.45
0.50
0
1000
2000
3000
4000
ChIP/chip rank (200 peak window)

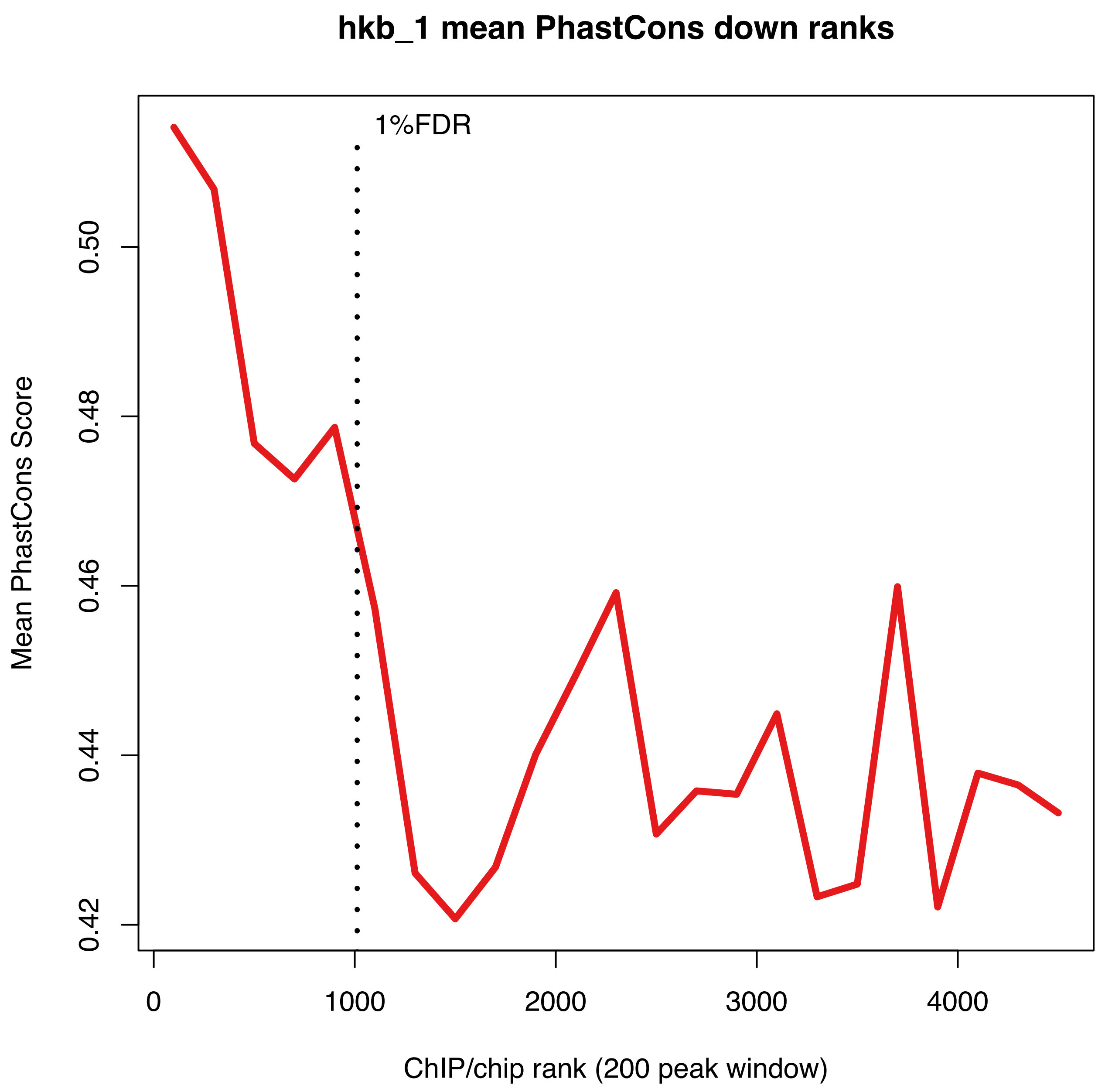
hkb_1 mean PhastCons down ranks
1%FDR
Mean PhastCons Score
0.42
0.44
0.46
0.48
0.50
0
1000
2000
3000
4000
ChIP/chip rank (200 peak window)

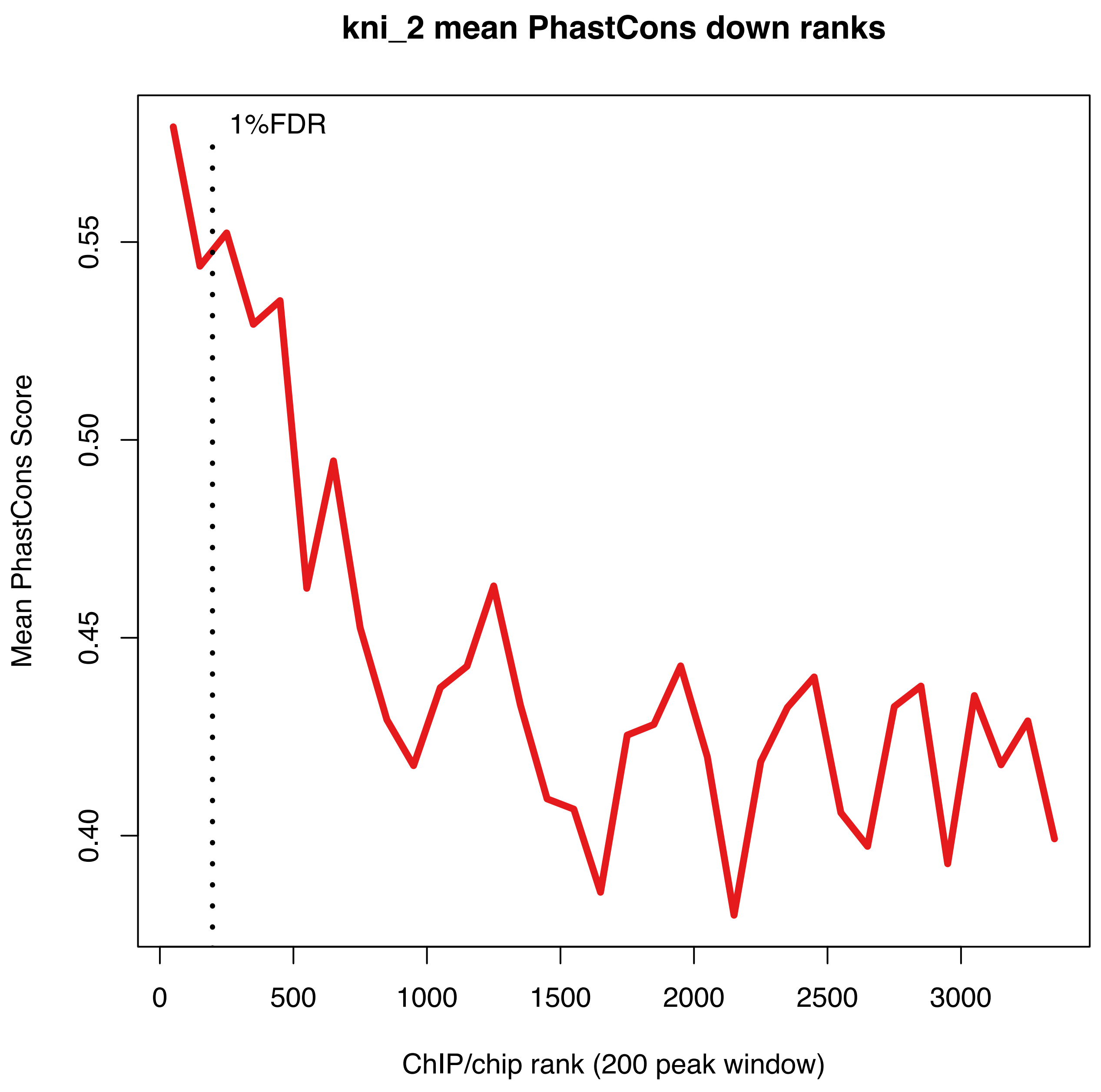
kni_2 mean PhastCons down ranks
1%FDR
Mean PhastCons Score
0.40
0.45
0.50
0.55
0
500
1000
1500
2000
2500
3000
ChIP/chip rank (200 peak window)

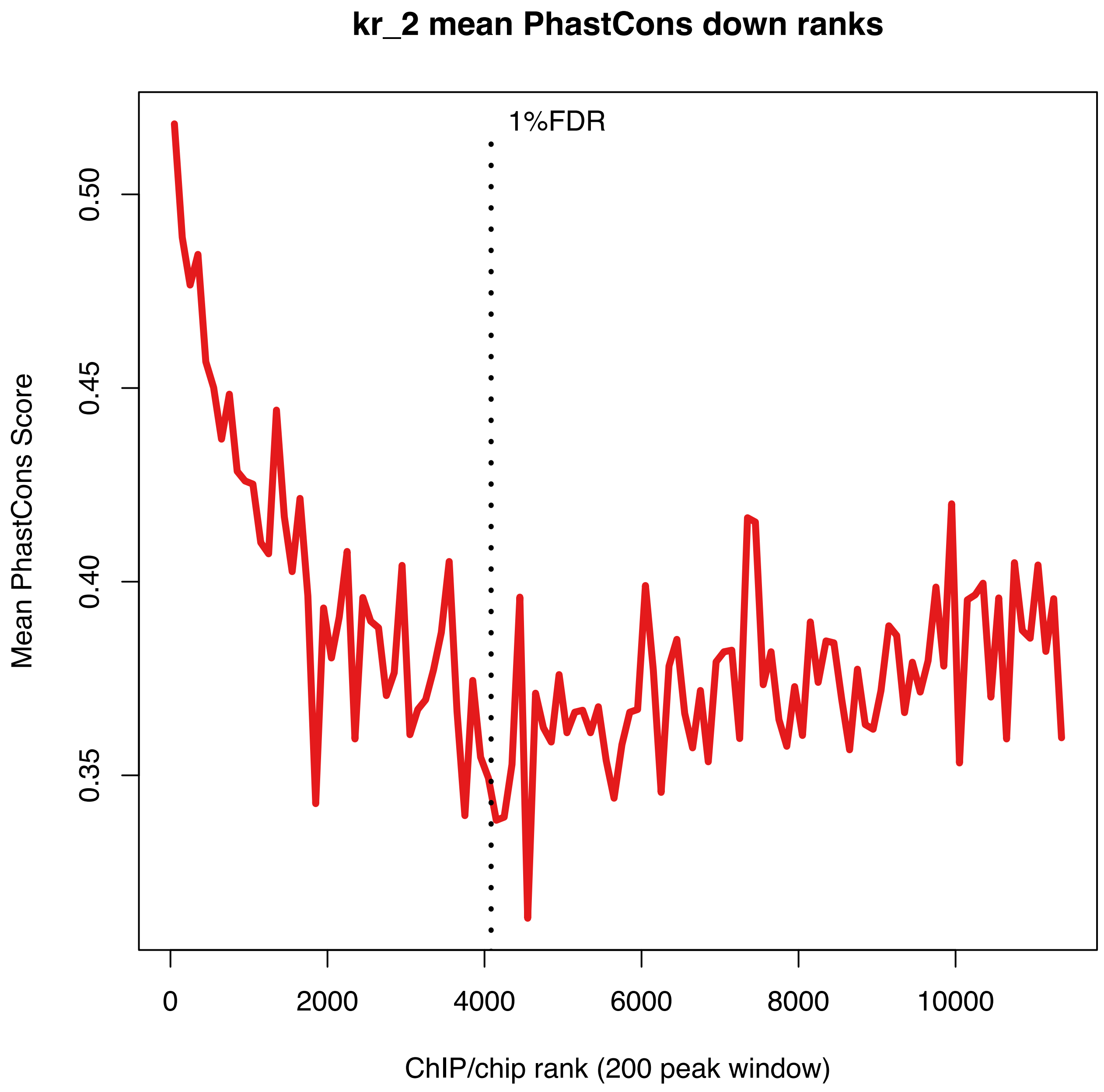
kr_2 mean PhastCons down ranks
1%FDR
Mean PhastCons Score
0.35
0.40
0.45
0.50
0
2000
4000
6000
8000
10000
ChIP/chip rank (200 peak window)

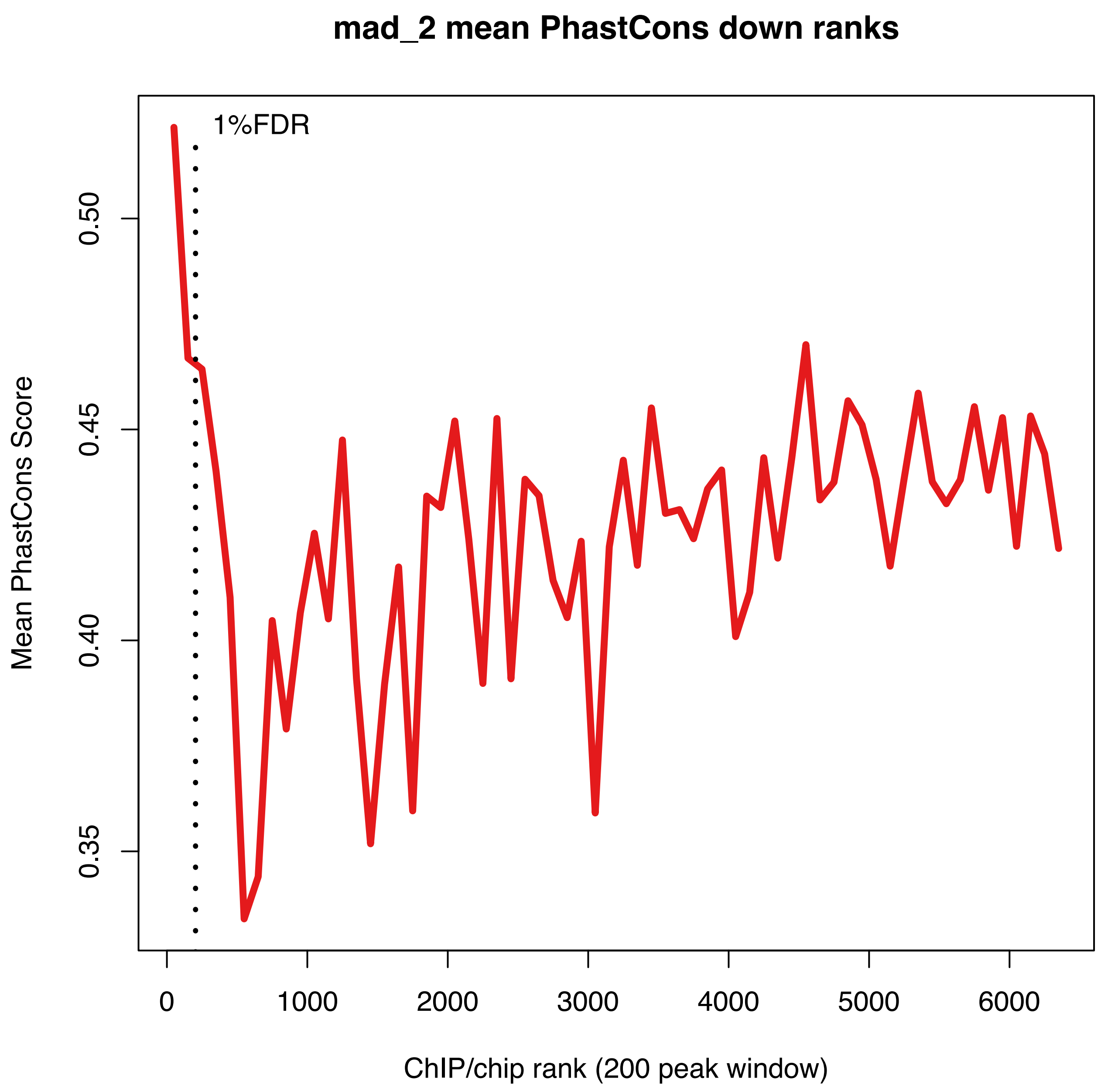
mad_2 mean PhastCons down ranks
1%FDR
Mean PhastCons Score
0.35
0.40
0.45
0.50
0
1000
2000
3000
4000
5000
6000
ChIP/chip rank (200 peak window)

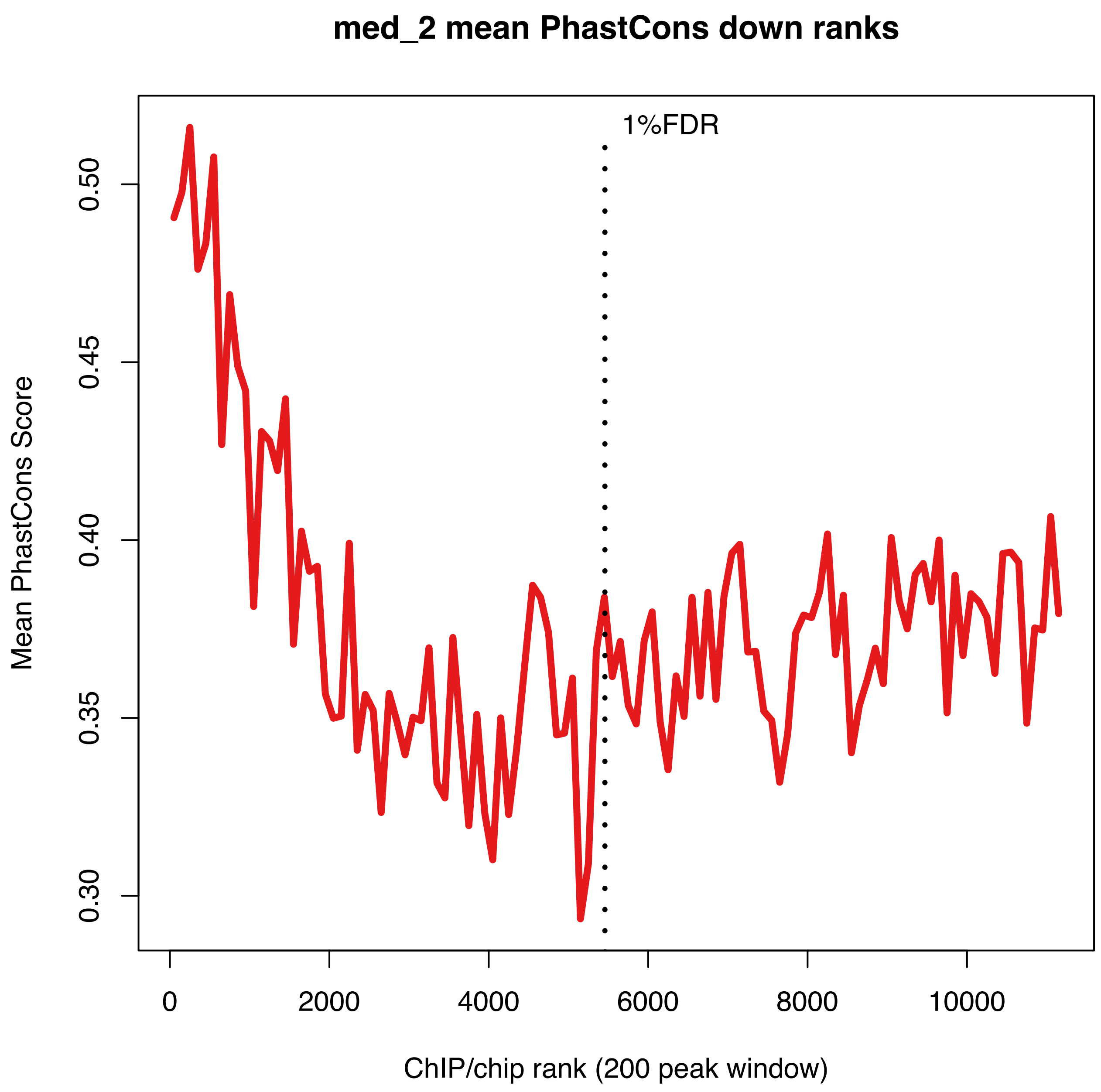
med_2 mean PhastCons down ranks
1%FDR
Mean PhastCons Score
0.30
0.35
0.40
0.45
0.50
0
2000
4000
6000
8000
10000
ChIP/chip rank (200 peak window)

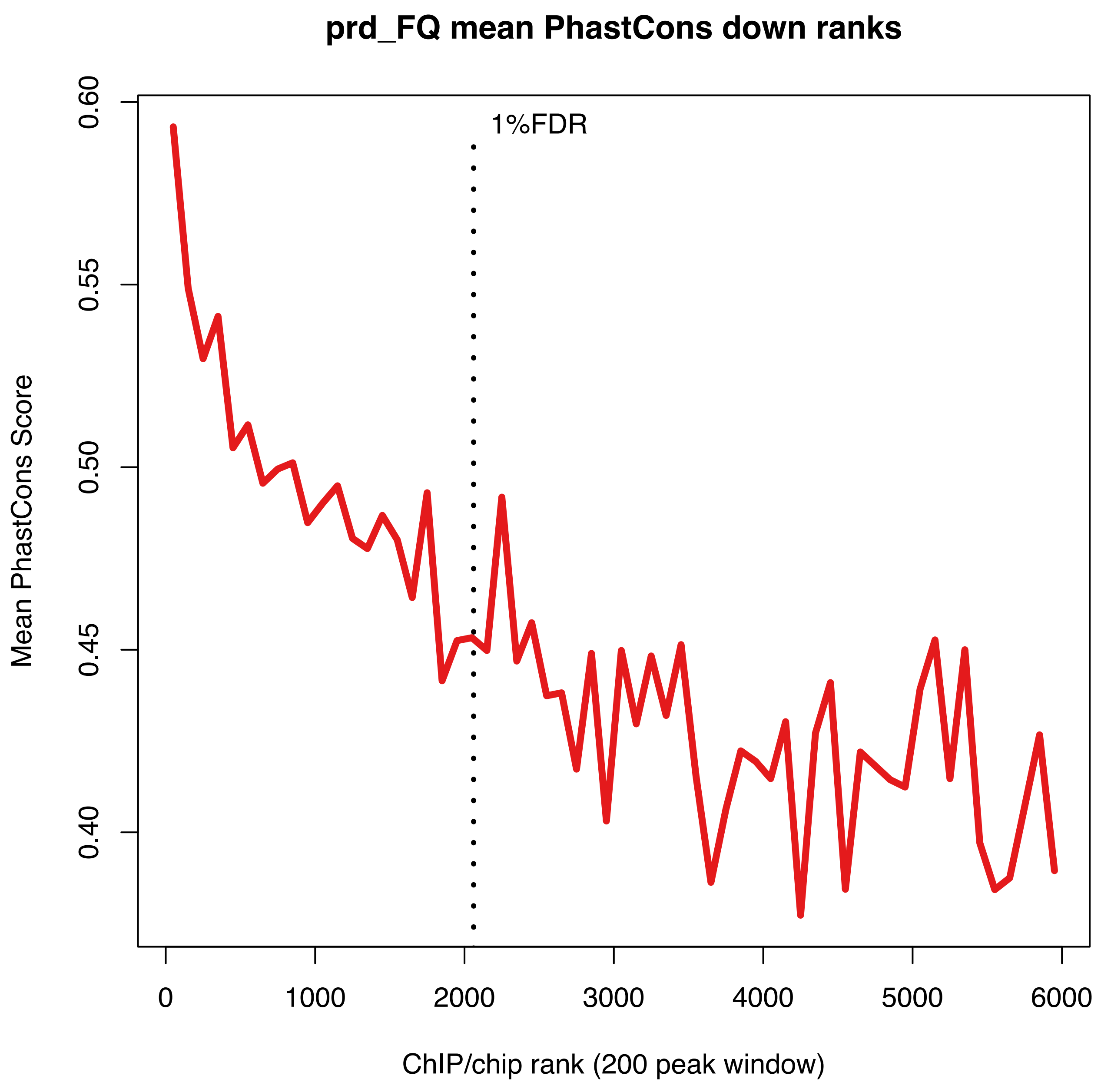
prd_FQ mean PhastCons down ranks
1%FDR
Mean PhastCons Score
0.40
0.45
0.50
0.55
0.60
0
1000
2000
3000
4000
5000
6000
ChIP/chip rank (200 peak window)

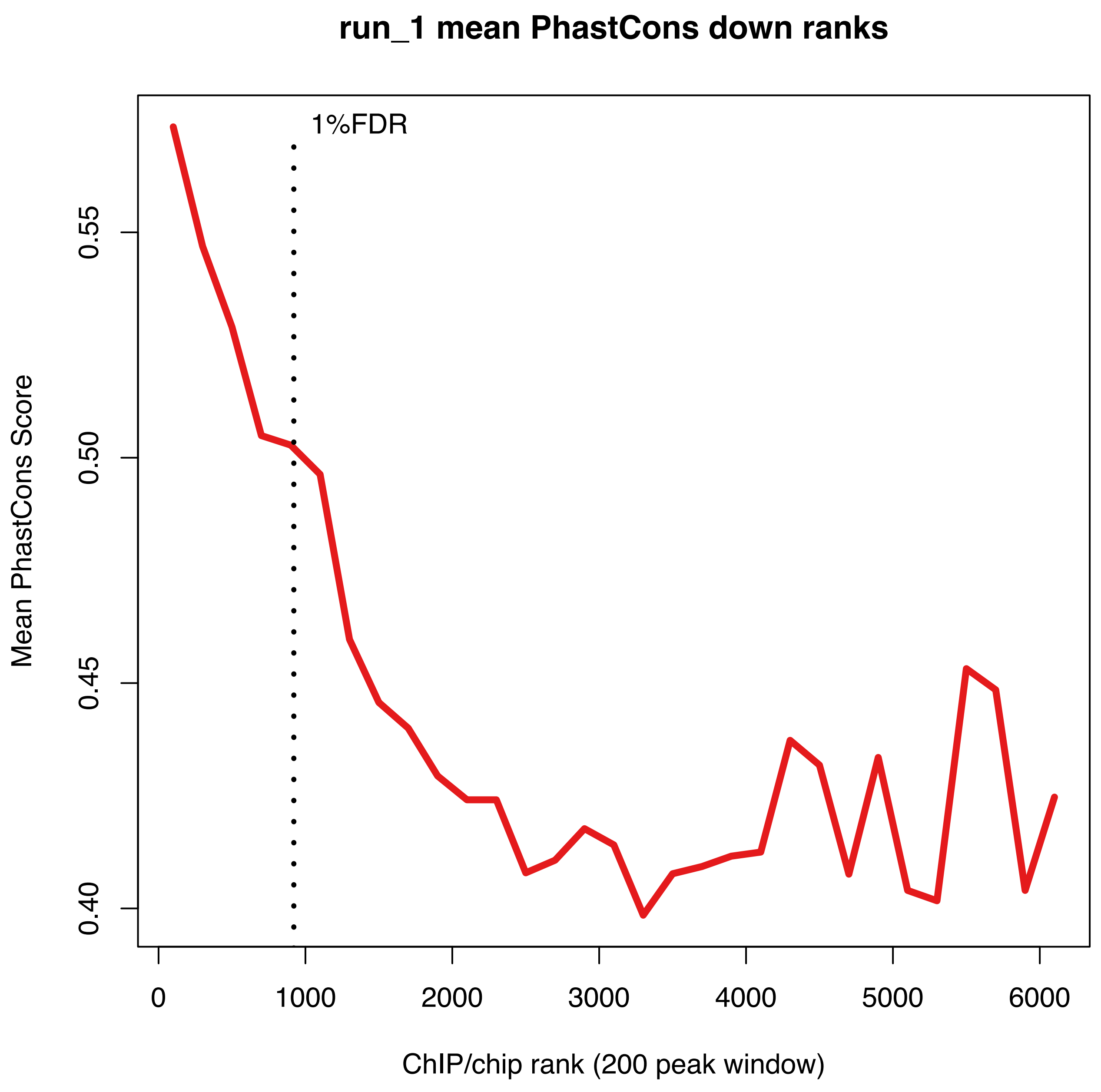
run_1 mean PhastCons down ranks
1%FDR
Mean PhastCons Score
0.40
0.45
0.50
0.55
0
1000
2000
3000
4000
5000
6000
ChIP/chip rank (200 peak window)

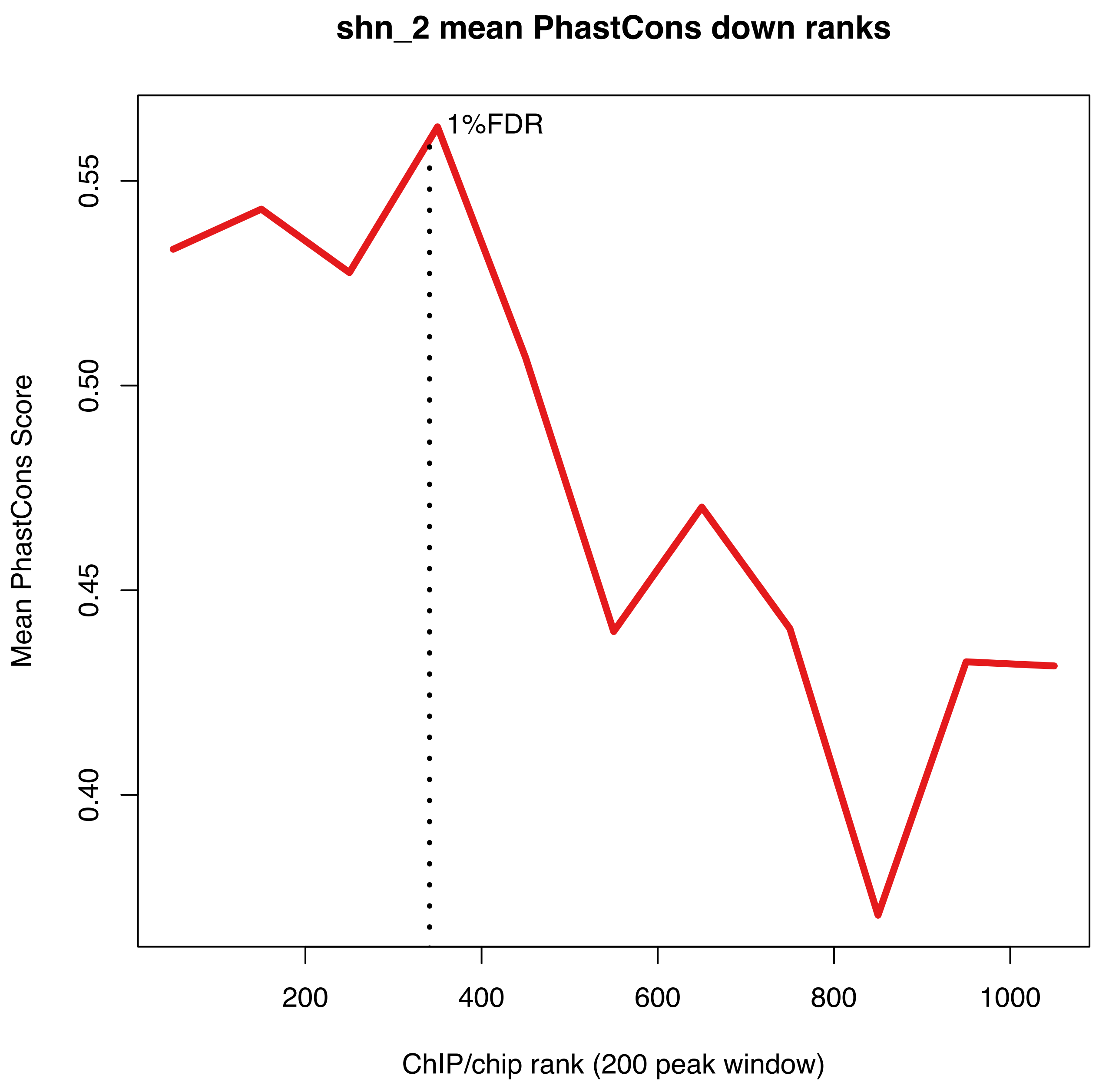
shn_2 mean PhastCons down ranks
1%FDR
0.40
0.45
0.50
0.55
Mean PhastCons Score
200
400
600
800
1000
ChIP/chip rank (200 peak window)

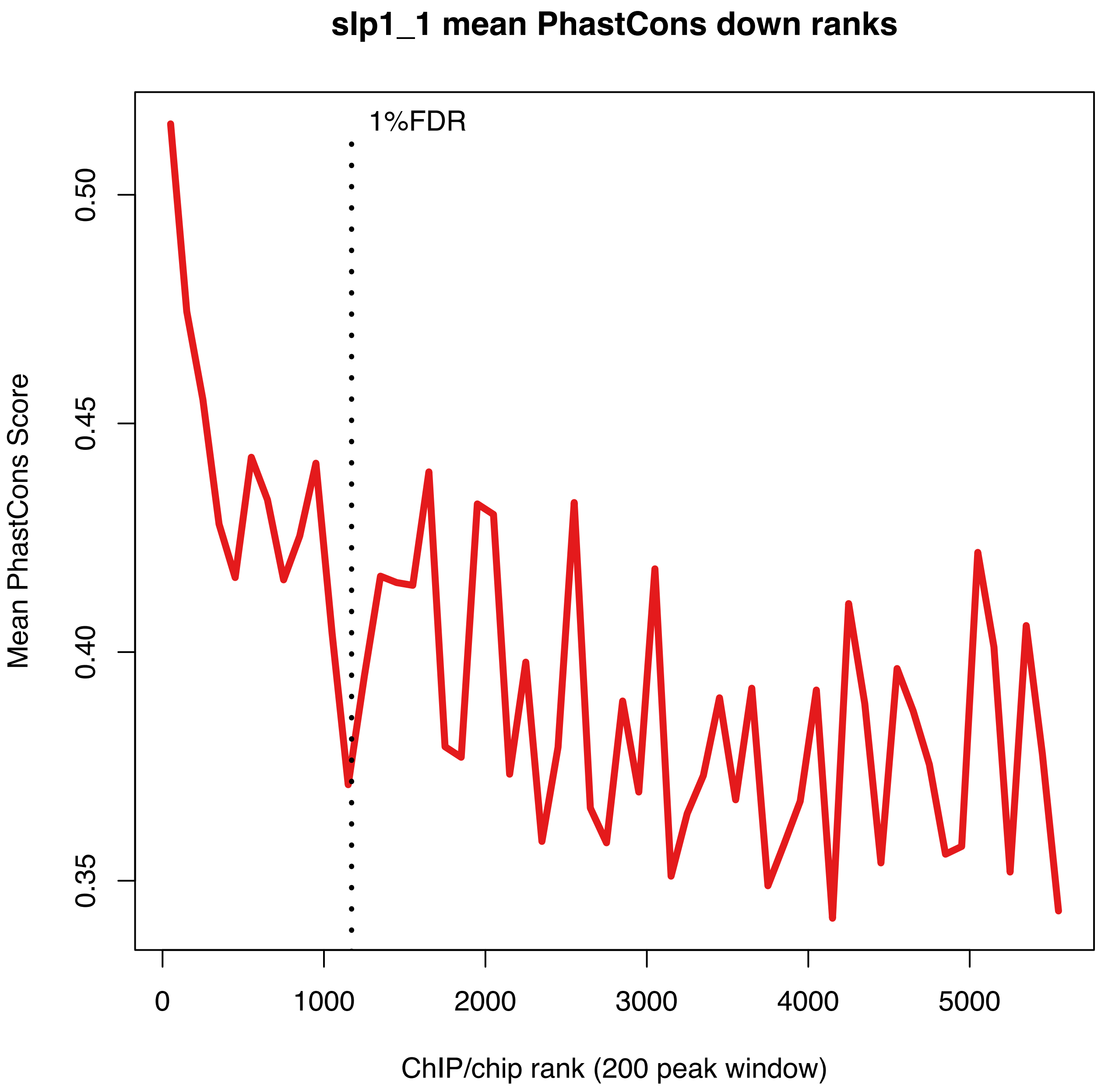
slp1_1 mean PhastCons down ranks
1%FDR
Mean PhastCons Score
0.35
0.40
0.45
0.50
0
1000
2000
3000
4000
5000
ChIP/chip rank (200 peak window)

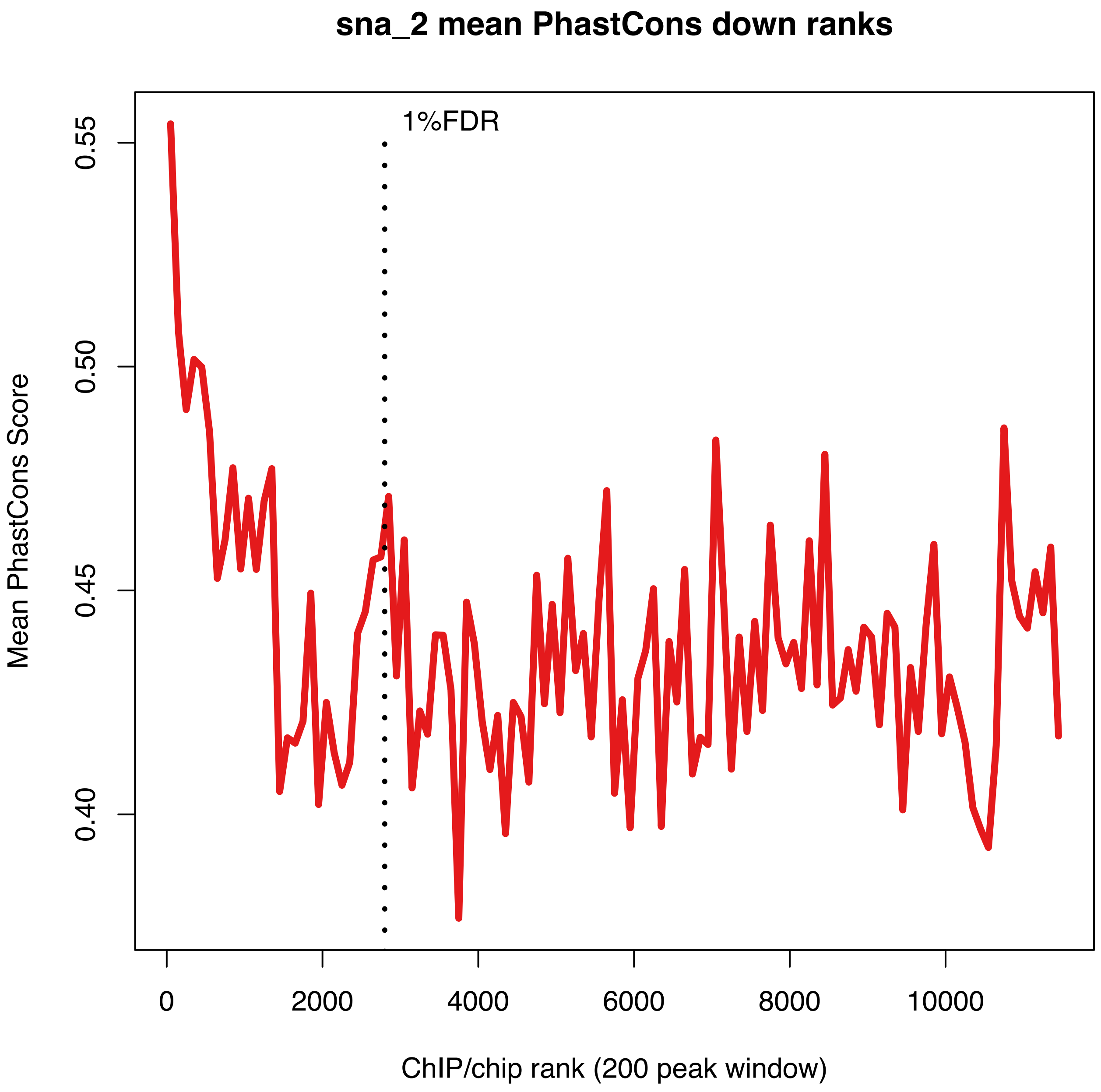
sna_2 mean PhastCons down ranks
1%FDR
Mean PhastCons Score
0.40
0.45
0.50
0.55
0
2000
4000
6000
8000
10000
ChIP/chip rank (200 peak window)

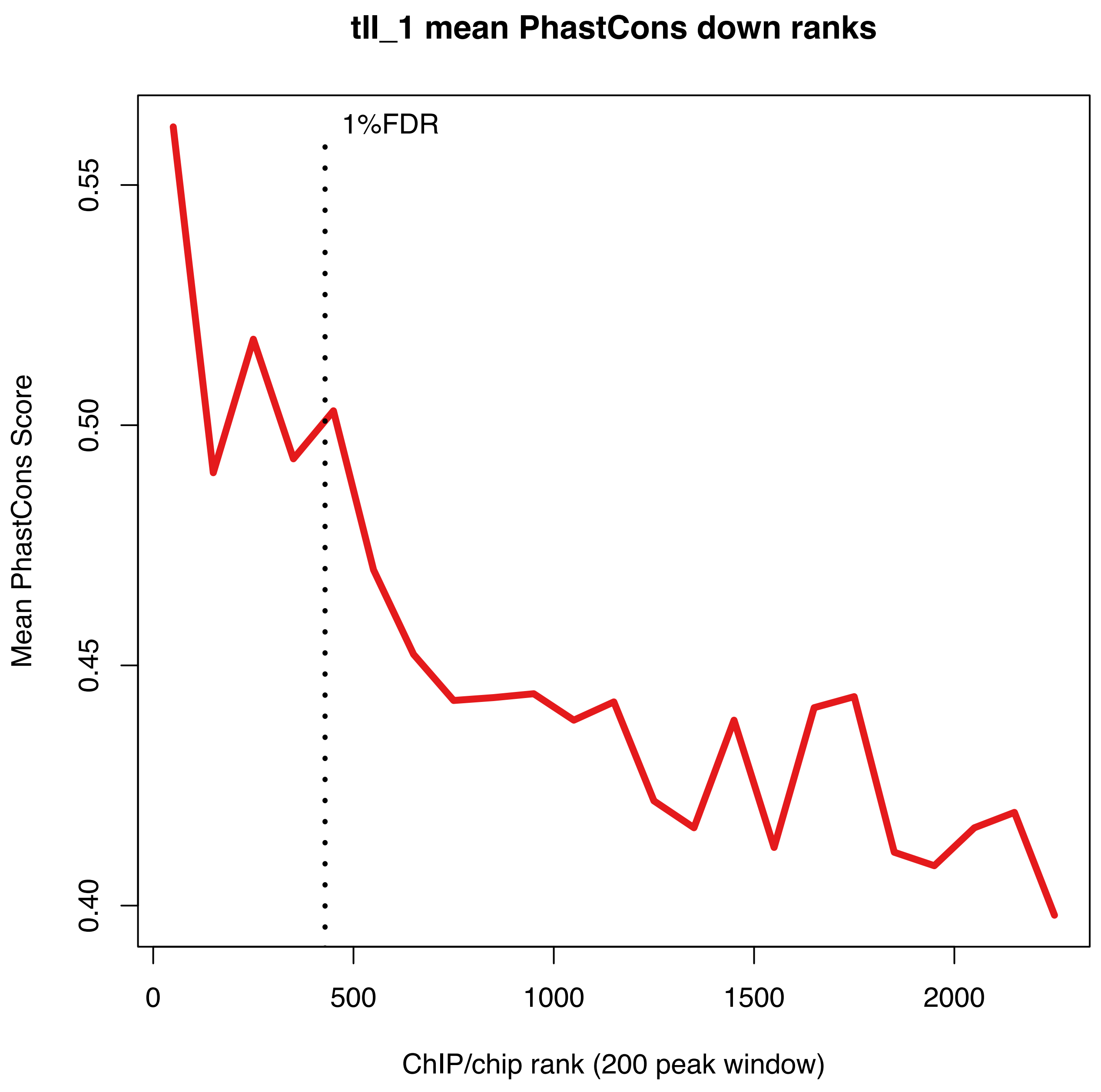
tll_1 mean PhastCons down ranks
1%FDR
Mean PhastCons Score
0.40
0.45
0.50
0.55
0
500
1000
1500
2000
ChIP/chip rank (200 peak window)

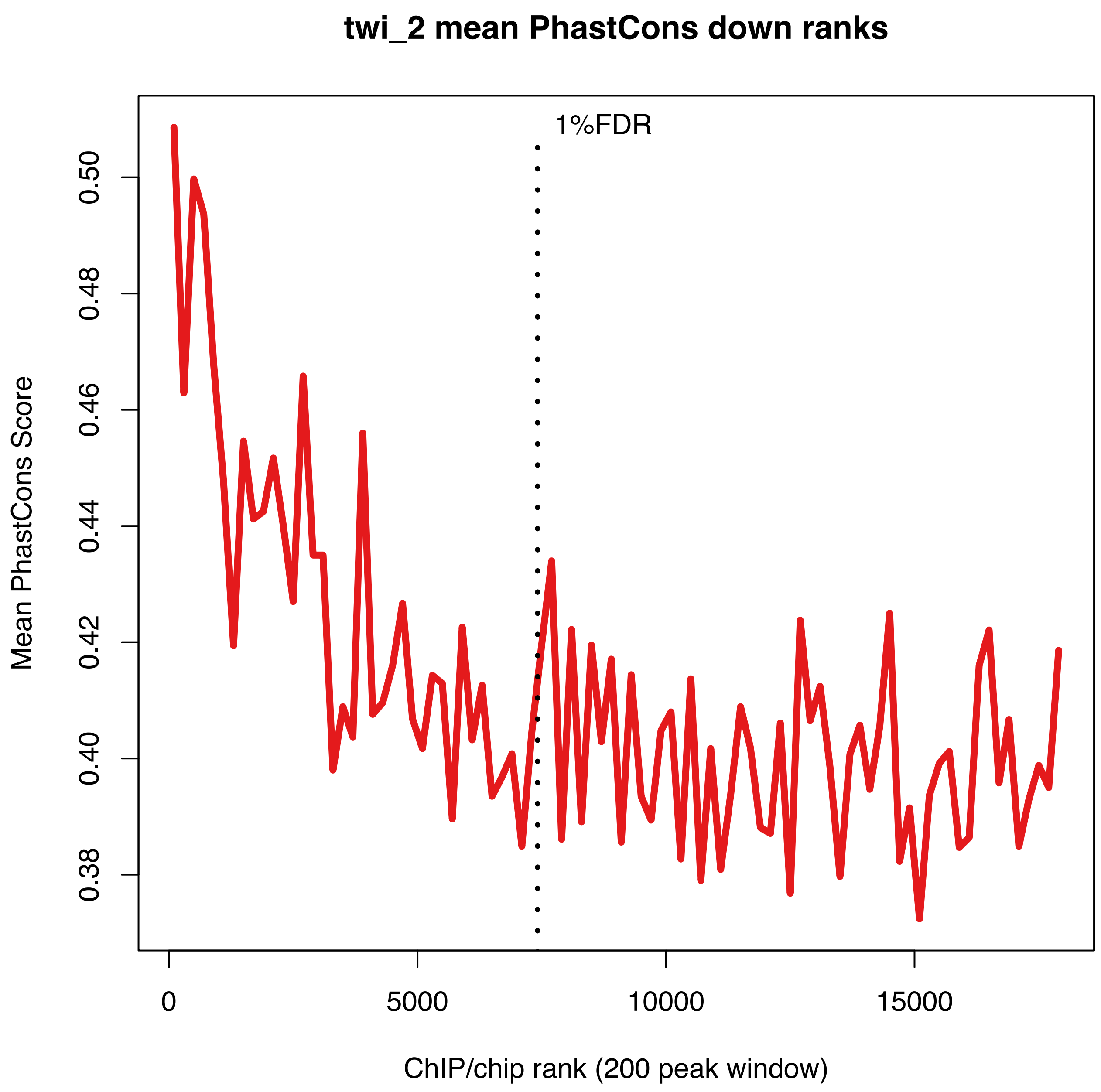
twi_2 mean PhastCons down ranks
1%FDR
Mean PhastCons Score
0.38
0.40
0.42
0.44
0.46
0.48
0.50
0
5000
10000
15000
ChIP/chip rank (200 peak window)

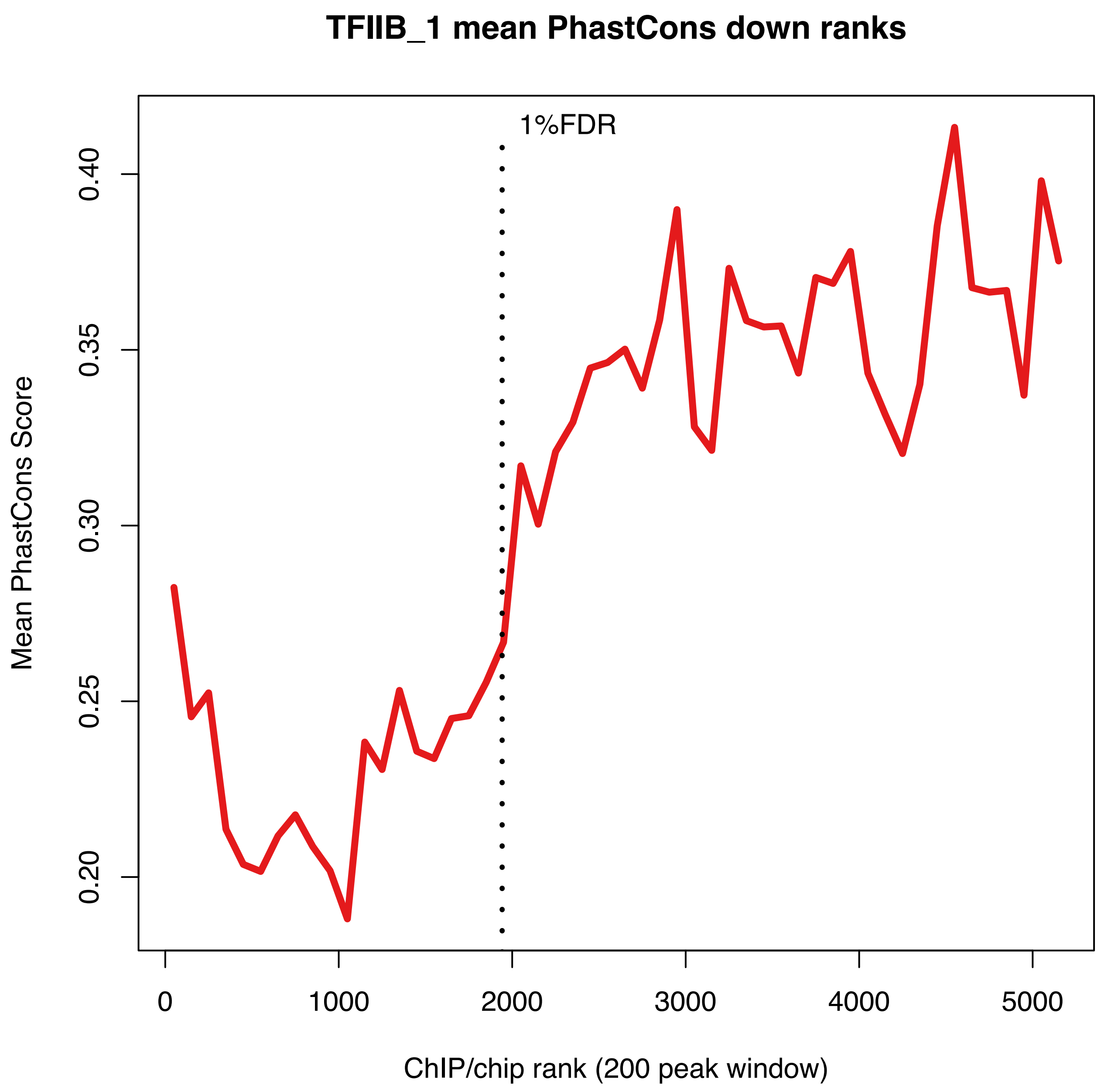
TFIIB_1 mean PhastCons down ranks
1%FDR
Mean PhastCons Score
0.20
0.25
0.30
0.35
0.40
0
1000
2000
3000
4000
5000
ChIP/chip rank (200 peak window)
