# Supplementary material for: Developmental roles of 21 Drosophila transcription factors are determined by quantitative differences in binding to an overlapping set of thousands of genomic regions
Source: Genome Biol. 2009 Jul 23;10(7):R80. doi: 10.1186/gb-2009-10-7-r80 (PMC2728534; doi:10.1186/gb-2009-10-7-r80)
Supplement: Additional data file 14 — The pattern of ChIP/chip scores on the eve gene for both factor and negative control immunoprecipitations for all antibodies shown in Table 2. [file gb-2009-10-7-r80-S14.pdf]

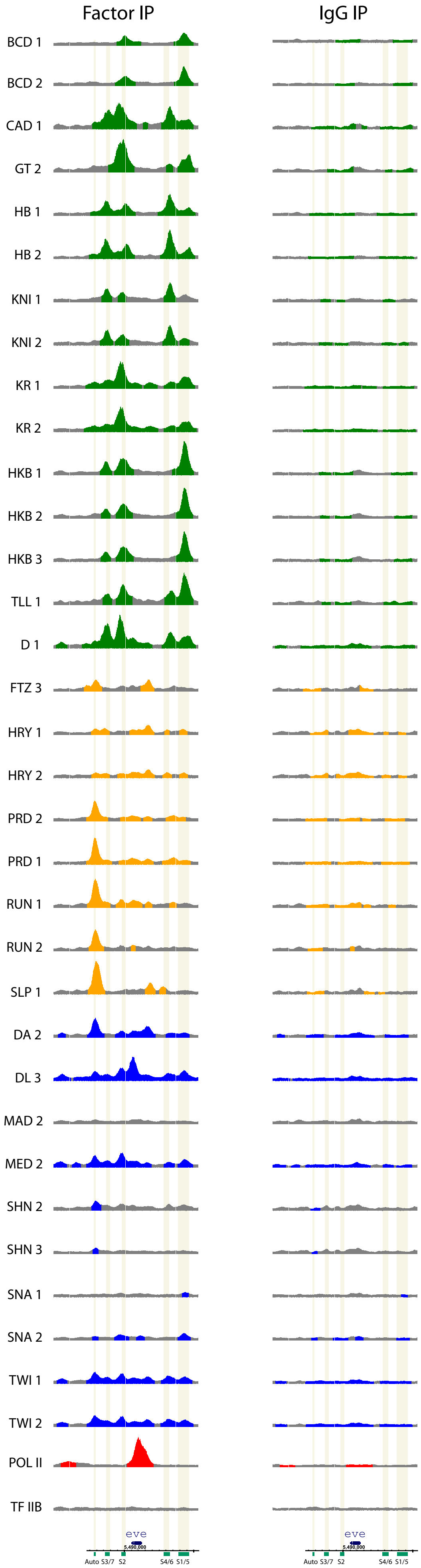

Additional Data file 14. Binding of factors to *eve* promoter sequences. Shown are the 675 bp window scores for immunoprecipitations using antibodies against portions of specific transcription factors (left, Factor IP) or for negative control immunoprecipitations carried out in parrallel with each of the corresponding factor IPs (right, IgG IP). Data for all oligonucleotides on the array are shown. The oligonucleotides with window scores above the 1% FDR threshold (using the symetric null test) in the Factor IP experiments are colored. The corresponding windows in the IgG immunoprecipitations are also colored. The locations of the five *eve* CRMs active in the blastoderm are shown. The coloring scheme for factors and the annotations of the eve gene are as described in Figure 10.

Additional ChIP/chip signal can be seen in the Factor IP (relative to the IgG IP) outside of the 1% FDR regions.
